# Supplementary material for: Stratifying Cumulus Cell Samples Based on Molecular Profiling to Help Resolve Biomarker Discrepancies and to Predict Oocyte Developmental Competence
Source: Int J Mol Sci. 2021 Jun 15;22(12):6377. doi: 10.3390/ijms22126377 (PMC8232172; doi:10.3390/ijms22126377)
Supplement: Supplementary file 1 [file ijms-22-06377-s001.zip › Suppl Table 1 Cumulus cell expression markers in different studies.pdf]

Supplementary Table 1

Reference	Materials	Stimulation protocol	ICSI/IVF	Isolation method	Number of samples	Global or selected loci	Detection methods	Markers end point measurements	Data availability	Top Markers
McKenzie *et al.* , 2004	non pooled CC	Long	ICSI	Mechanical: striping	8 patients: 108 CCs	Selected 3 genes	Quantitative PCR	morphological and physiological	None	PTGS2, HAS2, GREM1
Hasegawa *et al.* , 2007	non pooled CC	Long	ICSI	Mechanical	29 patients: 105 CCs	Selected 1 gene	Quantitative PCR	Morphology of embryo	None	Cx43
Anderson *et al.* , 2009	Individual CC	Long	ICSI	Enzymatic: cumulase enzme	75 donor: 674 CCs	Selected 6 genes	Quantitative PCR	Fertilisation, Embryo quality	None	BDNF, GREM1
Adriaenssens *et al.* , 2010	pooled CC/patient	Long	ICSI	Enzymatic: cumulase enzme	63 donor	Selected 10 genes	Quantitative PCR	Oocyte maturity	None	VCAN
Assidi *et al.* , 2011	pooled CC	Long	ICSI	Mechanical: scalpel	8 patients	Genomic + selected	Custom + OneArray	Clinical pregnancy	None	260/231 over/under expressed (list not provided)
Wathlet *et al.* , 2011	non pooled CC	Long+Short (17+25)	ICSI	Enzymatic: cumulase enzme	42 patients	Selected 6 genes	Quantitative PCR	Oocyte maturity morphology; pregnancy	None	PTGS2, VCAN, CALM2, SDC4, TRPM7, ITPKA, SDC4
Feuerstein *et al.* , 2012	non pooled CC	Long	ICSI	Enzymatic: strippers + hyaluronidase	106 patients: 197 CCs	Genomic	Microarray	Development competance to blastocyst	GSE37277	175/133 up/down regulated
Wathlet *et al.* , 2013	non pooled CC	Long	ICSI	Enzymatic: cumulase enzme	47 patients	Selected 12 genes	Quantitative PCR	Live birth, pregnancy, inter-patient analysis	None	GSR, PGR, EFNB2, CAMK1D, GSTA4, STC1, GSTA3, GPX3, THBS1, SLC2A1, ITPR1, TGFB1
Li *et al.* , 2014	pooled CC/patient	Long	ICSI	Enzymatic: cumulase enzme	196 patients: 2426 CCs	Selected 2 genes	Quantitative PCR	Morphology of embryo, pregnancy	None	GDF9, BMP15
Assidi *et al.* , 2015	pooled CC	Long	ICSI	Mechanical: scalpel	7 patients	Genomic + selected	Custom Arrays	Clinical pregnancy	None	List below in case A
Burnik Papler *et al.* , 2015	non pooled CC	Short	IVF	Mechanical: needle+denudation pipette	43 patients: 43 CCs	Selected 3 genes	Quantitative PCR	Clinical pregnancy	GSE55654	EFNB2
Xu *et al.* , 2015	pooled CC/patient	Long	IVF	Mechanical: striping	3 patients: 46 CCs	Genomic	Microarray	Embryo quality	http://rs.sagepub.com/	List below in case B
Borup et al. , 2016	non pooled CC	Long	IVF	Mechanical: 18G needle	60 patients: 27 CCs	Genomic	Microarray	Clinical pregnancy	E-MTAB-4012	490 Probes
Artini *et al.* , 2017	non pooled CC	Long	IVF / ICSI	Mechanical: scalpel	16 donor: 220 CCs	Selected 92 genes: PI3K/AKT	Quantitative PCR	Pregnancy outcome	None	AKT1, ARHGEF7, BCL2L1, CCND1, E2F1, HRAS, KCNH2, PIK3C2A, SHC1, SOS1 and SPP1
Green *et al.* , 2018	non pooled CC	Short	IVF	Mechanical: striping	17 patients: 34 CCs	Genomic	RNAseq	Live birth	None	List below in case C
Demiray *et al.* , 2019	non pooled CC	Long	ICSI	Mechanical: sterile pipette	10 patients: 10 CCs	Genomic	Microarray	Clinical pregnancy, Live birth	GSE113239	List below in case D
									A	48 (MOBKL1, ZNF793, RPL9, GGNBP2, PSMD6, RBMS2, RAN, KIF13B, CCAR1, CALM1, PPM1G, THOC2, HBA2, EIF4GI, FAM73A, TIE1, SYT11, SGCB, DDX51, PRRT2, PSIP1, GABARAP, RDH11, MRPS18B, SCARA5, RLIM, CASP9, CDKN1A, VPS16, HSD11B2, CALM2, HBB, GLB1, RPS4X, PANX1, PRDX2, RNF121, HSP90B1, POMP, CALU, VPS33A, ZFYVE20, RGS3, RPL37A, HDAC2, NRP1, EMP2, MALAT1) over expressed and 27 (SLC25A3, HNRNPM, CCNA2, ARFGAP3, RPL10, MT-CYB, TAX1BP1, HSPA8, CYP11A1, ENO1, FDPS, FOSB, RPL35A, LAMB1, DPH1, MT-ATP6, TNFAIP1, IQWD1, TMED5, HLA-DRA, RPL14, TTC15, RPS13, IFITM3, C20orf4, DOM3Z) underexpressed in Zona Good Pregnant
									B	719 were upregulated (eg: ITGA6, PPPIRI5B, SMARCAI, MORF4L2, ELOVL5, SEPT7, NM_019558, TRIP12, RNF103, MORF4L2, ACE2, VCAN, PPBP, RCANI, CSEIL, SNX14, MORF4L2, RPN2, NM_002141, CCL5, IARS2, RALB, STX4, ITPA, TPM3, CAND1, KDELR1, MORF4L2) in good quality embryo and 91 (eg: BAI2, EPB41L4A) were downregulated in bad quality embryo
									C	Nominal p 132 genes over expressed 80 (SLFNL1-AS1, SYT12, DNER, BCAR1, C5orf34, PHLDB1, BSND, FLJ42709, SNRNP40, HSPA1A, GPX1, IQGAP1, BIRC2, LMAN2, ESYT1, PLXNA3, CLTC, POLR3D, G0S2, ATP6V0C, TPP1, RTCA, CAPNS1, UBLCP1, MRPL53, DYNC1H1, SERPINE1, RPLP0P2, PRDX2, SAMD11, SMYD5, ZNF813, GTF3C5, APBB3, LINC00265, ARHGAP44, SPATA24, CYB5D2, MTHFSD, GALNT9, COL16A1, C3orf62, FKTN, ADK, ZNF589, ZNF2, CARS, TPRKB, CYP27A1, GPC2, BCL2, FAM117A, JAG1, FAT4, RFWD3, PNMA3, SOCS2, CLCNKB, NPPC, NEURL, TMEM136, ACTR6, GPR75, CHAC1, ONECUT1, SLC22A1, P2RX5, GNAS, SLC7A6, ZSCAN2, LOC100133985, ADORA2A, NCCRP1, RTN2, CLHC1, BEAN1, FAM186B, TMOD1, COL4A5, ADCY3.), under expressed 52 (PHC1, ZNRF2P1, KRTAP2-4, LCMT2, LOC100288748, OR11A1, OR52N1, OR2A14, LCE1A, GTSF1L, MARS2, KRTAP4-6, PPP1R3D, PCDHB5, HIST1H3H, LOC389791, LOC340073, PURG, HSP90AB2P, WWC2-AS2, KRTAP2-1, C16orf3, KRTAP2-2, TSPAN16, MESDC1, AHSP, MAMSTR, METTL20, GUCY2D, BCORL1, KRT25, RASAL2-AS1, HNRNPCL1, KLK2, HMX2, SYCE2, BREA2, NPR1, LOC79015, GPR33, MYH7, TNFSF12, LINC00466, C6orf25, TMEM190, F12, CELP, MIR7-3HG, TP53AIP1, CKMT2, SCGB1C1, GOLT1A)
									D	Clinical Pregnancy over expressed (CD7, TBX4, COL4A6, MAWBP, MUC17, WASPIP, FCRLM2, SPANXE, FLJ39502, ELA2) and under expressed (PPHLN1, ZFP57, ARHGEF4, SOX8, DKFZp686L1814, C15orf33, PRKACB, CNIH3, SSPN, DDAH1). Live birth over expressed (MYOC, NR4A3, BRF1, STXBP5L, CR1L, LDHAL6B, BTG2, AYA2, DKFZP686A01247, EPHA1, ACACB, LOC440508, DDX12, KIAA1841) and under expressed (FLJ20701, BTBD11, IGSF4, BDNF, RPS6KA1, KIAA1345)
