# Supplementary material for: Stratifying Cumulus Cell Samples Based on Molecular Profiling to Help Resolve Biomarker Discrepancies and to Predict Oocyte Developmental Competence
Source: Int J Mol Sci. 2021 Jun 15;22(12):6377. doi: 10.3390/ijms22126377 (PMC8232172; doi:10.3390/ijms22126377)
Supplement: Supplementary file 1 [file ijms-22-06377-s001.zip › Suppl Table 2 Genes sig corr both prot at p0.01.pdf]

Supplementary Table 2

PROBE_ID	SYMBOL	Estrogen (d12)	Endometriun	Lead Follice S	ZP	Lead Follicle	Spindle Prese	Estrogen slop	Endometriun	Pronuceli pat
ILMN_1792072	FUT4	NA	NA	NA	NA	NA	NA	NA	NA	0,00034058
ILMN_3247082	FAM150B	0,002102239	NA	0,00210224	NA	0,00280086	NA	NA	NA	0,00037504
ILMN_1787576	CLCNKA	0,060676964	NA	NA	NA	NA	NA	NA	NA	0,00038202
ILMN_2089752	LOC285016	0,002350558	NA	0,00235056	NA	0,00264246	NA	NA	NA	0,00048019
ILMN_3245507	LOC100133627	NA	NA	NA	NA	NA	NA	NA	NA	0,00048833
ILMN_1717099	DSCR3	NA	NA	NA	NA	NA	NA	NA	NA	0,00056245
ILMN_1713964	BTBD3	NA	NA	NA	NA	NA	NA	NA	NA	0,00059089
ILMN_1769288	LOC402560	NA	NA	NA	NA	NA	NA	NA	NA	0,00063224
ILMN_2364072	CLCNKA	0,021753159	NA	NA	NA	0,00323672	NA	NA	NA	0,00064676
ILMN_2392143	TRAF6	NA	NA	NA	NA	NA	NA	NA	NA	0,00076737
ILMN_1706502	EIF2AK2	0,001821232	NA	0,00182123	NA	0,00199739	NA	NA	NA	0,00088421
ILMN_1765222	CARS2	NA	NA	NA	NA	NA	NA	NA	NA	0,00096052
ILMN_1678680	DBR1	NA	NA	NA	NA	NA	NA	NA	NA	0,00109426
ILMN_2405297	NOTCH2	NA	NA	NA	NA	NA	NA	NA	NA	0,00131588
ILMN_1765409	STAM	NA	NA	NA	NA	NA	NA	NA	NA	0,00132192
ILMN_2131467	GNAI1	NA	NA	NA	NA	NA	NA	NA	NA	0,00134115
ILMN_2230998	TMEM186	0,001250288	NA	0,00125029	NA	NA	NA	NA	NA	0,00142417
ILMN_1677113	RNF8	NA	NA	NA	NA	NA	NA	NA	NA	0,00143427
ILMN_2374778	DUT	NA	NA	NA	NA	NA	NA	NA	NA	0,00149039
ILMN_1714444	KLF12	NA	NA	NA	NA	NA	NA	NA	NA	0,00172296
ILMN_1715680	NEIL2	NA	NA	NA	NA	NA	NA	NA	NA	0,00204248
ILMN_2334350	BTBD3	NA	NA	NA	NA	NA	NA	NA	NA	0,00220398
ILMN_2133996	SHE	NA	NA	0,00938893	NA	0,00122194	NA	NA	NA	0,00242677
ILMN_1719224	C17orf45	NA	NA	NA	NA	NA	NA	NA	NA	0,0024898
ILMN_2215211	ZNF514	NA	NA	NA	NA	NA	NA	NA	NA	0,00249959
ILMN_2394250	PLEKHA1	NA	NA	NA	NA	NA	NA	NA	NA	0,00256188
ILMN_1688464	MAP6D1	NA	NA	NA	NA	NA	NA	NA	NA	0,00263811
ILMN_1742618	XAF1	NA	NA	NA	NA	NA	NA	NA	NA	0,0026399
ILMN_1801121	SENP2	NA	NA	NA	NA	NA	NA	NA	NA	0,00278443
ILMN_1775012	BBS7	NA	NA	NA	NA	NA	NA	NA	NA	0,00279878
ILMN_3307786	L3MBTL2	NA	NA	NA	NA	NA	NA	NA	NA	0,00287977
ILMN_2083567	PHLPP2	NA	NA	NA	NA	NA	NA	NA	NA	0,00297979
ILMN_3226045	LOC728533	NA	NA	NA	NA	NA	NA	NA	NA	0,00301643
ILMN_1709233	F5	NA	NA	0,00805033	NA	0,00952148	NA	NA	NA	0,00310761
ILMN_1670490	PDPN	0,000489355	0,00048936	0,00048936	NA	3,81E-05	NA	NA	NA	0,00313535
ILMN_1815035	DENND2C	NA	NA	NA	NA	NA	NA	NA	NA	0,00318331
ILMN_3287266	LOC100133328	NA	NA	NA	NA	NA	NA	NA	NA	0,00336993
ILMN_1752741	TRIM23	NA	NA	NA	0,00644281	NA	NA	NA	NA	0,00340754
ILMN_1702946	THUMPD1	NA	NA	NA	NA	NA	NA	NA	NA	0,00374033
ILMN_1712806	AP1S1	NA	NA	NA	NA	NA	NA	NA	NA	0,00376265
ILMN_1652024	CSNK1G3	NA	NA	0,00784852	NA	NA	NA	NA	NA	0,00376873
ILMN_3238712	SNRNP25	NA	NA	NA	NA	NA	NA	NA	NA	0,00378559
ILMN_1803652	C9orf91	0,00329848	NA	0,00329848	NA	0,00209152	NA	NA	NA	0,00409526
ILMN_1739641	MTMR3	NA	NA	NA	NA	NA	NA	NA	NA	0,00422029
ILMN_1758673	SLC44A1	7,25E-06	NA	7,25E-06	NA	0,00045099	NA	0.000450993	NA	0,00422803
ILMN_1732336	RFC2	NA	NA	NA	NA	NA	NA	NA	NA	0,00425204
ILMN_1664855	PPP1R14C	NA	NA	NA	NA	NA	NA	NA	NA	0,00448588
ILMN_1660727	ENPP5	0,022643407	NA	NA	NA	NA	NA	NA	NA	0,00467782
ILMN_1778478	CCDC28B	NA	NA	NA	NA	NA	NA	NA	NA	0,00479545
ILMN_3243351	LOC646214	NA	NA	0,00760758	NA	0,00196738	NA	NA	NA	0,00479668
ILMN_1759154	PABPN1	0,025631633	NA	NA	NA	0,00134664	NA	NA	NA	0,00509367
ILMN_2415911	ENOX2	NA	NA	NA	NA	NA	NA	NA	NA	0,00514631
ILMN_1753639	MTAP	NA	NA	NA	NA	NA	NA	NA	NA	0,0053332
ILMN_2344047	ZNF398	NA	NA	NA	NA	NA	NA	NA	NA	0,00548334
ILMN_1699496	PHF21A	0,001719591	NA	0,00171959	NA	0,00200511	NA	NA	NA	0,00549946
ILMN_1686367	HSPA8	NA	NA	NA	NA	NA	NA	NA	NA	0,00570587
ILMN_1713529	SEMA6A	NA	NA	NA	NA	NA	NA	NA	NA	0,00581486
ILMN_1787539	ZNF177	NA	NA	NA	NA	NA	NA	NA	NA	0,00601107
ILMN_1663616	DNAJC7	NA	NA	NA	NA	NA	NA	NA	NA	0,00614742
ILMN_2405305	ARNTL	NA	NA	NA	NA	NA	NA	NA	NA	0,00632776
ILMN_2306565	MTX2	NA	NA	NA	NA	NA	NA	NA	NA	0,00653249
ILMN_3303965	ZC3H11B	NA	NA	NA	NA	NA	NA	NA	NA	0,00654538
ILMN_1795170	SLITRK2	NA	NA	NA	NA	NA	NA	NA	NA	0,00657713
ILMN_2334693	NARF	NA	NA	NA	NA	NA	NA	NA	NA	0,00663879
ILMN_1777322	FAM91A1	NA	NA	0,00095156	NA	NA	NA	NA	NA	0,00664369
ILMN_1789558	FAM164A	NA	NA	NA	NA	NA	NA	NA	NA	0,00669535
ILMN_1695945	MEIS2	NA	NA	NA	NA	NA	NA	NA	NA	0,00678127
ILMN_2115991	TNNT1	NA	NA	NA	NA	NA	NA	NA	NA	0,00679026
ILMN_2310703	RPS26L	NA	NA	NA	NA	NA	NA	NA	NA	0,00688509
ILMN_3235853	S1PR1	NA	NA	NA	NA	NA	NA	NA	NA	0,00699132
ILMN_1788099	LSM4	NA	NA	NA	NA	NA	NA	NA	NA	0,00705916
ILMN_2336109	L3MBTL2	0,051785762	NA	NA	NA	NA	NA	NA	NA	0,00706205

ILMN_1666325	ALG10B	NA	NA	NA	NA	NA	NA	NA	NA	0,00707068
ILMN_3209193	LOC644191	NA	NA	NA	NA	NA	NA	NA	NA	0,00713366
ILMN_2359029	C11orf17	NA	NA	NA	NA	NA	NA	NA	NA	0,0071455
ILMN_1792265	TRIM4	NA	NA	NA	NA	NA	NA	NA	NA	0,00724095
ILMN_1737146	TRAM1	NA	NA	NA	NA	NA	NA	NA	NA	0,00727596
ILMN_1707236	HCCS	0,001429459	NA	0,00142946	NA	NA	NA	NA	NA	0,00731413
ILMN_1676745	ZNF142	NA	NA	NA	NA	NA	NA	NA	NA	0,00734853
ILMN_1752988	C11orf17	NA	NA	NA	NA	NA	NA	NA	NA	0,00737034
ILMN_1671839	TAF1C	0,004269395	NA	0,0042694	NA	0,00135231	0,0051046	NA	NA	0,00739148
ILMN_1677432	SRGAP1	NA	NA	NA	NA	NA	NA	NA	NA	0,00743175
ILMN_2114876	RPL11	NA	NA	NA	NA	NA	NA	NA	NA	0,00752745
ILMN_2222750	RPL23AP7	NA	NA	0,005936	NA	0,00125771	NA	NA	NA	0,00765302
ILMN_2310589	DIABLO	NA	NA	NA	NA	NA	NA	NA	NA	0,00769291
ILMN_3237627	LOC154761	0,428976476	NA	NA	NA	NA	NA	NA	NA	0,00772472
ILMN_1661594	C2orf42	1,20E-05	NA	1,20E-05	0,00607009	0,00017693	NA	NA	NA	0,0077952
ILMN_1797668	INSM1	NA	NA	NA	NA	NA	NA	NA	NA	0,00794887
ILMN_1798081	PTPRF	NA	NA	NA	NA	NA	NA	NA	NA	0,00795522
ILMN_2257833	BBS7	0,017547358	NA	NA	NA	NA	NA	NA	NA	0,00797787
ILMN_1742521	GRB2	NA	0,89642126	NA	NA	NA	NA	NA	NA	0,00827947
ILMN_1706238	CSE1L	NA	NA	0,00531243	NA	0,00535282	NA	NA	NA	0,0084502
ILMN_1774828	VEZT	NA	NA	NA	NA	NA	NA	NA	NA	0,00853136
ILMN_2345872	SUMF2	NA	0,05632171	NA	NA	NA	NA	NA	NA	0,00855398
ILMN_3237385	NRBF2	NA	NA	NA	NA	NA	NA	NA	NA	0,00857315
ILMN_3237324	MMS19	NA	NA	NA	NA	NA	NA	NA	NA	0,00862179
ILMN_1697544	SLC25A29	NA	NA	NA	NA	NA	NA	NA	NA	0,0086442
ILMN_1738704	TRIM26	NA	NA	NA	NA	NA	NA	NA	NA	0,00865109
ILMN_1802843	PRCC	NA	NA	NA	NA	NA	NA	NA	NA	0,00867771
ILMN_1795228	ZFAND5	NA	NA	NA	NA	NA	NA	NA	NA	0,00876701
ILMN_1682197	NFXL1	NA	NA	NA	NA	NA	NA	NA	NA	0,00889299
ILMN_1784946	ORC3L	NA	NA	NA	NA	NA	NA	NA	NA	0,00891872
ILMN_1777118	INTS9	5,67E-05	NA	5,67E-05	NA	0,00057487	NA	NA	NA	0,00902263
ILMN_2129927	EXT1	NA	NA	NA	NA	NA	NA	NA	NA	0,00914709
ILMN_1845037		0,007174329	NA	0,00717433	NA	NA	NA	NA	NA	0,00933681
ILMN_2367710	PKN1	0,019635265	NA	NA	NA	NA	NA	NA	NA	0,00936287
ILMN_1676844	NR1D2	NA	NA	NA	NA	NA	NA	NA	NA	0,00948849
ILMN_1689237	BVES	NA	NA	0,00882909	NA	0,00452058	NA	0.004520579	NA	0,00982677
ILMN_1751898	C12orf4	0,010172619	NA	NA	NA	NA	NA	NA	NA	0,00992332
ILMN_1712628	GTF2IRD1	NA	NA	NA	NA	6,29E-05	NA	NA	6,29E+10	NA
ILMN_1813975	ADI1	NA	NA	NA	NA	0,00028151	NA	NA	0.000281508	NA
ILMN_1690105	STAT1	NA	0,00297209	0,00297209	NA	0,0006798	NA	NA	0.000679800	NA
ILMN_1704571	FAM53B	NA	NA	NA	NA	0,00075859	NA	NA	0.000758592	NA
ILMN_2135798	NR2C2AP	NA	0,02005408	NA	NA	0,00087761	NA	NA	0.000877610	NA
ILMN_1670218	EXOSC6	NA	0,03462456	NA	NA	0,00097409	NA	NA	0.000974087	NA
ILMN_2092664	ADSS	NA	NA	NA	NA	0,0013305	NA	NA	0.001330504	NA
ILMN_1705116	C6orf85	NA	0,0376977	NA	NA	0,00170617	NA	NA	0.001706173	NA
ILMN_1735045	A4GALT	NA	NA	NA	NA	0,00263133	NA	NA	0.002631332	NA
ILMN_1722726	RICS	NA	NA	0,00717128	NA	0,00299148	NA	NA	0.002991484	NA
ILMN_3227263	SLC22A23	NA	NA	NA	NA	0,00331397	NA	NA	0.003313964	NA
ILMN_1668179	HNRNPF	NA	0,00608169	0,00608169	NA	0,00355607	NA	NA	0.003556065	NA
ILMN_1666076	ARHGEF4	NA	0,02628616	NA	NA	0,00370675	NA	NA	0.003706754	NA
ILMN_2104877	CMPK1	NA	NA	NA	NA	0,00414423	NA	NA	0.004144226	NA
ILMN_1784300	TUBA4A	NA	NA	NA	NA	0,0050181	NA	NA	0.005018103	NA
ILMN_1727080	MYO6	NA	NA	NA	NA	0,00658976	NA	NA	0.006589764	NA
ILMN_1691053	LOC91561	NA	NA	NA	NA	0,00742041	NA	NA	0.007420405	NA
ILMN_2366490	ZNF706	NA	0,14082107	NA	NA	0,00752903	NA	NA	0.007529027	NA
ILMN_1733831	PGBD5	NA	NA	NA	NA	0,00753325	NA	NA	0.007533245	NA
ILMN_1705876	NAP1L1	NA	NA	NA	NA	0,00773319	NA	NA	0.007733193	NA
ILMN_2229032	NME6	NA	NA	NA	NA	0,00782199	NA	NA	0.007821988	NA
ILMN_1737398	PTPLAD1	NA	NA	NA	NA	0,00855452	NA	NA	0.008554519	NA
ILMN_1703487	LMO4	NA	NA	NA	NA	0,00943186	NA	NA	0.009431864	NA
ILMN_1677376	CHD7	NA	NA	NA	3,79E-05	NA	NA	N/A	N/A	NA
ILMN_1659255	RP2	NA	NA	NA	0,00549639	NA	NA	N/A	N/A	NA
ILMN_3236160	C4orf49	NA	NA	NA	0,0085432	NA	NA	N/A	N/A	NA
ILMN_1741200	RFX5	NA	0,35318555	NA	NA	NA	NA	N/A	N/A	NA
ILMN_1780132	PELI2	NA	0,52729736	NA	NA	NA	NA	N/A	N/A	NA
ILMN_1773643	SLC38A9	NA	0,55233755	NA	NA	NA	NA	N/A	N/A	NA
ILMN_1748481	TMEM199	NA	0,9479395	NA	NA	NA	NA	N/A	N/A	NA
ILMN_2376833	ZNF200	NA	NA	NA	NA	NA	0,00066414	NA	N/A	NA
ILMN_1772821	KIAA1671	NA	NA	NA	NA	NA	0,00936469	NA	N/A	NA
ILMN_1869897		NA	NA	NA	0,00123006	NA	NA	NA	N/A	NA
ILMN_1797534	RIOK1	NA	0,48814547	NA	0,00764569	NA	NA	NA	N/A	NA
ILMN_2306066	FUS	NA	NA	NA	0,00855516	NA	NA	NA	N/A	NA
ILMN_1669617	GRB10	NA	NA	NA	0,00857961	NA	NA	NA	N/A	NA
ILMN_3240754	FAM90A3	NA	NA	NA	0,00994763	NA	NA	NA	N/A	NA

ILMN_2150352	CBWD5	NA	NA	0,00574281	NA	NA	NA	NA	N/A	NA
ILMN_2194828	C16orf53	NA	0,01186878	NA	NA	NA	NA	NA	N/A	NA
ILMN_1701244	ITFG2	NA	0,02477079	NA	NA	NA	NA	NA	N/A	NA
ILMN_2412294	GNB5	NA	0,06739884	NA	NA	NA	NA	NA	N/A	NA
ILMN_2203544	OR2A9P	NA	0,07418168	NA	NA	NA	NA	NA	N/A	NA
ILMN_1725079	TSPAN31	NA	0,09364924	NA	NA	NA	NA	NA	N/A	NA
ILMN_1778836	SFRS7	NA	0,10295362	NA	NA	NA	NA	NA	N/A	NA
ILMN_2374692	WAC	NA	0,1062226	NA	NA	NA	NA	NA	N/A	NA
ILMN_1800721	VPS13A	NA	0,16913968	NA	NA	NA	NA	NA	N/A	NA
ILMN_1790537	C16orf75	NA	0,18869881	NA	NA	NA	NA	NA	N/A	NA
ILMN_2061732	YRDC	NA	0,22348639	NA	NA	NA	NA	NA	N/A	NA
ILMN_2121282	MRPS18B	NA	0,30899144	NA	NA	NA	NA	NA	N/A	NA
ILMN_2371825	AGL	NA	0,36427055	NA	NA	NA	NA	NA	N/A	NA
ILMN_1659766	BAG3	NA	0,37708156	NA	NA	NA	NA	NA	N/A	NA
ILMN_3235326	LOC388796	NA	0,40314633	NA	NA	NA	NA	NA	N/A	NA
ILMN_2055523	CSGALNACT1	NA	0,51803903	NA	NA	NA	NA	NA	N/A	NA
ILMN_1777058	HIATL1	NA	0,78140718	NA	NA	NA	NA	NA	N/A	NA
ILMN_3235379	LOC100134265	NA	0,85106983	NA	NA	NA	NA	NA	N/A	NA
ILMN_1709162	SHCBP1	0,028327302	NA	NA	NA	NA	NA	NA	N/A	NA
ILMN_1720344	NIPA2	0,637860578	NA	NA	NA	NA	NA	NA	N/A	NA
ILMN_1767612	BBS2	NA	NA	0,00014101	0,00683085	9,52E-05	NA	9,52E+10	NA	NA
ILMN_1694268	HES6	NA	NA	0,00012867	NA	9,97E-05	NA	9,97E+10	NA	NA
ILMN_1665510	ERRFI1	NA	NA	7,83E-05	NA	5,18E-05	NA	5,18E+11	NA	NA
ILMN_1787843	HSDL2	NA	NA	2,87E-05	NA	0,00010575	NA	0.000105749	NA	NA
ILMN_2350970	SOCS5	NA	NA	5,96E-05	NA	0,00011315	NA	0.000113146	NA	NA
ILMN_1761858	MID1	NA	NA	0,00045976	NA	0,00019811	NA	0.000198105	NA	NA
ILMN_1748538	ALDH1A2	NA	NA	9,60E-06	NA	0,00020508	NA	0.000205075	NA	NA
ILMN_1685496	RGS7	NA	NA	0,00027804	NA	0,00025531	NA	0.000255309	NA	NA
ILMN_1698038	FAM188B	NA	NA	0,00028993	NA	0,00029586	NA	0.000295854	NA	NA
ILMN_1657624	NSFL1C	NA	NA	0,00025307	NA	0,00038105	NA	0.000381048	NA	NA
ILMN_2230035	BBS2	NA	NA	0,00165055	NA	0,0004835	NA	0.000483499	NA	NA
ILMN_1787750	CD200	NA	NA	3,72E-05	0,00625078	0,00055637	NA	0.000556365	NA	NA
ILMN_2415144	SP110	NA	NA	0,00035882	NA	0,00073144	NA	0.000731438	NA	NA
ILMN_1742332	KCTD12	NA	NA	3,29E-05	0,00115985	0,00073299	NA	0.000732991	NA	NA
ILMN_2052208	GADD45A	NA	NA	0,00122765	NA	0,00078247	NA	0.000782469	NA	NA
ILMN_2081087	HSPA12A	NA	NA	0,00129304	NA	0,00078529	NA	0.000785291	NA	NA
ILMN_1694075	GADD45A	0,000129601	NA	0,0001296	NA	0,00085379	NA	0.000853785	NA	NA
ILMN_1684186	C1orf141	NA	NA	0,00019255	NA	0,00099171	NA	0.000991712	NA	NA
ILMN_1703016	CACNA1G	0,000106322	NA	0,00010632	NA	0,00106587	NA	0.001065873	NA	NA
ILMN_1687410	OSBPL11	NA	NA	0,00427399	NA	0,0010992	NA	0.001099196	NA	NA
ILMN_2167915	DSEL	NA	NA	0,00016513	NA	0,00110805	NA	0.001108047	NA	NA
ILMN_1869087		2,60E-05	NA	2,60E-05	NA	0,00126553	NA	0.001265533	NA	NA
ILMN_1694840	MATN2	NA	NA	0,0042576	NA	0,00127919	NA	0.001279186	NA	NA
ILMN_2234956	LEPR	NA	NA	NA	NA	0,00145385	NA	0.001453852	NA	NA
ILMN_1691444	UHRF2	NA	NA	0,00102163	NA	0,0015473	NA	0.001547298	NA	NA
ILMN_3279712	LOC642590	NA	NA	0,00830617	NA	0,00159936	NA	0.001599356	NA	NA
ILMN_1687757	AKR1C4	NA	NA	0,00012683	NA	0,00166485	NA	0.001664847	NA	NA
ILMN_1707464	MST1	NA	NA	0,00022886	NA	0,00174303	NA	0.001743031	NA	NA
ILMN_2104106	XPR1	NA	NA	0,00057478	NA	0,00188511	NA	0.001885109	NA	NA
ILMN_1798256	UPP1	NA	NA	0,00582196	NA	0,00193072	NA	0.001930720	NA	NA
ILMN_1755733	RPLP2	NA	NA	0,00955729	NA	0,00194321	NA	0.001943212	NA	NA
ILMN_1658290	C16orf68	NA	NA	0,00015382	NA	0,00202239	NA	0.002022388	NA	NA
ILMN_3260070	LOC100128266	NA	NA	1,16E-05	0,0004403	0,00210048	NA	0.002100477	NA	NA
ILMN_1746856	RAB21	NA	NA	0,00264849	NA	0,00223743	NA	0.002237428	NA	NA
ILMN_1719599	SYTL4	NA	NA	0,00280078	0,00647321	0,00227104	NA	0.002271037	NA	NA
ILMN_2081682	SMAP2	NA	NA	0,00451375	NA	0,00227286	NA	0.002272861	NA	NA
ILMN_1778242	CALM1	NA	NA	0,00034865	NA	0,0025049	NA	0.002504903	NA	NA
ILMN_1670305	SERPING1	NA	NA	0,0034498	NA	0,00257118	NA	0.002571175	NA	NA
ILMN_2184612	C3orf52	NA	NA	0,00025387	0,0030898	0,00258319	NA	0.002583189	NA	NA
ILMN_1740415	WFDC3	NA	NA	0,00015974	NA	0,00258856	NA	0.002588554	NA	NA
ILMN_1717173	ECT2	NA	NA	0,00408982	NA	0,00264511	NA	0.002645112	NA	NA
ILMN_3307930	RAN	NA	NA	0,00066399	NA	0,00268545	NA	0.002685449	NA	NA
ILMN_1670379	ANTXR1	NA	NA	0,00456809	NA	0,00283105	NA	0.002831052	NA	NA
ILMN_1653856	STS-1	NA	NA	0,00947857	NA	0,00298512	NA	0.002985123	NA	NA
ILMN_3249435	UBASH3B	NA	NA	0,00189633	NA	0,00302648	NA	0.003026480	NA	NA
ILMN_1795937	VIL2	NA	NA	0,00597441	NA	0,00303639	NA	0.003036392	NA	NA
ILMN_1732923	SIPA1L2	0,000423314	NA	0,00042331	0,00904807	0,00304807	NA	0.003048065	NA	NA
ILMN_2050813	CLGN	NA	NA	NA	NA	0,0031991	NA	0.003199099	NA	NA
ILMN_2308903	WFDC3	NA	NA	0,00022125	NA	0,0033717	NA	0.003371700	NA	NA
ILMN_1771599	PLOD2	NA	NA	0,0017122	NA	0,00342828	NA	0.003428283	NA	NA
ILMN_1714159	LUZP1	NA	NA	0,00180886	0,00624946	0,00353692	NA	0.003536919	NA	NA
ILMN_1775677	TYSND1	NA	NA	0,00057547	NA	0,00368099	NA	0.003680985	NA	NA
ILMN_2064725	METTL7B	NA	NA	0,0022963	NA	0,00380863	NA	0.003808632	NA	NA
ILMN_2169089	C18orf54	NA	NA	0,0056223	NA	0,00388089	NA	0.003880889	NA	NA

ILMN_1733956	IARS	0,000300869	NA	0,00030087	NA	0,00399577	NA	0.003995772	NA	NA
ILMN_1768812	FXYD6	NA	NA	NA	NA	0,00421851	NA	0.004218512	NA	NA
ILMN_1679133	SERPINB1	0,00075644	NA	0,00075644	NA	0,00432349	NA	0.004323488	NA	NA
ILMN_1713756	GLUD1	NA	NA	0,00242345	NA	0,00434627	NA	0.004346271	NA	NA
ILMN_1761797	CSTB	NA	NA	NA	NA	0,00437289	NA	0.004372885	NA	NA
ILMN_1814787	ICA1	NA	NA	NA	NA	0,0045989	NA	0.004598895	NA	NA
ILMN_1654099	LRIG2	NA	NA	NA	NA	0,00487766	NA	0.004877656	NA	NA
ILMN_3200921	LOC642590	NA	NA	0,00906709	NA	0,0049031	NA	0.004903098	NA	NA
ILMN_1698770	C5orf33	NA	NA	NA	NA	0,00492451	NA	0.004924508	NA	NA
ILMN_1750401	C17orf62	NA	NA	0,00020919	NA	0,00493452	NA	0.004934515	NA	NA
ILMN_1769633	CTSO	NA	NA	NA	NA	0,00495945	NA	0.004959452	NA	NA
ILMN_1784269	AASDH	NA	NA	NA	NA	0,00498499	NA	0.004984986	NA	NA
ILMN_1739222	ETV5	NA	NA	0,00035308	NA	0,00505754	NA	0.005057535	NA	NA
ILMN_1661432	NUP43	NA	NA	0,0001556	0,00864871	0,00507727	NA	0.005077267	NA	NA
ILMN_1661886	APEX1	NA	NA	0,00337906	NA	0,00515094	NA	0.005150937	NA	NA
ILMN_2183610	SERAC1	NA	NA	6,23E-05	NA	0,0053274	NA	0.005327402	NA	NA
ILMN_1678004	TMEM41B	NA	NA	0,00234304	NA	0,00537205	NA	0.005372045	NA	NA
ILMN_2146372	KCTD6	NA	NA	0,00030514	NA	0,00543758	NA	0.005437579	NA	NA
ILMN_1666933	ASH2L	NA	NA	0,00592005	NA	0,00577802	NA	0.005778020	NA	NA
ILMN_2387742	HNRNPA1L2	NA	NA	0,00483071	NA	0,0075166	NA	0.007516596	NA	NA
ILMN_1813104	GALNTL2	NA	NA	0,00054861	NA	0,00755438	NA	0.007554378	NA	NA
ILMN_1755411	RBMS2	6,86E-05	NA	6,86E-05	NA	0,00818602	NA	0.008186018	NA	NA
ILMN_2341254	STARD13	NA	NA	0,00967646	NA	0,00826893	NA	0.008268928	NA	NA
ILMN_3272378	EZR	NA	NA	NA	NA	0,00879054	NA	0.008790537	NA	NA
ILMN_1814650	TRAPPC4	NA	NA	0,00976487	NA	0,00884403	NA	0.008844027	NA	NA
ILMN_3211463	LOC644037	NA	NA	0,00559765	NA	0,00919081	NA	0.009190809	NA	NA
ILMN_1660021	M6PRBP1	NA	NA	0,00204399	NA	0,00921308	NA	0.009213078	NA	NA
ILMN_1684158	GPT2	NA	NA	0,00036475	0,00155805	0,00923728	NA	0.009237279	NA	NA
ILMN_1748770	CKAP5	NA	NA	NA	NA	0,00924547	NA	0.009245467	NA	NA
ILMN_1694847	TERF1	NA	NA	NA	NA	0,00937191	NA	0.009371912	NA	NA
ILMN_1744240	WDR67	NA	NA	0,00207529	NA	0,00981577	NA	0.009815767	NA	NA
ILMN_2066667	RRP8	NA	NA	0,00091996	NA	0,00986926	NA	0.009869258	NA	NA
ILMN_1805737	PFKP	NA	NA	NA	0,00352626	0,00998784	NA	0.009987837	NA	NA
ILMN_1695276	MAPRE2	NA	NA	0,00341403	6,27E-05	NA	NA	N/A	NA	NA
ILMN_1791511	TMEM176A	NA	NA	NA	6,40E-05	NA	NA	N/A	NA	NA
ILMN_1804945	ZNF667	NA	NA	NA	0,0003342	NA	NA	N/A	NA	NA
ILMN_1737343	FNIP1	NA	NA	NA	0,0003466	NA	NA	N/A	NA	NA
ILMN_1788213	FRAT2	NA	NA	NA	0,00044751	NA	NA	N/A	NA	NA
ILMN_1897864		NA	NA	NA	0,00046453	NA	NA	N/A	NA	NA
ILMN_1741727	QPCT	NA	NA	0,00050247	0,00054426	NA	NA	N/A	NA	NA
ILMN_1776998	DNAJA4	NA	NA	NA	0,00068219	NA	NA	N/A	NA	NA
ILMN_1745021	SLC30A1	NA	NA	NA	0,00083398	NA	NA	N/A	NA	NA
ILMN_1718863	KCNK1	NA	NA	0,00035793	0,00083651	NA	NA	N/A	NA	NA
ILMN_1654920	HNRPH3	NA	NA	NA	0,00099733	NA	NA	N/A	NA	NA
ILMN_3246004	LOC100133666	NA	NA	NA	0,00109452	NA	NA	N/A	NA	NA
ILMN_1738622	NDNL2	NA	NA	NA	0,00159527	NA	NA	N/A	NA	NA
ILMN_1790953	TBCB	NA	NA	NA	0,00165258	NA	NA	N/A	NA	NA
ILMN_1684620	SPAG4	NA	NA	0,00444255	0,00182646	NA	NA	N/A	NA	NA
ILMN_1736510	FOXN2	NA	NA	0,00697672	0,00224595	NA	NA	N/A	NA	NA
ILMN_1695020	NEK3	NA	NA	NA	0,00256524	NA	NA	N/A	NA	NA
ILMN_3243461	RPRD1B	NA	NA	NA	0,00300668	NA	NA	N/A	NA	NA
ILMN_1685104	C9orf6	NA	NA	NA	0,00348489	NA	NA	N/A	NA	NA
ILMN_1675038	PRMT2	NA	NA	NA	0,00350747	NA	NA	N/A	NA	NA
ILMN_1785177	DNAJC14	NA	NA	NA	0,0036006	NA	NA	N/A	NA	NA
ILMN_1718907	TSHZ1	NA	NA	0,00597505	0,00366617	NA	NA	N/A	NA	NA
ILMN_1707627	TPI1	NA	NA	NA	0,00434855	NA	NA	N/A	NA	NA
ILMN_1716264	ANKRD1	NA	NA	0,00023362	0,00438798	NA	NA	N/A	NA	NA
ILMN_2071641	KCNK1	NA	NA	0,00412501	0,00453545	NA	NA	N/A	NA	NA
ILMN_1771179	CYB561	NA	NA	0,00776392	0,00465696	NA	NA	N/A	NA	NA
ILMN_1746784	SLAIN1	NA	NA	NA	0,00483749	NA	NA	N/A	NA	NA
ILMN_1752899	BCL11A	NA	NA	0,00057743	0,00488958	NA	NA	N/A	NA	NA
ILMN_2413779	SEZ6L2	NA	NA	NA	0,0050108	NA	NA	N/A	NA	NA
ILMN_3270641	HNRNPH3	NA	NA	NA	0,00514498	NA	NA	N/A	NA	NA
ILMN_1747775	STX2	NA	NA	NA	0,00581769	NA	NA	N/A	NA	NA
ILMN_1735499	DCBLD2	NA	NA	NA	0,00589666	NA	NA	N/A	NA	NA
ILMN_1698231	RRM2B	NA	NA	0,00423584	0,00600253	NA	NA	N/A	NA	NA
ILMN_1651347	SERTAD2	NA	NA	NA	0,0064883	NA	NA	N/A	NA	NA
ILMN_1764754	RAMP1	NA	NA	0,00013871	0,00706236	NA	NA	N/A	NA	NA
ILMN_1680814	EDN2	NA	NA	NA	0,00743707	NA	NA	N/A	NA	NA
ILMN_1758250	TRAFD1	NA	NA	NA	0,00744309	NA	NA	N/A	NA	NA
ILMN_1666208	C14orf106	NA	NA	NA	0,00882248	NA	NA	N/A	NA	NA
ILMN_1804396	C14orf4	NA	NA	NA	0,00888204	NA	NA	N/A	NA	NA
ILMN_1723884	ARF4	NA	NA	NA	0,00893279	NA	NA	N/A	NA	NA
ILMN_3250893	CNNM3	NA	NA	0,00436126	0,00919684	NA	NA	N/A	NA	NA

ILMN_1711102	B3GNT2	NA	NA	0,00542865	0,0096914	NA	NA	N/A	NA	NA
ILMN_1811181	FLJ20444	NA	NA	NA	0,00982201	NA	NA	N/A	NA	NA
ILMN_1729430	FBXO18	NA	NA	1,33E-05	NA	NA	NA	N/A	NA	NA
ILMN_1725791	PTPLA	NA	NA	9,95E-05	NA	NA	NA	N/A	NA	NA
ILMN_2328835	IP6K2	NA	NA	0,00024444	NA	NA	NA	N/A	NA	NA
ILMN_1745933	LOC400713	NA	NA	0,00085395	NA	NA	NA	N/A	NA	NA
ILMN_1673113	F2RL1	0,001544282	NA	0,00154428	NA	NA	NA	N/A	NA	NA
ILMN_1803510	LOC650803	NA	NA	0,00157846	NA	NA	NA	N/A	NA	NA
ILMN_1662686	USPL1	NA	NA	0,00180821	NA	NA	NA	N/A	NA	NA
ILMN_2338921	C4orf41	NA	NA	0,00204624	NA	NA	NA	N/A	NA	NA
ILMN_2381758	G3BP2	NA	NA	0,00208226	NA	NA	NA	N/A	NA	NA
ILMN_1753312	PLXDC2	NA	NA	0,00231343	NA	NA	NA	N/A	NA	NA
ILMN_1723020	MAP3K1	NA	NA	0,0024297	NA	NA	NA	N/A	NA	NA
ILMN_2041190	F2RL1	0,002466994	NA	0,00246699	NA	NA	NA	N/A	NA	NA
ILMN_1709809	NHP2L1	NA	NA	0,00253882	NA	NA	NA	N/A	NA	NA
ILMN_1691506	NGRN	NA	NA	0,00258748	NA	NA	NA	N/A	NA	NA
ILMN_1732831	CHST7	NA	NA	0,00269739	NA	NA	NA	N/A	NA	NA
ILMN_2105919	FGF2	NA	NA	0,00291724	NA	NA	NA	N/A	NA	NA
ILMN_1675693	PPP2CB	NA	NA	0,00326324	NA	NA	NA	N/A	NA	NA
ILMN_1745499	UXT	NA	NA	0,0034267	NA	NA	NA	N/A	NA	NA
ILMN_1889752		NA	NA	0,00368924	NA	NA	NA	N/A	NA	NA
ILMN_1755222	C9orf82	NA	NA	0,00403137	NA	NA	NA	N/A	NA	NA
ILMN_1710863	GATAD1	NA	NA	0,00406312	NA	NA	NA	N/A	NA	NA
ILMN_1754969	LMCD1	NA	NA	0,00429719	NA	NA	NA	N/A	NA	NA
ILMN_2187533	CSRNP2	NA	NA	0,00439485	NA	NA	NA	N/A	NA	NA
ILMN_1760303	PIK3R1	NA	NA	0,00470038	NA	NA	NA	N/A	NA	NA
ILMN_1683328	IP6K2	NA	NA	0,00480903	NA	NA	NA	N/A	NA	NA
ILMN_1704154	TNFRSF19	NA	NA	0,00481948	NA	NA	NA	N/A	NA	NA
ILMN_2397028	SERPINB8	NA	NA	0,00497262	NA	NA	NA	N/A	NA	NA
ILMN_1754811	FBXO38	NA	NA	0,00501649	NA	NA	NA	N/A	NA	NA
ILMN_1694213	PLEKHO1	NA	NA	0,00510759	NA	NA	NA	N/A	NA	NA
ILMN_1766560	ANKHD1-EIF4EBP3	NA	NA	0,00525569	NA	NA	NA	N/A	NA	NA
ILMN_1664012	CANT1	NA	NA	0,00528573	NA	NA	NA	N/A	NA	NA
ILMN_1790136	C20orf20	NA	NA	0,00544824	NA	NA	NA	N/A	NA	NA
ILMN_1722774	VPS72	NA	NA	0,00549408	NA	NA	NA	N/A	NA	NA
ILMN_2318685	ABHD12	NA	NA	0,00551524	NA	NA	NA	N/A	NA	NA
ILMN_2356672	EIF2B4	NA	NA	0,00558972	NA	NA	NA	N/A	NA	NA
ILMN_1654385	ASB13	NA	NA	0,00559749	NA	NA	NA	N/A	NA	NA
ILMN_1739325	LOC284023	NA	NA	0,00572404	NA	NA	NA	N/A	NA	NA
ILMN_2129349	TSSC1	NA	NA	0,00598321	NA	NA	NA	N/A	NA	NA
ILMN_1752046	SH2B3	NA	NA	0,00599614	NA	NA	NA	N/A	NA	NA
ILMN_1652464	TUBA3E	NA	NA	0,00624897	NA	NA	NA	N/A	NA	NA
ILMN_2323979	WARS2	NA	NA	0,00641088	NA	NA	NA	N/A	NA	NA
ILMN_2046730	S100A10	NA	NA	0,00707811	NA	NA	NA	N/A	NA	NA
ILMN_2311779	TMUB2	NA	NA	0,00726399	NA	NA	NA	N/A	NA	NA
ILMN_2142117	LYPLAL1	NA	NA	0,00731039	NA	NA	NA	N/A	NA	NA
ILMN_1745116	ABHD12	NA	NA	0,00744428	NA	NA	NA	N/A	NA	NA
ILMN_1686968	ZNF362	NA	NA	0,00760179	NA	NA	NA	N/A	NA	NA
ILMN_1683146	FTH1	NA	NA	0,0079471	NA	NA	NA	N/A	NA	NA
ILMN_1754121	CSK	NA	NA	0,00817634	NA	NA	NA	N/A	NA	NA
ILMN_1812461	WISP2	NA	NA	0,00824139	NA	NA	NA	N/A	NA	NA
ILMN_1689004	TNFRSF12A	NA	NA	0,00866003	NA	NA	NA	N/A	NA	NA
ILMN_1748109	PEX1	NA	NA	0,00881724	NA	NA	NA	N/A	NA	NA
ILMN_1660718	GABBR2	NA	NA	0,00895948	NA	NA	NA	N/A	NA	NA
ILMN_2311537	HMGA1	NA	NA	0,00917582	NA	NA	NA	N/A	NA	NA
ILMN_2169676	ATXN10	NA	NA	0,0093104	NA	NA	NA	N/A	NA	NA
ILMN_3210304	LOC100131139	NA	NA	0,00937991	NA	NA	NA	N/A	NA	NA
ILMN_2356991	CD47	NA	0,29691834	NA	NA	NA	NA	N/A	NA	NA
ILMN_1665033	NPR3	NA	0,53159984	NA	NA	NA	NA	N/A	NA	NA
ILMN_1779034	NADSYN1	NA	0,65201075	NA	NA	NA	NA	N/A	NA	NA
ILMN_1699545	PCSK7	NA	0,73061611	NA	NA	NA	NA	N/A	NA	NA
ILMN_2353358	LGALS8	NA	0,74854744	NA	NA	NA	NA	N/A	NA	NA
ILMN_2356654	LGALS8	NA	0,75991081	NA	NA	NA	NA	N/A	NA	NA
ILMN_1721575	VPS18	NA	0,80768644	NA	NA	NA	NA	N/A	NA	NA
ILMN_1670875	PPM1D	NA	0,81815486	NA	NA	NA	NA	N/A	NA	NA
ILMN_3242603	GTF2H2B	NA	0,82327366	NA	NA	NA	NA	N/A	NA	NA
ILMN_1792910	MNT	NA	0,83830108	NA	NA	NA	NA	N/A	NA	NA
ILMN_1804445	ATF7IP2	NA	0,84392765	NA	NA	NA	NA	N/A	NA	NA
ILMN_1768510	MAN2B2	NA	0,88234178	NA	NA	NA	NA	N/A	NA	NA
ILMN_1748840	CALB2	NA	0,89051791	NA	NA	NA	NA	N/A	NA	NA
ILMN_1748271	ATF2	NA	0,96777619	NA	NA	NA	NA	N/A	NA	NA
ILMN_2303166	MBNL3	NA	0,98572082	NA	NA	NA	NA	N/A	NA	NA
ILMN_1694233	ACYP1	NA	NA	NA	NA	NA	0,00156967	NA	NA	NA
ILMN_1743582	NUDT22	NA	NA	NA	NA	NA	0,00173926	NA	NA	NA

ILMN_1690826	TNKS1BP1	NA	NA	NA	NA	NA	0,0020035	NA	NA	NA
ILMN_1679920	LOC651894	NA	NA	NA	NA	NA	0,00210662	NA	NA	NA
ILMN_1711327	TRIM37	NA	NA	NA	0,00514288	NA	0,00220804	NA	NA	NA
ILMN_2149164	SFRP1	NA	NA	NA	NA	NA	0,00222577	NA	NA	NA
ILMN_1773935	TMEM165	0,032334886	NA	NA	NA	NA	0,00232515	NA	NA	NA
ILMN_1833478		NA	NA	NA	NA	NA	0,00275726	NA	NA	NA
ILMN_1784678	PBX1	0,000321543	NA	0,00032154	NA	0,00313501	0,00326808	NA	NA	NA
ILMN_1902658		NA	NA	NA	NA	NA	0,00329006	NA	NA	NA
ILMN_1786469	FBXO22	NA	NA	NA	NA	NA	0,00357055	NA	NA	NA
ILMN_1701134	PTEN	NA	NA	NA	NA	NA	0,0037393	NA	NA	NA
ILMN_2410772	KEAP1	NA	NA	NA	NA	NA	0,00389208	NA	NA	NA
ILMN_1765779	ADAM17	NA	NA	NA	NA	0,00320995	0,0039185	NA	NA	NA
ILMN_3216794	LOC644860	NA	NA	NA	NA	NA	0,00408745	NA	NA	NA
ILMN_2372398	ALDH5A1	NA	NA	NA	NA	NA	0,00458833	NA	NA	NA
ILMN_1672564	FMO6P	NA	NA	NA	NA	NA	0,00477597	NA	NA	NA
ILMN_1809417	LRFN4	NA	NA	0,00895628	NA	0,00121176	0,00500153	NA	NA	NA
ILMN_3235949	HIST1H3E	NA	NA	NA	NA	NA	0,00508307	NA	NA	NA
ILMN_1748831	PPP1R13B	NA	NA	NA	NA	NA	0,00562749	NA	NA	NA
ILMN_1771139	FBXO31	NA	NA	NA	NA	NA	0,00567272	NA	NA	NA
ILMN_1719646	ZIK1	NA	NA	NA	NA	NA	0,0059383	NA	NA	NA
ILMN_1674201	LOC441511	NA	NA	NA	NA	NA	0,0063021	NA	NA	NA
ILMN_2286870	CSNK1D	NA	NA	NA	NA	0,00829403	0,00661108	NA	NA	NA
ILMN_1783805	PNMA3	NA	NA	NA	NA	NA	0,00673994	NA	NA	NA
ILMN_1721116	USP10	NA	NA	NA	NA	0,00611299	0,00690028	NA	NA	NA
ILMN_1707312	NFIL3	NA	NA	0,00020704	0,00655351	0,0004962	0,00702793	NA	NA	NA
ILMN_1708095	PANK2	NA	NA	0,0075898	NA	0,00032477	0,00722364	NA	NA	NA
ILMN_1790282	NUDT19	NA	NA	NA	NA	NA	0,00734457	NA	NA	NA
ILMN_1771135	ZKSCAN2	NA	NA	NA	NA	NA	0,00751044	NA	NA	NA
ILMN_1802355	RBM18	NA	NA	NA	NA	NA	0,00778935	NA	NA	NA
ILMN_1791119	NDUFA10	NA	NA	NA	NA	NA	0,00780974	NA	NA	NA
ILMN_2108339	THUMPD1	NA	NA	NA	NA	NA	0,00801984	NA	NA	NA
ILMN_1787251	DAAM1	0,015250334	NA	NA	NA	NA	0,00811805	NA	NA	NA
ILMN_2054213	MPHOSPH8	NA	NA	NA	NA	NA	0,00824215	NA	NA	NA
ILMN_1693107	MLH3	NA	NA	0,00295888	NA	NA	0,00834287	NA	NA	NA
ILMN_1784410	UBP1	NA	NA	NA	NA	NA	0,00871771	NA	NA	NA
ILMN_1787511	THUMPD2	NA	NA	NA	NA	NA	0,00874607	NA	NA	NA
ILMN_2366719	NSFL1C	NA	NA	0,00278595	NA	0,00356285	0,00889586	NA	NA	NA
ILMN_1686516	CUGBP1	NA	NA	0,00591534	NA	NA	0,0094136	NA	NA	NA
ILMN_2103591	MORC2	NA	NA	NA	NA	NA	0,00952579	NA	NA	NA
ILMN_1695961	CLK3	NA	NA	NA	NA	NA	0,00952875	NA	NA	NA
ILMN_1742544	MEF2C	NA	NA	NA	NA	NA	0,00955953	NA	NA	NA
ILMN_1678087	MAP3K4	NA	NA	NA	NA	NA	0,00964623	NA	NA	NA
ILMN_1745282	RAGE	NA	NA	NA	NA	NA	0,00965536	NA	NA	NA
ILMN_1690546	PPP3CC	NA	NA	0,00028302	NA	7,15E-06	NA	NA	NA	NA
ILMN_1730054	GSTT1	1,89E-07	NA	1,89E-07	NA	1,03E-05	NA	NA	NA	NA
ILMN_2383611	PTPRE	NA	NA	0,00025645	NA	1,34E-05	NA	NA	NA	NA
ILMN_2362581	FNDC3A	1,15E-05	NA	1,15E-05	NA	1,41E-05	NA	NA	NA	NA
ILMN_2344373	MVP	6,98E-07	NA	6,98E-07	NA	1,91E-05	NA	NA	NA	NA
ILMN_1734543	PTPRE	NA	NA	0,00103951	NA	1,94E-05	NA	NA	NA	NA
ILMN_1776653	SCML1	4,44E-06	NA	4,44E-06	NA	2,36E-05	NA	NA	NA	NA
ILMN_1787657	CLDN12	6,81E-05	NA	6,81E-05	NA	2,54E-05	NA	NA	NA	NA
ILMN_1674985	TMEM51	NA	NA	3,80E-05	NA	3,76E-05	NA	NA	NA	NA
ILMN_3307772	PACSIN2	NA	NA	0,00227026	NA	3,77E-05	NA	NA	NA	NA
ILMN_1729288	C1QTNF6	NA	NA	4,07E-05	NA	3,83E-05	NA	NA	NA	NA
ILMN_1745811	TDRD3	8,91E-05	NA	8,91E-05	NA	3,98E-05	NA	NA	NA	NA
ILMN_3250032	XPR1	NA	NA	0,00032048	NA	4,44E-05	NA	NA	NA	NA
ILMN_2339835	PTGS1	0,000569353	0,00056935	0,00056935	NA	4,94E-05	NA	NA	NA	NA
ILMN_1662910	SFXN3	NA	NA	0,00031395	NA	5,12E-05	NA	NA	NA	NA
ILMN_1652309	TTC8	NA	NA	NA	NA	5,57E-05	NA	NA	NA	NA
ILMN_2376859	PDGFD	NA	NA	NA	NA	5,66E-05	NA	NA	NA	NA
ILMN_1673522	MOCOS	NA	7,87E-05	7,87E-05	NA	5,77E-05	NA	NA	NA	NA
ILMN_1685441	ASAP3	NA	NA	1,88E-05	NA	7,58E-05	NA	NA	NA	NA
ILMN_1664602	LOC143543	NA	NA	0,00019532	NA	8,27E-05	NA	NA	NA	NA
ILMN_1681118	CAPRIN2	NA	NA	0,00341638	NA	8,37E-05	NA	NA	NA	NA
ILMN_1770244	CBX1	NA	0,01036066	NA	NA	8,59E-05	NA	NA	NA	NA
ILMN_2337551	HIPK3	NA	NA	0,00652613	NA	9,52E-05	NA	NA	NA	NA
ILMN_1738759	PIGT	NA	NA	NA	NA	9,62E-05	NA	NA	NA	NA
ILMN_2383707	ALDH1A2	2,06E-05	NA	2,06E-05	NA	9,89E-05	NA	NA	NA	NA
ILMN_1664912	IL11RA	NA	NA	0,00243608	NA	0,00010383	NA	NA	NA	NA
ILMN_1658071	ATP1B1	NA	NA	0,0052979	NA	0,00010709	NA	NA	NA	NA
ILMN_1729216	CRYAB	NA	NA	0,00104447	NA	0,00010809	NA	NA	NA	NA
ILMN_1672589	SEMA4B	5,26E-05	NA	5,26E-05	NA	0,00011175	NA	NA	NA	NA
ILMN_1692464	FLJ20699	NA	NA	0,00033555	NA	0,00011768	NA	NA	NA	NA
ILMN_2346997	RAB23	NA	NA	0,00082734	NA	0,00011955	NA	NA	NA	NA

ILMN_2214955	CCDC127	NA	0,00235155	0,00235155	NA	0,00012166	NA	NA	NA	NA
ILMN_1678323	AASS	NA	NA	9,06E-05	NA	0,00012599	NA	NA	NA	NA
ILMN_3199609	LOC400013	2,59E-05	NA	2,59E-05	NA	0,00012794	NA	NA	NA	NA
ILMN_1775182	GSR	NA	NA	9,29E-06	NA	0,00012858	NA	NA	NA	NA
ILMN_2141157	RANBP9	NA	NA	0,00017065	NA	0,00013694	NA	NA	NA	NA
ILMN_1761941	C4orf18	NA	NA	8,25E-05	NA	0,00014151	NA	NA	NA	NA
ILMN_3200652	LOC100131308	NA	NA	0,00022711	NA	0,0001478	NA	NA	NA	NA
ILMN_1706687	KLHL5	0,000102229	NA	0,00010223	NA	0,00015077	NA	NA	NA	NA
ILMN_1671891	PID1	NA	NA	NA	NA	0,0001519	NA	NA	NA	NA
ILMN_3236765	UPLP	NA	NA	7,46E-05	NA	0,00015266	NA	NA	NA	NA
ILMN_1903914		0,001766974	NA	0,00176697	NA	0,00015714	NA	NA	NA	NA
ILMN_1791896	EBAG9	NA	0,00075364	0,00075364	NA	0,00015869	NA	NA	NA	NA
ILMN_1802377	IQCG	NA	NA	5,54E-05	NA	0,00016305	NA	NA	NA	NA
ILMN_2057573	FAM62B	NA	NA	0,00036163	NA	0,00017429	NA	NA	NA	NA
ILMN_2214910	EPHB4	NA	NA	9,99E-05	NA	0,00017769	NA	NA	NA	NA
ILMN_1735192	COLEC11	NA	NA	0,00842896	NA	0,00017886	NA	NA	NA	NA
ILMN_3282292	LOC646316	NA	NA	0,00807955	NA	0,00018426	NA	NA	NA	NA
ILMN_1794825	ALDH3A2	NA	NA	0,00325625	NA	0,00018999	NA	NA	NA	NA
ILMN_3238452	FAM21D	NA	NA	0,00255767	NA	0,00019816	NA	NA	NA	NA
ILMN_1680673	NT5DC1	0,000112499	0,0001125	0,0001125	NA	0,00020118	NA	NA	NA	NA
ILMN_1710124	CMTM8	3,45E-05	NA	3,45E-05	NA	0,00020213	NA	NA	NA	NA
ILMN_1697189	PNCK	NA	NA	5,11E-05	NA	0,00021186	NA	NA	NA	NA
ILMN_2059886	TTC38	NA	NA	0,00190704	NA	0,00022708	NA	NA	NA	NA
ILMN_1755704	PRB4	NA	NA	0,00296754	NA	0,00023219	NA	NA	NA	NA
ILMN_3230215	TOX2	NA	NA	0,00166788	NA	0,00024471	NA	NA	NA	NA
ILMN_1803277	MVP	2,09E-06	NA	2,09E-06	NA	0,00024498	NA	NA	NA	NA
ILMN_1811148	BMP2K	0,000469049	NA	0,00046905	NA	0,00024867	NA	NA	NA	NA
ILMN_1782897	CAPRIN1	NA	NA	2,66E-05	NA	0,00025106	NA	NA	NA	NA
ILMN_1794875	AGPAT9	NA	NA	0,00752368	NA	0,00025575	NA	NA	NA	NA
ILMN_1656335	RIT1	3,41E-06	NA	3,41E-06	NA	0,00025885	NA	NA	NA	NA
ILMN_1898682		7,83E-05	NA	7,83E-05	NA	0,000259	NA	NA	NA	NA
ILMN_1652244	POPDC3	NA	NA	3,75E-05	NA	0,00027514	NA	NA	NA	NA
ILMN_1771385	GBP4	NA	NA	0,00053751	NA	0,00028606	NA	NA	NA	NA
ILMN_1771734	LOC728564	0,000467055	NA	0,00046706	NA	0,00029475	NA	NA	NA	NA
ILMN_2390974	DNAJB2	NA	NA	0,00182777	NA	0,00030143	NA	NA	NA	NA
ILMN_1786050	RBBP9	NA	0,00088576	0,00088576	NA	0,00030911	NA	NA	NA	NA
ILMN_2160005	NUMA1	NA	NA	0,00031347	NA	0,00031325	NA	NA	NA	NA
ILMN_1813544	OXCT1	NA	NA	0,00032041	NA	0,00031581	NA	NA	NA	NA
ILMN_2145116	TMEM173	NA	NA	NA	NA	0,00031836	NA	NA	NA	NA
ILMN_1723846	FAM119B	1,88E-05	1,88E-05	1,88E-05	NA	0,00032098	NA	NA	NA	NA
ILMN_1813840	AOF2	NA	NA	0,00028573	NA	0,00032447	NA	NA	NA	NA
ILMN_2337336	PVRL2	0,000492294	NA	0,00049229	NA	0,00032545	NA	NA	NA	NA
ILMN_1774127	STAC	0,000898946	NA	0,00089895	NA	0,0003311	NA	NA	NA	NA
ILMN_1693338	CYP1B1	0,000249934	NA	0,00024993	NA	0,00033636	NA	NA	NA	NA
ILMN_1732725	SAPS3	2,85E-06	2,85E-06	2,85E-06	NA	0,00034051	NA	NA	NA	NA
ILMN_1898124		NA	NA	1,14E-05	NA	0,00034176	NA	NA	NA	NA
ILMN_3199647	LOC645251	NA	NA	NA	NA	0,00034982	NA	NA	NA	NA
ILMN_3274578	LOC100131381	NA	NA	0,00305021	NA	0,00035028	NA	NA	NA	NA
ILMN_1800220	KCTD3	NA	NA	0,00094336	NA	0,00036341	NA	NA	NA	NA
ILMN_2389935	FYTTD1	NA	NA	NA	NA	0,00036914	NA	NA	NA	NA
ILMN_1795822	DIS3L	NA	NA	0,00199302	NA	0,00038423	NA	NA	NA	NA
ILMN_1805996	SIN3A	NA	NA	2,31E-05	NA	0,00039086	NA	NA	NA	NA
ILMN_1676358	RALB	0,000538946	NA	0,00053895	NA	0,00041414	NA	NA	NA	NA
ILMN_1716730	FAM44B	3,60E-06	NA	3,60E-06	NA	0,00041578	NA	NA	NA	NA
ILMN_1777139	MAK16	NA	NA	0,00052041	NA	0,00044791	NA	NA	NA	NA
ILMN_2333766	HISPPD2A	NA	NA	NA	NA	0,00046052	NA	NA	NA	NA
ILMN_2087528	CPSF3	NA	NA	0,00166373	NA	0,00046809	NA	NA	NA	NA
ILMN_3298423	TOX2	NA	NA	0,00120092	NA	0,00047608	NA	NA	NA	NA
ILMN_1701457	FAHD1	8,34E-05	NA	8,34E-05	0,00161586	0,00048757	NA	NA	NA	NA
ILMN_2319344	APEX1	NA	NA	0,00226938	NA	0,00052783	NA	NA	NA	NA
ILMN_1749521	SLC35E3	3,14E-05	NA	3,14E-05	0,00268015	0,00053462	NA	NA	NA	NA
ILMN_3309349	SNHG8	1,46E-08	1,46E-08	1,46E-08	NA	0,00053967	NA	NA	NA	NA
ILMN_1793672	SIX5	NA	NA	0,0002431	NA	0,00054356	NA	NA	NA	NA
ILMN_2077550	RACGAP1	NA	NA	0,00675182	NA	0,00055089	NA	NA	NA	NA
ILMN_1744048	LOC652455	NA	NA	0,00491196	NA	0,0005782	NA	NA	NA	NA
ILMN_2130180	RPL13L	NA	NA	0,00083135	NA	0,00061781	NA	NA	NA	NA
ILMN_1786972	SARS	0,000389108	NA	0,00038911	NA	0,00061803	NA	NA	NA	NA
ILMN_1735155	GLB1	NA	0,01303635	NA	NA	0,00062368	NA	NA	NA	NA
ILMN_2058347	NT5DC1	NA	NA	0,00432982	NA	0,00063407	NA	NA	NA	NA
ILMN_1814315	PBXIP1	NA	NA	0,00474064	NA	0,00063581	NA	NA	NA	NA
ILMN_1798177	CHURC1	NA	0,0088122	0,0088122	NA	0,00065859	NA	NA	NA	NA
ILMN_3245752	LOC100134011	NA	NA	0,0050998	NA	0,00071061	NA	NA	NA	NA
ILMN_1705111	FNDC3A	NA	NA	0,00017615	NA	0,00071808	NA	NA	NA	NA
ILMN_1724194	NPEPL1	NA	0,02140358	NA	NA	0,00073281	NA	NA	NA	NA

ILMN_1729051	MSH6	NA	NA	0,00336697	NA	0,00075861	NA	NA	NA	NA
ILMN_2187746	EMX2	NA	NA	0,00048437	NA	0,00077023	NA	NA	NA	NA
ILMN_2161746	TRIP10	0,001328591	NA	0,00132859	NA	0,00078348	NA	NA	NA	NA
ILMN_1669940	TMEM38B	NA	NA	7,86E-05	NA	0,00079143	NA	NA	NA	NA
ILMN_2175474	MTRF1L	NA	NA	0,00325221	NA	0,00079334	NA	NA	NA	NA
ILMN_1750051	FLJ39827	NA	NA	0,00146198	NA	0,00080655	NA	NA	NA	NA
ILMN_2339266	LAMA2	4,38E-05	4,38E-05	4,38E-05	NA	0,00080764	NA	NA	NA	NA
ILMN_1710752	NAPRT1	NA	0,00403785	0,00403785	NA	0,00082224	NA	NA	NA	NA
ILMN_1762224	HPS3	NA	NA	0,00027619	NA	0,00086509	NA	NA	NA	NA
ILMN_1703178	SCG2	0,00148337	NA	0,00148337	NA	0,00087057	NA	NA	NA	NA
ILMN_3225211	LOC729742	NA	NA	0,00703773	NA	0,00087319	NA	NA	NA	NA
ILMN_1789627	Sep 05	NA	NA	0,00145855	NA	0,00088227	NA	NA	NA	NA
ILMN_2299045	BMP2K	NA	NA	0,00246227	NA	0,00088305	NA	NA	NA	NA
ILMN_1700028	C9orf156	NA	NA	0,00078997	NA	0,00088591	NA	NA	NA	NA
ILMN_1748427	ZNF239	NA	NA	0,00059511	NA	0,00090928	NA	NA	NA	NA
ILMN_1812721	LOC728014	NA	NA	0,00410332	0,00669604	0,00094937	NA	NA	NA	NA
ILMN_1733937	MMD	NA	NA	0,00086006	NA	0,00095823	NA	NA	NA	NA
ILMN_1811702	GRN	NA	NA	0,00208924	NA	0,00096713	NA	NA	NA	NA
ILMN_1672536	FBLN1	NA	NA	NA	NA	0,00097228	NA	NA	NA	NA
ILMN_1780861	LOC653506	NA	NA	0,00042291	NA	0,00097325	NA	NA	NA	NA
ILMN_1799969	SNAPIN	NA	NA	0,00385263	NA	0,00097664	NA	NA	NA	NA
ILMN_1814194	TCF4	NA	NA	0,00010351	NA	0,00098395	NA	NA	NA	NA
ILMN_1670420	METAP2	NA	NA	0,00725707	NA	0,00098768	NA	NA	NA	NA
ILMN_1792972	ZNF439	7,32E-07	7,32E-07	7,32E-07	NA	0,00101159	NA	NA	NA	NA
ILMN_1787345	FKBP11	NA	NA	0,00086812	0,0047458	0,0010254	NA	NA	NA	NA
ILMN_1728202	TMEM22	NA	NA	0,00152568	NA	0,00105455	NA	NA	NA	NA
ILMN_1798030	XPR1	NA	NA	0,00046769	NA	0,00105926	NA	NA	NA	NA
ILMN_1738383	EEF2	NA	NA	7,51E-05	NA	0,00106382	NA	NA	NA	NA
ILMN_1794740	CD151	8,75E-06	NA	8,75E-06	NA	0,0010731	NA	NA	NA	NA
ILMN_1788019	LAMA2	0,000805869	0,00080587	0,00080587	NA	0,0010816	NA	NA	NA	NA
ILMN_2325347	B3GALNT1	NA	NA	0,00253524	NA	0,0010902	NA	NA	NA	NA
ILMN_3310491	MIR1978	NA	NA	NA	NA	0,0010969	NA	NA	NA	NA
ILMN_1810922	PCNT	0,000340664	NA	0,00034066	NA	0,00109773	NA	NA	NA	NA
ILMN_2089977	FKBP9L	NA	NA	NA	NA	0,00111199	NA	NA	NA	NA
ILMN_1789338	SORBS3	NA	NA	0,00887711	NA	0,00111934	NA	NA	NA	NA
ILMN_1688775	METRNL	NA	NA	0,00059606	NA	0,00113187	NA	NA	NA	NA
ILMN_2208495	LASS5	NA	NA	2,81E-05	NA	0,00113767	NA	NA	NA	NA
ILMN_1742569	PAPD1	NA	NA	NA	NA	0,00114745	NA	NA	NA	NA
ILMN_2117171	LMO4	NA	NA	0,00161522	NA	0,00116249	NA	NA	NA	NA
ILMN_1702396	PACSIN2	NA	NA	0,00658137	NA	0,00117155	NA	NA	NA	NA
ILMN_1653115	ECH1	NA	NA	NA	NA	0,00118881	NA	NA	NA	NA
ILMN_1788689	PHIP	NA	NA	0,00032706	NA	0,00119055	NA	NA	NA	NA
ILMN_2155228	CRLF3	NA	NA	0,00265666	NA	0,00119783	NA	NA	NA	NA
ILMN_1700811	UBFD1	NA	NA	0,00379837	NA	0,00120184	NA	NA	NA	NA
ILMN_3272424	LOC100128836	NA	NA	0,00221662	NA	0,00120541	NA	NA	NA	NA
ILMN_3236680	LOC100134393	NA	NA	NA	NA	0,00121339	NA	NA	NA	NA
ILMN_1888359		NA	NA	0,00142272	NA	0,00122477	NA	NA	NA	NA
ILMN_1700541	FBLN1	NA	NA	NA	NA	0,00124208	NA	NA	NA	NA
ILMN_3306215	LOC729926	NA	NA	NA	NA	0,00124625	NA	NA	NA	NA
ILMN_2147517	CD58	NA	NA	0,00113762	NA	0,0012672	NA	NA	NA	NA
ILMN_1729987	SRC	0,001114644	NA	0,00111464	NA	0,00127062	NA	NA	NA	NA
ILMN_1687315	RXRA	NA	NA	0,00473649	NA	0,00127992	NA	NA	NA	NA
ILMN_1661342	Sep 06	NA	NA	0,00731642	NA	0,00129829	NA	NA	NA	NA
ILMN_2354381	PON2	NA	NA	NA	NA	0,00130487	NA	NA	NA	NA
ILMN_1731418	SP110	NA	NA	0,00025421	0,00303285	0,00131392	NA	NA	NA	NA
ILMN_2206953	PLCL1	NA	NA	0,00245048	NA	0,00131409	NA	NA	NA	NA
ILMN_1729179	SPATA5L1	NA	NA	7,19E-05	NA	0,00131643	NA	NA	NA	NA
ILMN_1807177	KIAA1797	NA	NA	0,00428387	NA	0,00132209	NA	NA	NA	NA
ILMN_1805796	FLYWCH2	0,013948249	NA	NA	NA	0,00133322	NA	NA	NA	NA
ILMN_1745223	CDC42EP4	NA	NA	0,00608318	NA	0,00134467	NA	NA	NA	NA
ILMN_1769926	DBN1	NA	NA	0,00276514	NA	0,00137434	NA	NA	NA	NA
ILMN_1729281	SPHK2	NA	NA	0,00142602	NA	0,00137968	NA	NA	NA	NA
ILMN_2053567	FASTKD2	NA	NA	0,00289013	NA	0,00138018	NA	NA	NA	NA
ILMN_1691143	RAB18	NA	0,00109679	0,00109679	NA	0,00140536	NA	NA	NA	NA
ILMN_1770667	HECA	NA	NA	0,00014658	NA	0,00141295	NA	NA	NA	NA
ILMN_1665877	RNF149	0,000999276	NA	0,00099928	NA	0,00143393	NA	NA	NA	NA
ILMN_1703593	BAIAP2L1	NA	NA	NA	NA	0,00143591	NA	NA	NA	NA
ILMN_2154455	ZNF330	NA	0,0076295	0,0076295	NA	0,00144931	NA	NA	NA	NA
ILMN_1682165	NT5C2	NA	NA	2,17E-05	NA	0,00148734	NA	NA	NA	NA
ILMN_2358560	TIAM2	NA	NA	0,00958748	NA	0,00150258	NA	NA	NA	NA
ILMN_1708016	C20orf108	NA	NA	NA	NA	0,00150305	NA	NA	NA	NA
ILMN_1776347	TCP1	NA	NA	0,00314395	NA	0,00154027	NA	NA	NA	NA
ILMN_2384807	LRRCC1	NA	NA	0,00085419	NA	0,00154369	NA	NA	NA	NA
ILMN_1665049	SPG11	NA	NA	0,00011152	NA	0,0015579	NA	NA	NA	NA

ILMN_1764177	JARID2	NA	NA	NA	NA	0,00156492	NA	NA	NA	NA
ILMN_2186137	RRAD	0,003200815	0,00320082	0,00320082	NA	0,00158286	NA	NA	NA	NA
ILMN_2053345	KCNT2	NA	NA	0,0037085	NA	0,00158654	NA	NA	NA	NA
ILMN_2319077	FAS	NA	NA	9,55E-06	0,00414024	0,00158903	NA	NA	NA	NA
ILMN_1752299	RAB6B	NA	NA	0,00479445	NA	0,00160798	NA	NA	NA	NA
ILMN_1731224	PARP9	0,000276283	NA	0,00027628	NA	0,00161362	NA	NA	NA	NA
ILMN_1717046	MOBKL2B	NA	NA	0,00291053	NA	0,00161695	NA	NA	NA	NA
ILMN_2401927	TTC8	NA	NA	NA	NA	0,00162356	NA	NA	NA	NA
ILMN_3248026	RBM27	NA	NA	0,00048005	NA	0,00164023	NA	NA	NA	NA
ILMN_1667417	RAB23	NA	NA	0,00618621	NA	0,0016663	NA	NA	NA	NA
ILMN_1794490	FLJ10781	NA	NA	0,00791337	NA	0,00166646	NA	NA	NA	NA
ILMN_1746257	DAZAP1	NA	NA	0,0051873	NA	0,0016687	NA	NA	NA	NA
ILMN_1710937	IFI16	NA	NA	NA	NA	0,00167926	NA	NA	NA	NA
ILMN_1800942	KCTD6	NA	NA	0,00038498	NA	0,00168052	NA	NA	NA	NA
ILMN_2246661	FAHD1	6,94E-05	NA	6,94E-05	0,00453034	0,00168322	NA	NA	NA	NA
ILMN_2215640	TUBA3D	NA	NA	0,00295302	NA	0,00168485	NA	NA	NA	NA
ILMN_1703754	CEP192	NA	NA	0,00291897	NA	0,0017028	NA	NA	NA	NA
ILMN_1792978	HAS2	NA	NA	0,00242306	NA	0,00170746	NA	NA	NA	NA
ILMN_1712505	KDELC1	NA	NA	NA	NA	0,0017264	NA	NA	NA	NA
ILMN_1679640	FXR1	NA	NA	0,00785943	NA	0,00173323	NA	NA	NA	NA
ILMN_2400922	OPRL1	NA	NA	0,00098164	NA	0,0017347	NA	NA	NA	NA
ILMN_1790782	MED16	0,000728547	NA	0,00072855	NA	0,0017347	NA	NA	NA	NA
ILMN_1762095	TMTC4	NA	NA	0,00114816	NA	0,00173505	NA	NA	NA	NA
ILMN_1757646	UFM1	NA	NA	0,00010272	0,00260106	0,00174622	NA	NA	NA	NA
ILMN_1710514	BCL3	NA	NA	0,00025055	0,0044457	0,00175773	NA	NA	NA	NA
ILMN_1815184	ASPM	NA	NA	NA	NA	0,00176163	NA	NA	NA	NA
ILMN_2097858	KIAA1737	NA	0,0224079	NA	NA	0,00184453	NA	NA	NA	NA
ILMN_1662989	PRB2	NA	NA	0,00095885	NA	0,00184717	NA	NA	NA	NA
ILMN_2189605	FAM122B	NA	NA	0,0064259	NA	0,00185878	NA	NA	NA	NA
ILMN_1759792	CLIP4	NA	NA	0,00245841	NA	0,00189009	NA	NA	NA	NA
ILMN_1738407	ULBP1	NA	NA	0,00186552	NA	0,00189309	NA	NA	NA	NA
ILMN_1672124	C4orf18	NA	NA	0,00383668	NA	0,00192196	NA	NA	NA	NA
ILMN_1812355	WDR42C	NA	NA	NA	NA	0,00198669	NA	NA	NA	NA
ILMN_2351930	TRIM33	NA	NA	0,00290906	NA	0,00199495	NA	NA	NA	NA
ILMN_1696532	RBBP5	NA	NA	NA	NA	0,00202211	NA	NA	NA	NA
ILMN_1768505	IL13RA1	NA	NA	NA	NA	0,00204437	NA	NA	NA	NA
ILMN_1665235	CRTAP	NA	0,01017403	NA	NA	0,00204498	NA	NA	NA	NA
ILMN_1726108	LASS2	NA	NA	0,00012322	NA	0,0020509	NA	NA	NA	NA
ILMN_1728983	NR2C1	NA	NA	0,00076573	NA	0,00206931	NA	NA	NA	NA
ILMN_1835017		NA	NA	0,00645582	NA	0,00208662	NA	NA	NA	NA
ILMN_1708147	TBPL1	NA	0,00185882	0,00185882	NA	0,00209122	NA	NA	NA	NA
ILMN_1753413	TRIOBP	NA	NA	NA	NA	0,00210396	NA	NA	NA	NA
ILMN_1685005	TNFRSF1A	7,15E-05	NA	7,15E-05	0,0020545	0,00210526	NA	NA	NA	NA
ILMN_1667430	DEGS1	NA	NA	0,00139751	NA	0,00213444	NA	NA	NA	NA
ILMN_2214098	BIVM	0,000132887	NA	0,00013289	0,00658087	0,00213558	NA	NA	NA	NA
ILMN_2309245	BIN1	NA	NA	0,00404665	NA	0,00213713	NA	NA	NA	NA
ILMN_1775742	RNF128	NA	NA	0,00086879	NA	0,00214196	NA	NA	NA	NA
ILMN_1696003	GNAI3	NA	0,00477308	0,00477308	NA	0,00215097	NA	NA	NA	NA
ILMN_1672128	ATF4	NA	NA	0,00149352	NA	0,00217116	NA	NA	NA	NA
ILMN_1748844	CNKSR3	NA	NA	0,00670262	NA	0,00217372	NA	NA	NA	NA
ILMN_1747423	LOC389901	NA	NA	0,00608781	NA	0,00218989	NA	NA	NA	NA
ILMN_1694877	CASP6	NA	NA	0,00257775	NA	0,00222063	NA	NA	NA	NA
ILMN_2104696	ERICH1	NA	NA	NA	NA	0,00227414	NA	NA	NA	NA
ILMN_1814077	ZFPM2	0,002446725	NA	0,00244673	NA	0,00228718	NA	NA	NA	NA
ILMN_1798940	LOC644131	NA	NA	0,0021311	NA	0,00228803	NA	NA	NA	NA
ILMN_1727592	FRMD5	NA	NA	NA	NA	0,0023048	NA	NA	NA	NA
ILMN_1742147	UBL4A	NA	NA	NA	NA	0,00231501	NA	NA	NA	NA
ILMN_1777325	STAT1	NA	NA	0,00954953	NA	0,00234026	NA	NA	NA	NA
ILMN_3307892	PARVA	NA	NA	0,00026536	NA	0,00234611	NA	NA	NA	NA
ILMN_3282436	LOC651198	NA	0,00339884	0,00339884	NA	0,00236389	NA	NA	NA	NA
ILMN_1652280	FBXO32	NA	NA	0,00066795	NA	0,00236968	NA	NA	NA	NA
ILMN_2157219	AASS	NA	NA	0,00240542	NA	0,00238428	NA	NA	NA	NA
ILMN_1773847	DYNC1I2	NA	NA	0,00427849	NA	0,00241033	NA	NA	NA	NA
ILMN_1811370	HSD11B1	NA	NA	9,37E-05	0,0034477	0,0024154	NA	NA	NA	NA
ILMN_1728298	SBK1	NA	NA	0,00750277	NA	0,00244212	NA	NA	NA	NA
ILMN_1691410	BAMBI	0,001087153	NA	0,00108715	NA	0,00245254	NA	NA	NA	NA
ILMN_1674160	BIN1	NA	NA	0,00718438	NA	0,00245284	NA	NA	NA	NA
ILMN_1807633	HRSP12	NA	NA	NA	NA	0,00246049	NA	NA	NA	NA
ILMN_1666453	STK3	NA	NA	NA	NA	0,00246827	NA	NA	NA	NA
ILMN_2317923	TMEM132A	NA	NA	0,00562968	NA	0,00248181	NA	NA	NA	NA
ILMN_3281502	LOC653375	NA	0,0007317	0,0007317	NA	0,00250908	NA	NA	NA	NA
ILMN_1654398	RGL1	NA	NA	0,00411188	NA	0,00251018	NA	NA	NA	NA
ILMN_2166831	RPS4X	0,003987291	0,00398729	0,00398729	NA	0,00251264	NA	NA	NA	NA
ILMN_1791726	TUBB3	0,003273584	NA	0,00327358	NA	0,00251318	NA	NA	NA	NA

ILMN_1673566	ADAMTS1	NA	NA	0,00159886	NA	0,00255864	NA	NA	NA	NA
ILMN_1774733	SOCS1	0,000718059	NA	0,00071806	NA	0,00260681	NA	NA	NA	NA
ILMN_2120072	FLJ13305	0,010489205	0,01048921	NA	NA	0,00266306	NA	NA	NA	NA
ILMN_1691112	PIGN	NA	NA	0,00111881	NA	0,00268395	NA	NA	NA	NA
ILMN_1806533	PDE7B	NA	NA	NA	NA	0,00269342	NA	NA	NA	NA
ILMN_1773850	FXC1	NA	NA	NA	NA	0,00272618	NA	NA	NA	NA
ILMN_1671005	IRF2BP2	0,000792904	0,0007929	0,0007929	NA	0,00273327	NA	NA	NA	NA
ILMN_1761594	FAM35A	0,001181581	NA	0,00118158	NA	0,00276013	NA	NA	NA	NA
ILMN_1702526	C17orf48	NA	NA	0,00063652	NA	0,00277078	NA	NA	NA	NA
ILMN_2214473	ARHGEF5L	NA	0,00055774	0,00055774	NA	0,0028048	NA	NA	NA	NA
ILMN_1670931	PDS5A	3,91E-05	NA	3,91E-05	NA	0,00280579	NA	NA	NA	NA
ILMN_1696485	HNRNPAB	0,000474145	0,00047415	0,00047415	NA	0,0028128	NA	NA	NA	NA
ILMN_2382354	SENP7	NA	NA	0,00225759	NA	0,00282489	NA	NA	NA	NA
ILMN_2262044	PARP10	0,000493914	0,00049391	0,00049391	NA	0,00285289	NA	NA	NA	NA
ILMN_1696087	PHB2	NA	NA	0,00738769	NA	0,00286084	NA	NA	NA	NA
ILMN_1665717	EIF2S3	NA	NA	0,00075626	NA	0,00286782	NA	NA	NA	NA
ILMN_1656840	VPS13D	0,000771064	NA	0,00077106	NA	0,00289196	NA	NA	NA	NA
ILMN_2346460	NARG2	NA	NA	NA	NA	0,00291113	NA	NA	NA	NA
ILMN_1695034	LOC642817	NA	NA	NA	NA	0,00292399	NA	NA	NA	NA
ILMN_1775759	NRAS	NA	NA	0,0012742	NA	0,00294027	NA	NA	NA	NA
ILMN_2043816	ARPC5L	NA	NA	0,00065344	NA	0,00294383	NA	NA	NA	NA
ILMN_2196569	NUP93	NA	NA	0,00129781	NA	0,00294839	NA	NA	NA	NA
ILMN_1715647	VANGL2	NA	NA	NA	NA	0,00295681	NA	NA	NA	NA
ILMN_1745497	C12orf26	NA	NA	NA	NA	0,00297394	NA	NA	NA	NA
ILMN_1753525	TCEAL7	NA	NA	0,00043647	NA	0,00298685	NA	NA	NA	NA
ILMN_1756204	RPS6KA4	0,002054165	NA	0,00205417	NA	0,00300373	NA	NA	NA	NA
ILMN_1768279	NME6	NA	NA	0,00177755	NA	0,00300562	NA	NA	NA	NA
ILMN_1761722	ZNF579	NA	NA	NA	NA	0,0030584	NA	NA	NA	NA
ILMN_1774427	CALCOCO1	NA	NA	NA	NA	0,00306367	NA	NA	NA	NA
ILMN_1695792	CUL4A	NA	NA	NA	NA	0,00307581	NA	NA	NA	NA
ILMN_1738237	HS1BP3	NA	NA	0,00856929	NA	0,00307822	NA	NA	NA	NA
ILMN_1746588	TALDO1	NA	NA	NA	NA	0,00308803	NA	NA	NA	NA
ILMN_2134555	KCTD3	NA	NA	0,00094758	NA	0,00309462	NA	NA	NA	NA
ILMN_1716006	C18orf54	0,003552294	NA	0,00355229	NA	0,00310056	NA	NA	NA	NA
ILMN_1777663	TOP2B	NA	NA	0,00416131	NA	0,00311927	NA	NA	NA	NA
ILMN_1736340	ANGEL2	NA	NA	0,00155605	NA	0,00314199	NA	NA	NA	NA
ILMN_2159322	SLK	1,13E-05	1,13E-05	1,13E-05	NA	0,00315077	NA	NA	NA	NA
ILMN_1719219	ZNF616	NA	NA	0,00827227	NA	0,00317747	NA	NA	NA	NA
ILMN_3290385	LOC401640	NA	NA	NA	NA	0,00321681	NA	NA	NA	NA
ILMN_3251511	SFRS2IP	NA	NA	0,00230547	NA	0,00324546	NA	NA	NA	NA
ILMN_1744725	BTBD6	NA	NA	0,00735628	NA	0,00326696	NA	NA	NA	NA
ILMN_3235221	LOC644936	0,076722368	0,07672237	NA	NA	0,00328132	NA	NA	NA	NA
ILMN_1769911	SLC38A1	NA	NA	0,00998503	NA	0,00329361	NA	NA	NA	NA
ILMN_1801105	PRKCD	NA	NA	0,00088968	NA	0,00331167	NA	NA	NA	NA
ILMN_1661636	ZMYM2	NA	NA	NA	NA	0,00331183	NA	NA	NA	NA
ILMN_1713688	DHX32	NA	NA	NA	NA	0,00331678	NA	NA	NA	NA
ILMN_1729217	FAM131A	NA	NA	0,00450194	NA	0,00332448	NA	NA	NA	NA
ILMN_1740490	ZFP82	NA	NA	NA	NA	0,00333398	NA	NA	NA	NA
ILMN_1790014	METRNL	NA	NA	NA	NA	0,00336271	NA	NA	NA	NA
ILMN_1691798	ZNF26	NA	NA	0,00106539	NA	0,00336642	NA	NA	NA	NA
ILMN_1759350	MED27	0,016548132	NA	NA	NA	0,00337125	NA	NA	NA	NA
ILMN_1743104	RBM4B	NA	NA	0,00095119	NA	0,0033849	NA	NA	NA	NA
ILMN_2412922	C14orf43	NA	NA	0,00095867	NA	0,00338702	NA	NA	NA	NA
ILMN_1754145	CAPRIN1	0,002358248	0,00235825	0,00235825	NA	0,00339177	NA	NA	NA	NA
ILMN_2157435	DYNLRB1	NA	NA	0,001516	NA	0,0033939	NA	NA	NA	NA
ILMN_2342437	KLHL5	0,00011628	NA	0,00011628	NA	0,00340626	NA	NA	NA	NA
ILMN_2225318	SMS	NA	NA	NA	NA	0,00341369	NA	NA	NA	NA
ILMN_2192595	PRB2	NA	NA	0,00074029	NA	0,00344373	NA	NA	NA	NA
ILMN_1797005	PGLS	NA	NA	0,0023896	NA	0,00345441	NA	NA	NA	NA
ILMN_1778360	PYGB	NA	NA	0,00301306	NA	0,00345959	NA	NA	NA	NA
ILMN_1667564	ALDH3A2	NA	NA	0,00355014	NA	0,00346549	NA	NA	NA	NA
ILMN_1651513	SKIV2L2	NA	NA	NA	NA	0,00351445	NA	NA	NA	NA
ILMN_1741632	RAB3IL1	0,002051329	NA	0,00205133	NA	0,00352303	NA	NA	NA	NA
ILMN_1692517	LOC653381	NA	NA	NA	NA	0,00352484	NA	NA	NA	NA
ILMN_1768480	VGLL4	NA	NA	0,00235131	0,0032362	0,00354342	NA	NA	NA	NA
ILMN_1694070	FAM114A1	NA	NA	0,00043785	NA	0,00355763	NA	NA	NA	NA
ILMN_1792679	ITGA5	NA	NA	0,00534997	NA	0,00357417	NA	NA	NA	NA
ILMN_1651776	FHOD1	0,000171649	NA	0,00017165	NA	0,00359988	NA	NA	NA	NA
ILMN_1734915	C1orf156	0,001176428	NA	0,00117643	NA	0,00363268	NA	NA	NA	NA
ILMN_2362858	PILRA	NA	NA	NA	NA	0,00365281	NA	NA	NA	NA
ILMN_1718852	PLCL1	NA	NA	NA	NA	0,00366998	NA	NA	NA	NA
ILMN_1753472	SUMF1	NA	NA	NA	NA	0,00368995	NA	NA	NA	NA
ILMN_1713985	MAF1	NA	NA	5,15E-05	NA	0,00369794	NA	NA	NA	NA
ILMN_2070355	SDHAF1	0,001258714	0,00125871	0,00125871	NA	0,00370323	NA	NA	NA	NA

ILMN_1803939	YIPF6	NA	NA	0,00645033	NA	0,00372167	NA	NA	NA	NA
ILMN_1783337	DECR2	NA	NA	4,94E-05	NA	0,00372419	NA	NA	NA	NA
ILMN_1679127	CCAR1	NA	NA	0,00740107	NA	0,00372939	NA	NA	NA	NA
ILMN_2393169	THOC5	NA	NA	0,00023557	NA	0,00373628	NA	NA	NA	NA
ILMN_1808157	RUNDC3B	NA	NA	NA	NA	0,00374467	NA	NA	NA	NA
ILMN_1660544	ARRDC4	NA	NA	0,00067005	NA	0,00376232	NA	NA	NA	NA
ILMN_1730294	INO80C	NA	NA	0,00534859	NA	0,00376317	NA	NA	NA	NA
ILMN_2059294	RTCD1	NA	NA	NA	NA	0,00376389	NA	NA	NA	NA
ILMN_1811823	MED25	NA	NA	0,00071004	NA	0,00376459	NA	NA	NA	NA
ILMN_1764709	MAFB	NA	NA	NA	NA	0,0037724	NA	NA	NA	NA
ILMN_2188722	GLS	NA	NA	0,00538573	NA	0,00379548	NA	NA	NA	NA
ILMN_1807945	ANP32A	NA	NA	0,00181569	NA	0,0038001	NA	NA	NA	NA
ILMN_1665797	CSE1L	0,002897552	NA	0,00289755	NA	0,00383302	NA	NA	NA	NA
ILMN_1798957	C12orf47	NA	NA	0,00011458	NA	0,00383867	NA	NA	NA	NA
ILMN_2342066	METRNL	NA	NA	0,00784574	NA	0,00386995	NA	NA	NA	NA
ILMN_1737663	C7orf13	0,004583161	NA	0,00458316	NA	0,00387378	NA	NA	NA	NA
ILMN_1807448	FAM8A1	NA	NA	NA	NA	0,0038808	NA	NA	NA	NA
ILMN_2415439	NAE1	NA	NA	NA	NA	0,00388954	NA	NA	NA	NA
ILMN_2348788	CD44	NA	NA	NA	NA	0,00391513	NA	NA	NA	NA
ILMN_3251550	PHLDA1	NA	NA	0,00324783	0,00705393	0,00391813	NA	NA	NA	NA
ILMN_1740505	SGMS1	NA	NA	0,00433695	NA	0,00392076	NA	NA	NA	NA
ILMN_2096654	COMMD4	NA	NA	NA	NA	0,0039399	NA	NA	NA	NA
ILMN_2079803	LSM14A	NA	NA	0,00330021	NA	0,00396479	NA	NA	NA	NA
ILMN_2373556	SPAST	NA	NA	0,00046195	NA	0,00397761	NA	NA	NA	NA
ILMN_1683179	RRAD	0,002438176	0,00243818	0,00243818	NA	0,00398084	NA	NA	NA	NA
ILMN_1810486	RAB34	NA	NA	NA	NA	0,00398114	NA	NA	NA	NA
ILMN_1726421	METTL9	NA	0,00078095	0,00078095	NA	0,00404466	NA	NA	NA	NA
ILMN_1776352	MUTED	0,000211984	NA	0,00021198	NA	0,0040536	NA	NA	NA	NA
ILMN_1663493	C7orf28B	NA	NA	NA	NA	0,0040548	NA	NA	NA	NA
ILMN_1692742	DENND3	NA	NA	NA	NA	0,00405852	NA	NA	NA	NA
ILMN_1741564	DCTN4	NA	NA	0,0077411	NA	0,00408715	NA	NA	NA	NA
ILMN_1739297	GALNT4	0,001555162	NA	0,00155516	NA	0,00410691	NA	NA	NA	NA
ILMN_2392261	FABP6	NA	NA	NA	NA	0,00412542	NA	NA	NA	NA
ILMN_1653438	PHF14	NA	NA	0,00799374	NA	0,00412597	NA	NA	NA	NA
ILMN_1808811	SBNO2	NA	NA	0,00168072	NA	0,00414923	NA	NA	NA	NA
ILMN_1715508	NNMT	4,72E-05	NA	4,72E-05	NA	0,00417771	NA	NA	NA	NA
ILMN_1785071	SEPP1	NA	NA	0,00538215	0,00637978	0,00418524	NA	NA	NA	NA
ILMN_2335718	HNRNPAB	0,001281349	NA	0,00128135	NA	0,0042104	NA	NA	NA	NA
ILMN_2330341	TCEAL4	NA	NA	0,00027374	0,00071391	0,00421822	NA	NA	NA	NA
ILMN_1746720	TTC39C	2,33E-05	NA	2,33E-05	NA	0,00422619	NA	NA	NA	NA
ILMN_2045419	BNIP3L	NA	NA	0,00418199	NA	0,00423627	NA	NA	NA	NA
ILMN_1720710	HSPB3	NA	NA	0,00258777	NA	0,00424642	NA	NA	NA	NA
ILMN_2402805	TRPC4AP	0,002335649	NA	0,00233565	NA	0,00430506	NA	NA	NA	NA
ILMN_1815479	NOP10	NA	NA	0,00065421	NA	0,00433328	NA	NA	NA	NA
ILMN_3209631	LOC728060	NA	NA	NA	NA	0,00434758	NA	NA	NA	NA
ILMN_1719286	CTSA	NA	0,00741374	0,00741374	NA	0,00436495	NA	NA	NA	NA
ILMN_1727041	EWSR1	NA	NA	NA	NA	0,00436911	NA	NA	NA	NA
ILMN_2412192	CFH	NA	NA	NA	NA	0,00439845	NA	NA	NA	NA
ILMN_2319424	GYG2	0,000779255	NA	0,00077926	NA	0,0044171	NA	NA	NA	NA
ILMN_1718934	LOC728499	NA	NA	NA	NA	0,00442701	NA	NA	NA	NA
ILMN_1803094	PDGFD	NA	NA	NA	NA	0,0044627	NA	NA	NA	NA
ILMN_2082209	C20orf100	NA	NA	0,00378993	NA	0,00447779	NA	NA	NA	NA
ILMN_1660282	POPDC2	NA	0,00785322	0,00785322	NA	0,00453155	NA	NA	NA	NA
ILMN_1882590		NA	NA	0,00492357	NA	0,00453917	NA	NA	NA	NA
ILMN_1709101	AKAP13	1,53E-05	NA	1,53E-05	0,00737805	0,00460959	NA	NA	NA	NA
ILMN_1680347	ZNF317	NA	NA	0,00341993	NA	0,00461467	NA	NA	NA	NA
ILMN_1732489	SLC10A7	0,000862474	NA	0,00086247	0,00632239	0,00462069	NA	NA	NA	NA
ILMN_2227573	GSTO1	NA	NA	NA	NA	0,00463223	NA	NA	NA	NA
ILMN_1780141	TMEM66	0,001131657	NA	0,00113166	NA	0,00464051	NA	NA	NA	NA
ILMN_2396956	AKAP13	NA	NA	0,00019334	0,00362991	0,00464734	NA	NA	NA	NA
ILMN_3191695	LOC100128266	NA	NA	0,00032108	NA	0,00471053	NA	NA	NA	NA
ILMN_1785324	MTHFD1	0,000417228	0,00041723	0,00041723	NA	0,00473723	NA	NA	NA	NA
ILMN_1809433	XBP1	NA	NA	0,00405145	NA	0,00475362	NA	NA	NA	NA
ILMN_1661306	LOC643433	NA	NA	0,00299613	0,00259507	0,00477446	NA	NA	NA	NA
ILMN_2053527	PARP9	NA	NA	0,00252956	NA	0,00477457	NA	NA	NA	NA
ILMN_1784206	LOC653308	NA	NA	0,00181915	NA	0,00479267	NA	NA	NA	NA
ILMN_1713496	ST3GAL5	NA	NA	NA	NA	0,0048009	NA	NA	NA	NA
ILMN_1724708	RAB33A	NA	NA	0,00279938	NA	0,00480559	NA	NA	NA	NA
ILMN_2384513	C2CD2	NA	NA	0,0030767	0,00556954	0,00482895	NA	NA	NA	NA
ILMN_1659415	MAP2K1IP1	2,97E-05	NA	2,97E-05	0,004493	0,00482947	NA	NA	NA	NA
ILMN_1705397	PDK2	NA	NA	NA	NA	0,00484411	NA	NA	NA	NA
ILMN_1713301	DGCR2	NA	NA	0,00221288	NA	0,00488441	NA	NA	NA	NA
ILMN_1742044	GNAI1	NA	NA	NA	NA	0,00488459	NA	NA	NA	NA
ILMN_3241118	LOC641727	NA	NA	0,00239465	NA	0,00488516	NA	NA	NA	NA

ILMN_1658717	DEPDC5	9,89E-05	9,89E-05	9,89E-05	NA	0,00492208	NA	NA	NA	NA
ILMN_2387090	CGGBP1	NA	NA	NA	NA	0,00494603	NA	NA	NA	NA
ILMN_2389810	ATP11C	NA	NA	NA	NA	0,00495572	NA	NA	NA	NA
ILMN_3251383	CCDC74B	NA	NA	NA	NA	0,00496458	NA	NA	NA	NA
ILMN_1652333	FN3KRP	NA	NA	0,00166283	NA	0,00496525	NA	NA	NA	NA
ILMN_1778917	CDK7	NA	NA	9,51E-05	NA	0,00496547	NA	NA	NA	NA
ILMN_1741594	CLCN6	NA	NA	NA	NA	0,00497063	NA	NA	NA	NA
ILMN_1753370	ABTB2	NA	NA	NA	NA	0,00499777	NA	NA	NA	NA
ILMN_2059535	PPM1F	0,000971472	NA	0,00097147	NA	0,00501646	NA	NA	NA	NA
ILMN_2222234	PRDX4	NA	NA	0,00235144	NA	0,00502858	NA	NA	NA	NA
ILMN_1672661	SP110	NA	NA	0,00083381	0,00928328	0,00507709	NA	NA	NA	NA
ILMN_2230592	MRPL3	NA	NA	NA	NA	0,0050847	NA	NA	NA	NA
ILMN_1737965	ELOVL4	NA	NA	NA	NA	0,00512753	NA	NA	NA	NA
ILMN_1795454	CPSF6	NA	NA	NA	NA	0,00514381	NA	NA	NA	NA
ILMN_3256868	LOC100129585	NA	NA	NA	NA	0,00514438	NA	NA	NA	NA
ILMN_1887174	KIAA0146	NA	NA	0,00079187	0,00952413	0,00515105	NA	NA	NA	NA
ILMN_1786707	C19orf63	NA	NA	NA	NA	0,00518381	NA	NA	NA	NA
ILMN_1808779	CSTF3	NA	NA	NA	NA	0,00519865	NA	NA	NA	NA
ILMN_1761155	PABPC1	NA	0,00047599	0,00047599	NA	0,00524519	NA	NA	NA	NA
ILMN_1764723	SH3PXD2B	NA	NA	0,00056672	NA	0,00524896	NA	NA	NA	NA
ILMN_1805826	BIVM	NA	NA	0,00102664	0,0078276	0,00526707	NA	NA	NA	NA
ILMN_2231020	TMEM185B	NA	NA	NA	NA	0,00528456	NA	NA	NA	NA
ILMN_3201221	LOC341315	NA	NA	0,00236365	NA	0,00531097	NA	NA	NA	NA
ILMN_1683127	ZNF281	NA	NA	NA	NA	0,00531711	NA	NA	NA	NA
ILMN_1660451	STARD13	NA	NA	NA	NA	0,00534951	NA	NA	NA	NA
ILMN_3198367	LOC646347	NA	NA	NA	NA	0,0053693	NA	NA	NA	NA
ILMN_2410523	DDR2	NA	NA	0,00139863	NA	0,0053844	NA	NA	NA	NA
ILMN_1783709	RRAGA	NA	NA	NA	NA	0,00543013	NA	NA	NA	NA
ILMN_3282174	LOC646688	NA	NA	0,00463938	NA	0,00545116	NA	NA	NA	NA
ILMN_1712112	RCAN1	NA	NA	0,0002072	NA	0,00545909	NA	NA	NA	NA
ILMN_1738075	CMIP	NA	NA	NA	NA	0,00545982	NA	NA	NA	NA
ILMN_2080637	ZBTB44	NA	NA	NA	NA	0,00546196	NA	NA	NA	NA
ILMN_1705468	PIK3CA	NA	NA	0,0037376	NA	0,00546494	NA	NA	NA	NA
ILMN_1791949	PGBD1	NA	NA	NA	NA	0,00548795	NA	NA	NA	NA
ILMN_2150019	SUCLA2	6,74E-05	6,74E-05	6,74E-05	NA	0,0055085	NA	NA	NA	NA
ILMN_3239168	LOC645332	NA	NA	NA	NA	0,00562026	NA	NA	NA	NA
ILMN_1705570	H2AFY2	NA	NA	NA	NA	0,00562311	NA	NA	NA	NA
ILMN_1674706	MTHFD2	NA	NA	0,00621167	NA	0,00562651	NA	NA	NA	NA
ILMN_1815924	NUP107	NA	NA	NA	NA	0,00564533	NA	NA	NA	NA
ILMN_1675501	SENP5	0,003332195	NA	0,0033322	0,00729368	0,00566731	NA	NA	NA	NA
ILMN_1670901	COX10	0,001898695	0,0018987	0,0018987	NA	0,00568654	NA	NA	NA	NA
ILMN_1761363	VAMP4	NA	0,00142291	0,00142291	NA	0,00569755	NA	NA	NA	NA
ILMN_1654916	KIF7	NA	NA	0,0087924	NA	0,00572254	NA	NA	NA	NA
ILMN_1744023	MGC18216	NA	NA	NA	NA	0,00575414	NA	NA	NA	NA
ILMN_1670079	OMA1	NA	NA	0,00375124	NA	0,00576092	NA	NA	NA	NA
ILMN_2392189	CTDSPL	NA	NA	NA	NA	0,0057713	NA	NA	NA	NA
ILMN_1712082	GCNT3	0,000816747	NA	0,00081675	0,0094451	0,00579548	NA	NA	NA	NA
ILMN_1660193	ZNF529	NA	NA	NA	NA	0,00580917	NA	NA	NA	NA
ILMN_2396272	PDCD4	NA	NA	NA	NA	0,00582923	NA	NA	NA	NA
ILMN_1746408	MIDN	NA	NA	NA	NA	0,00583076	NA	NA	NA	NA
ILMN_2136133	PABPC1	NA	NA	0,0005067	NA	0,00585263	NA	NA	NA	NA
ILMN_2127605	LRP3	NA	NA	0,00463782	0,00411264	0,00585315	NA	NA	NA	NA
ILMN_1788955	PDLIM1	7,82E-05	NA	7,82E-05	NA	0,00586866	NA	NA	NA	NA
ILMN_1815124	LOC645317	NA	0,00752063	0,00752063	NA	0,00588987	NA	NA	NA	NA
ILMN_1770911	NRSN2	0,001779315	NA	0,00177932	NA	0,00589774	NA	NA	NA	NA
ILMN_1768004	PDCD4	NA	NA	NA	NA	0,00591994	NA	NA	NA	NA
ILMN_1673548	HSPC159	NA	NA	NA	NA	0,0059406	NA	NA	NA	NA
ILMN_1689665	NAE1	NA	NA	0,00441024	NA	0,00595313	NA	NA	NA	NA
ILMN_1784523	ATP6V1G1	NA	NA	0,00026216	NA	0,0059741	NA	NA	NA	NA
ILMN_1656285	METTL7A	NA	NA	NA	NA	0,00599378	NA	NA	NA	NA
ILMN_1745217	FLJ10081	NA	NA	NA	NA	0,00601866	NA	NA	NA	NA
ILMN_2075643	ANKRD29	NA	NA	0,00559912	NA	0,00605002	NA	NA	NA	NA
ILMN_1807600	NPLOC4	0,001717076	NA	0,00171708	NA	0,00610059	NA	NA	NA	NA
ILMN_2389501	HSD11B1	NA	NA	0,00255912	0,00423052	0,00610698	NA	NA	NA	NA
ILMN_1778691	TIA1	NA	NA	NA	NA	0,00611805	NA	NA	NA	NA
ILMN_1751016	LONRF2	NA	NA	NA	NA	0,00613568	NA	NA	NA	NA
ILMN_1665538	SKP2	NA	NA	0,00493855	NA	0,00614903	NA	NA	NA	NA
ILMN_3307659	SFT2D2	NA	NA	NA	NA	0,00615928	NA	NA	NA	NA
ILMN_2349393	MDK	NA	NA	NA	NA	0,00616078	NA	NA	NA	NA
ILMN_1746171	H2AFY	NA	NA	NA	NA	0,00616486	NA	NA	NA	NA
ILMN_2358457	ATF4	NA	NA	0,00388534	NA	0,00619316	NA	NA	NA	NA
ILMN_1796801	ABCA8	NA	NA	NA	NA	0,00621573	NA	NA	NA	NA
ILMN_2155516	QTRTD1	NA	NA	0,00416925	0,00717535	0,00622912	NA	NA	NA	NA
ILMN_1803060	MAML1	NA	NA	NA	NA	0,00623961	NA	NA	NA	NA

ILMN_1677567	EPYC	NA	NA	NA	NA	0,00624078	NA	NA	NA	NA
ILMN_3276859	LOC401127	NA	NA	NA	NA	0,00625239	NA	NA	NA	NA
ILMN_1677239	CCDC14	NA	NA	NA	NA	0,00626227	NA	NA	NA	NA
ILMN_1777286	LOC388344	NA	NA	NA	NA	0,00626749	NA	NA	NA	NA
ILMN_2041236	GARNL4	NA	NA	NA	NA	0,00628287	NA	NA	NA	NA
ILMN_1796179	HIST1H2BK	NA	NA	NA	NA	0,00631311	NA	NA	NA	NA
ILMN_2364357	RPS6KB2	NA	NA	NA	NA	0,00641424	NA	NA	NA	NA
ILMN_1838313		NA	NA	0,00633509	NA	0,00645584	NA	NA	NA	NA
ILMN_2391355	STAMBP	NA	NA	0,00694908	NA	0,00649733	NA	NA	NA	NA
ILMN_1667561	IFRD1	NA	NA	NA	NA	0,00649919	NA	NA	NA	NA
ILMN_1774083	TRIAP1	0,001264378	0,00126438	0,00126438	NA	0,0065214	NA	NA	NA	NA
ILMN_1740572	TCN2	NA	NA	NA	NA	0,006522	NA	NA	NA	NA
ILMN_1762426	DHFRL1	NA	NA	NA	NA	0,00655421	NA	NA	NA	NA
ILMN_1669972	DKFZp686O24166	NA	NA	NA	NA	0,00656509	NA	NA	NA	NA
ILMN_2231021	TMEM185B	NA	NA	NA	NA	0,00659454	NA	NA	NA	NA
ILMN_1738523	MYD88	9,26E-05	NA	9,26E-05	NA	0,00662127	NA	NA	NA	NA
ILMN_1796075	VPS41	NA	NA	NA	NA	0,00665521	NA	NA	NA	NA
ILMN_3299393	PNMAL1	NA	NA	NA	NA	0,00668483	NA	NA	NA	NA
ILMN_1666727	ZNF586	NA	NA	0,00068567	NA	0,00670763	NA	NA	NA	NA
ILMN_1731237	CLSTN2	NA	NA	0,00033736	NA	0,0067288	NA	NA	NA	NA
ILMN_1683096	ASB1	NA	NA	NA	NA	0,00674072	NA	NA	NA	NA
ILMN_3269405	HNRNPM	NA	NA	0,00759804	NA	0,00676573	NA	NA	NA	NA
ILMN_1771732	AS3MT	NA	NA	NA	NA	0,00681211	NA	NA	NA	NA
ILMN_3280842	LOC149501	NA	NA	0,00218803	NA	0,00684285	NA	NA	NA	NA
ILMN_1814966	PIAS3	0,003641642	NA	0,00364164	NA	0,00684502	NA	NA	NA	NA
ILMN_1773337	DKK1	NA	NA	NA	NA	0,00686839	NA	NA	NA	NA
ILMN_1802894	VKORC1L1	NA	NA	NA	NA	0,00688808	NA	NA	NA	NA
ILMN_1689156	MMAB	NA	NA	NA	NA	0,00690363	NA	NA	NA	NA
ILMN_1678692	MPRIP	NA	NA	NA	NA	0,006931	NA	NA	NA	NA
ILMN_1674032	ABCD3	NA	NA	NA	NA	0,0069505	NA	NA	NA	NA
ILMN_1684461	CADPS2	NA	NA	NA	NA	0,00697838	NA	NA	NA	NA
ILMN_1692168	UBE2Z	NA	NA	0,00584338	NA	0,00700923	NA	NA	NA	NA
ILMN_2235283	MAPK1	NA	NA	0,00997274	NA	0,00701786	NA	NA	NA	NA
ILMN_1787808	CEP63	NA	NA	NA	NA	0,00703273	NA	NA	NA	NA
ILMN_1724489	RFC4	NA	NA	NA	NA	0,00703352	NA	NA	NA	NA
ILMN_1652753	PAAF1	NA	NA	NA	NA	0,00707295	NA	NA	NA	NA
ILMN_1694305	SMS	NA	NA	NA	NA	0,00710785	NA	NA	NA	NA
ILMN_2057220	HRSP12	NA	NA	NA	NA	0,00711027	NA	NA	NA	NA
ILMN_1796710	PHC1	NA	NA	NA	NA	0,00713672	NA	NA	NA	NA
ILMN_2407824	ATP1B1	NA	NA	NA	NA	0,00717115	NA	NA	NA	NA
ILMN_1703949	KPNB1	0,000125732	NA	0,00012573	NA	0,0071783	NA	NA	NA	NA
ILMN_1806010	CUL9	NA	NA	NA	NA	0,00718998	NA	NA	NA	NA
ILMN_1666632	VPS52	0,000201225	NA	0,00020123	NA	0,0072018	NA	NA	NA	NA
ILMN_1815445	IDS	NA	NA	6,73E-05	NA	0,0072127	NA	NA	NA	NA
ILMN_1737604	FLJ10986	NA	NA	0,00982618	NA	0,00721773	NA	NA	NA	NA
ILMN_3276861	LOC441013	NA	NA	NA	NA	0,00723554	NA	NA	NA	NA
ILMN_1763941	LRRC49	NA	NA	NA	NA	0,00723773	NA	NA	NA	NA
ILMN_1803997	SDCCAG3	NA	NA	0,00407298	NA	0,00723993	NA	NA	NA	NA
ILMN_2376403	TSC22D3	NA	NA	0,0018757	0,00342935	0,00725149	NA	NA	NA	NA
ILMN_2403247	CMTM7	NA	NA	NA	NA	0,00725367	NA	NA	NA	NA
ILMN_1699208	NAP1L1	NA	NA	NA	NA	0,00727694	NA	NA	NA	NA
ILMN_1683415	CAMK2D	NA	NA	0,00258361	NA	0,00734205	NA	NA	NA	NA
ILMN_2330861	SMC4	NA	NA	NA	NA	0,00734295	NA	NA	NA	NA
ILMN_1705301	TEAD4	NA	NA	0,00012792	NA	0,00734489	NA	NA	NA	NA
ILMN_1662896	BRWD2	NA	NA	0,00283069	NA	0,00734489	NA	NA	NA	NA
ILMN_2173004	RAB8B	NA	NA	0,00801983	NA	0,00734704	NA	NA	NA	NA
ILMN_1675947	MT3	NA	NA	NA	NA	0,00735926	NA	NA	NA	NA
ILMN_1722059	SAFB	0,001285985	NA	0,00128599	NA	0,00736159	NA	NA	NA	NA
ILMN_1811238	ALPK2	NA	NA	0,00014972	NA	0,0074479	NA	NA	NA	NA
ILMN_1743319	PAFAH1B2	NA	NA	NA	NA	0,0074683	NA	NA	NA	NA
ILMN_1708203	OTUD4	NA	NA	NA	NA	0,00752136	NA	NA	NA	NA
ILMN_1702198	LOC643790	NA	NA	NA	NA	0,0075284	NA	NA	NA	NA
ILMN_2189458	SHQ1	NA	NA	0,00936528	NA	0,00754525	NA	NA	NA	NA
ILMN_1687848	C7	NA	NA	0,00786117	NA	0,00756517	NA	NA	NA	NA
ILMN_1735453	FAM98A	NA	NA	NA	NA	0,00758839	NA	NA	NA	NA
ILMN_2331266	NUMB	NA	NA	0,00818185	NA	0,00761084	NA	NA	NA	NA
ILMN_1759252	ADD1	NA	NA	9,62E-05	0,00438246	0,00761832	NA	NA	NA	NA
ILMN_1700044	SAP130	NA	NA	0,00093128	NA	0,00764743	NA	NA	NA	NA
ILMN_2217630	CDKL3	0,000453409	NA	0,00045341	NA	0,00764951	NA	NA	NA	NA
ILMN_3248773	C7orf40	NA	NA	0,0042435	NA	0,00766819	NA	NA	NA	NA
ILMN_1815057	PDGFRB	NA	NA	0,00513975	NA	0,0076769	NA	NA	NA	NA
ILMN_1767475	CERK	NA	NA	NA	NA	0,00768751	NA	NA	NA	NA
ILMN_1783285	CTPS	NA	NA	NA	NA	0,00772193	NA	NA	NA	NA
ILMN_2179778	PHLDB2	NA	NA	0,00894122	NA	0,00772579	NA	NA	NA	NA

ILMN_1724811	PARN	NA	NA	0,00405403	NA	0,00773283	NA	NA	NA	NA
ILMN_3304042	LOC729051	NA	NA	NA	NA	0,00773927	NA	NA	NA	NA
ILMN_1757406	HIST1H1C	NA	NA	NA	NA	0,00774665	NA	NA	NA	NA
ILMN_2139100	SHISA5	0,000482452	NA	0,00048245	NA	0,00777912	NA	NA	NA	NA
ILMN_2336647	NNT	NA	NA	0,00167213	NA	0,00778151	NA	NA	NA	NA
ILMN_1707503	C1orf144	NA	NA	NA	NA	0,00779282	NA	NA	NA	NA
ILMN_1673369	SEPHS1	NA	NA	NA	NA	0,00780225	NA	NA	NA	NA
ILMN_1720083	EHD4	NA	NA	0,00387985	NA	0,0078281	NA	NA	NA	NA
ILMN_1804384	ASMTL	NA	NA	0,00831158	NA	0,00782906	NA	NA	NA	NA
ILMN_3242205	GMPS	NA	NA	NA	NA	0,00784928	NA	NA	NA	NA
ILMN_1754489	FBXL20	NA	NA	0,00613233	NA	0,00788593	NA	NA	NA	NA
ILMN_1699631	GATS	NA	NA	0,00860446	0,00562925	0,00796756	NA	NA	NA	NA
ILMN_1726666	GPX3	NA	NA	NA	NA	0,00797629	NA	NA	NA	NA
ILMN_1777449	IFT74	NA	NA	NA	NA	0,00802879	NA	NA	NA	NA
ILMN_1765060	FBXO34	NA	NA	0,00264744	0,00917825	0,00803131	NA	NA	NA	NA
ILMN_2184184	ANXA1	NA	NA	NA	NA	0,00807513	NA	NA	NA	NA
ILMN_2146648	PRB3	NA	NA	0,00309701	NA	0,00808385	NA	NA	NA	NA
ILMN_1809708	KCTD21	NA	NA	0,00585877	0,00762654	0,00808693	NA	NA	NA	NA
ILMN_1659285	PSMG1	NA	NA	NA	NA	0,00810952	NA	NA	NA	NA
ILMN_2183692	PHYHD1	NA	NA	0,00200125	NA	0,00811313	NA	NA	NA	NA
ILMN_1743646	VASP	NA	0,0215583	NA	NA	0,00813576	NA	NA	NA	NA
ILMN_1760575	PTP4A1	NA	NA	NA	NA	0,00815734	NA	NA	NA	NA
ILMN_1700507	CLASP1	0,002617022	NA	0,00261702	NA	0,00820076	NA	NA	NA	NA
ILMN_1757415	C1orf163	NA	NA	NA	NA	0,0082045	NA	NA	NA	NA
ILMN_3217172	LOC286444	NA	NA	0,00514081	NA	0,008271	NA	NA	NA	NA
ILMN_1794885	FRMD5	NA	NA	NA	NA	0,00827677	NA	NA	NA	NA
ILMN_3238369	JHDM1D	NA	NA	NA	NA	0,0082841	NA	NA	NA	NA
ILMN_2387865	KCNIP4	NA	NA	0,0023184	0,00992187	0,0082998	NA	NA	NA	NA
ILMN_1677814	ABCC3	NA	0,05882345	NA	NA	0,0083046	NA	NA	NA	NA
ILMN_1706677	MAPK1	NA	NA	NA	NA	0,00831348	NA	NA	NA	NA
ILMN_2124951	RBMX	NA	NA	0,00634275	NA	0,00832921	NA	NA	NA	NA
ILMN_1667068	ZC3HAV1	NA	NA	0,0083329	NA	0,00834352	NA	NA	NA	NA
ILMN_1672759	CCDC109A	NA	NA	NA	NA	0,00841361	NA	NA	NA	NA
ILMN_1806634	NNT	NA	NA	0,00499203	NA	0,00844838	NA	NA	NA	NA
ILMN_3215759	LOC441484	NA	NA	NA	NA	0,00848039	NA	NA	NA	NA
ILMN_1801441	RFTN2	NA	NA	NA	NA	0,00848856	NA	NA	NA	NA
ILMN_1678757	BCYRN1	NA	NA	NA	NA	0,0085534	NA	NA	NA	NA
ILMN_1767837	GOLT1B	NA	NA	0,00740733	NA	0,0085653	NA	NA	NA	NA
ILMN_2098743	THEM2	NA	NA	NA	NA	0,00857892	NA	NA	NA	NA
ILMN_1698144	CFH	NA	NA	NA	NA	0,0085999	NA	NA	NA	NA
ILMN_1746368	SELT	NA	NA	NA	NA	0,00863705	NA	NA	NA	NA
ILMN_1682781	TEAD2	NA	NA	NA	NA	0,00864911	NA	NA	NA	NA
ILMN_1785891	PRKD1	NA	NA	NA	NA	0,00865141	NA	NA	NA	NA
ILMN_1781097	UBXN4	NA	NA	0,00075377	NA	0,00865638	NA	NA	NA	NA
ILMN_1740923	C20orf54	NA	NA	NA	NA	0,00866301	NA	NA	NA	NA
ILMN_1801610	METRNL	NA	NA	0,00374609	NA	0,0086702	NA	NA	NA	NA
ILMN_1719906	HADH	NA	NA	NA	NA	0,00871054	NA	NA	NA	NA
ILMN_3234547	LOC100133803	NA	NA	NA	NA	0,00871614	NA	NA	NA	NA
ILMN_1717781	VPS11	NA	NA	0,00141873	NA	0,00872305	NA	NA	NA	NA
ILMN_2405521	MTHFD2	NA	NA	0,00361689	NA	0,00874796	NA	NA	NA	NA
ILMN_1758672	FAM107B	0,027003003	NA	NA	NA	0,00875221	NA	NA	NA	NA
ILMN_1726981	VEGFB	NA	NA	NA	NA	0,00877424	NA	NA	NA	NA
ILMN_1679450	LOC730746	NA	NA	NA	NA	0,00880813	NA	NA	NA	NA
ILMN_2162234	NEK1	NA	NA	NA	NA	0,00881475	NA	NA	NA	NA
ILMN_1660533	RPN1	NA	NA	0,00588781	NA	0,00882219	NA	NA	NA	NA
ILMN_1770245	EPB41L5	NA	NA	NA	NA	0,00882307	NA	NA	NA	NA
ILMN_1703006	MTCH1	NA	NA	NA	NA	0,00882563	NA	NA	NA	NA
ILMN_3247325	LOC100190939	NA	NA	NA	NA	0,00883039	NA	NA	NA	NA
ILMN_2094377	TRPC3	NA	NA	NA	NA	0,00883373	NA	NA	NA	NA
ILMN_2191759	LOC440396	NA	NA	0,00098952	NA	0,00885355	NA	NA	NA	NA
ILMN_1685480	TARS	NA	NA	0,00867449	NA	0,00885408	NA	NA	NA	NA
ILMN_1761804	ALDH9A1	NA	NA	NA	NA	0,00886024	NA	NA	NA	NA
ILMN_2398847	ARHGAP17	NA	NA	NA	NA	0,00893366	NA	NA	NA	NA
ILMN_1666819	PHLDB1	NA	NA	NA	NA	0,00893911	NA	NA	NA	NA
ILMN_1661424	THAP6	NA	NA	0,00164057	NA	0,00894398	NA	NA	NA	NA
ILMN_1780058	DEGS1	NA	NA	0,00660824	NA	0,00899966	NA	NA	NA	NA
ILMN_1751561	CAMK1D	NA	NA	NA	NA	0,00908905	NA	NA	NA	NA
ILMN_3251251	GNL3L	NA	NA	0,00076029	NA	0,00909381	NA	NA	NA	NA
ILMN_1658928	GNB2	NA	NA	0,00041138	NA	0,009115	NA	NA	NA	NA
ILMN_2200503	NIT2	NA	NA	NA	NA	0,00915342	NA	NA	NA	NA
ILMN_2048700	ATAD2	NA	NA	NA	NA	0,00917078	NA	NA	NA	NA
ILMN_1709044	TGIF2	NA	NA	NA	NA	0,00918034	NA	NA	NA	NA
ILMN_1813091	ARL1	NA	NA	0,00663751	NA	0,00918643	NA	NA	NA	NA
ILMN_1771261	SYNC1	0,003792698	NA	0,0037927	NA	0,00923503	NA	NA	NA	NA

ILMN_1721241	PSCD2	0,000393529	NA	0,00039353	NA	0,00927783	NA	NA	NA	NA
ILMN_1751276	BDNF	NA	NA	NA	NA	0,00931417	NA	NA	NA	NA
ILMN_1733155	GIT1	NA	NA	0,00699191	NA	0,00935497	NA	NA	NA	NA
ILMN_1751403	NUDT15	0,000392267	NA	0,00039227	NA	0,0093802	NA	NA	NA	NA
ILMN_2263718	SPAG9	NA	NA	0,00741403	NA	0,00945589	NA	NA	NA	NA
ILMN_1803429	CD44	NA	NA	NA	NA	0,00954666	NA	NA	NA	NA
ILMN_2172202	NUDT15	0,002543691	0,00254369	0,00254369	NA	0,00957309	NA	NA	NA	NA
ILMN_1765076	APPL2	NA	NA	NA	NA	0,00957718	NA	NA	NA	NA
ILMN_1656316	ZMYM3	NA	NA	NA	NA	0,00957978	NA	NA	NA	NA
ILMN_2340908	SCML1	NA	NA	NA	NA	0,00958046	NA	NA	NA	NA
ILMN_2297626	PEG10	NA	NA	NA	NA	0,00959022	NA	NA	NA	NA
ILMN_1716766	CEBPG	NA	NA	0,00547999	NA	0,00961426	NA	NA	NA	NA
ILMN_1672122	P4HTM	NA	0,04086365	NA	NA	0,00962087	NA	NA	NA	NA
ILMN_1786139	VKORC1	NA	NA	0,00184422	0,00545275	0,00964907	NA	NA	NA	NA
ILMN_1740171	DUSP11	0,000140554	NA	0,00014055	NA	0,00967686	NA	NA	NA	NA
ILMN_1672504	PDXK	NA	NA	NA	NA	0,00968999	NA	NA	NA	NA
ILMN_1766165	SNCA	0,00382767	NA	0,00382767	NA	0,00970195	NA	NA	NA	NA
ILMN_1808196	GSTO1	NA	NA	NA	NA	0,00970754	NA	NA	NA	NA
ILMN_1723662	ABHD2	NA	NA	NA	NA	0,00971438	NA	NA	NA	NA
ILMN_1741133	NME1	NA	NA	0,00082913	0,0071375	0,00974861	NA	NA	NA	NA
ILMN_2401057	CANX	NA	NA	0,00336074	NA	0,00976718	NA	NA	NA	NA
ILMN_1787628	NOP56	NA	NA	NA	NA	0,00977286	NA	NA	NA	NA
ILMN_2143261	CXorf40B	0,00028125	NA	0,00028125	NA	0,00983954	NA	NA	NA	NA
ILMN_1698934	CMTM7	NA	NA	NA	NA	0,00986143	NA	NA	NA	NA
ILMN_1753782	ZNF266	0,02135706	NA	NA	NA	0,00989341	NA	NA	NA	NA
ILMN_1689446	EIF3G	0,003136336	NA	0,00313634	NA	0,00990536	NA	NA	NA	NA
ILMN_2089167	RHOD	NA	NA	NA	NA	0,00992364	NA	NA	NA	NA
ILMN_1755749	PGK1	NA	NA	NA	NA	0,00992847	NA	NA	NA	NA
ILMN_3208233	LOC100131735	NA	NA	NA	NA	0,00995264	NA	NA	NA	NA
ILMN_1766405	GOLM1	NA	NA	NA	NA	0,00996989	NA	NA	NA	NA
ILMN_1739821	EIF2S1	NA	NA	0,00082723	NA	0,00999786	NA	NA	NA	NA
ILMN_2220187	GFPT1	NA	NA	NA	2,83E-06	NA	NA	NA	NA	NA
ILMN_2189406	C15orf38	NA	NA	NA	2,07E-05	NA	NA	NA	NA	NA
ILMN_1692077	MXRA7	NA	NA	NA	2,98E-05	NA	NA	NA	NA	NA
ILMN_3188124	LOC100130511	NA	NA	NA	6,66E-05	NA	NA	NA	NA	NA
ILMN_1662232	DCTN2	NA	NA	NA	7,72E-05	NA	NA	NA	NA	NA
ILMN_2410783	GAA	NA	NA	0,00028578	8,68E-05	NA	NA	NA	NA	NA
ILMN_1788416	FAM108C1	0,00082932	NA	0,00082932	0,00011128	NA	NA	NA	NA	NA
ILMN_2067852	SLC30A1	NA	NA	NA	0,00017109	NA	NA	NA	NA	NA
ILMN_2228180	MSRA	NA	NA	NA	0,00017501	NA	NA	NA	NA	NA
ILMN_2408645	LOC653566	NA	NA	NA	0,00019362	NA	NA	NA	NA	NA
ILMN_2115633	CD320	NA	NA	NA	0,00019929	NA	NA	NA	NA	NA
ILMN_1664994	MINPP1	NA	NA	NA	0,00020475	NA	NA	NA	NA	NA
ILMN_2077905	PTGFRN	NA	NA	NA	0,00020922	NA	NA	NA	NA	NA
ILMN_1788059	PCGF5	NA	NA	NA	0,00023973	NA	NA	NA	NA	NA
ILMN_2220184	GFPT1	0,002040081	NA	0,00204008	0,00025637	NA	NA	NA	NA	NA
ILMN_1851376		NA	NA	NA	0,00029342	NA	NA	NA	NA	NA
ILMN_1653319	MC1R	NA	NA	NA	0,00031417	NA	NA	NA	NA	NA
ILMN_2141941	TOR1AIP1	NA	NA	0,00642638	0,0003211	NA	NA	NA	NA	NA
ILMN_1804117	FAM89B	NA	NA	NA	0,00033295	NA	NA	NA	NA	NA
ILMN_2322935	MAPKAPK5	NA	NA	NA	0,00034711	NA	NA	NA	NA	NA
ILMN_1704452	BCL9	NA	NA	NA	0,00046153	NA	NA	NA	NA	NA
ILMN_1768293	NUP155	NA	NA	NA	0,00047793	NA	NA	NA	NA	NA
ILMN_2411236	NRCAM	NA	NA	NA	0,0004809	NA	NA	NA	NA	NA
ILMN_3206343	LOC644914	NA	NA	NA	0,00054008	NA	NA	NA	NA	NA
ILMN_2175601	VDAC1	NA	NA	NA	0,00056836	NA	NA	NA	NA	NA
ILMN_1821280		NA	NA	NA	0,00057008	NA	NA	NA	NA	NA
ILMN_2126239	SMG5	NA	NA	NA	0,00057277	NA	NA	NA	NA	NA
ILMN_2072091	HNRNPUL2	NA	NA	NA	0,00069926	NA	NA	NA	NA	NA
ILMN_1651315	HMG20B	NA	NA	NA	0,00071525	NA	NA	NA	NA	NA
ILMN_1667883	THOC5	NA	NA	NA	0,00073434	NA	NA	NA	NA	NA
ILMN_3242120	RAP1BL	NA	NA	NA	0,00076878	NA	NA	NA	NA	NA
ILMN_1683959	MED13L	NA	NA	NA	0,0007751	NA	NA	NA	NA	NA
ILMN_1815023	PIM1	NA	NA	NA	0,00077665	NA	NA	NA	NA	NA
ILMN_2137066	ZNF7	NA	NA	0,00924099	0,00079776	NA	NA	NA	NA	NA
ILMN_1665243	FKBP14	NA	NA	NA	0,00083594	NA	NA	NA	NA	NA
ILMN_1802557	HEBP1	NA	NA	NA	0,00085066	NA	NA	NA	NA	NA
ILMN_2392352	CTPS2	NA	NA	NA	0,00085546	NA	NA	NA	NA	NA
ILMN_1651346	TICAM2	NA	NA	NA	0,00085733	NA	NA	NA	NA	NA
ILMN_1666545	GCNT1	NA	NA	0,00244922	0,00088241	NA	NA	NA	NA	NA
ILMN_2405400	GRK4	NA	NA	NA	0,00088998	NA	NA	NA	NA	NA
ILMN_2195482	CACNB3	NA	NA	NA	0,00089555	NA	NA	NA	NA	NA
ILMN_2101375	CCDC77	NA	NA	NA	0,00093715	NA	NA	NA	NA	NA
ILMN_1857017		NA	NA	0,00584612	0,00098091	NA	NA	NA	NA	NA

ILMN_1719998	C9orf45	NA	NA	NA	0,00098169	NA	NA	NA	NA	NA
ILMN_1724207	IVD	NA	NA	NA	0,0009905	NA	NA	NA	NA	NA
ILMN_1656386	SEC24D	NA	NA	NA	0,00115011	NA	NA	NA	NA	NA
ILMN_1756878	SLC39A9	NA	NA	NA	0,00119472	NA	NA	NA	NA	NA
ILMN_1780699	THAP11	NA	NA	NA	0,00124923	NA	NA	NA	NA	NA
ILMN_1717366	MDFIC	NA	NA	NA	0,00133517	NA	NA	NA	NA	NA
ILMN_3229552	MED7	NA	NA	0,00323357	0,00137475	NA	NA	NA	NA	NA
ILMN_1793267	ETHE1	NA	NA	NA	0,00141643	NA	NA	NA	NA	NA
ILMN_1690268	HNRPUL1	NA	NA	0,00210948	0,00142464	NA	NA	NA	NA	NA
ILMN_1763007	HIAT1	NA	NA	NA	0,00143125	NA	NA	NA	NA	NA
ILMN_1770787	DDAH2	NA	NA	NA	0,00147186	NA	NA	NA	NA	NA
ILMN_2415572	GALC	NA	NA	NA	0,00148001	NA	NA	NA	NA	NA
ILMN_2365686	ALG8	NA	NA	NA	0,00148209	NA	NA	NA	NA	NA
ILMN_1712390	CUTA	NA	NA	NA	0,00155282	NA	NA	NA	NA	NA
ILMN_1652371	KIAA1324L	NA	NA	NA	0,00159559	NA	NA	NA	NA	NA
ILMN_1699503	UBE2D2	NA	NA	NA	0,00166023	NA	NA	NA	NA	NA
ILMN_2389506	HSD11B1	NA	NA	0,00275428	0,00169134	NA	NA	NA	NA	NA
ILMN_1763433	TRIM9	NA	NA	NA	0,00173535	NA	NA	NA	NA	NA
ILMN_1813763	SLC20A2	NA	NA	NA	0,00178891	NA	NA	NA	NA	NA
ILMN_1776826	FLJ32810	NA	NA	NA	0,00180956	NA	NA	NA	NA	NA
ILMN_2362232	ZNF331	NA	NA	NA	0,00182813	NA	NA	NA	NA	NA
ILMN_1795949	CORO7	NA	NA	NA	0,00184578	NA	NA	NA	NA	NA
ILMN_1743747	RUSC1	NA	NA	NA	0,00185016	NA	NA	NA	NA	NA
ILMN_1677402	LOC387763	NA	NA	NA	0,00185957	NA	NA	NA	NA	NA
ILMN_1758633	CCDC130	NA	NA	NA	0,00190696	NA	NA	NA	NA	NA
ILMN_1756910	PLA2G15	NA	NA	0,00389311	0,00190784	NA	NA	NA	NA	NA
ILMN_1795876	GPS1	NA	NA	NA	0,00192159	NA	NA	NA	NA	NA
ILMN_1758311	NET1	NA	NA	NA	0,00192575	NA	NA	NA	NA	NA
ILMN_1757272	THAP1	NA	NA	0,00373492	0,00195364	NA	NA	NA	NA	NA
ILMN_1727740	SYNCRIP	NA	NA	NA	0,00198142	NA	NA	NA	NA	NA
ILMN_1653134	TMEM188	NA	NA	NA	0,00203165	NA	NA	NA	NA	NA
ILMN_1671486	HOMER2	NA	NA	NA	0,00209866	NA	NA	NA	NA	NA
ILMN_2077094	C11orf2	NA	NA	0,00210765	0,00211837	NA	NA	NA	NA	NA
ILMN_1760667	POLR3GL	NA	NA	NA	0,0021357	NA	NA	NA	NA	NA
ILMN_1765801	GAA	NA	NA	NA	0,00215523	NA	NA	NA	NA	NA
ILMN_1797522	DUSP3	NA	NA	NA	0,002162	NA	NA	NA	NA	NA
ILMN_3257145	LOC100129602	NA	NA	NA	0,00219762	NA	NA	NA	NA	NA
ILMN_1814971	TCF25	NA	NA	NA	0,00227952	NA	NA	NA	NA	NA
ILMN_1801101	ZBTB48	NA	NA	NA	0,00230266	NA	NA	NA	NA	NA
ILMN_3236866	ULK4	NA	NA	NA	0,00232456	NA	NA	NA	NA	NA
ILMN_2334765	ARMCX3	NA	NA	NA	0,00235941	NA	NA	NA	NA	NA
ILMN_1673892	GK5	NA	NA	NA	0,00236849	NA	NA	NA	NA	NA
ILMN_1693430	NME1-NME2	NA	NA	0,00731446	0,00237782	NA	NA	NA	NA	NA
ILMN_1746135	PHF23	NA	NA	NA	0,00242758	NA	NA	NA	NA	NA
ILMN_1755643	MGAT4A	NA	NA	NA	0,00243043	NA	NA	NA	NA	NA
ILMN_1670145	DFNA5	NA	NA	NA	0,00245157	NA	NA	NA	NA	NA
ILMN_1736533	RND2	NA	NA	NA	0,00246983	NA	NA	NA	NA	NA
ILMN_1711199	ZNF331	NA	NA	NA	0,00247165	NA	NA	NA	NA	NA
ILMN_2125010	SKAP2	NA	NA	0,00997191	0,00252365	NA	NA	NA	NA	NA
ILMN_1659843	DNAJC3	NA	NA	NA	0,00256849	NA	NA	NA	NA	NA
ILMN_1742382	RIMS3	NA	NA	NA	0,00259578	NA	NA	NA	NA	NA
ILMN_1717855	PFDN1	NA	NA	NA	0,0026219	NA	NA	NA	NA	NA
ILMN_1770822	RPS6KA3	NA	NA	NA	0,00263667	NA	NA	NA	NA	NA
ILMN_1741674	PPP1R9A	NA	NA	NA	0,00274338	NA	NA	NA	NA	NA
ILMN_3238982	PATE3	NA	NA	NA	0,00275209	NA	NA	NA	NA	NA
ILMN_1794187	FBXL3	NA	NA	NA	0,00282372	NA	NA	NA	NA	NA
ILMN_1721170	GREB1	NA	NA	NA	0,0028242	NA	NA	NA	NA	NA
ILMN_1812776	FBXO28	NA	NA	NA	0,00282658	NA	NA	NA	NA	NA
ILMN_1766195	ZNF558	NA	NA	NA	0,00286347	NA	NA	NA	NA	NA
ILMN_1768798	SPAG9	NA	NA	NA	0,00286923	NA	NA	NA	NA	NA
ILMN_3289650	LOC402112	NA	NA	NA	0,00294372	NA	NA	NA	NA	NA
ILMN_1664641	MED4	3,77E-06	NA	3,77E-06	0,00294816	NA	NA	NA	NA	NA
ILMN_2075757	LRIG3	NA	NA	NA	0,002967	NA	NA	NA	NA	NA
ILMN_3249645	HSPA13	NA	NA	0,00762872	0,00297836	NA	NA	NA	NA	NA
ILMN_3243142	KAT2B	NA	NA	NA	0,00300185	NA	NA	NA	NA	NA
ILMN_1810875	SYNGR1	NA	NA	NA	0,00302721	NA	NA	NA	NA	NA
ILMN_1801710	APBB1IP	NA	NA	NA	0,00310041	NA	NA	NA	NA	NA
ILMN_1797893	PFAAP5	NA	NA	NA	0,0031093	NA	NA	NA	NA	NA
ILMN_1685097	ASCC1	NA	NA	NA	0,00311861	NA	NA	NA	NA	NA
ILMN_1724293	KDELR2	NA	NA	0,00150512	0,00318888	NA	NA	NA	NA	NA
ILMN_2041161	DENND4A	NA	NA	NA	0,00320373	NA	NA	NA	NA	NA
ILMN_1691104	PGAM4	NA	NA	NA	0,0032039	NA	NA	NA	NA	NA
ILMN_1654915	LOC646786	NA	NA	NA	0,00321558	NA	NA	NA	NA	NA
ILMN_1756146	WDR45	NA	NA	0,00835031	0,00322124	NA	NA	NA	NA	NA

ILMN_1769091	PRCP	0,008168313	NA	0,00816831	0,0033275	NA	NA	NA	NA	NA
ILMN_1788988	THAP1	NA	NA	0,00124976	0,00333601	NA	NA	NA	NA	NA
ILMN_2047206	TMX3	NA	NA	NA	0,00334427	NA	NA	NA	NA	NA
ILMN_2210837	CDC26	NA	NA	NA	0,00335561	NA	NA	NA	NA	NA
ILMN_1776515	MPPE1	NA	NA	NA	0,00339936	NA	NA	NA	NA	NA
ILMN_1807211	NICN1	NA	NA	NA	0,00341524	NA	NA	NA	NA	NA
ILMN_2284744	HNMT	NA	NA	NA	0,00341595	NA	NA	NA	NA	NA
ILMN_1683082	RPUSD1	NA	NA	NA	0,00356142	NA	NA	NA	NA	NA
ILMN_1723480	BST2	NA	NA	NA	0,00358543	NA	NA	NA	NA	NA
ILMN_2068104	TFPI2	NA	NA	NA	0,00366428	NA	NA	NA	NA	NA
ILMN_1705261	CAPN1	NA	NA	NA	0,00367209	NA	NA	NA	NA	NA
ILMN_1684771	PGRMC1	NA	NA	NA	0,00370746	NA	NA	NA	NA	NA
ILMN_2352580	MBD1	NA	NA	NA	0,0037533	NA	NA	NA	NA	NA
ILMN_2088172	POLR2B	NA	NA	NA	0,0037721	NA	NA	NA	NA	NA
ILMN_1719316	TMED3	NA	NA	NA	0,00378334	NA	NA	NA	NA	NA
ILMN_1696419	STOM	NA	NA	NA	0,00379045	NA	NA	NA	NA	NA
ILMN_2082810	BRD7	NA	NA	NA	0,00382273	NA	NA	NA	NA	NA
ILMN_1695606	EFNB3	NA	NA	NA	0,003845	NA	NA	NA	NA	NA
ILMN_1728256	SPAG9	NA	NA	0,00821	0,00386435	NA	NA	NA	NA	NA
ILMN_1718629	NRIP1	NA	NA	NA	0,00389005	NA	NA	NA	NA	NA
ILMN_1660345	NGRN	NA	NA	NA	0,0038935	NA	NA	NA	NA	NA
ILMN_1730816	GPR162	NA	NA	NA	0,00389548	NA	NA	NA	NA	NA
ILMN_1682953	PGAM4	NA	NA	NA	0,00391162	NA	NA	NA	NA	NA
ILMN_1689842	SC4MOL	NA	NA	NA	0,00393374	NA	NA	NA	NA	NA
ILMN_1693620	PGM3	NA	NA	NA	0,00393777	NA	NA	NA	NA	NA
ILMN_2311989	CUTA	NA	NA	NA	0,00398677	NA	NA	NA	NA	NA
ILMN_2129563	LDLRAD3	NA	NA	NA	0,00402045	NA	NA	NA	NA	NA
ILMN_1781290	RHOA	NA	NA	NA	0,00402177	NA	NA	NA	NA	NA
ILMN_1800131	LOC652826	NA	NA	NA	0,00405868	NA	NA	NA	NA	NA
ILMN_1785795	METAP1	NA	NA	0,00082972	0,00410881	NA	NA	NA	NA	NA
ILMN_1730329	CRSP6	NA	NA	NA	0,00412501	NA	NA	NA	NA	NA
ILMN_1717490	RPL6	NA	NA	NA	0,00415116	NA	NA	NA	NA	NA
ILMN_1682864	SPSB3	NA	NA	NA	0,0042738	NA	NA	NA	NA	NA
ILMN_1657423	SPG21	NA	NA	NA	0,004307	NA	NA	NA	NA	NA
ILMN_1727495	L3MBTL3	NA	NA	NA	0,00432747	NA	NA	NA	NA	NA
ILMN_1719664	PTPN21	NA	NA	NA	0,00439237	NA	NA	NA	NA	NA
ILMN_1800447	PHKB	NA	NA	NA	0,00440492	NA	NA	NA	NA	NA
ILMN_2366654	NR5A2	NA	NA	NA	0,0044765	NA	NA	NA	NA	NA
ILMN_1722809	NRCAM	NA	NA	NA	0,00447678	NA	NA	NA	NA	NA
ILMN_1780382	LOC653566	NA	NA	NA	0,004488	NA	NA	NA	NA	NA
ILMN_1701032	TFPI	NA	NA	NA	0,0045004	NA	NA	NA	NA	NA
ILMN_1658472	APH1A	NA	NA	NA	0,00452705	NA	NA	NA	NA	NA
ILMN_3239181	ITPRIP	NA	NA	NA	0,0045528	NA	NA	NA	NA	NA
ILMN_1779258	LOC644774	NA	NA	NA	0,00458349	NA	NA	NA	NA	NA
ILMN_1749109	PSAP	NA	NA	NA	0,00460816	NA	NA	NA	NA	NA
ILMN_1748124	TSC22D3	NA	NA	0,00969242	0,00461204	NA	NA	NA	NA	NA
ILMN_1755737	TRABD	NA	NA	NA	0,00464191	NA	NA	NA	NA	NA
ILMN_3289090	LOC728059	NA	NA	NA	0,004674	NA	NA	NA	NA	NA
ILMN_3240838	SLC25A6	NA	NA	NA	0,00467736	NA	NA	NA	NA	NA
ILMN_1782983	KLF11	NA	NA	NA	0,0046809	NA	NA	NA	NA	NA
ILMN_2373982	PICK1	NA	NA	NA	0,00468436	NA	NA	NA	NA	NA
ILMN_1758548	NEK7	NA	NA	NA	0,00473663	NA	NA	NA	NA	NA
ILMN_2374164	HERPUD1	NA	NA	0,00068793	0,00474425	NA	NA	NA	NA	NA
ILMN_1765326	DGKD	NA	NA	NA	0,00494074	NA	NA	NA	NA	NA
ILMN_1730930	KIAA0652	NA	NA	NA	0,00496495	NA	NA	NA	NA	NA
ILMN_1814573	FTSJD1	NA	NA	NA	0,0049651	NA	NA	NA	NA	NA
ILMN_1696276	ZNF653	NA	NA	NA	0,00496931	NA	NA	NA	NA	NA
ILMN_1680624	CREG1	NA	NA	NA	0,00497296	NA	NA	NA	NA	NA
ILMN_3243253	LPAR3	NA	NA	NA	0,00498072	NA	NA	NA	NA	NA
ILMN_1815239	CLK2	NA	NA	NA	0,00499814	NA	NA	NA	NA	NA
ILMN_3239177	MEX3A	NA	NA	NA	0,00502116	NA	NA	NA	NA	NA
ILMN_2405031	TRIM24	NA	NA	NA	0,00502164	NA	NA	NA	NA	NA
ILMN_1721113	HLA-C	NA	NA	NA	0,00503194	NA	NA	NA	NA	NA
ILMN_1703324	PDSS1	NA	NA	NA	0,0050839	NA	NA	NA	NA	NA
ILMN_1740685	LOC652541	NA	NA	NA	0,00508587	NA	NA	NA	NA	NA
ILMN_2184789	HSCB	NA	NA	NA	0,00509703	NA	NA	NA	NA	NA
ILMN_1678652	C10orf61	NA	NA	NA	0,00512085	NA	NA	NA	NA	NA
ILMN_1791217	FAM40A	NA	NA	NA	0,00516069	NA	NA	NA	NA	NA
ILMN_3219340	LOC100131713	NA	NA	NA	0,00523121	NA	NA	NA	NA	NA
ILMN_1755077	HEBP2	NA	NA	NA	0,0053559	NA	NA	NA	NA	NA
ILMN_1659537	MED8	NA	NA	NA	0,00537254	NA	NA	NA	NA	NA
ILMN_2334760	ARMCX3	NA	NA	NA	0,00540507	NA	NA	NA	NA	NA
ILMN_2317658	SLC3A2	NA	NA	NA	0,00545649	NA	NA	NA	NA	NA
ILMN_1655635	METTL3	NA	NA	NA	0,00547333	NA	NA	NA	NA	NA

ILMN_1700268	QPRT	NA	NA	NA	0,005507	NA	NA	NA	NA	NA
ILMN_1670439	FYTTD1	NA	NA	NA	0,0055181	NA	NA	NA	NA	NA
ILMN_1657317	POLR2J	NA	NA	0,00120395	0,00560092	NA	NA	NA	NA	NA
ILMN_2403906	ARFIP1	NA	NA	NA	0,00560535	NA	NA	NA	NA	NA
ILMN_1743130	PTGFRN	NA	NA	NA	0,00563855	NA	NA	NA	NA	NA
ILMN_1763091	C14orf43	NA	NA	NA	0,00564257	NA	NA	NA	NA	NA
ILMN_1713406	FAM39DP	NA	NA	NA	0,00574041	NA	NA	NA	NA	NA
ILMN_1766115	PLEKHF2	NA	NA	NA	0,00577916	NA	NA	NA	NA	NA
ILMN_1708375	IRF1	NA	NA	NA	0,00579884	NA	NA	NA	NA	NA
ILMN_1688158	CYB5R4	NA	NA	0,00249886	0,0058191	NA	NA	NA	NA	NA
ILMN_1655952	FAM39E	NA	NA	NA	0,00593515	NA	NA	NA	NA	NA
ILMN_1741954	SMYD3	NA	NA	NA	0,00595857	NA	NA	NA	NA	NA
ILMN_1749866	GATA4	NA	NA	0,00873327	0,00596939	NA	NA	NA	NA	NA
ILMN_1889215		NA	NA	NA	0,00597323	NA	NA	NA	NA	NA
ILMN_1732410	SLC16A9	NA	NA	NA	0,00611242	NA	NA	NA	NA	NA
ILMN_1671693	C18orf10	NA	NA	NA	0,00612096	NA	NA	NA	NA	NA
ILMN_3293159	LOC100132767	NA	NA	NA	0,0061244	NA	NA	NA	NA	NA
ILMN_2194448	STT3B	NA	NA	NA	0,00613222	NA	NA	NA	NA	NA
ILMN_1662097	SLC25A43	NA	NA	NA	0,00619859	NA	NA	NA	NA	NA
ILMN_1669070	MIPEP	NA	NA	NA	0,00620641	NA	NA	NA	NA	NA
ILMN_1752340	ARF5	NA	NA	NA	0,00622282	NA	NA	NA	NA	NA
ILMN_2210729	STYXL1	NA	NA	NA	0,00626156	NA	NA	NA	NA	NA
ILMN_1708906	C2orf29	NA	NA	0,00575252	0,00630861	NA	NA	NA	NA	NA
ILMN_1679041	SLC3A2	NA	NA	NA	0,00634088	NA	NA	NA	NA	NA
ILMN_1768751	MTA3	NA	NA	NA	0,00634232	NA	NA	NA	NA	NA
ILMN_1685602	TMEM41A	NA	NA	NA	0,00639036	NA	NA	NA	NA	NA
ILMN_1725992	RAPGEF6	NA	NA	NA	0,00639744	NA	NA	NA	NA	NA
ILMN_1768181	TOR3A	NA	NA	NA	0,00649371	NA	NA	NA	NA	NA
ILMN_1801124	KIAA1826	NA	NA	NA	0,00649759	NA	NA	NA	NA	NA
ILMN_1700025	LOC732007	NA	NA	0,00964744	0,00651683	NA	NA	NA	NA	NA
ILMN_1721669	IDH3B	NA	NA	NA	0,0065498	NA	NA	NA	NA	NA
ILMN_2366634	PKM2	NA	NA	NA	0,00655195	NA	NA	NA	NA	NA
ILMN_1800412	BMP1	NA	NA	NA	0,00655448	NA	NA	NA	NA	NA
ILMN_1659801	ATP6V1C1	NA	NA	NA	0,00655959	NA	NA	NA	NA	NA
ILMN_1709750	SUSD1	NA	NA	NA	0,00657844	NA	NA	NA	NA	NA
ILMN_1726456	SLC3A2	NA	NA	NA	0,00658161	NA	NA	NA	NA	NA
ILMN_1757384	RAN	NA	NA	1,00E-04	0,00664832	NA	NA	NA	NA	NA
ILMN_1699082	MAPKAPK5	NA	NA	NA	0,00670941	NA	NA	NA	NA	NA
ILMN_1805192	ITPRIP	NA	NA	NA	0,00672347	NA	NA	NA	NA	NA
ILMN_1749875	LOC728715	7,07E-05	NA	7,07E-05	0,00678305	NA	NA	NA	NA	NA
ILMN_1676759	DDX27	NA	NA	0,00996655	0,00682042	NA	NA	NA	NA	NA
ILMN_1767642	C11orf46	NA	NA	NA	0,00683501	NA	NA	NA	NA	NA
ILMN_1741841	KIAA1429	NA	NA	NA	0,00685416	NA	NA	NA	NA	NA
ILMN_1727553	C5orf54	NA	NA	NA	0,00690596	NA	NA	NA	NA	NA
ILMN_1683059	SIRT5	NA	NA	NA	0,00700746	NA	NA	NA	NA	NA
ILMN_2112417	PGAM1	NA	NA	NA	0,00701462	NA	NA	NA	NA	NA
ILMN_1705032	SEH1L	NA	NA	0,00858055	0,0070668	NA	NA	NA	NA	NA
ILMN_2400183	DPH5	NA	NA	NA	0,00708289	NA	NA	NA	NA	NA
ILMN_2361603	NDRG2	NA	NA	NA	0,00713744	NA	NA	NA	NA	NA
ILMN_1780560	MYO18A	NA	NA	NA	0,00713868	NA	NA	NA	NA	NA
ILMN_1750409	RAB9A	NA	NA	0,00434809	0,0071565	NA	NA	NA	NA	NA
ILMN_2083833	CNOT6L	NA	NA	NA	0,00730115	NA	NA	NA	NA	NA
ILMN_1662768	NHEDC2	NA	NA	NA	0,00732386	NA	NA	NA	NA	NA
ILMN_1791057	IFNAR2	NA	NA	NA	0,00745068	NA	NA	NA	NA	NA
ILMN_1771084	ACSM3	NA	NA	NA	0,00745093	NA	NA	NA	NA	NA
ILMN_1756701	DHRS11	NA	NA	0,00292433	0,00747775	NA	NA	NA	NA	NA
ILMN_1719163	ZNF557	NA	NA	NA	0,00748469	NA	NA	NA	NA	NA
ILMN_2187727	NOC3L	NA	NA	NA	0,00761023	NA	NA	NA	NA	NA
ILMN_1809191	RAP1A	NA	NA	NA	0,00762305	NA	NA	NA	NA	NA
ILMN_2352303	RASSF2	NA	NA	NA	0,00765614	NA	NA	NA	NA	NA
ILMN_2083334	PMS2L5	NA	NA	NA	0,00768859	NA	NA	NA	NA	NA
ILMN_1787595	B3GALNT1	NA	NA	NA	0,00772217	NA	NA	NA	NA	NA
ILMN_1661593	FAM18B	NA	NA	0,00353478	0,00773105	NA	NA	NA	NA	NA
ILMN_1706511	TEF	NA	NA	NA	0,00779078	NA	NA	NA	NA	NA
ILMN_1736752	COMTD1	NA	NA	NA	0,00779861	NA	NA	NA	NA	NA
ILMN_1683026	PSMB10	NA	NA	NA	0,00782991	NA	NA	NA	NA	NA
ILMN_2367063	TPK1	NA	NA	NA	0,00788081	NA	NA	NA	NA	NA
ILMN_2046073	LCT	NA	NA	NA	0,00789002	NA	NA	NA	NA	NA
ILMN_1653143	ECD	NA	NA	NA	0,00793241	NA	NA	NA	NA	NA
ILMN_1657796	STMN1	NA	NA	NA	0,00794168	NA	NA	NA	NA	NA
ILMN_1809437	RHBDD2	NA	NA	NA	0,00795126	NA	NA	NA	NA	NA
ILMN_1703123	AXUD1	NA	NA	NA	0,00799163	NA	NA	NA	NA	NA
ILMN_1739770	CRSP9	NA	NA	NA	0,00803535	NA	NA	NA	NA	NA
ILMN_1718063	LIPA	NA	NA	NA	0,00811241	NA	NA	NA	NA	NA

ILMN_2094776	CCNL1	NA	NA	NA	0,00811833	NA	NA	NA	NA	NA
ILMN_2102951	FAHD2A	NA	NA	0,00864064	0,008131	NA	NA	NA	NA	NA
ILMN_1665945	ACBD3	NA	NA	0,00568802	0,00813578	NA	NA	NA	NA	NA
ILMN_2313926	CDC42SE2	NA	NA	NA	0,00815764	NA	NA	NA	NA	NA
ILMN_1814856	C9orf7	NA	NA	NA	0,00818403	NA	NA	NA	NA	NA
ILMN_2339006	KIAA0564	NA	NA	NA	0,00818657	NA	NA	NA	NA	NA
ILMN_2188862	GDF15	NA	NA	NA	0,00825449	NA	NA	NA	NA	NA
ILMN_1656373	BNC2	NA	NA	0,00127799	0,00828411	NA	NA	NA	NA	NA
ILMN_1710756	ENO1	NA	NA	NA	0,00834679	NA	NA	NA	NA	NA
ILMN_1803788	LGALS3	NA	NA	0,00386592	0,00835526	NA	NA	NA	NA	NA
ILMN_1677162	SFRS13A	0,001770699	NA	0,0017707	0,00838581	NA	NA	NA	NA	NA
ILMN_3244611	LOC100132060	NA	NA	NA	0,00843575	NA	NA	NA	NA	NA
ILMN_1770085	BTG2	NA	NA	NA	0,00844931	NA	NA	NA	NA	NA
ILMN_1790309	PINX1	0,002762049	NA	0,00276205	0,00848172	NA	NA	NA	NA	NA
ILMN_3248443	SNHG6	NA	NA	NA	0,00849754	NA	NA	NA	NA	NA
ILMN_1790637	C11orf80	NA	NA	NA	0,00851911	NA	NA	NA	NA	NA
ILMN_1751234	C1GALT1C1	NA	NA	NA	0,00858082	NA	NA	NA	NA	NA
ILMN_1671482	GALM	NA	NA	NA	0,00859138	NA	NA	NA	NA	NA
ILMN_2312275	SRP54	NA	NA	NA	0,00863655	NA	NA	NA	NA	NA
ILMN_3197732	LOC441642	NA	NA	NA	0,0086369	NA	NA	NA	NA	NA
ILMN_3272476	LOC100130624	NA	NA	NA	0,00866421	NA	NA	NA	NA	NA
ILMN_3227811	LOC729423	NA	NA	NA	0,0086663	NA	NA	NA	NA	NA
ILMN_1673024	RBM15B	NA	NA	NA	0,00872956	NA	NA	NA	NA	NA
ILMN_2411731	HPS5	NA	NA	NA	0,00873687	NA	NA	NA	NA	NA
ILMN_3246330	LOC100133578	NA	NA	NA	0,0087771	NA	NA	NA	NA	NA
ILMN_1730516	TMEM133	NA	NA	NA	0,00878522	NA	NA	NA	NA	NA
ILMN_1809488	SPCS2	NA	NA	NA	0,00880168	NA	NA	NA	NA	NA
ILMN_1725441	NFATC2IP	NA	NA	NA	0,0088078	NA	NA	NA	NA	NA
ILMN_1659952	MTMR2	NA	NA	0,00087059	0,00885597	NA	NA	NA	NA	NA
ILMN_1660986	PER3	NA	NA	NA	0,00890742	NA	NA	NA	NA	NA
ILMN_1734810	PJA1	NA	NA	NA	0,00899017	NA	NA	NA	NA	NA
ILMN_1712452	KIF20B	NA	NA	NA	0,00899895	NA	NA	NA	NA	NA
ILMN_2072541	RAB11FIP2	NA	NA	NA	0,00904213	NA	NA	NA	NA	NA
ILMN_1691418	CDRT4	NA	NA	NA	0,00905765	NA	NA	NA	NA	NA
ILMN_1727805	SYNGR1	NA	NA	NA	0,00907384	NA	NA	NA	NA	NA
ILMN_1694323	GLE1	NA	NA	NA	0,00908277	NA	NA	NA	NA	NA
ILMN_1684017	GYG2	NA	NA	NA	0,00909028	NA	NA	NA	NA	NA
ILMN_1744046	DIAPH2	NA	NA	NA	0,00915278	NA	NA	NA	NA	NA
ILMN_1714417	PSEN2	NA	NA	NA	0,00915777	NA	NA	NA	NA	NA
ILMN_2043918	DLEU1	NA	NA	NA	0,00916279	NA	NA	NA	NA	NA
ILMN_3278132	LOC440487	NA	NA	NA	0,00922986	NA	NA	NA	NA	NA
ILMN_1764314	FGD1	NA	NA	NA	0,00926472	NA	NA	NA	NA	NA
ILMN_1725071	CCDC12	NA	NA	NA	0,00930665	NA	NA	NA	NA	NA
ILMN_1661366	PGAM1	NA	NA	NA	0,0093473	NA	NA	NA	NA	NA
ILMN_1783593	CCL13	NA	NA	0,00281899	0,00938903	NA	NA	NA	NA	NA
ILMN_2220283	HNRPA1L-2	NA	NA	0,00228315	0,00941375	NA	NA	NA	NA	NA
ILMN_1740197	ZNF234	NA	NA	NA	0,00941507	NA	NA	NA	NA	NA
ILMN_2168314	PGM3	NA	NA	NA	0,00942396	NA	NA	NA	NA	NA
ILMN_1802753	TSSC4	NA	NA	NA	0,00944543	NA	NA	NA	NA	NA
ILMN_2367215	PRCP	0,005642128	NA	0,00564213	0,00947079	NA	NA	NA	NA	NA
ILMN_1708382	C3orf75	NA	NA	NA	0,0095319	NA	NA	NA	NA	NA
ILMN_1744403	KCNIP3	NA	NA	NA	0,00956777	NA	NA	NA	NA	NA
ILMN_1708605	LOC652481	NA	NA	NA	0,00956928	NA	NA	NA	NA	NA
ILMN_1781135	STAM2	NA	NA	NA	0,00956987	NA	NA	NA	NA	NA
ILMN_1806294	RPS6KA3	NA	NA	NA	0,00961409	NA	NA	NA	NA	NA
ILMN_1728934	PRC1	NA	NA	NA	0,00963134	NA	NA	NA	NA	NA
ILMN_1768127	EBNA1BP2	NA	NA	0,00461422	0,00964519	NA	NA	NA	NA	NA
ILMN_2332713	SLC25A3	NA	NA	NA	0,00965052	NA	NA	NA	NA	NA
ILMN_1655312	HPS5	NA	NA	NA	0,00967677	NA	NA	NA	NA	NA
ILMN_1718607	TSPAN4	NA	NA	NA	0,00969553	NA	NA	NA	NA	NA
ILMN_1721138	GRPEL2	NA	NA	NA	0,00972381	NA	NA	NA	NA	NA
ILMN_1708660	RWDD4A	NA	NA	NA	0,00977816	NA	NA	NA	NA	NA
ILMN_2381697	P4HA2	NA	NA	NA	0,00980875	NA	NA	NA	NA	NA
ILMN_1709683	RASSF2	NA	NA	NA	0,00986035	NA	NA	NA	NA	NA
ILMN_1777976	SLC25A26	NA	NA	0,00079254	0,00987052	NA	NA	NA	NA	NA
ILMN_1820767		NA	NA	NA	0,00987317	NA	NA	NA	NA	NA
ILMN_1766446	C6orf48	NA	NA	NA	0,00991303	NA	NA	NA	NA	NA
ILMN_1755383	LRRC1	NA	NA	NA	0,00994357	NA	NA	NA	NA	NA
ILMN_1774689	TUSC3	NA	NA	NA	0,00994696	NA	NA	NA	NA	NA
ILMN_3272500	IFI27L1	NA	NA	NA	0,00994718	NA	NA	NA	NA	NA
ILMN_1752197	PDDC1	NA	NA	NA	0,00997487	NA	NA	NA	NA	NA
ILMN_3236498	LOC253039	NA	NA	2,50E-05	NA	NA	NA	NA	NA	NA
ILMN_1679971	CCDC123	2,63E-05	NA	2,63E-05	NA	NA	NA	NA	NA	NA
ILMN_2326713	CD151	2,76E-05	NA	2,76E-05	NA	NA	NA	NA	NA	NA

ILMN_1784218	DDX23	6,72E-05	NA	6,72E-05	NA	NA	NA	NA	NA	NA
ILMN_2336094	ODZ3	7,55E-05	NA	7,55E-05	NA	NA	NA	NA	NA	NA
ILMN_1709114	MAP3K7IP1	NA	NA	9,20E-05	NA	NA	NA	NA	NA	NA
ILMN_2069945	SNRNP27	NA	NA	0,00011783	NA	NA	NA	NA	NA	NA
ILMN_1811195	ZNF211	0,000130214	NA	0,00013021	NA	NA	NA	NA	NA	NA
ILMN_3238676	ULBP2	NA	NA	0,00013263	NA	NA	NA	NA	NA	NA
ILMN_1737992	CRAT	0,000139041	NA	0,00013904	NA	NA	NA	NA	NA	NA
ILMN_1705849	SPR	NA	NA	0,00018252	NA	NA	NA	NA	NA	NA
ILMN_1806456	C14orf45	0,000192901	NA	0,0001929	NA	NA	NA	NA	NA	NA
ILMN_1716026	ARMCX6	0,00021447	NA	0,00021447	NA	NA	NA	NA	NA	NA
ILMN_1792456	CCDC104	0,000220231	NA	0,00022023	NA	NA	NA	NA	NA	NA
ILMN_1758173	TMEM99	NA	NA	0,0002305	NA	NA	NA	NA	NA	NA
ILMN_1744138	CHCHD7	0,000285265	NA	0,00028527	NA	NA	NA	NA	NA	NA
ILMN_3301197	MAPKSP1	0,000293219	NA	0,00029322	NA	NA	NA	NA	NA	NA
ILMN_1766637	GLA	NA	NA	0,00030895	NA	NA	NA	NA	NA	NA
ILMN_2290618	SEC22C	0,000324565	NA	0,00032457	NA	NA	NA	NA	NA	NA
ILMN_1667589	ZNF283	0,000328725	NA	0,00032873	NA	NA	NA	NA	NA	NA
ILMN_1700168	LARS2	0,000399743	NA	0,00039974	NA	NA	NA	NA	NA	NA
ILMN_1695432	TPST2	NA	NA	0,00042334	NA	NA	NA	NA	NA	NA
ILMN_1730464	DNAL1	NA	NA	0,00042808	NA	NA	NA	NA	NA	NA
ILMN_1715635	ATP6V0E1	NA	NA	0,00048465	NA	NA	NA	NA	NA	NA
ILMN_2222065	FZD1	0,000487714	NA	0,00048771	NA	NA	NA	NA	NA	NA
ILMN_2056479	TXNL2	0,000487971	NA	0,00048797	NA	NA	NA	NA	NA	NA
ILMN_1745501	DNALI1	0,000514086	NA	0,00051409	NA	NA	NA	NA	NA	NA
ILMN_1690911	C16orf87	0,000523512	NA	0,00052351	NA	NA	NA	NA	NA	NA
ILMN_2366177	IFT122	0,000523773	NA	0,00052377	NA	NA	NA	NA	NA	NA
ILMN_1726547	MAP3K5	NA	NA	0,00055388	NA	NA	NA	NA	NA	NA
ILMN_1798083	CHERP	NA	NA	0,00058446	NA	NA	NA	NA	NA	NA
ILMN_2267787	HPS1	0,00060643	NA	0,00060643	NA	NA	NA	NA	NA	NA
ILMN_1726222	FLOT2	NA	NA	0,00060849	NA	NA	NA	NA	NA	NA
ILMN_2260991	TSPO	NA	NA	0,00062962	NA	NA	NA	NA	NA	NA
ILMN_2150258	ZFP36L2	0,000636397	NA	0,0006364	NA	NA	NA	NA	NA	NA
ILMN_1794085	SAPS1	0,000648258	NA	0,00064826	NA	NA	NA	NA	NA	NA
ILMN_1752303	YY1AP1	0,000667891	NA	0,00066789	NA	NA	NA	NA	NA	NA
ILMN_1713499	WISP1	NA	NA	0,00067219	NA	NA	NA	NA	NA	NA
ILMN_1668408	AIFM1	0,000687737	NA	0,00068774	NA	NA	NA	NA	NA	NA
ILMN_1733288	C1RL	NA	NA	0,00068926	NA	NA	NA	NA	NA	NA
ILMN_1752213	TMEM60	NA	NA	0,00073311	NA	NA	NA	NA	NA	NA
ILMN_1813746	CORO2A	NA	NA	0,00075194	NA	NA	NA	NA	NA	NA
ILMN_2329679	TPST2	NA	NA	0,00078819	NA	NA	NA	NA	NA	NA
ILMN_3307858	STIM2	NA	NA	0,00079225	NA	NA	NA	NA	NA	NA
ILMN_1713471	ENOX1	NA	NA	0,00080759	NA	NA	NA	NA	NA	NA
ILMN_1680579	ATP2B4	0,000841609	NA	0,00084161	NA	NA	NA	NA	NA	NA
ILMN_1805481	TRMT11	0,000859977	NA	0,00085998	NA	NA	NA	NA	NA	NA
ILMN_1751143	C7orf23	0,000874313	0,00087431	0,00087431	NA	NA	NA	NA	NA	NA
ILMN_2340721	TMEM134	NA	NA	0,00087864	NA	NA	NA	NA	NA	NA
ILMN_1750144	C3orf19	NA	NA	0,00090353	NA	NA	NA	NA	NA	NA
ILMN_3210946	LOC645969	NA	NA	0,0009103	NA	NA	NA	NA	NA	NA
ILMN_1772207	LOC653377	NA	NA	0,00091044	NA	NA	NA	NA	NA	NA
ILMN_1691291	PIGS	0,000910557	NA	0,00091056	NA	NA	NA	NA	NA	NA
ILMN_1690101	FAIM	NA	NA	0,00092986	NA	NA	NA	NA	NA	NA
ILMN_1813766	RCL1	NA	NA	0,00093876	NA	NA	NA	NA	NA	NA
ILMN_1784766	MCM3AP	NA	NA	0,00093953	NA	NA	NA	NA	NA	NA
ILMN_1760779	ENSA	NA	NA	0,00096031	NA	NA	NA	NA	NA	NA
ILMN_2313821	AIFM1	0,000970633	NA	0,00097063	NA	NA	NA	NA	NA	NA
ILMN_2104356	COL1A2	NA	NA	0,00099805	NA	NA	NA	NA	NA	NA
ILMN_1719064	KCTD10	0,001025104	NA	0,0010251	NA	NA	NA	NA	NA	NA
ILMN_2232430	NMD3	0,001093271	NA	0,00109327	NA	NA	NA	NA	NA	NA
ILMN_2228873	STARD3NL	NA	NA	0,00109369	NA	NA	NA	NA	NA	NA
ILMN_1771376	PEA15	NA	NA	0,00114425	NA	NA	NA	NA	NA	NA
ILMN_1683305	COMMD2	NA	NA	0,00114885	NA	NA	NA	NA	NA	NA
ILMN_1688621	C9orf80	NA	NA	0,00118202	NA	NA	NA	NA	NA	NA
ILMN_3246608	CENPV	NA	NA	0,00119808	NA	NA	NA	NA	NA	NA
ILMN_1791388	ZNF787	NA	NA	0,00124042	NA	NA	NA	NA	NA	NA
ILMN_1729123	PPP2R4	NA	NA	0,00124616	NA	NA	NA	NA	NA	NA
ILMN_2343105	LIPT1	0,001259471	NA	0,00125947	NA	NA	NA	NA	NA	NA
ILMN_3249618	LOC100133477	NA	NA	0,00130076	NA	NA	NA	NA	NA	NA
ILMN_1805225	LPCAT3	NA	NA	0,00130847	NA	NA	NA	NA	NA	NA
ILMN_1691188	UIMC1	NA	NA	0,00133933	NA	NA	NA	NA	NA	NA
ILMN_1774596	BSCL2	0,001348864	0,00134886	0,00134886	NA	NA	NA	NA	NA	NA
ILMN_1720422	G3BP2	0,00135744	NA	0,00135744	NA	NA	NA	NA	NA	NA
ILMN_1687430	EIF2B4	NA	NA	0,00137631	NA	NA	NA	NA	NA	NA
ILMN_3240247	NOP10	NA	NA	0,00137904	NA	NA	NA	NA	NA	NA
ILMN_2094905	COMMD10	NA	NA	0,0014035	NA	NA	NA	NA	NA	NA

ILMN_3213792	LOC439953	NA	NA	0,0014184	NA	NA	NA	NA	NA	NA	
ILMN_3284114	LOC399748	0,001420841	NA	0,00142084	NA	NA	NA	NA	NA	NA	
ILMN_2251978	C21orf66	NA	NA	0,00142292	NA	NA	NA	NA	NA	NA	
ILMN_3288830	LOC100132918	NA	NA	0,00142628	NA	NA	NA	NA	NA	NA	
ILMN_2121816	GPR137B	NA	NA	0,0014464	NA	NA	NA	NA	NA	NA	
ILMN_1763080	QARS	NA	NA	0,00145806	NA	NA	NA	NA	NA	NA	
ILMN_2072178	ECHDC3	0,001506636	NA	0,00150664	NA	NA	NA	NA	NA	NA	
ILMN_1705900	ATXN10	NA	NA	0,00152444	NA	NA	NA	NA	NA	NA	
ILMN_1683927	ITGAE	NA	NA	0,00154524	NA	NA	NA	NA	NA	NA	
ILMN_1727813	BRP44	NA	NA	0,00155214	NA	NA	NA	NA	NA	NA	
ILMN_1799516	DNAJC9	0,001576563	NA	0,00157656	NA	NA	NA	NA	NA	NA	
ILMN_1684045	CDCA4	NA	NA	0,00158472	NA	NA	NA	NA	NA	NA	
ILMN_1813455	SP110	NA	NA	0,00158993	NA	NA	NA	NA	NA	NA	
ILMN_1731619	DAD1	NA	NA	0,00159119	NA	NA	NA	NA	NA	NA	
ILMN_1756920	ADAM15	NA	NA	0,00162866	NA	NA	NA	NA	NA	NA	
ILMN_1714384	PCCA	NA	NA	0,00165122	NA	NA	NA	NA	NA	NA	
ILMN_1745385	HNRPM	NA	NA	0,00165509	NA	NA	NA	NA	NA	NA	
ILMN_1803799	LOC649555	NA	NA	0,00166644	NA	NA	NA	NA	NA	NA	
ILMN_3231621	LOC730235	NA	0,00168417	0,00168417	NA	NA	NA	NA	NA	NA	
ILMN_1662362	LGR6	0,001690258	NA	0,00169026	NA	NA	NA	NA	NA	NA	
ILMN_3273493	LOC100130092	0,00172162	NA	0,00172162	NA	NA	NA	NA	NA	NA	
ILMN_1789342	NDUFS2	0,001725602	NA	0,0017256	NA	NA	NA	NA	NA	NA	
ILMN_1785272	COL1A2	NA	NA	0,00173479	NA	NA	NA	NA	NA	NA	
ILMN_1671314	UXT	NA	NA	0,00175257	NA	NA	NA	NA	NA	NA	
ILMN_1683811	TNPO3	NA	NA	0,00175265	NA	NA	NA	NA	NA	NA	
ILMN_1712517	ZNF696	NA	NA	0,00179936	NA	NA	NA	NA	NA	NA	
ILMN_1667418	LOC283953	NA	NA	0,00180563	NA	NA	NA	NA	NA	NA	
ILMN_2112049	DNLZ	0,001859424	NA	0,00185942	NA	NA	NA	NA	NA	NA	
ILMN_1807074	MIF	NA	NA	0,0018779	NA	NA	NA	NA	NA	NA	
ILMN_1657949	RHEB	0,001908619	NA	0,00190862	NA	NA	NA	NA	NA	NA	
ILMN_2125562	MOBK1B	NA	NA	0,00191735	NA	NA	NA	NA	NA	NA	
ILMN_3307648	CS	0,001918883	NA	0,00191888	NA	NA	NA	NA	NA	NA	
ILMN_2131493	VISA	NA	NA	0,0019289	NA	NA	NA	NA	NA	NA	
ILMN_1789407	FAM18B2	NA	NA	0,00194296	NA	NA	NA	NA	NA	NA	
ILMN_1705364	BAT3	0,001969483	NA	0,00196948	NA	NA	NA	NA	NA	NA	
ILMN_1799128	SLC30A9	NA	NA	0,00200696	NA	NA	NA	NA	NA	NA	
ILMN_1772644	EML3	NA	NA	0,00202391	NA	NA	NA	NA	NA	NA	
ILMN_1688606	RNFT1	0,002072626	NA	0,00207263	NA	NA	NA	NA	NA	NA	
ILMN_1808712	RNF40	0,002091537	NA	0,00209154	NA	NA	NA	NA	NA	NA	
ILMN_1799024	VAC14	0,00217261	NA	0,00217261	NA	NA	NA	NA	NA	NA	
ILMN_2175465	RSL24D1	0,002185641	NA	0,00218564	NA	NA	NA	NA	NA	NA	
ILMN_1681845	PAPD4	0,002225045	NA	0,00222505	NA	NA	NA	NA	NA	NA	
ILMN_2400583	USP9X	0,002226438	NA	0,00222644	NA	NA	NA	NA	NA	NA	
ILMN_1776260	LOC653505	NA	NA	0,00223677	NA	NA	NA	NA	NA	NA	
ILMN_1684440	PXN	NA	NA	0,00225001	NA	NA	NA	NA	NA	NA	
ILMN_1798657	TBL1XR1	NA	NA	0,00225547	NA	NA	NA	NA	NA	NA	
ILMN_1724897	C14orf93	NA	NA	0,00225787	NA	NA	NA	NA	NA	NA	
ILMN_1665423	ZFP91	NA	NA	0,00229834	NA	NA	NA	NA	NA	NA	
ILMN_2225537	PTGR1	NA	NA	0,00231279	NA	NA	NA	NA	NA	NA	
ILMN_1814022	NR1H3	NA	NA	0,00234072	NA	NA	NA	NA	NA	NA	
ILMN_1751079	TAP1	0,002351685	NA	0,00235169	NA	NA	NA	NA	NA	NA	
ILMN_1768197	ROD1	0,002362175	NA	0,00236218	NA	NA	NA	NA	NA	NA	
ILMN_1753010	PET112L	NA	NA	0,00238626	NA	NA	NA	NA	NA	NA	
ILMN_1757408	ZNF256	0,002416672	NA	0,00241667	NA	NA	NA	NA	NA	NA	
ILMN_2192281	CARD8	0,002419502	0,0024195	0,0024195	NA	NA	NA	NA	NA	NA	
ILMN_1794522	EIF5A	NA	NA	0,00245052	NA	NA	NA	NA	NA	NA	
ILMN_1667519	RRAS2	NA	NA	0,00246001	NA	NA	NA	NA	NA	NA	
ILMN_1778401	HLA-B	0,002460787	NA	0,00246079	NA	NA	NA	NA	NA	NA	
ILMN_1698233	RWDD2B	NA	NA	0,00246498	NA	NA	NA	NA	NA	NA	
ILMN_1690282	CRADD	NA	NA	0,00248919	NA	NA	NA	NA	NA	NA	
ILMN_1667893	TNS3	0,002492609	NA	0,00249261	NA	NA	NA	NA	NA	NA	
ILMN_1690066	TIGD2	0,002493216	NA	0,00249322	NA	NA	NA	NA	NA	NA	
ILMN_1706057	CS	0,002498952	NA	0,00249895	NA	NA	NA	NA	NA	NA	
ILMN_1804332	GPR137	0,002512033	NA	0,00251203	NA	NA	NA	NA	NA	NA	
ILMN_1774547	MPRIP	0,002583885	NA	0,00258389	NA	NA	NA	NA	NA	NA	
ILMN_1808238	RBPMS2	NA	NA	0,0026044	NA	NA	NA	NA	NA	NA	
ILMN_2071937	ATP6V0E1	NA	NA	0,00263524	NA	NA	NA	NA	NA	NA	
ILMN_2344204	PRR13	0,002636733	NA	0,00263673	NA	NA	NA	NA	NA	NA	
ILMN_1729117	COL5A2	NA	NA	0,00271775	NA	NA	NA	NA	NA	NA	
ILMN_1720819	LOC653566	NA	NA	0,00272014	NA	NA	NA	NA	NA	NA	
ILMN_1703695	C19orf12	NA	NA	0,00272813	NA	NA	NA	NA	NA	NA	
ILMN_1803464	PHTF1	0,002740021	NA	0,00274002	NA	NA	NA	NA	NA	NA	
ILMN_1781231	SLC25A38	NA	NA	0,00277661	NA	NA	NA	NA	NA	NA	
ILMN_2381753	G3BP2	NA	NA	0,00281317	NA	NA	NA	NA	NA	NA	

ILMN_1674560	GBA2	0,002813274	NA	0,00281327	NA	NA	NA	NA	NA	NA
ILMN_1802971	FAM160B2	NA	NA	0,00282178	NA	NA	NA	NA	NA	NA
ILMN_1760676	MORF4L1	NA	NA	0,0028359	NA	NA	NA	NA	NA	NA
ILMN_1763837	ANPEP	NA	NA	0,00287893	NA	NA	NA	NA	NA	NA
ILMN_2358278	RSRC2	NA	0,00291887	0,00291887	NA	NA	NA	NA	NA	NA
ILMN_1658411	CHD4	NA	NA	0,00293243	NA	NA	NA	NA	NA	NA
ILMN_1717154	AQR	0,002992953	0,00299295	0,00299295	NA	NA	NA	NA	NA	NA
ILMN_1701933	SNCA	0,003030492	NA	0,00303049	NA	NA	NA	NA	NA	NA
ILMN_3233179	LOC728969	NA	NA	0,00304545	NA	NA	NA	NA	NA	NA
ILMN_1675130	NFIC	NA	NA	0,00304586	NA	NA	NA	NA	NA	NA
ILMN_1767111	ANO10	NA	NA	0,00307145	NA	NA	NA	NA	NA	NA
ILMN_1709891	LOC647074	NA	NA	0,00308635	NA	NA	NA	NA	NA	NA
ILMN_1792435	STAG1	NA	NA	0,00314091	NA	NA	NA	NA	NA	NA
ILMN_1735955	LOC644033	NA	NA	0,00314334	NA	NA	NA	NA	NA	NA
ILMN_1730631	C2orf44	NA	NA	0,0031795	NA	NA	NA	NA	NA	NA
ILMN_3258424	LOC100129118	NA	NA	0,00319964	NA	NA	NA	NA	NA	NA
ILMN_1815705	LZTFL1	NA	NA	0,00322413	NA	NA	NA	NA	NA	NA
ILMN_2077886	C1orf109	0,003229874	0,00322987	0,00322987	NA	NA	NA	NA	NA	NA
ILMN_3246953	FTSJD2	NA	NA	0,00328875	NA	NA	NA	NA	NA	NA
ILMN_1731374	CPE	0,003331014	NA	0,00333101	NA	NA	NA	NA	NA	NA
ILMN_1673795	HSD17B4	NA	0,00337164	0,00337164	NA	NA	NA	NA	NA	NA
ILMN_1761312	CRHBP	0,003384498	NA	0,0033845	NA	NA	NA	NA	NA	NA
ILMN_2173909	ZNF14	0,003388153	NA	0,00338815	NA	NA	NA	NA	NA	NA
ILMN_1738369	TUFM	NA	0,00339852	0,00339852	NA	NA	NA	NA	NA	NA
ILMN_2407589	CTDP1	NA	NA	0,0034502	NA	NA	NA	NA	NA	NA
ILMN_1714296	DNAH2	NA	NA	0,00345356	NA	NA	NA	NA	NA	NA
ILMN_1671703	ACTA2	NA	NA	0,00345539	NA	NA	NA	NA	NA	NA
ILMN_1657129	SKAP2	NA	NA	0,00347563	NA	NA	NA	NA	NA	NA
ILMN_1793615	ME3	NA	NA	0,00349067	NA	NA	NA	NA	NA	NA
ILMN_1701930	EEF1B2	NA	NA	0,00350335	NA	NA	NA	NA	NA	NA
ILMN_2227968	NTHL1	NA	NA	0,00354364	NA	NA	NA	NA	NA	NA
ILMN_1750092	SEPSECS	NA	NA	0,00358816	NA	NA	NA	NA	NA	NA
ILMN_3305949	LOC730246	NA	NA	0,0035921	NA	NA	NA	NA	NA	NA
ILMN_2119535	RIPK1	0,003592833	NA	0,00359283	NA	NA	NA	NA	NA	NA
ILMN_1747857	SMARCE1	NA	NA	0,00363881	NA	NA	NA	NA	NA	NA
ILMN_1752947	C17orf79	0,003647537	NA	0,00364754	NA	NA	NA	NA	NA	NA
ILMN_1769704	FLJ39632	NA	NA	0,00365441	NA	NA	NA	NA	NA	NA
ILMN_1655418	CAPNS1	0,003666178	NA	0,00366618	NA	NA	NA	NA	NA	NA
ILMN_1742889	WDR77	0,003706566	0,00370657	0,00370657	NA	NA	NA	NA	NA	NA
ILMN_1654457	TXN2	NA	NA	0,00374129	NA	NA	NA	NA	NA	NA
ILMN_1805543	ADAMTS9	0,003748119	NA	0,00374812	NA	NA	NA	NA	NA	NA
ILMN_1757660	CAPS	NA	0,00376679	0,00376679	NA	NA	NA	NA	NA	NA
ILMN_2414399	NME1	NA	NA	0,00377709	NA	NA	NA	NA	NA	NA
ILMN_1714759	CNIH4	NA	NA	0,00378124	NA	NA	NA	NA	NA	NA
ILMN_1677237	CHCHD9	NA	NA	0,00378641	NA	NA	NA	NA	NA	NA
ILMN_1651433	DCK	NA	NA	0,00378696	NA	NA	NA	NA	NA	NA
ILMN_1678086	CCDC74A	NA	NA	0,00384345	NA	NA	NA	NA	NA	NA
ILMN_3269775	C14orf109	NA	NA	0,003885	NA	NA	NA	NA	NA	NA
ILMN_1742866	F2R	0,003944098	NA	0,0039441	NA	NA	NA	NA	NA	NA
ILMN_1685551	CHD4	NA	NA	0,00396854	NA	NA	NA	NA	NA	NA
ILMN_1732615	ASMTL	NA	NA	0,00399079	NA	NA	NA	NA	NA	NA
ILMN_2191681	CHCHD2	NA	NA	0,00404636	NA	NA	NA	NA	NA	NA
ILMN_1758626	IDS	NA	NA	0,00408838	NA	NA	NA	NA	NA	NA
ILMN_1713752	SERINC3	NA	NA	0,00410814	NA	NA	NA	NA	NA	NA
ILMN_2374159	HERPUD1	NA	NA	0,00416001	NA	NA	NA	NA	NA	NA
ILMN_1715661	TFAM	0,004176334	0,00417633	0,00417633	NA	NA	NA	NA	NA	NA
ILMN_1746716	LOC643035	NA	NA	0,00420041	NA	NA	NA	NA	NA	NA
ILMN_1796106	MGAT4B	NA	0,00421251	0,00421251	NA	NA	NA	NA	NA	NA
ILMN_1895915		NA	NA	0,00422218	NA	NA	NA	NA	NA	NA
ILMN_1761068	MGC52000	NA	NA	0,004225	NA	NA	NA	NA	NA	NA
ILMN_1663401	LARP6	NA	NA	0,00429113	NA	NA	NA	NA	NA	NA
ILMN_2307032	OSBPL5	0,004297125	NA	0,00429713	NA	NA	NA	NA	NA	NA
ILMN_1787705	ATP6V1B2	NA	NA	0,00430666	NA	NA	NA	NA	NA	NA
ILMN_1654032	ZER1	NA	NA	0,00432241	NA	NA	NA	NA	NA	NA
ILMN_1728071	KRAS	NA	NA	0,00435825	NA	NA	NA	NA	NA	NA
ILMN_1799856	NOMO2	NA	NA	0,00439027	NA	NA	NA	NA	NA	NA
ILMN_1813625	TRIM25	NA	NA	0,00442414	NA	NA	NA	NA	NA	NA
ILMN_1807171	PLA2G5	NA	NA	0,0044422	NA	NA	NA	NA	NA	NA
ILMN_2189222	KLHL8	NA	NA	0,00448919	NA	NA	NA	NA	NA	NA
ILMN_1688127	LOC341457	NA	NA	0,00457721	NA	NA	NA	NA	NA	NA
ILMN_1692177	TSC22D1	NA	NA	0,00461994	NA	NA	NA	NA	NA	NA
ILMN_1726030	GPX7	0,004633377	NA	0,00463338	NA	NA	NA	NA	NA	NA
ILMN_1714349	GLCE	NA	NA	0,00463616	NA	NA	NA	NA	NA	NA
ILMN_1710758	RNF20	NA	NA	0,00468476	NA	NA	NA	NA	NA	NA

ILMN_1814526	ADD3	NA	NA	0,00471751	NA	NA	NA	NA	NA	NA
ILMN_3187680	ACCS	NA	NA	0,00471954	NA	NA	NA	NA	NA	NA
ILMN_1800451	MED16	NA	NA	0,00475609	NA	NA	NA	NA	NA	NA
ILMN_1778662	DIP2A	NA	NA	0,00480151	NA	NA	NA	NA	NA	NA
ILMN_1746025	MINA	NA	NA	0,00480208	NA	NA	NA	NA	NA	NA
ILMN_1654773	CASK	NA	NA	0,00480709	NA	NA	NA	NA	NA	NA
ILMN_1792078	RNF114	NA	NA	0,00481105	NA	NA	NA	NA	NA	NA
ILMN_1793990	ID2	NA	NA	0,0048178	NA	NA	NA	NA	NA	NA
ILMN_1792314	ACTR1A	0,004820096	NA	0,0048201	NA	NA	NA	NA	NA	NA
ILMN_1784292	ANKMY2	NA	NA	0,00486512	NA	NA	NA	NA	NA	NA
ILMN_1746375	CSNK2A1P	NA	0,00487021	0,00487021	NA	NA	NA	NA	NA	NA
ILMN_1772981	EPN1	NA	NA	0,00487052	NA	NA	NA	NA	NA	NA
ILMN_2126957	NOMO1	NA	NA	0,0049116	NA	NA	NA	NA	NA	NA
ILMN_1763365	FAM103A1	NA	NA	0,0049998	NA	NA	NA	NA	NA	NA
ILMN_2394576	FBXW11	NA	NA	0,0050332	NA	NA	NA	NA	NA	NA
ILMN_1694223	DGCR8	NA	NA	0,00503985	NA	NA	NA	NA	NA	NA
ILMN_1691117	DNTTIP1	NA	NA	0,00515136	NA	NA	NA	NA	NA	NA
ILMN_1729180	GATM	0,005172437	NA	0,00517244	NA	NA	NA	NA	NA	NA
ILMN_1703617	AHSA1	NA	NA	0,00517532	NA	NA	NA	NA	NA	NA
ILMN_1687351	ANKRA2	0,00518884	NA	0,00518884	NA	NA	NA	NA	NA	NA
ILMN_1718182	THRA	0,005190828	NA	0,00519083	NA	NA	NA	NA	NA	NA
ILMN_1742705	SLC39A11	0,0052004	NA	0,0052004	NA	NA	NA	NA	NA	NA
ILMN_2225548	ZNF521	NA	NA	0,0052067	NA	NA	NA	NA	NA	NA
ILMN_1759325	C16orf91	NA	NA	0,00521662	NA	NA	NA	NA	NA	NA
ILMN_1708414	GNL3L	NA	NA	0,00524519	NA	NA	NA	NA	NA	NA
ILMN_2379469	EIF3B	0,005280313	NA	0,00528031	NA	NA	NA	NA	NA	NA
ILMN_1718206	KIAA0100	0,005320416	NA	0,00532042	NA	NA	NA	NA	NA	NA
ILMN_2274420	SPTLC1	NA	NA	0,00532997	NA	NA	NA	NA	NA	NA
ILMN_1739397	GLMN	0,005337423	NA	0,00533742	NA	NA	NA	NA	NA	NA
ILMN_2115434	RAB32	NA	NA	0,00535814	NA	NA	NA	NA	NA	NA
ILMN_1656540	RUVBL1	NA	NA	0,00539421	NA	NA	NA	NA	NA	NA
ILMN_1734194	EXOSC3	NA	0,00543464	0,00543464	NA	NA	NA	NA	NA	NA
ILMN_1735367	HSD17B11	NA	NA	0,00550215	NA	NA	NA	NA	NA	NA
ILMN_2344850	VPS26A	NA	NA	0,00552201	NA	NA	NA	NA	NA	NA
ILMN_2399497	RFXANK	NA	NA	0,00553405	NA	NA	NA	NA	NA	NA
ILMN_2056975	HPRT1	NA	NA	0,00554304	NA	NA	NA	NA	NA	NA
ILMN_2394027	CLK3	0,005555547	NA	0,00555555	NA	NA	NA	NA	NA	NA
ILMN_3185144	LOC100128975	0,005633514	0,00563351	0,00563351	NA	NA	NA	NA	NA	NA
ILMN_1744442	TTPAL	0,005666196	NA	0,0056662	NA	NA	NA	NA	NA	NA
ILMN_1733746	REEP1	0,005684248	NA	0,00568425	NA	NA	NA	NA	NA	NA
ILMN_1677843	RAB24	NA	NA	0,00569494	NA	NA	NA	NA	NA	NA
ILMN_2149053	RIF1	NA	NA	0,00570522	NA	NA	NA	NA	NA	NA
ILMN_3246401	AIF1L	0,00573298	NA	0,00573298	NA	NA	NA	NA	NA	NA
ILMN_1725528	LOC400657	NA	NA	0,00575583	NA	NA	NA	NA	NA	NA
ILMN_1761058	ACAD11	NA	NA	0,00582469	NA	NA	NA	NA	NA	NA
ILMN_1695422	NCL	0,005851011	0,00585101	0,00585101	NA	NA	NA	NA	NA	NA
ILMN_1715693	LOC440160	NA	NA	0,00585354	NA	NA	NA	NA	NA	NA
ILMN_1720578	PRAF2	0,005861624	NA	0,00586162	NA	NA	NA	NA	NA	NA
ILMN_2367753	ATP2B4	NA	NA	0,00590331	NA	NA	NA	NA	NA	NA
ILMN_1701434	RAP1B	NA	NA	0,00592621	NA	NA	NA	NA	NA	NA
ILMN_1780773	LOC400027	0,005928482	NA	0,00592848	NA	NA	NA	NA	NA	NA
ILMN_1797875	ALOX5AP	NA	NA	0,00595275	NA	NA	NA	NA	NA	NA
ILMN_3234997	MAP7D1	NA	NA	0,00598124	NA	NA	NA	NA	NA	NA
ILMN_1668283	HYAL2	NA	NA	0,00598902	NA	NA	NA	NA	NA	NA
ILMN_2053829	CBLN3	0,006016257	NA	0,00601626	NA	NA	NA	NA	NA	NA
ILMN_1683277	KIAA0319L	0,006024552	NA	0,00602455	NA	NA	NA	NA	NA	NA
ILMN_2385647	ALAS1	0,006043471	NA	0,00604347	NA	NA	NA	NA	NA	NA
ILMN_2113938	TOR1AIP2	NA	NA	0,00605052	NA	NA	NA	NA	NA	NA
ILMN_1708743	NT5DC2	NA	NA	0,00605365	NA	NA	NA	NA	NA	NA
ILMN_2054607	CYP4V2	NA	NA	0,00606127	NA	NA	NA	NA	NA	NA
ILMN_1704793	MYPOP	NA	NA	0,00609849	NA	NA	NA	NA	NA	NA
ILMN_1676611	PHPT1	NA	NA	0,00610915	NA	NA	NA	NA	NA	NA
ILMN_1714438	MUTYH	NA	NA	0,00613277	NA	NA	NA	NA	NA	NA
ILMN_1815043	MRPS2	NA	NA	0,00613702	NA	NA	NA	NA	NA	NA
ILMN_3241469	LOC100134537	NA	NA	0,00616564	NA	NA	NA	NA	NA	NA
ILMN_1713901	KDELR3	NA	NA	0,00616669	NA	NA	NA	NA	NA	NA
ILMN_2394557	WNK3	0,006221958	NA	0,00622196	NA	NA	NA	NA	NA	NA
ILMN_1785785	NCOA1	NA	NA	0,00622534	NA	NA	NA	NA	NA	NA
ILMN_2117223	ROD1	0,006253088	NA	0,00625309	NA	NA	NA	NA	NA	NA
ILMN_2224290	ZNF322B	0,006258072	NA	0,00625807	NA	NA	NA	NA	NA	NA
ILMN_1791328	STK39	0,006266653	NA	0,00626665	NA	NA	NA	NA	NA	NA
ILMN_1721921	BLMH	NA	NA	0,00630123	NA	NA	NA	NA	NA	NA
ILMN_1676289	NCAM1	NA	NA	0,00630401	NA	NA	NA	NA	NA	NA
ILMN_1792389	RNF165	NA	NA	0,00630546	NA	NA	NA	NA	NA	NA

ILMN_1694106	GPD1L	NA	NA	0,00632697	NA	NA	NA	NA	NA	NA
ILMN_1651262	HNRNPAB	0,006327564	NA	0,00632756	NA	NA	NA	NA	NA	NA
ILMN_1762990	KIAA0141	0,006328198	NA	0,0063282	NA	NA	NA	NA	NA	NA
ILMN_1658460	LOC653884	NA	NA	0,00635501	NA	NA	NA	NA	NA	NA
ILMN_1796113	BCDIN3D	NA	NA	0,00636375	NA	NA	NA	NA	NA	NA
ILMN_1706610	PLEKHA9	NA	NA	0,00639983	NA	NA	NA	NA	NA	NA
ILMN_3251404	NUCKS1	NA	NA	0,00645262	NA	NA	NA	NA	NA	NA
ILMN_1658486	MRPL54	NA	NA	0,00650267	NA	NA	NA	NA	NA	NA
ILMN_1742230	BAZ1A	0,006577316	NA	0,00657732	NA	NA	NA	NA	NA	NA
ILMN_1782685	LOC652672	NA	NA	0,00658301	NA	NA	NA	NA	NA	NA
ILMN_1691393	DNPEP	NA	NA	0,00659886	NA	NA	NA	NA	NA	NA
ILMN_1655561	ARPC3	NA	NA	0,00661255	NA	NA	NA	NA	NA	NA
ILMN_1671791	PCK2	NA	NA	0,00661824	NA	NA	NA	NA	NA	NA
ILMN_1807972	MICAL1	NA	NA	0,0066249	NA	NA	NA	NA	NA	NA
ILMN_1663033	TMEM129	NA	NA	0,006626	NA	NA	NA	NA	NA	NA
ILMN_2045911	FBXO28	NA	NA	0,00664378	NA	NA	NA	NA	NA	NA
ILMN_1794333	POU2F1	NA	NA	0,00665511	NA	NA	NA	NA	NA	NA
ILMN_3251742	ZNF322A	NA	NA	0,00666236	NA	NA	NA	NA	NA	NA
ILMN_1777106	LRRC57	0,006662556	NA	0,00666256	NA	NA	NA	NA	NA	NA
ILMN_2223380	PPWD1	NA	NA	0,00672432	NA	NA	NA	NA	NA	NA
ILMN_2082893	LOC440157	NA	NA	0,00673055	NA	NA	NA	NA	NA	NA
ILMN_2098446	PMAIP1	0,006747508	NA	0,00674751	NA	NA	NA	NA	NA	NA
ILMN_3307887	C22orf30	0,006776483	NA	0,00677648	NA	NA	NA	NA	NA	NA
ILMN_1773620	SMARCC2	NA	NA	0,00680113	NA	NA	NA	NA	NA	NA
ILMN_2324998	NGDN	NA	0,00680418	0,00680418	NA	NA	NA	NA	NA	NA
ILMN_1669502	E2F3	NA	NA	0,00680884	NA	NA	NA	NA	NA	NA
ILMN_1807031	C14orf28	NA	NA	0,00684269	NA	NA	NA	NA	NA	NA
ILMN_1734826	NUP88	0,006847101	NA	0,0068471	NA	NA	NA	NA	NA	NA
ILMN_2311278	ADD3	NA	0,0068981	0,0068981	NA	NA	NA	NA	NA	NA
ILMN_1732537	HEATR2	NA	NA	0,00691073	NA	NA	NA	NA	NA	NA
ILMN_1810797	WASF3	NA	0,00691592	0,00691592	NA	NA	NA	NA	NA	NA
ILMN_1738921	ACAA1	NA	NA	0,0069813	NA	NA	NA	NA	NA	NA
ILMN_1676528	BTN3A2	NA	0,00698206	0,00698206	NA	NA	NA	NA	NA	NA
ILMN_1738554	LOC647346	NA	NA	0,00698965	NA	NA	NA	NA	NA	NA
ILMN_1723467	ITGB1	NA	NA	0,00699325	NA	NA	NA	NA	NA	NA
ILMN_1718129	MAP2K5	NA	NA	0,00701596	NA	NA	NA	NA	NA	NA
ILMN_1746012	MBD6	NA	NA	0,00703146	NA	NA	NA	NA	NA	NA
ILMN_1807873	SNX6	NA	NA	0,00705464	NA	NA	NA	NA	NA	NA
ILMN_1755822	SYDE1	NA	NA	0,00705766	NA	NA	NA	NA	NA	NA
ILMN_1756767	EIF5B	0,007075536	NA	0,00707554	NA	NA	NA	NA	NA	NA
ILMN_1802397	GNA11	NA	NA	0,00709477	NA	NA	NA	NA	NA	NA
ILMN_1694759	C19orf42	NA	NA	0,00712656	NA	NA	NA	NA	NA	NA
ILMN_1811468	IRX3	NA	NA	0,0071373	NA	NA	NA	NA	NA	NA
ILMN_1799082	ZNF609	0,00717286	NA	0,00717286	NA	NA	NA	NA	NA	NA
ILMN_1787844	C19orf10	NA	NA	0,00717622	NA	NA	NA	NA	NA	NA
ILMN_1820244		NA	NA	0,00718455	NA	NA	NA	NA	NA	NA
ILMN_1795285	PHF15	NA	NA	0,00725281	NA	NA	NA	NA	NA	NA
ILMN_1797804	BACE1	NA	NA	0,00728216	NA	NA	NA	NA	NA	NA
ILMN_1681741	C1orf31	NA	NA	0,00728249	NA	NA	NA	NA	NA	NA
ILMN_2134110	SELK	NA	NA	0,00728894	NA	NA	NA	NA	NA	NA
ILMN_1809173	LOC729021	NA	0,00732638	0,00732638	NA	NA	NA	NA	NA	NA
ILMN_3208014	LOC100131866	NA	NA	0,00732762	NA	NA	NA	NA	NA	NA
ILMN_3245869	LOC440957	NA	NA	0,00734498	NA	NA	NA	NA	NA	NA
ILMN_1673352	IFITM2	NA	NA	0,00737573	NA	NA	NA	NA	NA	NA
ILMN_1780188	B3GALNT2	NA	NA	0,00738323	NA	NA	NA	NA	NA	NA
ILMN_1752285	RPL4	NA	NA	0,00740776	NA	NA	NA	NA	NA	NA
ILMN_2047240	ARSA	NA	NA	0,00742924	NA	NA	NA	NA	NA	NA
ILMN_1762582	ARNT	NA	NA	0,00749966	NA	NA	NA	NA	NA	NA
ILMN_1742798	SFRS10	0,007530617	0,00753062	0,00753062	NA	NA	NA	NA	NA	NA
ILMN_1654488	UTX	0,0075401	NA	0,0075401	NA	NA	NA	NA	NA	NA
ILMN_1771026	GARS	NA	NA	0,00754838	NA	NA	NA	NA	NA	NA
ILMN_1784036	CDH15	NA	NA	0,00758972	NA	NA	NA	NA	NA	NA
ILMN_1788024	PCID2	0,007631209	NA	0,00763121	NA	NA	NA	NA	NA	NA
ILMN_1654497	ATAD1	NA	NA	0,00764539	NA	NA	NA	NA	NA	NA
ILMN_1779076	HINT3	NA	NA	0,00768344	NA	NA	NA	NA	NA	NA
ILMN_2379788	HIF1A	NA	NA	0,00769696	NA	NA	NA	NA	NA	NA
ILMN_1741224	GPR137C	0,007698003	NA	0,007698	NA	NA	NA	NA	NA	NA
ILMN_1667839	UBR7	0,007721609	NA	0,00772161	NA	NA	NA	NA	NA	NA
ILMN_1705114	NUMB	NA	NA	0,00773362	NA	NA	NA	NA	NA	NA
ILMN_1747058	TRAPPC2L	NA	0,00775498	0,00775498	NA	NA	NA	NA	NA	NA
ILMN_1897310		NA	NA	0,00776788	NA	NA	NA	NA	NA	NA
ILMN_1705153	NEFH	0,00778907	0,00778907	0,00778907	NA	NA	NA	NA	NA	NA
ILMN_1778078	VPS16	NA	0,00779052	0,00779052	NA	NA	NA	NA	NA	NA
ILMN_1707137	C17orf97	0,007804952	NA	0,00780495	NA	NA	NA	NA	NA	NA

ILMN_3238106	FAM161A	0,007851344	NA	0,00785134	NA	NA	NA	NA	NA	NA
ILMN_3292320	LOC642513	NA	NA	0,00786434	NA	NA	NA	NA	NA	NA
ILMN_1695719	EIF2C2	NA	NA	0,00789151	NA	NA	NA	NA	NA	NA
ILMN_3290380	LOC387703	NA	NA	0,00789538	NA	NA	NA	NA	NA	NA
ILMN_2101719	C10orf84	0,007897251	0,00789725	0,00789725	NA	NA	NA	NA	NA	NA
ILMN_1738656	GLOD4	0,007952011	0,00795201	0,00795201	NA	NA	NA	NA	NA	NA
ILMN_1658759	PEX19	NA	NA	0,00795262	NA	NA	NA	NA	NA	NA
ILMN_1654639	HERC6	0,00798196	NA	0,00798196	NA	NA	NA	NA	NA	NA
ILMN_1699772	RRAGD	NA	NA	0,00799142	NA	NA	NA	NA	NA	NA
ILMN_1667374	C14orf149	NA	0,00800084	0,00800084	NA	NA	NA	NA	NA	NA
ILMN_1847822	KIAA0368	NA	NA	0,00809015	NA	NA	NA	NA	NA	NA
ILMN_1734410	BNIP1	0,008147744	0,00814774	0,00814774	NA	NA	NA	NA	NA	NA
ILMN_2136455	C3orf64	NA	NA	0,00819345	NA	NA	NA	NA	NA	NA
ILMN_1810225	KIAA1530	0,008213602	NA	0,0082136	NA	NA	NA	NA	NA	NA
ILMN_1661264	SHMT2	0,008226802	NA	0,0082268	NA	NA	NA	NA	NA	NA
ILMN_2366790	DDX52	NA	NA	0,00823606	NA	NA	NA	NA	NA	NA
ILMN_1662340	ZNF358	NA	NA	0,00829057	NA	NA	NA	NA	NA	NA
ILMN_2063168	MALL	NA	NA	0,00832076	NA	NA	NA	NA	NA	NA
ILMN_1815320	NR5A1	0,008339258	NA	0,00833926	NA	NA	NA	NA	NA	NA
ILMN_1667510	C12orf65	NA	NA	0,00835045	NA	NA	NA	NA	NA	NA
ILMN_1774074	RXRB	NA	NA	0,0083696	NA	NA	NA	NA	NA	NA
ILMN_2402817	ZBTB16	NA	NA	0,00840654	NA	NA	NA	NA	NA	NA
ILMN_1659189	C9orf89	NA	NA	0,0084109	NA	NA	NA	NA	NA	NA
ILMN_2223922	AGTPBP1	NA	NA	0,00842605	NA	NA	NA	NA	NA	NA
ILMN_1677534	SCAP	NA	NA	0,00845899	NA	NA	NA	NA	NA	NA
ILMN_1906187		NA	NA	0,00848014	NA	NA	NA	NA	NA	NA
ILMN_1655876	TMEM159	NA	NA	0,00848606	NA	NA	NA	NA	NA	NA
ILMN_1718718	MKKS	NA	NA	0,0084914	NA	NA	NA	NA	NA	NA
ILMN_1737738	NDUFA12	NA	NA	0,00850702	NA	NA	NA	NA	NA	NA
ILMN_1695386	RAD51C	NA	NA	0,00853116	NA	NA	NA	NA	NA	NA
ILMN_3236259	PPIAL4A	NA	NA	0,0085844	NA	NA	NA	NA	NA	NA
ILMN_1661439	FLOT1	NA	NA	0,00861641	NA	NA	NA	NA	NA	NA
ILMN_1714216	TSC2	NA	0,00861953	0,00861953	NA	NA	NA	NA	NA	NA
ILMN_1784207	C1orf128	NA	NA	0,0086206	NA	NA	NA	NA	NA	NA
ILMN_1811264	C15orf57	NA	NA	0,00862534	NA	NA	NA	NA	NA	NA
ILMN_1811110	TDRD9	NA	NA	0,00862934	NA	NA	NA	NA	NA	NA
ILMN_3192791	HNRNPM	NA	NA	0,00863953	NA	NA	NA	NA	NA	NA
ILMN_1763310	LOC654260	NA	NA	0,00866417	NA	NA	NA	NA	NA	NA
ILMN_1712678	RPS27L	NA	NA	0,00874033	NA	NA	NA	NA	NA	NA
ILMN_1756408	PARVA	NA	0,00874579	0,00874579	NA	NA	NA	NA	NA	NA
ILMN_3277872	LOC644684	NA	NA	0,00875677	NA	NA	NA	NA	NA	NA
ILMN_1693108	RUVBL1	NA	NA	0,00877298	NA	NA	NA	NA	NA	NA
ILMN_2097546	NUDC	NA	NA	0,00879307	NA	NA	NA	NA	NA	NA
ILMN_2151739	CAT	NA	NA	0,00883794	NA	NA	NA	NA	NA	NA
ILMN_1739236	ZNF668	0,008868324	NA	0,00886832	NA	NA	NA	NA	NA	NA
ILMN_1805992	KIAA1598	NA	NA	0,00888827	NA	NA	NA	NA	NA	NA
ILMN_2380237	C1QTNF1	NA	NA	0,00893713	NA	NA	NA	NA	NA	NA
ILMN_1653712	UAP1L1	NA	NA	0,00894495	NA	NA	NA	NA	NA	NA
ILMN_1728426	INPPL1	NA	NA	0,00896941	NA	NA	NA	NA	NA	NA
ILMN_3245907	FAM160B2	NA	NA	0,00898416	NA	NA	NA	NA	NA	NA
ILMN_2365465	XBP1	NA	NA	0,00900621	NA	NA	NA	NA	NA	NA
ILMN_1651336	MLYCD	NA	NA	0,00900839	NA	NA	NA	NA	NA	NA
ILMN_1715789	DOCK1	NA	NA	0,00901946	NA	NA	NA	NA	NA	NA
ILMN_3239621	SNRNP27	NA	NA	0,00903256	NA	NA	NA	NA	NA	NA
ILMN_1673773	GALNTL4	NA	NA	0,00903262	NA	NA	NA	NA	NA	NA
ILMN_2367239	RCAN1	NA	NA	0,00904288	NA	NA	NA	NA	NA	NA
ILMN_2288232	PHKB	NA	NA	0,00905432	NA	NA	NA	NA	NA	NA
ILMN_1790797	VPS28	NA	NA	0,00905455	NA	NA	NA	NA	NA	NA
ILMN_1861057		NA	NA	0,00916331	NA	NA	NA	NA	NA	NA
ILMN_2301725	PDE8B	NA	NA	0,00922047	NA	NA	NA	NA	NA	NA
ILMN_3307025	ZDHHC4	0,009239895	NA	0,0092399	NA	NA	NA	NA	NA	NA
ILMN_1685012	EAF1	NA	NA	0,00924403	NA	NA	NA	NA	NA	NA
ILMN_1802151	OSBPL5	NA	NA	0,00925232	NA	NA	NA	NA	NA	NA
ILMN_1796749	KIF1C	NA	NA	0,00927251	NA	NA	NA	NA	NA	NA
ILMN_1670472	FAF2	NA	NA	0,00932115	NA	NA	NA	NA	NA	NA
ILMN_1683950	SNX11	NA	NA	0,00932352	NA	NA	NA	NA	NA	NA
ILMN_2408430	LARGE	0,009386842	0,00938684	0,00938684	NA	NA	NA	NA	NA	NA
ILMN_1900263		NA	NA	0,00943059	NA	NA	NA	NA	NA	NA
ILMN_1766505	COMMD10	NA	NA	0,00943222	NA	NA	NA	NA	NA	NA
ILMN_1723116	AMFR	NA	NA	0,00943527	NA	NA	NA	NA	NA	NA
ILMN_1672503	DPYSL2	NA	NA	0,00946331	NA	NA	NA	NA	NA	NA
ILMN_2367020	SEC61G	NA	NA	0,00952704	NA	NA	NA	NA	NA	NA
ILMN_3239946	LOC729375	NA	NA	0,00960261	NA	NA	NA	NA	NA	NA
ILMN_2399877	COG5	NA	NA	0,00961239	NA	NA	NA	NA	NA	NA

ILMN_3244286	TANC2	0,009671795	NA	0,0096718	NA	NA	NA	NA	NA	NA
ILMN_2278729	EIF3B	0,009683822	NA	0,00968382	NA	NA	NA	NA	NA	NA
ILMN_1667018	ACE2	NA	NA	0,00969404	NA	NA	NA	NA	NA	NA
ILMN_1703316	LOC255783	0,009700157	NA	0,00970016	NA	NA	NA	NA	NA	NA
ILMN_1687978	PHLDA1	NA	NA	0,00972592	NA	NA	NA	NA	NA	NA
ILMN_1762825	CANX	NA	NA	0,00974055	NA	NA	NA	NA	NA	NA
ILMN_1718013	CPSF3L	NA	NA	0,00974383	NA	NA	NA	NA	NA	NA
ILMN_2304624	EIF4H	0,009767654	NA	0,00976765	NA	NA	NA	NA	NA	NA
ILMN_1787815	TRIB3	NA	NA	0,00977907	NA	NA	NA	NA	NA	NA
ILMN_2298936	THOC3	NA	NA	0,00979265	NA	NA	NA	NA	NA	NA
ILMN_1729142	CENPV	NA	NA	0,0097957	NA	NA	NA	NA	NA	NA
ILMN_1743340	CHST14	NA	NA	0,00980264	NA	NA	NA	NA	NA	NA
ILMN_2354269	FAM164C	NA	0,00983044	0,00983044	NA	NA	NA	NA	NA	NA
ILMN_1760245	TMEM42	NA	NA	0,00987507	NA	NA	NA	NA	NA	NA
ILMN_3246209	SCARNA10	NA	NA	0,00989672	NA	NA	NA	NA	NA	NA
ILMN_2340052	NCOR2	NA	NA	0,00991607	NA	NA	NA	NA	NA	NA
ILMN_1710482	APLP2	0,009984841	0,00998484	0,00998484	NA	NA	NA	NA	NA	NA
ILMN_2363231	SMAP1	NA	0,01051849	NA	NA	NA	NA	NA	NA	NA
ILMN_1800889	FIG4	NA	0,01067714	NA	NA	NA	NA	NA	NA	NA
ILMN_2389273	FXR1	NA	0,01068399	NA	NA	NA	NA	NA	NA	NA
ILMN_2067708	TFB2M	NA	0,01138821	NA	NA	NA	NA	NA	NA	NA
ILMN_1735679	DHX38	NA	0,01246517	NA	NA	NA	NA	NA	NA	NA
ILMN_1846922		NA	0,01265459	NA	NA	NA	NA	NA	NA	NA
ILMN_3305849	LOC728431	0,013075228	0,01307523	NA	NA	NA	NA	NA	NA	NA
ILMN_1740976	NONO	NA	0,0142826	NA	NA	NA	NA	NA	NA	NA
ILMN_1772946	STRN3	0,014585647	0,01458565	NA	NA	NA	NA	NA	NA	NA
ILMN_1712405	LOC642468	0,015007101	0,0150071	NA	NA	NA	NA	NA	NA	NA
ILMN_1679520	AGPAT1	0,01551367	0,01551367	NA	NA	NA	NA	NA	NA	NA
ILMN_2209748	DERL1	NA	0,01673367	NA	NA	NA	NA	NA	NA	NA
ILMN_1718069	MIS12	NA	0,01784587	NA	NA	NA	NA	NA	NA	NA
ILMN_2349129	DPP3	0,018083053	0,01808305	NA	NA	NA	NA	NA	NA	NA
ILMN_2351309	TIAL1	NA	0,01822701	NA	NA	NA	NA	NA	NA	NA
ILMN_1686135	CCDC45	NA	0,01861244	NA	NA	NA	NA	NA	NA	NA
ILMN_1751956	MGST3	NA	0,0191281	NA	NA	NA	NA	NA	NA	NA
ILMN_3224856	LOC729660	0,019348913	0,01934891	NA	NA	NA	NA	NA	NA	NA
ILMN_1749009	REXO2	NA	0,02003672	NA	NA	NA	NA	NA	NA	NA
ILMN_1716728	C6orf64	NA	0,0203339	NA	NA	NA	NA	NA	NA	NA
ILMN_3208330	LOC100132797	NA	0,02064712	NA	NA	NA	NA	NA	NA	NA
ILMN_1665107	ITGB1BP1	0,02176041	0,02176041	NA	NA	NA	NA	NA	NA	NA
ILMN_2356955	PLAGL1	NA	0,02269126	NA	NA	NA	NA	NA	NA	NA
ILMN_1687392	NRK	0,022907833	0,02290783	NA	NA	NA	NA	NA	NA	NA
ILMN_1658425	DAG1	0,024543258	0,02454326	NA	NA	NA	NA	NA	NA	NA
ILMN_1678546	PEX11B	NA	0,02470404	NA	NA	NA	NA	NA	NA	NA
ILMN_2053281	C14orf149	0,028627659	0,02862766	NA	NA	NA	NA	NA	NA	NA
ILMN_1781966	OSBP2	0,029454439	0,02945444	NA	NA	NA	NA	NA	NA	NA
ILMN_1758895	CTSK	0,031164193	0,03116419	NA	NA	NA	NA	NA	NA	NA
ILMN_1793829	TMCO1	NA	0,03137474	NA	NA	NA	NA	NA	NA	NA
ILMN_1801852	SEC61B	NA	0,03138628	NA	NA	NA	NA	NA	NA	NA
ILMN_1904980		NA	0,03172198	NA	NA	NA	NA	NA	NA	NA
ILMN_1789616	NUPL2	0,032530148	0,03253015	NA	NA	NA	NA	NA	NA	NA
ILMN_1805449	TAPBPL	0,034388393	0,03438839	NA	NA	NA	NA	NA	NA	NA
ILMN_1697777	TSTA3	NA	0,03469784	NA	NA	NA	NA	NA	NA	NA
ILMN_1747204	HTRA2	0,036687765	0,03668777	NA	NA	NA	NA	NA	NA	NA
ILMN_1786189	MKI67IP	NA	0,03672806	NA	NA	NA	NA	NA	NA	NA
ILMN_1686392	NBAS	NA	0,03721244	NA	NA	NA	NA	NA	NA	NA
ILMN_2310968	RUFY1	0,037436297	0,0374363	NA	NA	NA	NA	NA	NA	NA
ILMN_1814044	CABIN1	0,03798449	0,03798449	NA	NA	NA	NA	NA	NA	NA
ILMN_1663113	TTLL12	NA	0,03799067	NA	NA	NA	NA	NA	NA	NA
ILMN_1701229	RBM12	NA	0,03875531	NA	NA	NA	NA	NA	NA	NA
ILMN_1804642	SMUG1	NA	0,03887069	NA	NA	NA	NA	NA	NA	NA
ILMN_1704753	EPAS1	0,041252178	0,04125218	NA	NA	NA	NA	NA	NA	NA
ILMN_2351241	RNF14	0,041783141	0,04178314	NA	NA	NA	NA	NA	NA	NA
ILMN_2182120	SF3B14	NA	0,04184376	NA	NA	NA	NA	NA	NA	NA
ILMN_2375386	RNPS1	NA	0,04334019	NA	NA	NA	NA	NA	NA	NA
ILMN_1832252		NA	0,04428953	NA	NA	NA	NA	NA	NA	NA
ILMN_2328986	SREBF1	NA	0,04632272	NA	NA	NA	NA	NA	NA	NA
ILMN_1726906	AKT1S1	NA	0,0467741	NA	NA	NA	NA	NA	NA	NA
ILMN_1669718	PSENEN	0,047087092	0,04708709	NA	NA	NA	NA	NA	NA	NA
ILMN_1667707	SPCS3	NA	0,04979964	NA	NA	NA	NA	NA	NA	NA
ILMN_2096604	NIP30	0,053804693	0,05380469	NA	NA	NA	NA	NA	NA	NA
ILMN_1765520	MTIF2	NA	0,05581895	NA	NA	NA	NA	NA	NA	NA
ILMN_1701131	C2orf49	NA	0,05745762	NA	NA	NA	NA	NA	NA	NA
ILMN_2314208	S100PBP	0,057861649	0,05786165	NA	NA	NA	NA	NA	NA	NA
ILMN_1670925	CYB5D1	NA	0,05806498	NA	NA	NA	NA	NA	NA	NA

ILMN_3236156	OSTC	NA	0,06030702	NA	NA	NA	NA	NA	NA	NA
ILMN_1801842	PTX3	NA	0,06871543	NA	NA	NA	NA	NA	NA	NA
ILMN_1690695	PEX11A	NA	0,07023766	NA	NA	NA	NA	NA	NA	NA
ILMN_1739154	LSAMP	0,073547155	0,07354716	NA	NA	NA	NA	NA	NA	NA
ILMN_1742074	C8orf76	NA	0,07416045	NA	NA	NA	NA	NA	NA	NA
ILMN_1736054	SUB1	NA	0,08027505	NA	NA	NA	NA	NA	NA	NA
ILMN_2344002	SIP1	NA	0,08283677	NA	NA	NA	NA	NA	NA	NA
ILMN_1903021		NA	0,08306686	NA	NA	NA	NA	NA	NA	NA
ILMN_1679800	BRIX1	NA	0,08661115	NA	NA	NA	NA	NA	NA	NA
ILMN_1679917	ARL16	0,087261966	0,08726197	NA	NA	NA	NA	NA	NA	NA
ILMN_1796063	TRIM44	NA	0,08749837	NA	NA	NA	NA	NA	NA	NA
ILMN_2213558	TMED10P	NA	0,09391619	NA	NA	NA	NA	NA	NA	NA
ILMN_1783996	DEM1	NA	0,09660473	NA	NA	NA	NA	NA	NA	NA
ILMN_1714756	YIPF5	NA	0,0995053	NA	NA	NA	NA	NA	NA	NA
ILMN_1812669	HTRA3	0,100716212	0,10071621	NA	NA	NA	NA	NA	NA	NA
ILMN_1707506	YTHDC1	NA	0,10084875	NA	NA	NA	NA	NA	NA	NA
ILMN_1677043	AKR7A2	NA	0,1011213	NA	NA	NA	NA	NA	NA	NA
ILMN_1777318	C9orf64	NA	0,1021553	NA	NA	NA	NA	NA	NA	NA
ILMN_2124386	RGL2	NA	0,10362389	NA	NA	NA	NA	NA	NA	NA
ILMN_1722034	KIAA1586	NA	0,10424415	NA	NA	NA	NA	NA	NA	NA
ILMN_1668743	LOC728069	NA	0,1042836	NA	NA	NA	NA	NA	NA	NA
ILMN_1787885	NUDT18	NA	0,10860159	NA	NA	NA	NA	NA	NA	NA
ILMN_1659777	RUFY1	NA	0,10938698	NA	NA	NA	NA	NA	NA	NA
ILMN_2359014	TBCE	0,11404511	0,11404511	NA	NA	NA	NA	NA	NA	NA
ILMN_1804798	BEXL1	0,116000585	0,11600059	NA	NA	NA	NA	NA	NA	NA
ILMN_1678494	ZNF438	0,116793665	0,11679367	NA	NA	NA	NA	NA	NA	NA
ILMN_1735959	CNOT4	NA	0,11860257	NA	NA	NA	NA	NA	NA	NA
ILMN_1750178	LOC653450	NA	0,12272838	NA	NA	NA	NA	NA	NA	NA
ILMN_1719471	MSH3	NA	0,12569208	NA	NA	NA	NA	NA	NA	NA
ILMN_1759341	MAN2B1	NA	0,13377512	NA	NA	NA	NA	NA	NA	NA
ILMN_1814247	TCFL5	NA	0,13614529	NA	NA	NA	NA	NA	NA	NA
ILMN_1655557	INTS6	NA	0,13698137	NA	NA	NA	NA	NA	NA	NA
ILMN_1651278	SNIP1	NA	0,13999671	NA	NA	NA	NA	NA	NA	NA
ILMN_1792110	C10orf76	NA	0,140795	NA	NA	NA	NA	NA	NA	NA
ILMN_1735353	IRX1	NA	0,1418751	NA	NA	NA	NA	NA	NA	NA
ILMN_1789349	UBQLN4	NA	0,14502218	NA	NA	NA	NA	NA	NA	NA
ILMN_1710598	SKI	NA	0,14565244	NA	NA	NA	NA	NA	NA	NA
ILMN_1689431	APCDD1L	NA	0,14855878	NA	NA	NA	NA	NA	NA	NA
ILMN_2395926	MANBAL	NA	0,1490485	NA	NA	NA	NA	NA	NA	NA
ILMN_1763989	SEC63	NA	0,15549914	NA	NA	NA	NA	NA	NA	NA
ILMN_1678934	POLR1E	0,168581089	0,16858109	NA	NA	NA	NA	NA	NA	NA
ILMN_1737964	HIATL1	NA	0,18009368	NA	NA	NA	NA	NA	NA	NA
ILMN_1782095	C20orf55	NA	0,19017185	NA	NA	NA	NA	NA	NA	NA
ILMN_1773485	QKI	0,190768501	0,1907685	NA	NA	NA	NA	NA	NA	NA
ILMN_2351638	BEX4	0,192430636	0,19243064	NA	NA	NA	NA	NA	NA	NA
ILMN_2318733	BNIP1	NA	0,19531342	NA	NA	NA	NA	NA	NA	NA
ILMN_2117885	FAM122A	NA	0,20203477	NA	NA	NA	NA	NA	NA	NA
ILMN_1730698	ODF2	NA	0,20277676	NA	NA	NA	NA	NA	NA	NA
ILMN_1667476	LTBR	NA	0,20587512	NA	NA	NA	NA	NA	NA	NA
ILMN_3279960	LOC642784	NA	0,20930524	NA	NA	NA	NA	NA	NA	NA
ILMN_1714461	RNF14	0,213439029	0,21343903	NA	NA	NA	NA	NA	NA	NA
ILMN_1719449	DCLK2	NA	0,21486157	NA	NA	NA	NA	NA	NA	NA
ILMN_1685112	TACO1	NA	0,21493376	NA	NA	NA	NA	NA	NA	NA
ILMN_1735151	EIF5A2	NA	0,22327777	NA	NA	NA	NA	NA	NA	NA
ILMN_1753143	RHPN2	NA	0,23074159	NA	NA	NA	NA	NA	NA	NA
ILMN_1712400	SERPINB6	NA	0,2309997	NA	NA	NA	NA	NA	NA	NA
ILMN_1667657	TPP2	0,235061895	0,2350619	NA	NA	NA	NA	NA	NA	NA
ILMN_1734231	DDOST	0,250646995	0,250647	NA	NA	NA	NA	NA	NA	NA
ILMN_1723156	PTPRA	NA	0,25232779	NA	NA	NA	NA	NA	NA	NA
ILMN_2277099	YWHAB	NA	0,25434816	NA	NA	NA	NA	NA	NA	NA
ILMN_1666376	TRIM56	NA	0,26198391	NA	NA	NA	NA	NA	NA	NA
ILMN_1784540	KBTBD2	NA	0,26559896	NA	NA	NA	NA	NA	NA	NA
ILMN_1682015	GAL	NA	0,26916154	NA	NA	NA	NA	NA	NA	NA
ILMN_1671351	MAP3K7IP3	NA	0,26975652	NA	NA	NA	NA	NA	NA	NA
ILMN_1723743	ROM1	NA	0,27044912	NA	NA	NA	NA	NA	NA	NA
ILMN_2297710	PLEKHB2	NA	0,27879702	NA	NA	NA	NA	NA	NA	NA
ILMN_2375484	CPEB2	NA	0,29201101	NA	NA	NA	NA	NA	NA	NA
ILMN_2263054	FEZ1	NA	0,29413765	NA	NA	NA	NA	NA	NA	NA
ILMN_1784554	LOC647389	NA	0,32020881	NA	NA	NA	NA	NA	NA	NA
ILMN_1754570	KCTD8	NA	0,32506063	NA	NA	NA	NA	NA	NA	NA
ILMN_1665428	GSDMD	NA	0,33478618	NA	NA	NA	NA	NA	NA	NA
ILMN_1772702	SFRS2B	NA	0,36941864	NA	NA	NA	NA	NA	NA	NA
ILMN_1746396	CTNNB1	NA	0,37735957	NA	NA	NA	NA	NA	NA	NA
ILMN_2355042	CLUAP1	NA	0,38326553	NA	NA	NA	NA	NA	NA	NA

ILMN_3239388	LOC100133950	NA	0,38681547	NA	NA	NA	NA	NA	NA	NA
ILMN_1678831	GCC2	NA	0,38953088	NA	NA	NA	NA	NA	NA	NA
ILMN_1747184	PUS7L	NA	0,39116828	NA	NA	NA	NA	NA	NA	NA
ILMN_1737818	C12orf43	NA	0,3973315	NA	NA	NA	NA	NA	NA	NA
ILMN_3251155	PCBP2	0,402529172	0,40252917	NA	NA	NA	NA	NA	NA	NA
ILMN_1706859	C22orf32	NA	0,4074731	NA	NA	NA	NA	NA	NA	NA
ILMN_1674367	SPRR2F	0,437720336	0,43772034	NA	NA	NA	NA	NA	NA	NA
ILMN_1655734	RPF1	NA	0,4462112	NA	NA	NA	NA	NA	NA	NA
ILMN_1907705		NA	0,45641218	NA	NA	NA	NA	NA	NA	NA
ILMN_1826531		NA	0,46507814	NA	NA	NA	NA	NA	NA	NA
ILMN_2393712	CTTN	NA	0,46876127	NA	NA	NA	NA	NA	NA	NA
ILMN_1808566	TMEM180	NA	0,48228209	NA	NA	NA	NA	NA	NA	NA
ILMN_3308663	MIR1228	NA	0,49719977	NA	NA	NA	NA	NA	NA	NA
ILMN_1811754	NDUFB10	NA	0,50184363	NA	NA	NA	NA	NA	NA	NA
ILMN_1744912	CTTN	NA	0,50993264	NA	NA	NA	NA	NA	NA	NA
ILMN_1704291	LOC645317	NA	0,53873567	NA	NA	NA	NA	NA	NA	NA
ILMN_1759766	CTXN1	NA	0,55997592	NA	NA	NA	NA	NA	NA	NA
ILMN_1707337	MSTO1	NA	0,57996991	NA	NA	NA	NA	NA	NA	NA
ILMN_1701466	PEX16	NA	0,59566186	NA	NA	NA	NA	NA	NA	NA
ILMN_1712708	TRIM47	NA	0,59636711	NA	NA	NA	NA	NA	NA	NA
ILMN_2360184	INVS	NA	0,6286607	NA	NA	NA	NA	NA	NA	NA
ILMN_1700584	IER2	NA	0,64034502	NA	NA	NA	NA	NA	NA	NA
ILMN_1690863	ZNF470	NA	0,64478906	NA	NA	NA	NA	NA	NA	NA
ILMN_2414826	VPS13A	NA	0,64542125	NA	NA	NA	NA	NA	NA	NA
ILMN_1792885	CTSC	NA	0,6628969	NA	NA	NA	NA	NA	NA	NA
ILMN_1755882	C13orf7	NA	0,69470649	NA	NA	NA	NA	NA	NA	NA
ILMN_1863939		NA	0,72479032	NA	NA	NA	NA	NA	NA	NA
ILMN_1849941		NA	0,72897712	NA	NA	NA	NA	NA	NA	NA
ILMN_1679150	LOC387647	NA	0,73361495	NA	NA	NA	NA	NA	NA	NA
ILMN_1719517	CTTNBP2NL	NA	0,75504573	NA	NA	NA	NA	NA	NA	NA
ILMN_2251184	ERP29	NA	0,75962248	NA	NA	NA	NA	NA	NA	NA
ILMN_1803813	ASTE1	NA	0,84488259	NA	NA	NA	NA	NA	NA	NA
ILMN_1753164	IPO8	NA	0,84868364	NA	NA	NA	NA	NA	NA	NA
ILMN_2367258	SMOX	NA	0,88423692	NA	NA	NA	NA	NA	NA	NA
ILMN_1697594	ANKRD28	NA	0,89986957	NA	NA	NA	NA	NA	NA	NA
ILMN_3235964	HSPA7	NA	0,92321475	NA	NA	NA	NA	NA	NA	NA
ILMN_1737394	LMNA	NA	0,94135722	NA	NA	NA	NA	NA	NA	NA
ILMN_1717337	MARCH7	NA	0,95346393	NA	NA	NA	NA	NA	NA	NA
ILMN_1662557	MAP7D3	NA	0,97562495	NA	NA	NA	NA	NA	NA	NA
ILMN_1736112	ARHGAP10	0,010049582	NA	NA	NA	NA	NA	NA	NA	NA
ILMN_2354515	NDUFAF3	0,010379653	NA	NA	NA	NA	NA	NA	NA	NA
ILMN_1778764	BUB3	0,01040118	NA	NA	NA	NA	NA	NA	NA	NA
ILMN_1754531	AP4E1	0,01074699	NA	NA	NA	NA	NA	NA	NA	NA
ILMN_1771962	GLI3	0,010880306	NA	NA	NA	NA	NA	NA	NA	NA
ILMN_2197030	ZFYVE21	0,010996478	NA	NA	NA	NA	NA	NA	NA	NA
ILMN_1797964	ARL6IP6	0,01147258	NA	NA	NA	NA	NA	NA	NA	NA
ILMN_2103295	TINP1	0,011504791	NA	NA	NA	NA	NA	NA	NA	NA
ILMN_1778236	PTPN11	0,011774006	NA	NA	NA	NA	NA	NA	NA	NA
ILMN_1765401	RRN3	0,011883417	NA	NA	NA	NA	NA	NA	NA	NA
ILMN_1801504	RUNX1	0,012568922	NA	NA	NA	NA	NA	NA	NA	NA
ILMN_1763198	STAT6	0,013012668	NA	NA	NA	NA	NA	NA	NA	NA
ILMN_1814859	DDX47	0,013071605	NA	NA	NA	NA	NA	NA	NA	NA
ILMN_2364131	TTPAL	0,013106086	NA	NA	NA	NA	NA	NA	NA	NA
ILMN_1658858	SP140L	0,013192205	NA	NA	NA	NA	NA	NA	NA	NA
ILMN_1790549	TSPAN3	0,013352863	NA	NA	NA	NA	NA	NA	NA	NA
ILMN_1846517		0,013651518	NA	NA	NA	NA	NA	NA	NA	NA
ILMN_1662617	PPP2R3C	0,014187505	NA	NA	NA	NA	NA	NA	NA	NA
ILMN_1789596	ETV6	0,01435284	NA	NA	NA	NA	NA	NA	NA	NA
ILMN_1705151	SF3A3	0,014740356	NA	NA	NA	NA	NA	NA	NA	NA
ILMN_1663664	MRPS10	0,014865232	NA	NA	NA	NA	NA	NA	NA	NA
ILMN_1813491	SPTLC1	0,0150441	NA	NA	NA	NA	NA	NA	NA	NA
ILMN_1746465	FJX1	0,015149286	NA	NA	NA	NA	NA	NA	NA	NA
ILMN_1733562	TFB1M	0,015207711	NA	NA	NA	NA	NA	NA	NA	NA
ILMN_1815552	NTAN1	0,015359889	NA	NA	NA	NA	NA	NA	NA	NA
ILMN_1815079	TICAM1	0,016292111	NA	NA	NA	NA	NA	NA	NA	NA
ILMN_1653429	SLC35A3	0,016653554	NA	NA	NA	NA	NA	NA	NA	NA
ILMN_1669881	TSPAN13	0,016684584	NA	NA	NA	NA	NA	NA	NA	NA
ILMN_1657509	TSEN54	0,017076961	NA	NA	NA	NA	NA	NA	NA	NA
ILMN_1783350	PCNXL3	0,017137098	NA	NA	NA	NA	NA	NA	NA	NA
ILMN_1811991	C17orf101	0,017276106	NA	NA	NA	NA	NA	NA	NA	NA
ILMN_3243366	C2orf55	0,017658364	NA	NA	NA	NA	NA	NA	NA	NA
ILMN_1689702	STAR	0,018020579	NA	NA	NA	NA	NA	NA	NA	NA
ILMN_1700822	DPP3	0,018502735	NA	NA	NA	NA	NA	NA	NA	NA
ILMN_1749478	TCEAL3	0,018832837	NA	NA	NA	NA	NA	NA	NA	NA

ILMN_1679919	ASCC2	0,018987812	NA	NA	NA	NA	NA	NA	NA	NA
ILMN_1707720	SLC1A5	0,019434512	NA	NA	NA	NA	NA	NA	NA	NA
ILMN_1790533	PHACTR2	0,01963548	NA	NA	NA	NA	NA	NA	NA	NA
ILMN_2391178	STAR	0,020772174	NA	NA	NA	NA	NA	NA	NA	NA
ILMN_1726704	RSC1A1	0,020913355	NA	NA	NA	NA	NA	NA	NA	NA
ILMN_2391176	STAR	0,021166119	NA	NA	NA	NA	NA	NA	NA	NA
ILMN_1721093	TAF10	0,021241834	NA	NA	NA	NA	NA	NA	NA	NA
ILMN_1711368	TRAPPC9	0,021420158	NA	NA	NA	NA	NA	NA	NA	NA
ILMN_1716552	ENAH	0,02180368	NA	NA	NA	NA	NA	NA	NA	NA
ILMN_1774604	PNKD	0,022556002	NA	NA	NA	NA	NA	NA	NA	NA
ILMN_1673804	ZNF426	0,022567549	NA	NA	NA	NA	NA	NA	NA	NA
ILMN_1729775	OPA1	0,022739656	NA	NA	NA	NA	NA	NA	NA	NA
ILMN_1718042	ZNF549	0,023586865	NA	NA	NA	NA	NA	NA	NA	NA
ILMN_2301955	THAP1	0,023652611	NA	NA	NA	NA	NA	NA	NA	NA
ILMN_1772124	ATRN	0,024130675	NA	NA	NA	NA	NA	NA	NA	NA
ILMN_1697959	SLC35B4	0,024141341	NA	NA	NA	NA	NA	NA	NA	NA
ILMN_1781374	TUFT1	0,024208642	NA	NA	NA	NA	NA	NA	NA	NA
ILMN_1655904	ROR1	0,024411726	NA	NA	NA	NA	NA	NA	NA	NA
ILMN_1809850	RCN3	0,025127668	NA	NA	NA	NA	NA	NA	NA	NA
ILMN_1696975	USP1	0,02529475	NA	NA	NA	NA	NA	NA	NA	NA
ILMN_1712751	HADHA	0,025895425	NA	NA	NA	NA	NA	NA	NA	NA
ILMN_1723481	CHST3	0,026293682	NA	NA	NA	NA	NA	NA	NA	NA
ILMN_1782257	METT11D1	0,026317535	NA	NA	NA	NA	NA	NA	NA	NA
ILMN_1694259	NSA2	0,02648742	NA	NA	NA	NA	NA	NA	NA	NA
ILMN_1740737	DCPS	0,027461624	NA	NA	NA	NA	NA	NA	NA	NA
ILMN_1747697	CHML	0,027766068	NA	NA	NA	NA	NA	NA	NA	NA
ILMN_1815134	PI4K2B	0,028512744	NA	NA	NA	NA	NA	NA	NA	NA
ILMN_1704795	MARK3	0,028960667	NA	NA	NA	NA	NA	NA	NA	NA
ILMN_1739161	PPAP2A	0,029583849	NA	NA	NA	NA	NA	NA	NA	NA
ILMN_1781761	ENPP4	0,029757375	NA	NA	NA	NA	NA	NA	NA	NA
ILMN_1738124	ZNF772	0,030936193	NA	NA	NA	NA	NA	NA	NA	NA
ILMN_1677877	UBE2L3	0,031074906	NA	NA	NA	NA	NA	NA	NA	NA
ILMN_1749821	MED28	0,032261731	NA	NA	NA	NA	NA	NA	NA	NA
ILMN_2123871	TMEM18	0,032782166	NA	NA	NA	NA	NA	NA	NA	NA
ILMN_2121437	NCL	0,032903856	NA	NA	NA	NA	NA	NA	NA	NA
ILMN_3232219	LOC100134189	0,033282806	NA	NA	NA	NA	NA	NA	NA	NA
ILMN_1804522	CCDC47	0,033346153	NA	NA	NA	NA	NA	NA	NA	NA
ILMN_2346573	PSME3	0,033987109	NA	NA	NA	NA	NA	NA	NA	NA
ILMN_1698307	DBNL	0,034285714	NA	NA	NA	NA	NA	NA	NA	NA
ILMN_1715416	NUP188	0,034345649	NA	NA	NA	NA	NA	NA	NA	NA
ILMN_1786328	WDR40A	0,035282648	NA	NA	NA	NA	NA	NA	NA	NA
ILMN_1682233	ESCO1	0,035422309	NA	NA	NA	NA	NA	NA	NA	NA
ILMN_1766169	BCAT1	0,036258928	NA	NA	NA	NA	NA	NA	NA	NA
ILMN_1688178	RRP7A	0,03774389	NA	NA	NA	NA	NA	NA	NA	NA
ILMN_2226304	ANKRD50	0,039302731	NA	NA	NA	NA	NA	NA	NA	NA
ILMN_2157075	LRCH4	0,0394535	NA	NA	NA	NA	NA	NA	NA	NA
ILMN_1802519	VPS36	0,040950679	NA	NA	NA	NA	NA	NA	NA	NA
ILMN_2354855	OTUB1	0,045056496	NA	NA	NA	NA	NA	NA	NA	NA
ILMN_3277332	LOC441453	0,046033758	NA	NA	NA	NA	NA	NA	NA	NA
ILMN_1667306	RANBP10	0,04617792	NA	NA	NA	NA	NA	NA	NA	NA
ILMN_1708369	EPS15L1	0,046482718	NA	NA	NA	NA	NA	NA	NA	NA
ILMN_1797181	LOC93622	0,046836335	NA	NA	NA	NA	NA	NA	NA	NA
ILMN_1795419	C2orf24	0,046842274	NA	NA	NA	NA	NA	NA	NA	NA
ILMN_1676449	SLIT2	0,047425161	NA	NA	NA	NA	NA	NA	NA	NA
ILMN_1752837	ARL8B	0,050868103	NA	NA	NA	NA	NA	NA	NA	NA
ILMN_1775074	TUBGCP2	0,051270597	NA	NA	NA	NA	NA	NA	NA	NA
ILMN_1734483	BSDC1	0,051842783	NA	NA	NA	NA	NA	NA	NA	NA
ILMN_1778673	GOLGA7	0,053400835	NA	NA	NA	NA	NA	NA	NA	NA
ILMN_1751851	CECR1	0,053871588	NA	NA	NA	NA	NA	NA	NA	NA
ILMN_1687958	SLC25A22	0,05510663	NA	NA	NA	NA	NA	NA	NA	NA
ILMN_1657754	RPAP2	0,055355134	NA	NA	NA	NA	NA	NA	NA	NA
ILMN_1857113		0,056310709	NA	NA	NA	NA	NA	NA	NA	NA
ILMN_1717261	HLA-DRB3	0,056406002	NA	NA	NA	NA	NA	NA	NA	NA
ILMN_1801864	LOC730455	0,057772187	NA	NA	NA	NA	NA	NA	NA	NA
ILMN_2115154	NUPL2	0,057854448	NA	NA	NA	NA	NA	NA	NA	NA
ILMN_1663092	CITED2	0,059078914	NA	NA	NA	NA	NA	NA	NA	NA
ILMN_1800308	GTF2H4	0,059668669	NA	NA	NA	NA	NA	NA	NA	NA
ILMN_1803136	RAB4B	0,05978495	NA	NA	NA	NA	NA	NA	NA	NA
ILMN_1776121	MGC42367	0,070605251	NA	NA	NA	NA	NA	NA	NA	NA
ILMN_1756873	USP48	0,071346938	NA	NA	NA	NA	NA	NA	NA	NA
ILMN_1863185		0,07258326	NA	NA	NA	NA	NA	NA	NA	NA
ILMN_1708778	ASS1	0,074671382	NA	NA	NA	NA	NA	NA	NA	NA
ILMN_2391765	C6orf48	0,075024391	NA	NA	NA	NA	NA	NA	NA	NA
ILMN_3218306	LOC439994	0,076039782	NA	NA	NA	NA	NA	NA	NA	NA

ILMN_1752247	AKAP13	0,08170235	NA	NA	NA	NA	NA	NA	NA	NA
ILMN_2346649	ZNF473	0,081926607	NA	NA	NA	NA	NA	NA	NA	NA
ILMN_1655563	KIAA0427	0,085431871	NA	NA	NA	NA	NA	NA	NA	NA
ILMN_1695731	TUBG1	0,087164749	NA	NA	NA	NA	NA	NA	NA	NA
ILMN_1804929	OXTR	0,088768603	NA	NA	NA	NA	NA	NA	NA	NA
ILMN_2394523	TMEM231	0,090188734	NA	NA	NA	NA	NA	NA	NA	NA
ILMN_2185563	ANKRA2	0,095883427	NA	NA	NA	NA	NA	NA	NA	NA
ILMN_2369953	ARL4A	0,096054528	NA	NA	NA	NA	NA	NA	NA	NA
ILMN_1907556		0,099355069	NA	NA	NA	NA	NA	NA	NA	NA
ILMN_1676749	C3orf15	0,103564737	NA	NA	NA	NA	NA	NA	NA	NA
ILMN_1745620	KRCC1	0,107249294	NA	NA	NA	NA	NA	NA	NA	NA
ILMN_1740571	LOC647859	0,108634429	NA	NA	NA	NA	NA	NA	NA	NA
ILMN_2343278	PPAP2A	0,109340281	NA	NA	NA	NA	NA	NA	NA	NA
ILMN_1692058	NDN	0,112240889	NA	NA	NA	NA	NA	NA	NA	NA
ILMN_2396786	ZNF266	0,114392116	NA	NA	NA	NA	NA	NA	NA	NA
ILMN_1714364	PTK2	0,118754076	NA	NA	NA	NA	NA	NA	NA	NA
ILMN_1730082	RPUSD4	0,125661609	NA	NA	NA	NA	NA	NA	NA	NA
ILMN_1727761	GMEB1	0,134463484	NA	NA	NA	NA	NA	NA	NA	NA
ILMN_1696701	LOC344595	0,144943405	NA	NA	NA	NA	NA	NA	NA	NA
ILMN_3253304	BRI3P1	0,146239237	NA	NA	NA	NA	NA	NA	NA	NA
ILMN_1778677	MRS2	0,151172052	NA	NA	NA	NA	NA	NA	NA	NA
ILMN_1782085	ATPBD4	0,152917547	NA	NA	NA	NA	NA	NA	NA	NA
ILMN_1659075	HLA-DOA	0,16269301	NA	NA	NA	NA	NA	NA	NA	NA
ILMN_1725760	YTHDC2	0,166007531	NA	NA	NA	NA	NA	NA	NA	NA
ILMN_1679178	ATP5D	0,167046225	NA	NA	NA	NA	NA	NA	NA	NA
ILMN_1874530	DIAPH3	0,167633352	NA	NA	NA	NA	NA	NA	NA	NA
ILMN_1776516	ITPKA	0,167810259	NA	NA	NA	NA	NA	NA	NA	NA
ILMN_2410986	STAT3	0,178136028	NA	NA	NA	NA	NA	NA	NA	NA
ILMN_1811648	DCAKD	0,178608708	NA	NA	NA	NA	NA	NA	NA	NA
ILMN_2229379	KIT	0,178906997	NA	NA	NA	NA	NA	NA	NA	NA
ILMN_1781400	SLC7A2	0,192569375	NA	NA	NA	NA	NA	NA	NA	NA
ILMN_1809400	FAM49B	0,21680123	NA	NA	NA	NA	NA	NA	NA	NA
ILMN_1654851	FAM134A	0,234864425	NA	NA	NA	NA	NA	NA	NA	NA
ILMN_3228688	LOC730415	0,239028632	NA	NA	NA	NA	NA	NA	NA	NA
ILMN_1661485	RBM34	0,2421712	NA	NA	NA	NA	NA	NA	NA	NA
ILMN_1767360	IL10RB	0,243716007	NA	NA	NA	NA	NA	NA	NA	NA
ILMN_1698745	CLRN1	0,266803375	NA	NA	NA	NA	NA	NA	NA	NA
ILMN_1658802	KRTCAP2	0,304968477	NA	NA	NA	NA	NA	NA	NA	NA
ILMN_1752592	HLA-DRB4	0,325668566	NA	NA	NA	NA	NA	NA	NA	NA
ILMN_3245413	DENND5A	0,325950457	NA	NA	NA	NA	NA	NA	NA	NA
ILMN_1781983	AP1B1	0,348657339	NA	NA	NA	NA	NA	NA	NA	NA
ILMN_1708427	KPNA3	0,369231955	NA	NA	NA	NA	NA	NA	NA	NA
ILMN_1802257	PCTP	0,382714959	NA	NA	NA	NA	NA	NA	NA	NA
ILMN_1745807	TMEM62	0,415815063	NA	NA	NA	NA	NA	NA	NA	NA
ILMN_2204876	FLVCR2	0,417716726	NA	NA	NA	NA	NA	NA	NA	NA
ILMN_1720431	ZNF420	0,434920712	NA	NA	NA	NA	NA	NA	NA	NA
ILMN_1712231	SH3YL1	0,436068448	NA	NA	NA	NA	NA	NA	NA	NA
ILMN_1697567	TPM3	0,441598375	NA	NA	NA	NA	NA	NA	NA	NA
ILMN_2194229	TMEM128	0,443671893	NA	NA	NA	NA	NA	NA	NA	NA
ILMN_1695959	C21orf63	0,454886083	NA	NA	NA	NA	NA	NA	NA	NA
ILMN_1697971	TRIM38	0,508912981	NA	NA	NA	NA	NA	NA	NA	NA
ILMN_1770281	ERC1	0,571164171	NA	NA	NA	NA	NA	NA	NA	NA
ILMN_1847965		0,573758788	NA	NA	NA	NA	NA	NA	NA	NA
ILMN_1686388	LOC644330	0,608274114	NA	NA	NA	NA	NA	NA	NA	NA
ILMN_1658356	PAMR1	0,700501118	NA	NA	NA	NA	NA	NA	NA	NA
ILMN_2157709	C14orf19	0,808161576	NA	NA	NA	NA	NA	NA	NA	NA
