# Supplementary material for: Stratifying Cumulus Cell Samples Based on Molecular Profiling to Help Resolve Biomarker Discrepancies and to Predict Oocyte Developmental Competence
Source: Int J Mol Sci. 2021 Jun 15;22(12):6377. doi: 10.3390/ijms22126377 (PMC8232172; doi:10.3390/ijms22126377)
Supplement: Supplementary file 1 [file ijms-22-06377-s001.zip › Suppl Table 3 Genes sig correlated long prot only with their pvalues at p0.01.pdf]

Supplementary Table 3

PROBE_ID	SYMBOL	Estrogen (d12)	Endometrium Size (d12)	Lead Follice Size (d12)	ZP birefringe	Lead Follicle Size Slope	Spindle Presence	Estrogen slope	Endometrium Size Slope	Pronuceli pattern	LongNeg vs L
ILMN_3245057	ASAP1	NA	0,002328637	NA	NA	0,003238415	NA	NA	NA	NA	1,94E-06
ILMN_1690963	ASAP1	NA	0,006405945	NA	NA	0,008604614	NA	NA	NA	NA	2,87E-06
ILMN_2229032	NME6	NA	0,002711681	NA	NA	0,006838231	NA	NA	NA	NA	3,84E-06
ILMN_1727592	FRMD5	NA	NA	NA	NA	0,00782061	NA	NA	0,000628978	NA	3,86E-06
ILMN_1784300	TUBA4A	NA	NA	NA	NA	NA	NA	NA	0,001042513	NA	4,73E-05
ILMN_1687315	RXRA	NA	NA	NA	NA	NA	NA	NA	NA	NA	5,26E-05
ILMN_1793522	PRKAB1	NA	NA	NA	NA	NA	NA	NA	0,002449521	NA	6,51E-05
ILMN_2098418	LOC652968	NA	0,001117633	NA	NA	NA	NA	NA	NA	NA	7,19E-05
ILMN_1685580	CBLB	NA	0,005109406	NA	NA	NA	NA	NA	NA	NA	7,75E-05
ILMN_1700257	C4orf32	NA	NA	NA	NA	NA	NA	NA	NA	NA	8,44E-05
ILMN_1700384	KIAA1522	NA	NA	NA	NA	NA	NA	NA	0,004026255	NA	8,51E-05
ILMN_2056760	MKRN2	NA	NA	NA	NA	NA	NA	NA	NA	NA	8,86E-05
ILMN_2048700	ATAD2	NA	NA	NA	NA	0,008038488	NA	NA	NA	NA	9,09E-05
ILMN_2392356	CTPS2	NA	NA	NA	NA	NA	NA	NA	NA	0,008709029	9,49E-05
ILMN_1794885	FRMD5	NA	0,003350692	NA	NA	NA	NA	NA	0,006457393	NA	9,71E-05
ILMN_1767068	SMAD6	NA	NA	0,00935199	NA	0,001051693	NA	NA	NA	NA	0,00010001
ILMN_1730658	YTHDF2	NA	NA	0,004763106	NA	6,94E-07	NA	NA	NA	NA	0,00010023
ILMN_2391355	STAMBP	NA	NA	NA	NA	NA	NA	NA	NA	NA	0,00011895
ILMN_2387090	CGGBP1	NA	NA	NA	NA	0,008626681	NA	NA	NA	NA	0,00012082
ILMN_1728202	TMEM22	NA	NA	NA	NA	NA	NA	NA	0,006134888	NA	0,00012101
ILMN_1807044	UBAC1	NA	NA	NA	NA	NA	NA	NA	0,002906172	NA	0,00015542
ILMN_1748272	MAP3K7IP3	NA	NA	NA	NA	0,008476593	NA	NA	0,007314027	NA	0,00016277
ILMN_1703228	AGFG2	NA	0,005645769	NA	NA	NA	NA	NA	NA	NA	0,00016442
ILMN_1664802	WSB1	NA	NA	NA	NA	NA	NA	NA	NA	NA	0,00016986
ILMN_1664772	ATP2B4	NA	NA	NA	NA	NA	NA	NA	0,002402601	NA	0,00017247
ILMN_1794490	FLJ10781	NA	NA	NA	NA	0,002100052	NA	NA	0,005028692	NA	0,00017379
ILMN_1704571	FAM53B	NA	NA	NA	NA	0,00472146	NA	NA	NA	NA	0,00021626
ILMN_1739253	STAMBP	NA	NA	NA	NA	NA	NA	NA	NA	NA	0,00021759
ILMN_1794875	AGPAT9	NA	NA	0,00131796	NA	0,000155881	NA	NA	NA	NA	0,00022091
ILMN_1797298	ARMC7	NA	0,00260388	NA	NA	NA	NA	NA	NA	NA	0,00022226
ILMN_3238452	FAM21D	NA	0,006115872	NA	NA	0,001126144	NA	NA	NA	NA	0,00023966
ILMN_1778991	NFIB	NA	5,32E-05	NA	NA	NA	NA	NA	NA	NA	0,00024089
ILMN_3234142	LOC728855	NA	NA	NA	NA	NA	NA	NA	NA	NA	0,00024947
ILMN_1811148	BMP2K	NA	NA	NA	NA	NA	NA	NA	NA	NA	0,00026702
ILMN_1739810	RAI1	NA	NA	NA	NA	NA	NA	NA	NA	NA	0,00026975
ILMN_2231020	TMEM185B	NA	NA	0,006203819	NA	0,000412056	NA	NA	NA	NA	0,00030456
ILMN_1770610	MERTK	NA	0,006045728	NA	NA	NA	NA	NA	0,008753576	NA	0,00031183
ILMN_1729051	MSH6	NA	NA	NA	NA	0,002938628	NA	NA	NA	NA	0,00031243
ILMN_1702320	JAKMIP2	NA	NA	NA	NA	0,006040431	NA	NA	NA	NA	0,00032113
ILMN_1705116	C6orf85	NA	NA	NA	NA	NA	NA	NA	NA	NA	0,00032219
ILMN_1667417	RAB23	NA	NA	NA	NA	NA	NA	NA	NA	NA	0,00033729
ILMN_1912737		NA	NA	NA	NA	NA	NA	NA	NA	NA	0,00037455
ILMN_1757406	HIST1H1C	NA	NA	NA	NA	NA	NA	NA	NA	NA	0,00037561
ILMN_2373556	SPAST	NA	NA	NA	NA	0,004451789	NA	NA	NA	0,005885664	0,00039046
ILMN_1713496	ST3GAL5	NA	NA	0,009126816	NA	0,006038069	NA	NA	NA	NA	0,00039305
ILMN_1808157	RUNDC3B	NA	NA	NA	NA	NA	NA	NA	NA	NA	0,00044845
ILMN_1708841	GOLPH3	NA	NA	NA	NA	NA	NA	NA	0,000471122	NA	0,00046693
ILMN_1797031	HSPBAP1	NA	NA	NA	NA	NA	NA	NA	NA	NA	0,00046835
ILMN_1784113	NAT14	NA	NA	NA	NA	NA	NA	NA	NA	NA	0,00047338
ILMN_2346997	RAB23	NA	NA	NA	NA	0,008045882	NA	NA	NA	NA	0,00047946
ILMN_2138589	MERTK	NA	NA	NA	NA	NA	NA	NA	0,008237115	NA	0,00052713
ILMN_3240524	MFSD6	NA	0,007659396	NA	NA	NA	NA	NA	0,003612931	NA	0,00054106
ILMN_1767475	CERK	NA	NA	NA	NA	NA	NA	NA	NA	NA	0,00055345
ILMN_1674706	MTHFD2	NA	NA	NA	NA	NA	NA	NA	NA	NA	0,00055716
ILMN_1720710	HSPB3	NA	NA	NA	NA	0,006635494	NA	NA	NA	NA	0,00056364
ILMN_1706959	TIMM22	NA	NA	NA	NA	NA	NA	NA	NA	NA	0,00056599
ILMN_2384591	HN1	NA	0,002661281	NA	NA	NA	NA	NA	0,00107	NA	0,0005752
ILMN_1717046	MOBKL2B	NA	NA	NA	NA	0,005630739	NA	NA	NA	NA	0,00060001
ILMN_1848552		NA	9,93E-05	NA	NA	NA	NA	NA	0,007597487	NA	0,0006086
ILMN_2128795	LRIG1	NA	NA	NA	NA	NA	NA	NA	NA	NA	0,00063182
ILMN_1734543	PTPRE	NA	NA	NA	NA	0,005859951	NA	NA	NA	NA	0,00063264
ILMN_1745497	C12orf26	NA	NA	NA	NA	0,002295083	NA	NA	NA	NA	0,00063821
ILMN_2233366	ASAP1	NA	0,006368067	NA	NA	NA	NA	NA	0,002877589	NA	0,00066362
ILMN_2383611	PTPRE	NA	NA	NA	NA	0,007722139	NA	NA	NA	NA	0,00067949
ILMN_1681634	PXMP2	NA	0,001352778	NA	NA	0,007056848	NA	NA	NA	NA	0,00070709
ILMN_1770084	TACC1	NA	NA	NA	NA	NA	NA	NA	0,009934497	NA	0,00072844
ILMN_1738407	ULBP1	NA	0,003493328	NA	NA	NA	NA	NA	0,001495073	NA	0,00075693
ILMN_1811636	IFT57	NA	0,000306416	NA	NA	0,003215598	NA	NA	NA	NA	0,00078088
ILMN_2364521	AXL	NA	NA	NA	NA	0,008871759	NA	NA	NA	NA	0,00079149
ILMN_1707342	LRIG1	NA	NA	NA	NA	NA	NA	NA	NA	NA	0,00080885
ILMN_1672589	SEMA4B	NA	NA	NA	NA	0,00965021	NA	NA	NA	NA	0,00080936
ILMN_1651496	HIST1H2BD	NA	0,000247912	NA	NA	0,002250783	NA	NA	NA	NA	0,00082582
ILMN_1788098	LHCGR	NA	NA	NA	NA	NA	NA	NA	NA	NA	0,00083538
ILMN_1710209	MFSD6	NA	NA	NA	NA	NA	NA	NA	0,003741459	NA	0,00083877
ILMN_1813975	ADI1	NA	0,005880408	NA	NA	0,00338794	NA	NA	0,003767084	NA	0,00084359
ILMN_3299393	PNMAL1	NA	NA	NA	NA	0,005154775	NA	NA	0,001989765	NA	0,00085097
ILMN_1803988	MCL1	NA	NA	NA	NA	NA	NA	NA	NA	NA	0,00086497
ILMN_1734288	DUSP18	NA	0,005809524	NA	NA	NA	NA	NA	NA	0,00525063	0,00087374
ILMN_2160005	NUMA1	NA	NA	NA	NA	0,000819092	NA	NA	NA	NA	0,00091902
ILMN_1768480	VGLL4	NA	NA	NA	NA	0,003546714	NA	NA	NA	NA	0,00095625
ILMN_1810832	ZNF343	NA	NA	NA	NA	0,002258793	NA	NA	NA	NA	0,00097318
ILMN_1735045	A4GALT	NA	NA	NA	NA	0,003366518	NA	NA	NA	NA	0,00099472
ILMN_3227263	SLC22A23	NA	NA	NA	NA	NA	NA	NA	NA	NA	0,00101639
ILMN_1764380	GLTP	NA	NA	NA	NA	NA	NA	NA	NA	NA	0,00103423
ILMN_3298423	TOX2	NA	NA	NA	NA	NA	NA	NA	NA	NA	0,00103454
ILMN_2376263	SMARCA1	NA	NA	NA	NA	NA	NA	NA	NA	NA	0,00103944
ILMN_1722309	ENDOG	NA	NA	NA	NA	NA	NA	NA	NA	NA	0,00105285
ILMN_1737988	PRNP	NA	0,001602142	NA	NA	NA	NA	NA	NA	NA	0,00107482
ILMN_1748844	CNKSR3	NA	NA	NA	NA	NA	NA	NA	0,003131764	NA	0,00109099
ILMN_1723662	ABHD2	NA	NA	NA	NA	NA	NA	NA	NA	NA	0,00109518
ILMN_3244323	LOC148413	NA	NA	NA	NA	NA	NA	NA	NA	NA	0,00109586
ILMN_1796075	VPS41	NA	NA	NA	NA	0,000402579	NA	NA	NA	NA	0,00110673
ILMN_1718852	PLCL1	NA	0,005799443	NA	NA	0,007785357	NA	NA	NA	NA	0,00111611
ILMN_2377900	MAP1B	NA	NA	NA	NA	NA	NA	NA	NA	NA	0,00112587
ILMN_1677396	NDFIP2	NA	NA	NA	NA	NA	NA	NA	NA	NA	0,00114142
ILMN_1905548		NA	0,006285366	NA	NA	NA	NA	NA	NA	NA	0,00114415
ILMN_1731720	PDRG1	NA	NA	NA	NA	NA	NA	NA	NA	NA	0,00114467
ILMN_1697286	SF3A1	NA	0,001375449	NA	NA	NA	NA	0,003995768	0,009084597	NA	0,00116473
ILMN_1802377	IQCG	NA	NA	NA	NA	0,00388188	NA	NA	NA	NA	0,00119114
ILMN_1763540	PSME4	NA	NA	NA	NA	NA	NA	NA	NA	NA	0,00119216
ILMN_1713688	DHX32	NA	NA	NA	NA	NA	NA	NA	NA	NA	0,00119286
ILMN_2377430	AGPAT2	NA	4,37E-05	NA	NA	0,008730626	NA	NA	NA	NA	0,00119813
ILMN_1665384	SH3BP5L	NA	NA	NA	NA	0,001550117	NA	NA	NA	NA	0,00123827
ILMN_2049766	NFE2L3	NA	NA	NA	NA	NA	NA	NA	NA	NA	0,00128169
ILMN_2055523	CSGALNACT1	NA	NA	NA	NA	NA	NA	NA	0,000545408	NA	0,00130728
ILMN_1740923	C20orf54	NA	NA	NA	NA	NA	NA	NA	NA	NA	0,00130906
ILMN_1789338	SORBS3	NA	0,001064507	NA	NA	0,001838894	NA	NA	NA	NA	0,00132254

ILMN_2358560	TIAM2	NA	NA	NA	NA	NA	NA	NA	NA	NA	0,00132894
ILMN_1745223	CDC42EP4	NA	NA	NA	NA	NA	NA	NA	NA	NA	0,00133
ILMN_3235472	WDYHV1	NA	NA	NA	NA	NA	NA	NA	NA	NA	0,00133003
ILMN_1717326	SLC29A3	NA	0,00347194	NA	NA	0,003490282	NA	NA	NA	NA	0,0013631
ILMN_1706990	ZNF271	NA	0,002462327	NA	NA	0,001389795	NA	NA	NA	NA	0,00136679
ILMN_1803060	MAML1	NA	NA	NA	NA	NA	NA	NA	NA	NA	0,00136902
ILMN_2299045	BMP2K	NA	NA	NA	NA	NA	NA	NA	NA	NA	0,00137222
ILMN_1740903	C7orf49	NA	NA	NA	NA	NA	NA	NA	0,003082166	NA	0,00137463
ILMN_2082314	TOM1	NA	NA	NA	NA	NA	NA	NA	NA	NA	0,0014297
ILMN_1745152	UQCC	NA	NA	NA	NA	NA	NA	NA	NA	NA	0,00144425
ILMN_1693145	BUB3	NA	0,005224858	NA	NA	NA	NA	NA	NA	0,004395829	0,00146689
ILMN_2350801	SLC25A29	NA	NA	NA	NA	0,004113917	NA	NA	NA	NA	0,00150045
ILMN_2401641	ALDH3A2	NA	NA	0,007171416	NA	0,001630791	NA	NA	NA	NA	0,0015065
ILMN_1712628	GTF2IRD1	NA	NA	NA	NA	0,000286255	NA	NA	NA	NA	0,00150915
ILMN_2405521	MTHFD2	NA	NA	0,008019869	NA	NA	NA	NA	NA	0,009004634	0,00152165
ILMN_1724194	NPEPL1	NA	2,20E-06	NA	NA	NA	NA	NA	NA	NA	0,00158588
ILMN_2043816	ARPC5L	NA	NA	NA	NA	NA	NA	NA	NA	NA	0,00161509
ILMN_1659975	C1orf216	NA	NA	NA	NA	NA	NA	NA	NA	0,003649828	0,00162498
ILMN_1700541	FBLN1	NA	0,002536232	0,002942737	NA	0,000242862	NA	NA	NA	NA	0,00164353
ILMN_1717393	PTCHD1	NA	0,002108043	NA	NA	NA	NA	NA	0,006584286	NA	0,00165078
ILMN_1799015	PXMP2	NA	0,001423022	NA	NA	NA	NA	NA	NA	NA	0,00165328
ILMN_1760855	OCRL	NA	NA	NA	NA	NA	NA	NA	NA	NA	0,00165497
ILMN_2403237	CHN2	NA	NA	NA	NA	NA	NA	NA	NA	NA	0,00169304
ILMN_1756877	C14orf179	NA	0,000691241	NA	NA	NA	NA	NA	NA	NA	0,00172034
ILMN_3307659	SFT2D2	NA	NA	NA	NA	NA	NA	NA	0,009811776	NA	0,00172327
ILMN_2389935	FYTTD1	NA	NA	NA	NA	0,002016263	NA	NA	NA	NA	0,00172862
ILMN_1670076	FAM155B	NA	NA	NA	NA	NA	NA	NA	NA	NA	0,00174188
ILMN_1677962	GPHN	NA	0,0038161	NA	NA	NA	NA	NA	0,001910624	NA	0,00185388
ILMN_2366490	ZNF706	NA	NA	NA	NA	0,000734199	NA	NA	NA	NA	0,00186761
ILMN_1731181	TEX2	NA	NA	NA	NA	NA	NA	NA	3,47E-05	NA	0,00187831
ILMN_1757415	C1orf163	NA	NA	NA	NA	0,004404835	NA	NA	NA	NA	0,00188228
ILMN_1682919	PAFAH2	NA	0,003165318	NA	NA	NA	NA	NA	NA	NA	0,00190113
ILMN_2145116	TMEM173	NA	NA	NA	NA	0,000614802	NA	NA	NA	NA	0,00191204
ILMN_1704353	IGSF3	NA	NA	NA	NA	NA	NA	NA	NA	NA	0,00192284
ILMN_1661636	ZMYM2	NA	NA	NA	NA	0,002621634	NA	NA	NA	NA	0,00198148
ILMN_3307772	PACSIN2	NA	0,007212882	NA	NA	0,000394862	NA	NA	NA	NA	0,00201125
ILMN_1697218	MED22	NA	NA	NA	NA	NA	NA	NA	NA	NA	0,00203275
ILMN_1804834	C6orf130	NA	NA	NA	NA	NA	NA	NA	0,005066951	NA	0,00203795
ILMN_1799819	MARS	NA	0,008261481	NA	NA	NA	NA	NA	NA	NA	0,00205657
ILMN_2117171	LMO4	NA	NA	NA	NA	NA	NA	NA	NA	NA	0,00208236
ILMN_2181445	BCL2L13	NA	NA	NA	NA	NA	NA	NA	NA	NA	0,00210305
ILMN_1745813	KIAA1279	NA	NA	NA	NA	NA	NA	NA	NA	NA	0,0021058
ILMN_1737685	CRLS1	NA	NA	NA	NA	NA	NA	NA	0,000133115	NA	0,00210979
ILMN_1652753	PAAF1	NA	NA	NA	NA	0,006779525	NA	NA	NA	NA	0,00211244
ILMN_2147517	CD58	NA	NA	NA	NA	NA	NA	NA	0,005343614	NA	0,00213531
ILMN_3180196	LOC1001280	NA	0,00689277	NA	NA	NA	NA	NA	0,002906778	NA	0,00214249
ILMN_1779751	C7orf55	NA	0,004747938	NA	NA	NA	NA	NA	NA	NA	0,00214341
ILMN_1735192	COLEC11	NA	0,0015614	0,005260639	NA	0,00075467	NA	NA	NA	NA	0,00218967
ILMN_2186137	RRAD	NA	NA	NA	NA	NA	NA	NA	NA	NA	0,00221123
ILMN_3238740	CENPBD1	NA	NA	NA	NA	NA	NA	NA	NA	0,003333048	0,00222302
ILMN_1774127	STAC	NA	NA	NA	NA	0,007023979	NA	NA	NA	NA	0,00227855
ILMN_2206953	PLCL1	NA	NA	0,005413197	NA	0,001617599	NA	NA	NA	NA	0,00228286
ILMN_2092664	ADSS	NA	NA	NA	NA	NA	NA	NA	NA	NA	0,00230096
ILMN_1674032	ABCD3	NA	NA	NA	NA	NA	NA	NA	NA	NA	0,00231253
ILMN_1764177	JARID2	NA	NA	NA	NA	0,000994047	NA	NA	NA	NA	0,0023524
ILMN_1762426	DHFRL1	NA	NA	NA	NA	NA	NA	NA	NA	NA	0,00236084
ILMN_2051731	DUSP5P	0,008594252	NA	0,004836529	NA	NA	NA	NA	NA	0,008054452	0,00236109
ILMN_1673586	SLC6A6	NA	NA	NA	NA	NA	NA	NA	NA	NA	0,00238724
ILMN_2376258	SMARCA1	NA	NA	NA	NA	NA	NA	NA	NA	NA	0,00238748
ILMN_3199647	LOC645251	NA	0,00439589	0,001103094	NA	0,00011622	NA	NA	NA	NA	0,00239505
ILMN_1801441	RFTN2	NA	NA	0,005764357	NA	0,004891506	NA	NA	NA	NA	0,00241626
ILMN_3246538	LOC1001338	NA	NA	NA	NA	NA	NA	NA	NA	NA	0,00242567
ILMN_1796179	HIST1H2BK	NA	0,000675127	NA	NA	NA	NA	NA	NA	NA	0,00242778
ILMN_2382121	OCRL	NA	NA	NA	NA	NA	NA	NA	NA	NA	0,00244661
ILMN_2157099	CCNA1	NA	NA	NA	NA	0,006220661	NA	NA	NA	NA	0,00247886
ILMN_1696330	GUF1	NA	NA	NA	NA	NA	NA	NA	NA	NA	0,00248885
ILMN_1694708	LOC650737	NA	NA	NA	NA	0,002256571	NA	NA	NA	NA	0,00250899
ILMN_1776105	PSPH	NA	NA	NA	NA	NA	NA	NA	NA	NA	0,00251457
ILMN_1655482	TRIM27	NA	0,002537218	NA	NA	NA	NA	NA	NA	NA	0,00253164
ILMN_3230215	TOX2	NA	NA	NA	NA	0,000749073	NA	NA	NA	NA	0,00253422
ILMN_1731699	RAB15	NA	0,009998771	NA	NA	NA	NA	NA	0,009704177	NA	0,00255498
ILMN_1785268	CD58	NA	NA	NA	NA	NA	NA	NA	0,007776319	NA	0,00260227
ILMN_1838313		NA	NA	0,002856529	NA	NA	NA	NA	NA	0,002776847	0,00261999
ILMN_1708095	PANK2	NA	0,002258689	NA	NA	0,002194086	NA	NA	NA	NA	0,00264022
ILMN_3239168	LOC645332	NA	0,003423115	NA	NA	NA	NA	NA	NA	NA	0,00268025
ILMN_1700955	TCTEX1D2	NA	NA	NA	NA	NA	NA	NA	NA	NA	0,00268137
ILMN_1719219	ZNF616	NA	0,002437312	NA	NA	NA	NA	NA	NA	NA	0,00270664
ILMN_1652525	FAM125B	NA	0,001940801	NA	NA	0,001779411	NA	NA	NA	NA	0,00275326
ILMN_1791896	EBAG9	NA	NA	NA	NA	0,008978605	NA	NA	0,007358769	NA	0,00276736
ILMN_3243351	LOC646214	NA	NA	NA	NA	NA	NA	NA	NA	0,000378743	0,00284023
ILMN_1709044	TGIF2	0,000770741	0,002917014	0,004471193	NA	NA	NA	NA	NA	0,005199003	0,00294705
ILMN_1746408	MIDN	NA	0,001707222	NA	NA	NA	NA	NA	NA	NA	0,00297054
ILMN_3251550	PHLDA1	NA	NA	0,00119618	NA	0,007681719	NA	NA	NA	NA	0,00298846
ILMN_2143261	CXorf40B	NA	NA	0,007723953	NA	NA	NA	NA	NA	NA	0,00300992
ILMN_1726437	EFCAB3	NA	NA	NA	NA	NA	NA	NA	0,004926913	NA	0,00301114
ILMN_1794825	ALDH3A2	NA	NA	0,009507007	NA	0,003113208	NA	NA	NA	NA	0,0030352
ILMN_2313074	DEDD	NA	NA	0,003836398	NA	0,004910439	NA	NA	NA	NA	0,00304218
ILMN_2234956	LEPR	NA	NA	0,004437918	NA	0,000697259	NA	NA	NA	NA	0,00305259
ILMN_1777139	MAK16	NA	NA	NA	NA	NA	NA	NA	NA	NA	0,00306154
ILMN_1768004	PDCD4	NA	NA	NA	NA	NA	NA	NA	0,002552921	NA	0,00309876
ILMN_1792689	HIST1H2AC	0,001849456	NA	NA	NA	0,009673568	NA	NA	NA	0,001803482	0,00310485
ILMN_2397024	SPOP	NA	NA	NA	NA	NA	NA	NA	NA	NA	0,0031173
ILMN_1815035	DENND2C	NA	NA	NA	NA	NA	NA	NA	NA	0,002210116	0,00314671
ILMN_1770244	CBX1	NA	0,002167336	0,001518851	NA	8,16E-06	NA	NA	NA	NA	0,00314709
ILMN_1759154	PABPN1	0,004739908	NA	0,002138498	NA	0,003724473	NA	NA	NA	NA	0,00317696
ILMN_1687410	OSBPL11	NA	NA	0,00061595	NA	0,000119366	NA	NA	NA	NA	0,00321182
ILMN_1674034	H2AFY	NA	0,003420328	NA	NA	NA	NA	NA	0,001225397	NA	0,00322896
ILMN_3226769	LOC730074	NA	NA	NA	NA	NA	NA	NA	NA	NA	0,00326667
ILMN_1656393	PPP2R3A	NA	NA	NA	NA	NA	NA	NA	NA	NA	0,00334418
ILMN_2129927	EXT1	0,003907011	NA	NA	NA	NA	NA	NA	NA	0,005788692	0,00335219
ILMN_1676026	MRPS26	NA	NA	NA	NA	NA	NA	NA	0,006764942	NA	0,00335945
ILMN_1690546	PPP3CC	NA	NA	NA	NA	0,004610075	NA	NA	NA	NA	0,00340496
ILMN_1681118	CAPRIN2	NA	NA	NA	NA	0,000970803	NA	NA	NA	NA	0,00342195
ILMN_1710514	BCL3	NA	NA	0,000118536	NA	0,00072958	NA	NA	NA	NA	0,00344304
ILMN_2070355	SDHAF1	NA	NA	NA	NA	NA	NA	NA	NA	NA	0,00345787
ILMN_3208330	LOC1001327	NA	NA	NA	NA	NA	NA	NA	0,002886217	NA	0,0034812
ILMN_1671891	PID1	NA	NA	NA	NA	0,001242332	NA	NA	NA	NA	0,00350494
ILMN_3239378	TADA2B	NA	NA	NA	NA	NA	NA	NA	NA	NA	0,00352048
ILMN_1712786	AHCYL2	NA	NA	NA	NA	NA	NA	NA	NA	NA	0,00355857

ILMN_2179778	PHLDB2	NA	NA	NA	NA	NA	NA	NA	NA	0,003200384	0,00356235
ILMN_1752435	SEC22C	NA	NA	NA	NA	NA	NA	NA	NA	NA	0,00357413
ILMN_1809417	LRFN4	NA	NA	NA	NA	NA	NA	NA	NA	NA	0,00358414
ILMN_1761911	SCYL1BP1	NA	NA	NA	NA	NA	NA	NA	NA	NA	0,00366913
ILMN_2051519	RPL37A	NA	NA	NA	NA	NA	NA	NA	NA	NA	0,00368066
ILMN_3251472	TOMM20	NA	NA	NA	NA	NA	NA	NA	NA	NA	0,0036817
ILMN_1707631	MED10	NA	NA	NA	NA	NA	NA	NA	NA	NA	0,00372882
ILMN_1722726	RICS	NA	NA	NA	NA	NA	NA	NA	NA	NA	0,00375423
ILMN_1727458	HDAC1	NA	NA	NA	NA	NA	NA	NA	NA	NA	0,00375951
ILMN_1801928	YWHAZ	NA	NA	NA	NA	NA	NA	NA	0,00903923	NA	0,0037641
ILMN_1785158	HERPUD2	NA	NA	NA	NA	NA	NA	NA	0,003014787	NA	0,00381537
ILMN_1806818	MCM3	NA	NA	0,005754311	NA	0,002285792	NA	NA	NA	NA	0,00386795
ILMN_1712587	ARSB	NA	NA	NA	NA	NA	NA	NA	0,005573379	NA	0,00390594
ILMN_3250592	FAM86A	NA	NA	NA	NA	NA	NA	NA	NA	NA	0,00400663
ILMN_1708375	IRF1	NA	NA	NA	NA	NA	NA	NA	NA	NA	0,004024
ILMN_1716815	CEACAM1	NA	NA	NA	NA	NA	NA	NA	NA	NA	0,00404015
ILMN_1726704	RSC1A1	NA	NA	NA	NA	NA	NA	NA	NA	NA	0,00404547
ILMN_1775448	PFN2	NA	NA	NA	NA	NA	NA	NA	NA	NA	0,00404571
ILMN_1683127	ZNF281	NA	NA	NA	NA	NA	NA	NA	NA	NA	0,00406325
ILMN_1793672	SIX5	NA	NA	0,00848114	NA	0,002243433	NA	NA	NA	NA	0,00409717
ILMN_1812169	SERAC1	NA	NA	NA	NA	NA	NA	NA	0,003342764	NA	0,00410464
ILMN_1656840	VPS13D	NA	NA	0,006963341	NA	0,000646688	NA	NA	NA	0,003400513	0,00412872
ILMN_1779076	HINT3	NA	NA	NA	NA	NA	NA	NA	NA	NA	0,00414543
ILMN_2087060	TOMM7	0,003651894	0,001802331	0,003413895	NA	0,006528607	NA	NA	NA	NA	0,00414555
ILMN_1746426	TOMM70A	NA	0,00146143	NA	NA	NA	NA	NA	NA	NA	0,00414641
ILMN_1713529	SEMA6A	NA	NA	NA	NA	0,005697788	NA	NA	NA	NA	0,00417872
ILMN_1695491	WDYHV1	NA	0,002801978	NA	NA	NA	NA	NA	NA	NA	0,00421588
ILMN_1727041	EWSR1	NA	NA	NA	NA	0,004190416	NA	NA	NA	0,009922743	0,00423189
ILMN_2356311	C21orf51	0,004959301	NA	0,007523647	NA	0,007019101	NA	NA	NA	NA	0,00423523
ILMN_1662578	C1GALT1	NA	NA	NA	NA	NA	NA	NA	2,31E-05	NA	0,00425891
ILMN_1753370	ABTB2	NA	NA	NA	NA	NA	NA	NA	NA	NA	0,00426633
ILMN_2082209	C20orf100	NA	NA	NA	NA	NA	NA	NA	0,006444769	NA	0,00427042
ILMN_1802023	OR7E156P	NA	NA	NA	NA	NA	NA	NA	NA	NA	0,00433191
ILMN_1748840	CALB2	NA	9,96E-05	NA	NA	NA	NA	0,001094961	NA	NA	0,0043454
ILMN_1667430	DEGS1	NA	NA	NA	NA	NA	NA	NA	NA	NA	0,00445711
ILMN_1738657	SATB2	NA	NA	NA	NA	NA	NA	NA	9,73E-05	NA	0,00446262
ILMN_3250032	XPR1	NA	NA	NA	NA	0,001321372	NA	NA	NA	NA	0,00448649
ILMN_2222750	RPL23AP7	NA	NA	NA	NA	NA	NA	NA	NA	0,005530199	0,00448809
ILMN_1756402	TMEM177	NA	NA	NA	NA	NA	NA	NA	NA	NA	0,00449181
ILMN_1763091	C14orf43	NA	0,009436851	NA	NA	NA	NA	NA	NA	NA	0,00449628
ILMN_1687824	SEPHS2	NA	NA	NA	NA	NA	NA	NA	0,007105348	NA	0,00451444
ILMN_1708016	C20orf108	NA	NA	NA	NA	0,009191814	NA	NA	NA	NA	0,0045391
ILMN_2367469	CARS	NA	NA	NA	NA	NA	NA	NA	NA	NA	0,00455459
ILMN_1796339	PLEKHA2	NA	NA	NA	NA	NA	NA	NA	NA	NA	0,00458988
ILMN_1768553	LOC283050	NA	NA	NA	NA	0,008636488	NA	NA	NA	NA	0,00459775
ILMN_1715482	ULK2	NA	NA	NA	NA	NA	NA	NA	NA	NA	0,0046034
ILMN_2089977	FKBP9L	NA	NA	NA	NA	0,005620288	NA	NA	NA	NA	0,00461323
ILMN_1749641	FBXO3	NA	NA	NA	NA	NA	NA	NA	NA	NA	0,00463052
ILMN_1659156	ANKRD16	NA	NA	NA	NA	NA	NA	NA	NA	NA	0,00465067
ILMN_1786707	C19orf63	NA	0,004205203	NA	NA	NA	NA	NA	NA	NA	0,00465624
ILMN_3238680	C7orf55	0,003361183	0,006050937	0,007120505	NA	NA	NA	NA	NA	NA	0,00467512
ILMN_1743034	KIF1B	NA	NA	NA	NA	NA	NA	NA	NA	NA	0,0046943
ILMN_1765076	APPL2	NA	0,006657319	NA	NA	NA	NA	NA	NA	NA	0,00469593
ILMN_1768732	SPAG16	NA	0,001425587	NA	NA	NA	NA	NA	NA	NA	0,00473221
ILMN_2343097	NCALD	NA	NA	NA	NA	NA	NA	NA	0,002998201	NA	0,00475953
ILMN_1773337	DKK1	NA	NA	NA	NA	NA	NA	NA	NA	NA	0,00476241
ILMN_2392189	CTDSPL	NA	NA	NA	NA	NA	NA	NA	NA	NA	0,00476822
ILMN_2091792	ENTPD6	NA	0,002740097	NA	NA	NA	NA	NA	0,003817608	NA	0,00478935
ILMN_1664920	C19orf12	NA	NA	NA	NA	NA	NA	NA	NA	NA	0,0048257
ILMN_1686116	THBS1	NA	NA	NA	NA	NA	NA	NA	NA	0,003953817	0,00482892
ILMN_1730940	KLHDC3	NA	NA	NA	NA	NA	NA	NA	NA	NA	0,00496032
ILMN_2115991	TNNT1	0,007387721	NA	NA	NA	NA	NA	NA	NA	NA	0,00498056
ILMN_3249608	ANKLE2	NA	0,004522978	NA	NA	NA	NA	NA	NA	NA	0,00498373
ILMN_1765779	ADAM17	NA	NA	NA	NA	0,006825551	0,007110424	NA	NA	NA	0,00499257
ILMN_1753782	ZNF266	NA	NA	0,006499748	NA	0,002761811	NA	NA	NA	NA	0,0049949
ILMN_1810085	ABCF3	NA	NA	NA	NA	NA	NA	NA	NA	NA	0,00499964
ILMN_1699208	NAP1L1	NA	NA	NA	NA	NA	NA	NA	0,007044817	NA	0,0050129
ILMN_1688103	CTNNBIP1	NA	0,002663035	NA	NA	NA	NA	NA	0,005828244	NA	0,0050294
ILMN_1754988	N6AMT1	NA	NA	NA	NA	NA	NA	NA	NA	NA	0,00506817
ILMN_1722532	JMJD1A	NA	NA	NA	NA	NA	NA	NA	NA	NA	0,00508313
ILMN_1787815	TRIB3	NA	NA	NA	NA	NA	NA	NA	NA	NA	0,00508484
ILMN_1813246	LOC728554	NA	NA	NA	NA	NA	NA	NA	NA	NA	0,00509828
ILMN_2211122	TRAPPC2P1	0,001265297	NA	0,003166442	NA	0,007990701	NA	NA	NA	NA	0,00511597
ILMN_1778985	PRMT10	NA	NA	NA	NA	NA	NA	NA	NA	NA	0,0051671
ILMN_1715674	ITPK1	NA	NA	NA	NA	NA	NA	NA	0,000193992	NA	0,00517081
ILMN_1737663	C7orf13	NA	0,007636751	NA	NA	NA	NA	NA	NA	0,000566877	0,00517534
ILMN_1698766	PYCARD	NA	NA	NA	NA	NA	NA	NA	NA	NA	0,00518932
ILMN_2269564	ARID4B	NA	NA	NA	NA	NA	NA	NA	NA	0,009375101	0,0052023
ILMN_1795428	WDR59	NA	NA	NA	NA	NA	NA	NA	9,12E-06	NA	0,00521563
ILMN_1729281	SPHK2	NA	NA	NA	NA	NA	NA	NA	NA	NA	0,00521733
ILMN_1802843	PRCC	NA	NA	NA	NA	NA	NA	NA	NA	NA	0,00522843
ILMN_1708881	RAB20	NA	NA	NA	NA	NA	NA	NA	NA	NA	0,00523883
ILMN_3248635	LOC1001337	NA	NA	NA	NA	NA	NA	NA	NA	NA	0,00532785
ILMN_1738401	FOXC1	NA	NA	NA	NA	NA	NA	NA	0,001445165	NA	0,0053549
ILMN_1750088	VRK2	NA	NA	NA	NA	NA	NA	NA	NA	NA	0,00541547
ILMN_1687440	HIPK2	NA	NA	NA	NA	NA	NA	NA	0,008528827	NA	0,00543864
ILMN_1749892	EGLN1	NA	NA	NA	NA	NA	NA	NA	NA	NA	0,00545022
ILMN_3200652	LOC1001313	NA	NA	7,89E-05	NA	5,63E-05	NA	NA	NA	NA	0,00546243
ILMN_1731418	SP110	NA	NA	NA	NA	NA	NA	NA	NA	NA	0,00547449
ILMN_1719343	WDR26	NA	NA	NA	NA	NA	NA	NA	NA	NA	0,00549414
ILMN_1730575	GCLC	NA	NA	NA	NA	NA	NA	NA	NA	NA	0,00549586
ILMN_1721128	TOMM34	NA	NA	NA	NA	NA	NA	NA	NA	NA	0,00552588
ILMN_2386818	URG4	NA	NA	NA	NA	NA	NA	NA	0,002815037	NA	0,00553203
ILMN_1748884	TOB2	NA	NA	NA	NA	NA	NA	NA	NA	NA	0,00554919
ILMN_2072296	CKS2	NA	0,006389575	NA	NA	NA	NA	NA	NA	NA	0,00558034
ILMN_1766010	YARS	NA	NA	NA	NA	NA	NA	NA	NA	NA	0,00562098
ILMN_1723235	DUS3L	NA	NA	NA	NA	NA	NA	NA	NA	NA	0,00577318
ILMN_3235379	LOC1001342	NA	NA	NA	NA	NA	NA	NA	NA	NA	0,00580762
ILMN_3209631	LOC728060	0,001234278	NA	0,000173693	NA	0,000142657	NA	NA	NA	NA	0,00584561
ILMN_1680874	TUBB2B	NA	NA	NA	NA	NA	NA	NA	NA	NA	0,0058966
ILMN_2121282	MRPS18B	NA	NA	NA	NA	NA	NA	NA	NA	NA	0,00594223
ILMN_1790472	SLC25A28	NA	NA	NA	NA	NA	NA	NA	2,90E-06	NA	0,00595036
ILMN_1705953	LONRF1	NA	NA	NA	NA	NA	NA	NA	NA	0,004936126	0,00595239
ILMN_1810037	RUSC2	NA	NA	NA	NA	NA	NA	NA	NA	NA	0,00597165
ILMN_1753249	DDX10	NA	0,005861596	NA	NA	NA	NA	NA	NA	NA	0,00600577
ILMN_2388975	CERK	NA	NA	NA	NA	NA	NA	NA	NA	NA	0,00601376
ILMN_1799028	TSPAN5	NA	NA	NA	NA	NA	NA	NA	NA	0,000973726	0,0060185
ILMN_1667561	IFRD1	NA	NA	NA	NA	NA	NA	NA	NA	NA	0,00603606
ILMN_1711899	ANXA2	NA	NA	NA	NA	NA	NA	NA	NA	NA	0,00604243

ILMN_1700546	ELOVL6	NA	NA	NA	NA	NA	NA	NA	0,000853116	NA	0,00607815
ILMN_1656625	RPS24	NA	NA	NA	NA	0,005384334	NA	NA	NA	NA	0,006084
ILMN_1758337	ZNF213	NA	NA	NA	NA	NA	NA	NA	NA	NA	0,0060961
ILMN_1703593	BAIAP2L1	NA	NA	NA	NA	NA	NA	NA	NA	NA	0,0061272
ILMN_1773964	H1FX	NA	NA	0,00779352	NA	NA	NA	NA	NA	NA	0,00612881
ILMN_3241237	LOC1001325	NA	NA	NA	NA	NA	NA	NA	NA	NA	0,00614552
ILMN_1666364	COQ10A	NA	NA	NA	NA	NA	NA	NA	NA	NA	0,00615061
ILMN_2211583	KIAA1279	NA	NA	NA	NA	NA	NA	NA	NA	NA	0,0061553
ILMN_1668179	HNRNPF	NA	NA	NA	NA	NA	NA	NA	0,005472888	NA	0,00615831
ILMN_1771286	LOC653513	NA	4,67E-05	NA	NA	NA	NA	NA	NA	NA	0,00618795
ILMN_3245507	LOC1001336	NA	NA	NA	NA	NA	NA	NA	NA	NA	0,00621326
ILMN_1810058	RABL2B	NA	NA	NA	NA	NA	NA	NA	NA	NA	0,00622623
ILMN_1745811	TDRD3	NA	NA	0,003190507	NA	0,000568675	NA	NA	NA	NA	0,00632562
ILMN_1652309	TTC8	NA	NA	0,008436324	NA	0,000374431	NA	NA	NA	NA	0,00633482
ILMN_1759729	NDUFA8	0,006814886	0,009051458	NA	NA	NA	NA	NA	NA	0,004602251	0,00634145
ILMN_1738237	HS1BP3	NA	NA	NA	NA	NA	NA	NA	NA	NA	0,0063651
ILMN_2214955	CCDC127	NA	0,000745682	NA	NA	NA	NA	NA	NA	NA	0,00641411
ILMN_1813669	ANKS1A	NA	NA	NA	NA	NA	NA	NA	0,00104614	NA	0,00654304
ILMN_1729216	CRYAB	NA	NA	NA	NA	0,004327469	NA	NA	NA	NA	0,00656402
ILMN_2085722	ING2	NA	NA	NA	NA	NA	NA	NA	NA	NA	0,00660067
ILMN_1678757	BCYRN1	NA	NA	NA	NA	NA	NA	NA	NA	NA	0,00661619
ILMN_1750273	RPL23AP7	NA	NA	NA	NA	NA	NA	NA	0,006042759	NA	0,00665013
ILMN_2388701	ST3GAL5	NA	NA	0,001541179	NA	0,006802225	NA	NA	NA	0,008492372	0,00665559
ILMN_1700896	SAP30	NA	NA	NA	NA	NA	NA	NA	NA	NA	0,00668193
ILMN_1778360	PYGB	NA	NA	NA	NA	NA	NA	NA	NA	NA	0,00673089
ILMN_3209676	LOC644315	NA	NA	NA	NA	NA	NA	NA	NA	NA	0,0067532
ILMN_1735415	NUDT16L1	NA	NA	NA	NA	NA	NA	NA	NA	NA	0,00676255
ILMN_3235221	LOC644936	NA	0,000297594	0,003185443	NA	0,002559349	NA	NA	NA	NA	0,00680214
ILMN_1770667	HECA	NA	NA	NA	NA	NA	NA	NA	NA	NA	0,00680413
ILMN_1761721	VPS35	NA	NA	NA	NA	NA	NA	NA	0,008074967	NA	0,00681425
ILMN_1737965	ELOVL4	NA	NA	0,008441525	NA	NA	NA	NA	NA	0,004734008	0,00683477
ILMN_1700031	PRAME	NA	NA	NA	NA	NA	NA	NA	NA	NA	0,00690163
ILMN_1786050	RBBP9	NA	NA	NA	NA	0,009272534	NA	NA	NA	NA	0,0069092
ILMN_1730888	ZNF680	NA	NA	NA	NA	NA	NA	NA	NA	NA	0,00691242
ILMN_1652215	LOC644310	NA	NA	NA	NA	NA	NA	NA	NA	NA	0,0069201
ILMN_2147306	PNRC2	NA	NA	NA	NA	NA	NA	NA	NA	NA	0,0069231
ILMN_1755115	RPL23	NA	NA	NA	NA	NA	NA	NA	NA	NA	0,00693097
ILMN_1767894	POLB	NA	0,009945304	NA	NA	NA	NA	NA	NA	NA	0,00696268
ILMN_1778691	TIA1	NA	NA	NA	NA	NA	NA	NA	NA	NA	0,00696365
ILMN_1782897	CAPRIN1	NA	NA	NA	NA	NA	NA	NA	0,005434571	NA	0,00699466
ILMN_1677814	ABCC3	NA	0,001917233	NA	NA	NA	NA	NA	NA	NA	0,00701244
ILMN_1655915	MMP11	NA	NA	NA	NA	NA	NA	NA	NA	NA	0,00702575
ILMN_1787324	C16orf48	NA	0,009954517	NA	NA	NA	NA	NA	NA	NA	0,00710042
ILMN_1697448	TXNIP	NA	0,007273046	0,008135947	NA	NA	NA	NA	NA	NA	0,00710779
ILMN_1805863	WDR81	NA	NA	NA	NA	NA	NA	NA	NA	NA	0,00716056
ILMN_1725193	IGFBP2	NA	0,000366112	NA	NA	0,007465324	NA	NA	NA	NA	0,00719167
ILMN_1798014	EIF2S2	NA	NA	NA	NA	NA	NA	NA	0,000533775	NA	0,00722734
ILMN_1700811	UBFD1	NA	NA	NA	NA	NA	NA	NA	NA	NA	0,0072439
ILMN_3305508	LOC728779	NA	NA	0,001203993	NA	1,27E-05	NA	NA	NA	NA	0,00725621
ILMN_1654563	EFNB1	NA	NA	NA	NA	NA	NA	NA	NA	NA	0,00730977
ILMN_1787378	ADD3	NA	0,001208097	NA	NA	NA	NA	NA	NA	NA	0,00732452
ILMN_1804454	FAM135A	NA	NA	NA	NA	0,004760628	NA	NA	NA	NA	0,00735332
ILMN_1695945	MEIS2	NA	NA	NA	NA	0,008026702	NA	NA	NA	0,005002187	0,00741953
ILMN_2179397	TATDN1	NA	NA	NA	NA	NA	NA	NA	0,009288694	NA	0,00743756
ILMN_1744959	NFX1	NA	NA	NA	NA	NA	NA	NA	NA	NA	0,00755042
ILMN_1698225	MYO5A	NA	NA	NA	NA	NA	NA	NA	NA	NA	0,00756593
ILMN_1710752	NAPRT1	NA	0,000126595	NA	NA	NA	NA	NA	NA	NA	0,00762375
ILMN_1743303	TTC1	0,00334397	NA	0,000248697	NA	0,005377057	NA	NA	NA	NA	0,00765249
ILMN_1741594	CLCN6	NA	NA	NA	NA	0,008507272	NA	NA	NA	NA	0,00767349
ILMN_2342174	WASF1	NA	NA	NA	NA	NA	NA	NA	0,003020303	NA	0,00772407
ILMN_1806266	RAP1GDS1	NA	0,003265621	NA	NA	NA	NA	NA	NA	NA	0,00781439
ILMN_2225608	FAM135A	NA	NA	NA	NA	NA	NA	NA	NA	NA	0,00788022
ILMN_1785926	ZNF621	NA	NA	NA	NA	0,004988475	NA	NA	NA	NA	0,00788396
ILMN_1658071	ATP1B1	NA	NA	NA	NA	0,005862956	NA	NA	NA	NA	0,00788449
ILMN_2358457	ATF4	NA	NA	0,009063039	NA	NA	NA	NA	NA	NA	0,00792285
ILMN_1718934	LOC728499	NA	NA	0,007565213	NA	0,001013584	NA	NA	NA	NA	0,0079643
ILMN_2337551	HIPK3	NA	NA	NA	NA	0,001591674	NA	NA	NA	NA	0,00797951
ILMN_1685480	TARS	NA	NA	NA	NA	NA	NA	NA	NA	NA	0,00799058
ILMN_1681855	TBC1D25	NA	NA	NA	NA	NA	NA	NA	NA	NA	0,00799439
ILMN_1748427	ZNF239	NA	NA	NA	NA	NA	NA	NA	NA	NA	0,00799801
ILMN_1777325	STAT1	NA	NA	NA	NA	NA	NA	NA	NA	NA	0,0080125
ILMN_1781472	CDC42BPA	NA	NA	NA	NA	NA	NA	NA	NA	NA	0,00802844
ILMN_1814315	PBXIP1	NA	NA	0,006119686	NA	0,000396863	NA	NA	NA	NA	0,00803182
ILMN_1727361	LEMD3	NA	NA	NA	NA	NA	NA	NA	NA	NA	0,00804282
ILMN_1657058	PPIL6	NA	0,006446628	NA	NA	NA	NA	NA	0,007217435	NA	0,00805895
ILMN_1651692	STK10	NA	NA	NA	NA	NA	NA	NA	NA	0,00735184	0,00810435
ILMN_1697000	KIAA0258	NA	NA	NA	NA	NA	NA	NA	NA	NA	0,00815344
ILMN_1772845	SNRNP35	NA	NA	NA	NA	NA	NA	NA	NA	NA	0,00816963
ILMN_1656482	OSBPL2	NA	NA	NA	NA	NA	NA	NA	NA	NA	0,00829151
ILMN_1694877	CASP6	NA	NA	NA	NA	NA	NA	NA	NA	NA	0,00831887
ILMN_1699472	ST3GAL3	NA	NA	NA	NA	NA	NA	NA	NA	NA	0,00836542
ILMN_2065606	TOMM40L	NA	NA	NA	NA	NA	NA	NA	0,003978852	NA	0,00838115
ILMN_1811624	THADA	NA	NA	NA	NA	NA	NA	NA	NA	NA	0,00839289
ILMN_1794552	GAP43	NA	NA	NA	NA	NA	NA	NA	NA	NA	0,00842713
ILMN_2353358	LGALS8	NA	0,001075557	NA	NA	NA	NA	0,009848402	NA	NA	0,00844276
ILMN_1742044	GNAI1	NA	NA	NA	NA	NA	NA	NA	NA	NA	0,00855884
ILMN_3237627	LOC154761	NA	NA	NA	NA	NA	NA	NA	NA	0,006062079	0,00859417
ILMN_1780444	ARL3	NA	NA	NA	NA	NA	NA	NA	NA	NA	0,00859887
ILMN_2385688	RABL2B	NA	NA	NA	NA	NA	NA	NA	NA	NA	0,00863046
ILMN_2277523	DIP2A	NA	NA	0,001516346	NA	0,007768253	NA	NA	NA	NA	0,00863163
ILMN_1679209	HSPA9	NA	NA	NA	NA	NA	NA	NA	0,002334808	NA	0,00864454
ILMN_1745887	FBXO21	NA	NA	NA	NA	NA	NA	NA	NA	NA	0,00865503
ILMN_1803279	TMED5	NA	NA	NA	NA	NA	NA	NA	0,002268072	NA	0,00871066
ILMN_1813314	HIST1H2BK	NA	0,000608744	NA	NA	NA	NA	NA	NA	NA	0,00871666
ILMN_2053490	FAM53B	NA	NA	NA	NA	NA	NA	NA	NA	NA	0,00879276
ILMN_1680727	GLRX2	NA	NA	NA	NA	NA	NA	NA	NA	NA	0,00879512
ILMN_1794074	MXI1	NA	NA	NA	NA	NA	NA	NA	NA	NA	0,00882871
ILMN_1810289	FER1L3	NA	0,004978082	NA	NA	NA	NA	NA	0,002062026	NA	0,00887723
ILMN_1742569	PAPD1	0,003423843	2,57E-05	NA	NA	NA	NA	NA	NA	NA	0,00888316
ILMN_2288928	PPP4R4	NA	NA	NA	NA	NA	NA	NA	0,000515913	NA	0,0089725
ILMN_1678323	AASS	NA	NA	NA	NA	0,009988964	NA	NA	NA	NA	0,00907186
ILMN_1695792	CUL4A	0,001119444	NA	0,00141684	NA	0,005526682	NA	NA	NA	0,007250493	0,00908512
ILMN_1787750	CD200	NA	NA	0,004656263	NA	0,003803719	NA	NA	NA	NA	0,00910863
ILMN_1666453	STK3	NA	NA	0,007055572	NA	NA	NA	NA	NA	NA	0,00914847
ILMN_2334042	THYN1	NA	NA	NA	NA	NA	NA	NA	NA	NA	0,00915433
ILMN_1690807	MKL2	NA	0,005995655	NA	NA	NA	NA	NA	NA	NA	0,0091565
ILMN_1719518	ARF4	NA	NA	NA	NA	NA	NA	NA	NA	NA	0,0091896
ILMN_2096604	NIP30	0,003590596	NA	NA	NA	NA	NA	NA	NA	NA	0,00920477
ILMN_1761797	CSTB	NA	NA	NA	NA	NA	NA	NA	NA	NA	0,00923112

ILMN_1699496	PHF21A	NA	NA	NA	NA	NA	NA	NA	NA	0,004732744	0,00926679
ILMN_1911605		NA	NA	NA	NA	NA	NA	NA	NA	NA	0,009267
ILMN_1662129	RCN2	NA	NA	NA	NA	NA	NA	NA	NA	NA	0,00938635
ILMN_1715886	CNOT7	NA	NA	NA	NA	NA	NA	NA	NA	NA	0,00941423
ILMN_1741736	DDX28	NA	NA	NA	NA	0,001169986	NA	NA	NA	NA	0,00943987
ILMN_1716766	CEBPG	NA	NA	NA	NA	NA	NA	NA	NA	NA	0,0094746
ILMN_1714445	SLC6A9	NA	NA	NA	NA	NA	NA	NA	0,000970629	NA	0,00947545
ILMN_2308689	AGBL5	NA	NA	NA	NA	0,00810324	NA	NA	NA	NA	0,00947914
ILMN_1732071	HIST2H2BE	NA	NA	NA	NA	NA	NA	NA	NA	NA	0,0094906
ILMN_2303170	MBNL3	NA	0,000616536	NA	NA	NA	NA	NA	NA	NA	0,00949375
ILMN_1793549	PTPN4	NA	NA	NA	NA	NA	NA	NA	NA	NA	0,00950889
ILMN_1731644	SETDB2	NA	NA	NA	NA	NA	NA	NA	0,000617967	NA	0,00951656
ILMN_1762899	EGR1	NA	NA	NA	NA	NA	NA	NA	0,004577391	NA	0,00959206
ILMN_1659027	SLC2A1	NA	NA	NA	NA	NA	NA	NA	NA	NA	0,00959962
ILMN_1679587	LOC651202	NA	NA	NA	NA	0,009884202	NA	NA	NA	NA	0,00962588
ILMN_3231944	LOC1001305	NA	NA	NA	NA	0,002231041	NA	NA	NA	NA	0,0096406
ILMN_1731596	AP3S2	NA	0,005377929	NA	NA	NA	NA	NA	NA	NA	0,0096805
ILMN_3246214	B3GNT1	NA	NA	NA	NA	NA	NA	NA	NA	NA	0,00968542
ILMN_1775304	DNAJB1	NA	NA	NA	NA	NA	NA	NA	NA	NA	0,00972396
ILMN_2361478	PDLIM5	NA	NA	NA	NA	NA	NA	NA	NA	0,006395242	0,00972583
ILMN_1756220	DDX18	NA	NA	NA	NA	NA	NA	NA	NA	NA	0,00975497
ILMN_1778917	CDK7	NA	NA	NA	NA	NA	NA	NA	NA	NA	0,00984232
ILMN_1730816	GPR162	NA	0,008198752	NA	NA	NA	NA	NA	NA	NA	0,00984634
ILMN_1688318	MGC72104	NA	NA	NA	NA	NA	NA	NA	NA	NA	0,00987428
ILMN_1703487	LMO4	NA	NA	NA	NA	NA	NA	NA	NA	NA	0,00989366
ILMN_3241870	FRMD8	NA	NA	NA	NA	NA	NA	NA	NA	NA	0,00992235
ILMN_1673369	SEPHS1	NA	NA	0,000614161	NA	0,000559402	NA	NA	NA	NA	0,00993548
ILMN_1723480	BST2	NA	NA	NA	NA	NA	NA	NA	0,000434502	NA	0,00993631
ILMN_1785891	PRKD1	NA	NA	NA	NA	NA	NA	NA	0,006140952	NA	0,01006695
ILMN_2133996	SHE	NA	NA	0,002716107	NA	NA	NA	NA	NA	NA	0,01006981
ILMN_2200503	NIT2	NA	NA	NA	NA	NA	NA	NA	NA	NA	0,01009315
ILMN_1806533	PDE7B	NA	0,002498535	NA	NA	NA	NA	NA	NA	NA	0,0100965
ILMN_3257145	LOC1001296	0,008388399	NA	NA	NA	NA	NA	NA	NA	NA	0,01010759
ILMN_1665117	C6orf89	NA	NA	NA	NA	NA	NA	NA	NA	NA	0,01012438
ILMN_1781010	ARHGEF3	NA	0,000157886	NA	NA	NA	NA	NA	NA	NA	0,01017666
ILMN_1741585	SFMBT1	0,00028789	0,006279188	NA	NA	NA	NA	NA	NA	0,007589847	0,01023637
ILMN_1698478	SNAPC2	NA	NA	0,001382115	NA	0,005417608	NA	NA	NA	NA	0,01024448
ILMN_1736577	ZNF688	NA	NA	NA	NA	NA	NA	NA	NA	NA	0,01026137
ILMN_1674385	YWHAQ	NA	NA	NA	NA	NA	NA	NA	NA	NA	0,01026524
ILMN_1713505	NPC1	NA	NA	NA	NA	NA	NA	NA	NA	NA	0,01027116
ILMN_1670420	METAP2	NA	NA	0,008960327	NA	0,00917744	NA	NA	NA	NA	0,01028984
ILMN_1761981	FAM96A	NA	NA	NA	NA	NA	NA	NA	NA	NA	0,01033986
ILMN_2072598	LOC401357	NA	NA	NA	NA	NA	NA	NA	NA	NA	0,01038691
ILMN_1653115	ECH1	NA	NA	0,001620501	NA	0,00066503	NA	NA	NA	NA	0,01039047
ILMN_1775965	PPP4R4	NA	NA	NA	NA	NA	NA	NA	NA	NA	0,01042797
ILMN_1653438	PHF14	NA	NA	NA	NA	NA	NA	NA	NA	NA	0,0104398
ILMN_2389810	ATP11C	NA	NA	NA	NA	0,000526037	NA	NA	NA	NA	0,01045128
ILMN_1736256	CALR	NA	0,004380848	NA	NA	NA	NA	NA	NA	NA	0,01046573
ILMN_2199947	REV3L	NA	NA	NA	NA	NA	NA	NA	NA	NA	0,01047877
ILMN_1712687	PAK2	NA	NA	NA	NA	NA	NA	NA	NA	NA	0,01049834
ILMN_2320250	NOL6	NA	NA	NA	NA	NA	NA	NA	0,007931821	NA	0,0105213
ILMN_1705397	PDK2	0,000934846	0,008828023	0,000849467	NA	0,000323923	NA	NA	NA	NA	0,01052792
ILMN_1740572	TCN2	NA	NA	0,006406372	NA	0,007286411	NA	NA	NA	NA	0,01053484
ILMN_2394557	WNK3	0,007827861	NA	0,008544362	NA	NA	NA	NA	NA	NA	0,01059693
ILMN_1789616	NUPL2	NA	NA	NA	NA	NA	NA	NA	NA	NA	0,01060113
ILMN_1695000	TFIP11	NA	NA	0,006066245	NA	0,009868887	NA	NA	NA	NA	0,01060904
ILMN_1747197	SLC41A2	NA	NA	NA	NA	NA	NA	NA	0,007771234	NA	0,01062703
ILMN_1665964	GAB2	NA	NA	NA	NA	NA	NA	NA	0,004957228	NA	0,0106315
ILMN_1835017		NA	NA	NA	NA	NA	NA	NA	NA	NA	0,01067319
ILMN_1769532	FAM86B1	NA	NA	NA	NA	NA	NA	NA	NA	NA	0,01067882
ILMN_1751692	ZNF17	NA	NA	NA	NA	0,009545909	NA	NA	NA	NA	0,01069169
ILMN_1804448	MSI2	NA	NA	NA	NA	NA	NA	NA	0,001587549	NA	0,01070663
ILMN_1740490	ZFP82	NA	NA	NA	NA	NA	NA	NA	NA	NA	0,01074781
ILMN_1715181	ARRDC2	NA	NA	0,005113702	NA	NA	NA	NA	NA	NA	0,01075816
ILMN_1667018	ACE2	NA	NA	NA	NA	NA	NA	NA	0,008057679	NA	0,01081121
ILMN_1653612	KCTD9	0,000172115	NA	0,001163604	NA	0,006699933	NA	NA	NA	0,000825574	0,01081254
ILMN_1713751	ADAM19	NA	NA	NA	NA	NA	NA	NA	0,000271283	NA	0,01081422
ILMN_1764629	SLC39A14	NA	NA	NA	NA	NA	NA	NA	NA	NA	0,01090954
ILMN_1723962	LXN	NA	NA	NA	NA	NA	NA	NA	NA	NA	0,01096017
ILMN_2140990	CAMK1	NA	0,00085912	NA	NA	NA	NA	NA	NA	NA	0,01103391
ILMN_1745954	CORO1C	NA	NA	NA	NA	NA	NA	NA	NA	NA	0,01108877
ILMN_1746403	GTF2IRD2P	NA	NA	NA	NA	NA	NA	NA	NA	NA	0,0111037
ILMN_1765446	EMP3	NA	NA	NA	NA	NA	NA	NA	NA	NA	0,01111899
ILMN_2173004	RAB8B	0,007176381	NA	0,00096268	NA	NA	NA	NA	NA	NA	0,01115281
ILMN_1757237	DDEF2	NA	NA	NA	NA	NA	NA	NA	NA	NA	0,01119348
ILMN_1652223	WDR91	NA	NA	NA	NA	NA	NA	NA	1,90E-05	NA	0,01120549
ILMN_3241250	FAM18A	NA	NA	NA	NA	NA	NA	NA	0,00540837	NA	0,01122531
ILMN_1809708	KCTD21	NA	NA	NA	NA	NA	NA	NA	NA	NA	0,01123151
ILMN_1728626	WDR5	NA	NA	NA	NA	NA	NA	NA	NA	NA	0,01123204
ILMN_1718182	THRA	NA	NA	NA	NA	0,009788426	NA	NA	NA	NA	0,01123342
ILMN_1805796	FLYWCH2	0,000579394	0,001275869	0,003633755	NA	0,006322602	NA	NA	NA	NA	0,01128537
ILMN_1764362	LYAR	NA	NA	NA	NA	NA	NA	NA	NA	NA	0,01131078
ILMN_2060770	RAI1	0,001873225	0,005715114	NA	NA	NA	NA	NA	NA	0,005655555	0,01140297
ILMN_1722781	EGR3	NA	NA	NA	NA	NA	NA	NA	NA	NA	0,0114126
ILMN_2089167	RHOD	NA	NA	NA	NA	NA	NA	NA	0,00031318	NA	0,01149224
ILMN_1784206	LOC653308	NA	0,003689951	NA	NA	NA	NA	NA	NA	NA	0,01152488
ILMN_1776102	PSMD10	NA	NA	0,009489491	NA	NA	NA	NA	NA	NA	0,01153257
ILMN_1671583	MKRN1	NA	NA	NA	NA	NA	NA	NA	NA	NA	0,01158893
ILMN_1670925	CYB5D1	NA	NA	NA	NA	NA	NA	NA	NA	NA	0,01159938
ILMN_2218648	RAG1AP1	NA	NA	NA	NA	NA	NA	NA	0,003489593	NA	0,01160403
ILMN_1712400	SERPINB6	NA	NA	NA	NA	NA	NA	NA	NA	NA	0,01162195
ILMN_1683859	SLC7A1	NA	NA	0,005454222	NA	NA	NA	NA	NA	NA	0,01164981
ILMN_1691949	LOC728554	NA	NA	NA	NA	NA	NA	NA	NA	NA	0,01167444
ILMN_1751589	NUDCD2	NA	NA	NA	NA	0,002766467	NA	NA	NA	NA	0,01173389
ILMN_1813840	AOF2	NA	NA	NA	NA	0,002128228	NA	NA	NA	NA	0,01174334
ILMN_1760049	SERGEF	NA	NA	NA	NA	NA	NA	NA	NA	NA	0,01180308
ILMN_1814194	TCF4	NA	NA	NA	NA	NA	NA	NA	0,006816401	NA	0,0118548
ILMN_1692072	LOC728006	0,009123656	NA	NA	NA	NA	NA	NA	NA	0,000352366	0,01190336
ILMN_1667081	CCND2	NA	NA	NA	NA	NA	NA	NA	0,0072066	NA	0,01192933
ILMN_1715927	VSTM2L	NA	NA	NA	NA	NA	NA	NA	NA	NA	0,01207973
ILMN_1700268	QPRT	NA	NA	NA	NA	NA	NA	NA	NA	NA	0,01209126
ILMN_1656656	COX19	NA	NA	NA	NA	NA	NA	NA	0,000112171	NA	0,01209335
ILMN_1706677	MAPK1	NA	NA	NA	NA	NA	NA	NA	NA	NA	0,01210906
ILMN_1725169	INTS12	NA	NA	NA	NA	NA	NA	NA	NA	NA	0,01211416
ILMN_2306033	PRAME	NA	NA	NA	NA	NA	NA	NA	NA	NA	0,01214947
ILMN_2189859	FLJ38482	NA	0,004079436	NA	NA	NA	NA	NA	NA	NA	0,01220273
ILMN_1691341	IL7R	NA	NA	NA	NA	NA	NA	NA	8,28E-05	NA	0,01226123
ILMN_1701507	EHHADH	NA	NA	NA	NA	NA	NA	NA	NA	NA	0,01228622
ILMN_1756590	SYS1	NA	0,004077996	NA	NA	NA	NA	NA	0,008870192	NA	0,0122905

ILMN_1685441	ASAP3	NA	NA	NA	NA	0,0083887	NA	NA	NA	NA	0,01233927
ILMN_3244963	WDR42A	0,008551164	NA	0,008393147	NA	0,009900102	NA	NA	NA	NA	0,01236874
ILMN_1729288	C1QTNF6	NA	NA	0,001368181	NA	0,001030971	NA	NA	NA	NA	0,01237988
ILMN_1706005	GALT	NA	NA	NA	NA	NA	NA	NA	NA	NA	0,01245977
ILMN_1761120	TM2D3	NA	NA	NA	NA	NA	NA	NA	NA	NA	0,01245996
ILMN_1679405	DDX56	NA	NA	NA	NA	NA	NA	NA	NA	NA	0,01246809
ILMN_2367113	CASP6	NA	NA	NA	NA	NA	NA	NA	0,001665411	NA	0,01247223
ILMN_1683234	DNAJC1	NA	NA	NA	NA	NA	NA	NA	0,003337717	NA	0,01249471
ILMN_2131467	GNAI1	NA	NA	NA	NA	NA	NA	NA	NA	NA	0,01256921
ILMN_1669972	DKFZp686O2	NA	NA	NA	NA	0,009142037	NA	NA	NA	NA	0,01269798
ILMN_1742461	UAP1	NA	NA	NA	NA	NA	NA	NA	0,005199912	NA	0,01273425
ILMN_2230035	BBS2	NA	NA	0,000209988	NA	0,003854175	NA	NA	NA	NA	0,01275399
ILMN_1695706	H3F3B	NA	NA	NA	NA	NA	NA	NA	NA	NA	0,01275788
ILMN_2077550	RACGAP1	NA	NA	0,000565325	NA	0,001157195	NA	NA	NA	NA	0,01276584
ILMN_1853876		NA	NA	NA	NA	NA	NA	NA	NA	NA	0,01277408
ILMN_1743199	EGR2	NA	NA	NA	NA	NA	NA	NA	NA	NA	0,0127904
ILMN_1672536	FBLN1	NA	NA	NA	NA	0,001450927	NA	NA	NA	NA	0,01280221
ILMN_1659766	BAG3	NA	0,000946114	NA	NA	NA	NA	NA	NA	NA	0,01289564
ILMN_1685540	SHRM	NA	NA	NA	NA	NA	NA	NA	NA	NA	0,01290576
ILMN_1811551	DERA	NA	NA	NA	NA	NA	NA	NA	0,003301806	NA	0,01290793
ILMN_2415144	SP110	NA	NA	NA	NA	NA	NA	NA	NA	NA	0,01295012
ILMN_2372136	P4HTM	NA	0,002753469	NA	NA	NA	NA	NA	NA	NA	0,0129702
ILMN_2083833	CNOT6L	NA	NA	NA	NA	NA	NA	NA	NA	NA	0,01299253
ILMN_2213297	C11orf54	NA	0,009133012	NA	NA	NA	NA	NA	NA	NA	0,0130236
ILMN_1791006	AHI1	NA	NA	NA	NA	NA	NA	NA	0,001709014	NA	0,01302989
ILMN_1691410	BAMBI	NA	NA	NA	NA	NA	NA	NA	NA	NA	0,01303201
ILMN_1769926	DBN1	NA	0,006901069	NA	NA	NA	NA	NA	NA	NA	0,01303792
ILMN_1813701	RASAL2	NA	0,007044119	NA	NA	NA	NA	NA	NA	NA	0,01305237
ILMN_1751607	FOSB	0,004658131	NA	NA	NA	NA	NA	NA	NA	NA	0,0130554
ILMN_3188005	LOC1001279	NA	NA	NA	NA	NA	NA	NA	NA	NA	0,01307087
ILMN_1718924	ETFA	NA	NA	NA	NA	NA	NA	NA	NA	NA	0,01308722
ILMN_1653203	EFEMP2	NA	0,00283678	NA	NA	NA	NA	NA	NA	NA	0,01310126
ILMN_2396272	PDCD4	NA	NA	NA	NA	NA	NA	NA	NA	NA	0,0131241
ILMN_1665235	CRTAP	NA	NA	NA	NA	NA	NA	NA	NA	NA	0,0131555
ILMN_1751851	CECR1	6,61E-05	NA	NA	NA	NA	NA	NA	NA	0,000566736	0,01317336
ILMN_1715684	LAMB3	0,002231791	NA	0,003879309	NA	0,009000048	NA	NA	NA	NA	0,01319007
ILMN_1703337	LOC441763	NA	NA	NA	NA	NA	NA	NA	NA	NA	0,0133014
ILMN_2209299	ZNF333	NA	NA	NA	NA	NA	NA	NA	NA	NA	0,01335195
ILMN_1770085	BTG2	NA	NA	NA	NA	NA	NA	NA	NA	NA	0,01342363
ILMN_2275502	RAPH1	NA	NA	NA	NA	NA	NA	NA	NA	NA	0,01345603
ILMN_1735155	GLB1	NA	NA	NA	NA	0,008192127	NA	NA	NA	NA	0,01348621
ILMN_3251269	SRP14	NA	NA	NA	NA	NA	NA	NA	NA	NA	0,01352863
ILMN_3249419	LOC728457	NA	NA	0,004606054	NA	0,002342137	NA	NA	NA	NA	0,01356999
ILMN_1812721	LOC728014	NA	0,005558995	NA	NA	0,004422381	NA	NA	NA	NA	0,01357407
ILMN_1670218	EXOSC6	NA	NA	NA	NA	NA	NA	NA	0,003680634	NA	0,01357472
ILMN_1685088	RPL36	NA	NA	NA	NA	NA	NA	NA	NA	NA	0,01360114
ILMN_1701434	RAP1B	NA	NA	NA	NA	NA	NA	NA	NA	NA	0,01361008
ILMN_2356895	MRPL42	NA	NA	NA	NA	NA	NA	NA	NA	NA	0,01361479
ILMN_1737949	GALNS	NA	NA	NA	NA	NA	NA	NA	NA	NA	0,0136401
ILMN_3294033	LOC339970	NA	0,007111116	NA	NA	NA	NA	NA	NA	NA	0,01365302
ILMN_1807719	CTNS	NA	NA	NA	NA	NA	NA	NA	NA	NA	0,0136572
ILMN_1679322	SH2D4A	NA	NA	NA	NA	NA	NA	NA	NA	NA	0,01366498
ILMN_1753164	IPO8	NA	NA	NA	NA	NA	NA	NA	NA	NA	0,01367193
ILMN_2331163	CUL4A	NA	NA	NA	NA	NA	NA	NA	NA	0,007532353	0,01368713
ILMN_1664761	TMEM138	NA	NA	NA	NA	NA	NA	NA	NA	NA	0,01368898
ILMN_1714433	MARCKSL1	NA	NA	NA	NA	NA	NA	NA	NA	NA	0,01371167
ILMN_1703123	AXUD1	NA	NA	0,00654876	NA	NA	NA	NA	NA	NA	0,01373473
ILMN_1696532	RBBP5	NA	NA	NA	NA	NA	NA	NA	NA	NA	0,01375762
ILMN_3246388	MED14	NA	NA	NA	NA	NA	NA	NA	NA	NA	0,01380516
ILMN_3292320	LOC642513	0,002213945	NA	NA	NA	NA	NA	NA	NA	0,000985038	0,01381631
ILMN_3183620	LOC1001283	NA	NA	NA	NA	NA	NA	NA	NA	NA	0,01382755
ILMN_1718285	HOXC8	NA	NA	NA	NA	NA	NA	NA	NA	NA	0,01383374
ILMN_1795419	C2orf24	0,005307007	NA	0,000391406	NA	0,004559336	NA	NA	NA	0,005284664	0,01386133
ILMN_2155025	ZNF681	NA	NA	NA	NA	NA	NA	NA	NA	NA	0,01399595
ILMN_1661940	CAMTA1	NA	NA	NA	NA	NA	NA	NA	0,000534046	NA	0,01406434
ILMN_1751598	SESN2	NA	NA	NA	NA	NA	NA	NA	NA	NA	0,0140759
ILMN_1780058	DEGS1	NA	NA	NA	NA	NA	NA	NA	NA	NA	0,01407756
ILMN_2208495	LASS5	NA	NA	0,002462108	NA	NA	NA	NA	NA	NA	0,01408969
ILMN_1711383	STK4	NA	NA	0,005726696	NA	NA	NA	NA	NA	0,004116587	0,01410106
ILMN_1688853	ZBTB9	NA	NA	NA	NA	NA	NA	NA	NA	NA	0,01416104
ILMN_1768719	RDH11	NA	NA	NA	NA	NA	NA	NA	NA	NA	0,01423806
ILMN_3278132	LOC440487	NA	NA	NA	NA	NA	NA	NA	NA	NA	0,0142386
ILMN_1682781	TEAD2	NA	NA	NA	NA	NA	NA	NA	NA	NA	0,01424193
ILMN_1775243	LOC646766	NA	NA	NA	NA	NA	NA	NA	NA	NA	0,01424585
ILMN_1900661		NA	NA	0,008273969	NA	NA	NA	NA	NA	NA	0,0142899
ILMN_1738767	PLP2	NA	NA	NA	NA	NA	NA	NA	NA	NA	0,01434712
ILMN_1771026	GARS	NA	NA	NA	NA	NA	NA	NA	NA	NA	0,01439401
ILMN_1783996	DEM1	NA	NA	NA	NA	NA	NA	NA	NA	NA	0,01446332
ILMN_1803398	SRF	NA	NA	NA	NA	NA	NA	NA	NA	NA	0,01446614
ILMN_1750101	S100A11	NA	NA	NA	NA	NA	NA	NA	NA	NA	0,01459685
ILMN_2412922	C14orf43	NA	NA	NA	NA	NA	NA	NA	NA	NA	0,01460857
ILMN_3243945	C13orf37	NA	NA	NA	NA	0,00245305	NA	NA	NA	NA	0,01464074
ILMN_1786972	SARS	0,00118389	NA	0,000384868	NA	NA	NA	NA	NA	NA	0,01466989
ILMN_3251383	CCDC74B	NA	0,00415968	NA	NA	NA	NA	NA	NA	NA	0,01467721
ILMN_1693830	LACTB	NA	NA	NA	NA	NA	NA	NA	NA	NA	0,01472824
ILMN_1774229	SLC7A4	NA	NA	NA	NA	NA	NA	NA	NA	NA	0,01474287
ILMN_1809433	XBP1	NA	NA	NA	NA	NA	NA	NA	NA	NA	0,01474484
ILMN_2059886	TTC38	NA	NA	NA	NA	NA	NA	NA	NA	NA	0,01482244
ILMN_1779492	SMURF1	NA	NA	NA	NA	NA	NA	NA	0,004634916	NA	0,01482793
ILMN_1746012	MBD6	NA	0,003332645	NA	NA	NA	NA	NA	NA	NA	0,01483058
ILMN_2376403	TSC22D3	NA	NA	0,004603813	NA	NA	NA	NA	NA	NA	0,01484675
ILMN_1675048	IGF1R	NA	NA	NA	NA	NA	NA	NA	NA	NA	0,01485547
ILMN_1762071	C17orf80	NA	NA	NA	NA	NA	NA	NA	NA	NA	0,014867
ILMN_1705570	H2AFY2	NA	NA	NA	NA	NA	NA	NA	NA	NA	0,01487258
ILMN_1774844	MAPKAPK2	NA	NA	NA	NA	NA	NA	NA	NA	NA	0,01487912
ILMN_3302919	MYOF	NA	0,007432556	NA	NA	NA	NA	NA	0,001131559	NA	0,01489939
ILMN_3236061	ZNF783	NA	NA	NA	NA	NA	NA	NA	NA	0,002907582	0,01491008
ILMN_1732328	LOC646200	NA	NA	NA	NA	NA	NA	NA	NA	NA	0,01495539
ILMN_1814077	ZFPM2	NA	0,007245403	NA	NA	NA	NA	NA	NA	NA	0,01496951
ILMN_1652280	FBXO32	NA	NA	0,009721415	NA	NA	NA	NA	NA	NA	0,01498209
ILMN_1719543	MAF	NA	0,005733116	NA	NA	NA	NA	NA	NA	NA	0,01498378
ILMN_1744822	BECN1	NA	0,001242806	NA	NA	NA	NA	NA	NA	NA	0,01498569
ILMN_3233135	FAM178A	NA	NA	NA	NA	NA	NA	NA	NA	NA	0,0149905
ILMN_2186061	PFKFB3	0,007327316	0,005347706	NA	NA	NA	NA	NA	NA	NA	0,01503656
ILMN_1719696	PLD1	NA	0,00687505	NA	NA	NA	NA	NA	NA	NA	0,01503984
ILMN_1703433	PLSCR3	NA	0,005517847	NA	NA	NA	NA	NA	NA	NA	0,01504697
ILMN_2123415	BMP8B	NA	NA	NA	NA	NA	NA	NA	NA	NA	0,01506553
ILMN_1743299	PXMP3	NA	NA	NA	NA	NA	NA	NA	NA	NA	0,01508711
ILMN_1709626	FAM14B	NA	NA	5,16E-05	NA	0,000842835	NA	NA	NA	NA	0,01510827

ILMN_1788538	NCALD	NA	NA	NA	NA	NA	NA	NA	0,00289087	NA	0,01514382
ILMN_3237956	ZC3H12C	NA	NA	NA	NA	NA	NA	NA	NA	NA	0,01515392
ILMN_1661594	C2orf42	NA	NA	0,004360745	NA	NA	NA	NA	NA	0,000843522	0,01517058
ILMN_3242883	AGAP8	NA	NA	NA	NA	0,000446382	NA	NA	NA	NA	0,01519358
ILMN_1773850	FXC1	NA	NA	NA	NA	NA	NA	NA	NA	NA	0,01524904
ILMN_2378048	HNRPK	NA	NA	NA	NA	0,004476291	NA	NA	NA	NA	0,01528827
ILMN_1654916	KIF7	NA	NA	0,009417424	NA	0,004434615	NA	NA	NA	NA	0,01529556
ILMN_1712505	KDELC1	NA	NA	NA	NA	0,004434961	NA	NA	NA	NA	0,01530443
ILMN_1658885	DAGLB	NA	NA	NA	NA	0,003915106	NA	NA	NA	NA	0,015385
ILMN_1701877	AXL	NA	NA	NA	NA	NA	NA	NA	NA	NA	0,01539195
ILMN_1662316	VPS33A	NA	NA	NA	NA	NA	NA	NA	NA	NA	0,01539733
ILMN_1776325	UBE2Q1	0,004240675	NA	0,009406284	NA	NA	NA	NA	NA	NA	0,01543756
ILMN_1717477	PSD3	NA	0,002666323	NA	NA	NA	NA	NA	NA	NA	0,01546322
ILMN_1854469		NA	NA	NA	NA	NA	NA	NA	NA	NA	0,01549238
ILMN_2249920	FYN	0,0087607	NA	NA	NA	NA	NA	NA	NA	NA	0,01554444
ILMN_2188722	GLS	NA	NA	NA	NA	NA	NA	NA	NA	NA	0,01555384
ILMN_2384857	DHRS2	NA	NA	NA	NA	NA	NA	0,006138507	NA	NA	0,01565259
ILMN_1783846	RAPH1	NA	NA	NA	NA	NA	NA	NA	NA	NA	0,01566948
ILMN_2359887	PPP2R5D	NA	NA	NA	NA	NA	NA	NA	0,007721537	NA	0,01569104
ILMN_1731001	ERICH1	NA	NA	NA	NA	NA	NA	NA	NA	NA	0,01569737
ILMN_2347044	SLC25A14	NA	0,000615652	NA	NA	NA	NA	NA	NA	NA	0,01571374
ILMN_1751561	CAMK1D	NA	NA	NA	NA	NA	NA	NA	0,006958137	NA	0,01575535
ILMN_1670322	FCHO2	NA	NA	NA	NA	NA	NA	NA	NA	NA	0,01575966
ILMN_1801610	METRNL	NA	NA	0,008270446	NA	0,003432121	NA	NA	NA	NA	0,01577001
ILMN_2157219	AASS	NA	NA	NA	NA	NA	NA	NA	NA	NA	0,0157936
ILMN_1814213	PQLC3	NA	0,004155756	NA	NA	NA	NA	NA	NA	NA	0,01584008
ILMN_1750051	FLJ39827	NA	NA	0,005071421	NA	0,000570581	NA	NA	NA	NA	0,01585554
ILMN_1719759	TNC	NA	0,007584872	NA	NA	NA	NA	NA	NA	NA	0,01586232
ILMN_2185845	BRSK1	NA	0,004296835	NA	NA	NA	NA	NA	NA	NA	0,0158699
ILMN_2398847	ARHGAP17	NA	NA	NA	NA	NA	NA	NA	NA	NA	0,01590528
ILMN_1804148	TMED4	NA	NA	NA	NA	NA	NA	NA	NA	NA	0,01591833
ILMN_1709486	SRPX	NA	NA	NA	NA	NA	NA	NA	NA	NA	0,01595426
ILMN_1741131	CHRNB1	NA	NA	NA	NA	NA	NA	NA	NA	NA	0,01602627
ILMN_1723709	C9orf116	NA	NA	0,002148917	NA	0,000547584	NA	NA	NA	NA	0,01603355
ILMN_2342579	IL7R	NA	NA	NA	NA	NA	NA	NA	0,000105391	NA	0,01622056
ILMN_2090802	TMEM79	NA	NA	NA	NA	NA	NA	NA	NA	NA	0,01623261
ILMN_2210934	NR3C2	NA	NA	NA	NA	NA	NA	NA	NA	NA	0,01625572
ILMN_1750689	MPP5	0,008015834	0,002050434	NA	NA	NA	NA	NA	NA	0,005678381	0,01626838
ILMN_1672743	ZNF334	NA	NA	NA	NA	NA	NA	NA	NA	NA	0,01628819
ILMN_1740185	TPMT	NA	NA	NA	NA	NA	NA	NA	NA	NA	0,01629798
ILMN_1755704	PRB4	NA	NA	NA	NA	0,002944722	NA	NA	NA	NA	0,01633968
ILMN_1790757	ADSL	NA	0,002050119	NA	NA	NA	NA	NA	NA	NA	0,01637304
ILMN_1774708	ORMDL2	NA	NA	NA	NA	NA	NA	NA	NA	NA	0,01639099
ILMN_3233179	LOC728969	NA	NA	NA	NA	NA	NA	NA	0,005661909	NA	0,01640595
ILMN_1801130	STOML1	NA	NA	NA	NA	NA	NA	NA	0,000184223	NA	0,01642189
ILMN_3251620	JMY	NA	NA	NA	NA	NA	NA	NA	0,003520665	NA	0,01643756
ILMN_3244395	LOC728877	NA	NA	NA	NA	NA	NA	NA	0,000112869	NA	0,016525
ILMN_1660275	C2orf43	NA	NA	NA	NA	NA	NA	NA	NA	NA	0,01656484
ILMN_1713846	PPM1H	NA	NA	NA	NA	NA	NA	NA	0,000445229	NA	0,01658762
ILMN_1675501	SENP5	0,008316022	NA	NA	NA	NA	NA	NA	NA	NA	0,01667571
ILMN_1756079	WASF1	NA	NA	NA	NA	NA	NA	NA	NA	NA	0,01668385
ILMN_1809344	BTBD10	NA	NA	NA	NA	NA	NA	0,004864417	NA	NA	0,01668402
ILMN_1705153	NEFH	NA	NA	NA	NA	NA	NA	NA	NA	NA	0,01668634
ILMN_2248589	DHX40	NA	NA	NA	NA	NA	NA	NA	NA	NA	0,01668914
ILMN_1756898	COQ9	NA	NA	NA	NA	NA	NA	NA	NA	NA	0,01671929
ILMN_1783337	DECR2	NA	NA	NA	NA	NA	NA	NA	NA	NA	0,0167281
ILMN_1670841	CPNE1	NA	NA	NA	NA	NA	NA	NA	0,009453307	NA	0,01674453
ILMN_1751276	BDNF	NA	NA	NA	NA	NA	NA	NA	NA	NA	0,01680627
ILMN_2096985	ALDH6A1	NA	NA	NA	NA	NA	NA	NA	NA	NA	0,01681574
ILMN_1790537	C16orf75	NA	NA	NA	NA	NA	NA	NA	NA	NA	0,01688411
ILMN_1801105	PRKCD	NA	NA	NA	NA	NA	NA	NA	NA	NA	0,01694887
ILMN_1703178	SCG2	NA	NA	0,006059471	NA	NA	NA	NA	NA	NA	0,01702088
ILMN_1760575	PTP4A1	NA	NA	NA	NA	NA	NA	NA	NA	NA	0,01707778
ILMN_1809141	ING4	NA	NA	NA	NA	0,001595713	NA	NA	NA	NA	0,0170861
ILMN_1654262	ZMAT3	NA	NA	NA	NA	NA	NA	NA	NA	NA	0,01709885
ILMN_3253627	LOC1001309	NA	0,002342656	NA	NA	NA	NA	NA	NA	NA	0,01717711
ILMN_1657680	CCDC69	NA	NA	NA	NA	NA	NA	NA	NA	NA	0,01729664
ILMN_2113074	UFM1	NA	0,000275309	NA	NA	NA	NA	NA	NA	NA	0,0174174
ILMN_1799579	CCDC51	NA	NA	NA	NA	NA	NA	NA	NA	NA	0,0174289
ILMN_1776181	BIRC3	NA	NA	NA	NA	0,002175124	NA	NA	NA	NA	0,0174371
ILMN_3248521	UBXN8	NA	NA	NA	NA	NA	NA	NA	NA	NA	0,01751356
ILMN_1681283	HIF1A	NA	NA	NA	NA	NA	NA	NA	NA	NA	0,01754537
ILMN_2094106	HSD17B12	NA	NA	NA	NA	NA	NA	NA	0,003979713	NA	0,01764977
ILMN_1788283	COTL1	NA	NA	NA	NA	NA	NA	NA	NA	NA	0,01773474
ILMN_1680341	PPIE	NA	0,008142061	NA	NA	NA	NA	NA	0,002161952	NA	0,01775981
ILMN_2365479	SNX1	NA	NA	NA	NA	NA	NA	NA	NA	NA	0,0179249
ILMN_1675331	PEG3	NA	NA	NA	NA	NA	NA	NA	NA	NA	0,01800719
ILMN_1685365	ZNF773	NA	NA	NA	NA	NA	NA	NA	NA	NA	0,01813688
ILMN_1709307	GPSM1	NA	NA	NA	NA	NA	NA	NA	NA	NA	0,01816525
ILMN_1797181	LOC93622	0,005634028	0,002750899	NA	NA	NA	NA	NA	NA	NA	0,01819096
ILMN_1713636	S100A6	NA	NA	NA	NA	NA	NA	NA	NA	NA	0,01823508
ILMN_1667716	TMEM101	NA	NA	NA	NA	NA	NA	NA	NA	NA	0,01824142
ILMN_2140389	TMEM185A	NA	NA	NA	NA	NA	NA	NA	NA	NA	0,01826513
ILMN_1748124	TSC22D3	NA	NA	0,0058618	NA	NA	NA	NA	NA	NA	0,01826911
ILMN_1789243	VPS33B	NA	NA	NA	NA	NA	NA	NA	NA	NA	0,0183277
ILMN_1652082	ELF4	NA	NA	NA	NA	NA	NA	NA	NA	NA	0,01840799
ILMN_1801387	YEATS4	NA	NA	NA	NA	NA	NA	NA	NA	NA	0,01841164
ILMN_2231021	TMEM185B	NA	NA	0,002112843	NA	NA	NA	NA	NA	NA	0,01858637
ILMN_1692121	USO1	NA	NA	NA	NA	NA	NA	NA	NA	NA	0,01858893
ILMN_1710962	TMEM97	NA	NA	NA	NA	NA	NA	NA	NA	NA	0,01859135
ILMN_1798712	USP4	NA	NA	NA	NA	NA	NA	NA	NA	NA	0,01867216
ILMN_3262936	LCLAT1	NA	NA	NA	NA	NA	NA	NA	0,009277588	NA	0,01868806
ILMN_1739199	FAM39DP	NA	NA	NA	NA	NA	NA	NA	NA	NA	0,0187134
ILMN_1759341	MAN2B1	NA	0,003280156	NA	NA	NA	NA	NA	NA	NA	0,01872917
ILMN_1730986	MALT1	NA	NA	NA	NA	NA	NA	NA	NA	NA	0,01877924
ILMN_1800796	RPL37	NA	0,008461204	NA	NA	NA	NA	NA	NA	NA	0,01881786
ILMN_1675541	EEF1B2	NA	NA	NA	NA	0,004558805	NA	NA	NA	NA	0,01883001
ILMN_1703477	ARHGEF2	NA	NA	NA	NA	NA	NA	NA	NA	NA	0,01884349
ILMN_1778876	FAM179B	NA	NA	NA	NA	NA	NA	NA	NA	NA	0,0188709
ILMN_1721605	SMYD2	NA	NA	NA	NA	NA	NA	NA	0,000766401	NA	0,01888143
ILMN_1661409	C3orf63	NA	NA	NA	NA	NA	NA	NA	NA	NA	0,01903295
ILMN_1687896	PIK3C3	NA	NA	NA	NA	NA	NA	NA	NA	NA	0,01905086
ILMN_1740976	NONO	NA	NA	NA	NA	NA	NA	NA	NA	NA	0,01905856
ILMN_1669394	EI24	NA	NA	0,000663963	NA	0,00776428	NA	NA	NA	NA	0,01907856
ILMN_2097858	KIAA1737	NA	NA	NA	NA	0,001314463	NA	NA	NA	NA	0,01908605
ILMN_1752199	LHPP	NA	NA	NA	NA	NA	NA	NA	NA	NA	0,01910738
ILMN_1742124	KIAA1128	NA	NA	NA	NA	NA	NA	NA	NA	NA	0,01913421
ILMN_1692742	DENND3	NA	NA	NA	NA	NA	NA	NA	NA	NA	0,01914097
ILMN_1665982	AKTIP	NA	NA	NA	NA	NA	NA	NA	NA	NA	0,01916918

ILMN_1682494	RSRC1	NA	NA	NA	NA	NA	NA	NA	NA	NA	0,01923371
ILMN_1776826	FLJ32810	NA	NA	NA	NA	NA	NA	NA	NA	NA	0,01924845
ILMN_1652244	POPDC3	NA	NA	NA	NA	NA	NA	NA	NA	NA	0,01925714
ILMN_3218306	LOC439994	0,001859308	0,000569778	NA	NA	NA	NA	NA	NA	NA	0,01931221
ILMN_2066348	HERPUD2	NA	NA	NA	NA	NA	NA	NA	NA	NA	0,01945435
ILMN_1672122	P4HTM	NA	NA	NA	NA	NA	NA	NA	NA	NA	0,01946006
ILMN_1752965	GREM1	NA	NA	NA	NA	NA	NA	NA	1,69E-07	NA	0,0195084
ILMN_1832252		NA	0,003073637	NA	NA	NA	NA	NA	NA	NA	0,01955509
ILMN_2258816	SAMD3	NA	0,001414751	NA	NA	NA	NA	NA	NA	NA	0,01969577
ILMN_1770245	EPB41L5	NA	NA	NA	NA	NA	NA	NA	0,006983297	NA	0,01971333
ILMN_1745217	FLJ10081	NA	NA	0,004337913	NA	0,004892881	NA	NA	NA	NA	0,01974861
ILMN_2296036	PTPN2	NA	NA	NA	NA	NA	NA	NA	NA	NA	0,01976606
ILMN_2316540	MRPL11	NA	NA	NA	NA	NA	NA	NA	NA	NA	0,01980838
ILMN_1754738	AP1M1	NA	NA	NA	NA	NA	NA	NA	NA	NA	0,01981588
ILMN_1692464	FLJ20699	NA	NA	NA	NA	NA	NA	NA	NA	NA	0,01989417
ILMN_3245476	PHRF1	NA	NA	NA	NA	NA	NA	NA	NA	NA	0,01989952
ILMN_2174081	ZNF133	NA	NA	NA	NA	NA	NA	NA	NA	NA	0,01999989
ILMN_1773154	NFKBIA	NA	NA	NA	NA	NA	NA	NA	NA	NA	0,02001392
ILMN_2278561	RABL2A	NA	NA	NA	NA	NA	NA	NA	NA	NA	0,02003035
ILMN_1677942	SHISA4	0,002547026	0,008543768	NA	NA	NA	NA	NA	NA	NA	0,02003307
ILMN_1756311	UFSP2	NA	NA	NA	NA	NA	NA	NA	0,000425239	NA	0,02003325
ILMN_3215759	LOC441484	NA	NA	NA	NA	NA	NA	NA	NA	NA	0,02004993
ILMN_2090105	TAGLN2	NA	NA	NA	NA	NA	NA	NA	NA	NA	0,02008929
ILMN_1790014	METRNL	NA	NA	NA	NA	NA	NA	NA	NA	NA	0,02016468
ILMN_1796411	TBCCD1	NA	NA	NA	NA	NA	NA	NA	NA	NA	0,02019909
ILMN_1813544	OXCT1	NA	NA	NA	NA	NA	NA	NA	NA	NA	0,02021074
ILMN_1718718	MKKS	NA	0,00210922	NA	NA	NA	NA	NA	NA	NA	0,02026805
ILMN_2224143	MCM3	NA	NA	0,004630428	NA	0,001738035	NA	NA	NA	NA	0,02027602
ILMN_1722294	CPNE8	NA	NA	NA	NA	NA	NA	NA	NA	NA	0,02027748
ILMN_1735955	LOC644033	NA	NA	NA	NA	NA	NA	NA	NA	NA	0,02035436
ILMN_1808301	MRPL45	NA	NA	NA	NA	NA	NA	NA	0,000392801	NA	0,02038241
ILMN_2214910	EPHB4	NA	NA	0,001328111	NA	0,001099595	NA	NA	NA	NA	0,02043295
ILMN_1666853	LOC644482	NA	NA	NA	NA	0,006996561	NA	NA	NA	NA	0,02044741
ILMN_2189222	KLHL8	NA	NA	NA	NA	NA	NA	NA	NA	NA	0,020459
ILMN_1808354	SLC4A7	NA	NA	NA	NA	0,009091887	NA	NA	NA	NA	0,02048463
ILMN_2309926	ZDHHC14	NA	NA	NA	NA	NA	NA	NA	NA	NA	0,02049906
ILMN_3282292	LOC646316	NA	NA	NA	NA	NA	NA	NA	NA	NA	0,02051342
ILMN_1743021	CAMKK2	NA	NA	NA	NA	NA	NA	NA	NA	NA	0,02055822
ILMN_1681812	HIF1AN	NA	NA	NA	NA	NA	NA	NA	NA	NA	0,02056339
ILMN_1808634	TMEM77	NA	NA	NA	NA	NA	NA	NA	0,000256507	NA	0,02056837
ILMN_1751452	NDFIP1	NA	NA	NA	NA	NA	NA	NA	NA	NA	0,02058818
ILMN_1758623	HIST1H2BD	0,009851771	0,004463484	NA	NA	NA	NA	NA	NA	NA	0,02059456
ILMN_1725889	AFF2	NA	NA	NA	NA	NA	NA	NA	NA	NA	0,02061222
ILMN_3189585	FLJ31306	NA	NA	NA	NA	NA	NA	NA	NA	NA	0,02065733
ILMN_1727080	MYO6	NA	NA	NA	NA	NA	NA	NA	0,006588643	NA	0,02073905
ILMN_1811650	DUS2L	NA	NA	NA	NA	NA	NA	NA	NA	NA	0,02074758
ILMN_1695853	CLK4	NA	NA	NA	NA	NA	NA	NA	NA	NA	0,02079314
ILMN_1743770	SLC25A14	NA	0,003416475	NA	NA	NA	NA	NA	NA	NA	0,02083529
ILMN_1683179	RRAD	NA	NA	NA	NA	NA	NA	NA	NA	NA	0,02085707
ILMN_2387865	KCNIP4	NA	0,006572904	NA	NA	0,0086868	NA	NA	NA	NA	0,02091092
ILMN_1759008	ZNF689	NA	NA	NA	NA	NA	NA	NA	NA	NA	0,02103153
ILMN_1672128	ATF4	NA	NA	NA	NA	NA	NA	NA	NA	NA	0,02104229
ILMN_1660847	PFKFB3	0,005518854	NA	NA	NA	NA	NA	NA	NA	NA	0,02105905
ILMN_2354381	PON2	NA	NA	NA	NA	0,007437154	NA	NA	NA	NA	0,02109064
ILMN_1705390	KLHL22	NA	NA	NA	NA	NA	NA	NA	NA	NA	0,0211236
ILMN_1729980	RNF216	NA	NA	NA	NA	NA	NA	NA	0,009915716	NA	0,021203
ILMN_2359456	ERGIC3	NA	NA	NA	NA	NA	NA	NA	NA	NA	0,02121507
ILMN_1798485	ATP6V1E1	NA	NA	NA	NA	NA	NA	NA	0,002132077	NA	0,02123668
ILMN_3274904	LOC284230	0,002629287	NA	NA	NA	NA	NA	NA	NA	0,008838961	0,02123999
ILMN_1781536	FAH	NA	NA	NA	NA	NA	NA	NA	NA	NA	0,02126311
ILMN_2362581	FNDC3A	0,002942863	0,009544594	0,002449417	NA	NA	NA	NA	NA	NA	0,02133158
ILMN_1776656	BBS7	NA	NA	NA	NA	NA	NA	NA	0,005932623	NA	0,02134228
ILMN_2393169	THOC5	NA	NA	NA	NA	NA	NA	NA	NA	NA	0,02135786
ILMN_3238845	FAM165B	NA	NA	NA	NA	NA	NA	NA	NA	NA	0,02136043
ILMN_2228044	TBC1D23	0,001264011	NA	NA	NA	NA	NA	NA	NA	0,000426373	0,02138225
ILMN_1719471	MSH3	NA	NA	NA	NA	NA	NA	NA	NA	NA	0,02142135
ILMN_3266482	LOC1001291	NA	NA	NA	NA	NA	NA	NA	NA	NA	0,02146303
ILMN_1803392	TAX1BP3	NA	NA	NA	NA	NA	NA	NA	NA	NA	0,02154951
ILMN_1688775	METRNL	NA	NA	NA	NA	0,006780334	NA	NA	NA	NA	0,02157046
ILMN_3305899	LOC728572	NA	NA	NA	NA	NA	NA	NA	NA	NA	0,0216307
ILMN_2184612	C3orf52	NA	NA	0,003184325	NA	NA	NA	NA	NA	NA	0,02163618
ILMN_2166865	ENY2	NA	0,00765961	NA	NA	NA	NA	NA	NA	NA	0,02164636
ILMN_1683447	LOC440737	NA	NA	NA	NA	NA	NA	NA	NA	NA	0,02168264
ILMN_1696066	CARS	0,005788296	NA	NA	NA	NA	NA	NA	NA	NA	0,02176172
ILMN_1724052	LOC730051	NA	NA	NA	NA	NA	NA	NA	NA	NA	0,02182519
ILMN_2333766	HISPPD2A	NA	NA	NA	NA	0,009882324	NA	NA	NA	NA	0,02187304
ILMN_1669113	ATF5	NA	7,04E-05	NA	NA	NA	NA	0,009333709	NA	NA	0,02189453
ILMN_1709894	CLPX	NA	NA	NA	NA	NA	NA	NA	NA	NA	0,0219532
ILMN_1663042	SDC4	NA	NA	NA	NA	NA	NA	NA	0,007314526	NA	0,02199106
ILMN_1713088	MSI2	NA	NA	NA	NA	NA	NA	NA	NA	NA	0,02200956
ILMN_1693452	GAL3ST4	NA	NA	NA	NA	NA	NA	NA	0,003394288	NA	0,02206154
ILMN_1692517	LOC653381	NA	NA	NA	NA	0,005303303	NA	NA	NA	NA	0,02212753
ILMN_2157277	AKR1D1	NA	NA	NA	NA	0,002084173	NA	NA	NA	NA	0,02226392
ILMN_1660111	UCHL3	NA	NA	NA	NA	NA	NA	NA	NA	NA	0,02226625
ILMN_1800317	WNT5A	NA	NA	NA	NA	0,008253585	NA	NA	NA	NA	0,02227767
ILMN_1702396	PACSIN2	NA	NA	NA	NA	NA	NA	NA	NA	NA	0,02232027
ILMN_1738642	CMPK1	NA	NA	NA	NA	NA	NA	NA	0,006237849	NA	0,02232772
ILMN_1803744	SELS	NA	NA	NA	NA	NA	NA	NA	NA	NA	0,02234768
ILMN_1736623	NCKIPSD	NA	NA	NA	NA	NA	NA	NA	NA	NA	0,02235629
ILMN_1690105	STAT1	NA	NA	NA	NA	NA	NA	NA	0,006679505	NA	0,02237525
ILMN_2387553	PSMA3	NA	NA	NA	NA	NA	NA	NA	NA	NA	0,02238429
ILMN_1815733	EIF5	NA	NA	NA	NA	NA	NA	NA	0,001425691	NA	0,02239502
ILMN_1659285	PSMG1	NA	NA	0,001684424	NA	0,003751758	NA	NA	NA	NA	0,02241106
ILMN_1726239	TBCA	NA	NA	NA	NA	NA	NA	NA	NA	NA	0,02244075
ILMN_1724718	NCK2	NA	NA	NA	NA	NA	NA	NA	NA	NA	0,02247599
ILMN_2369104	TRAPPC6B	NA	NA	NA	NA	NA	NA	NA	NA	NA	0,02247606
ILMN_1808783	STRBP	NA	NA	NA	NA	NA	NA	NA	NA	NA	0,0225704
ILMN_1768820	CYP11A1	NA	NA	NA	NA	NA	NA	NA	0,001529571	NA	0,02258589
ILMN_2415748	WSB1	NA	NA	NA	NA	NA	NA	NA	NA	NA	0,02259026
ILMN_2048591	LRRN3	NA	0,009487818	NA	NA	NA	NA	NA	NA	NA	0,02261571
ILMN_2364674	TRPT1	NA	0,005479212	NA	NA	NA	NA	NA	NA	NA	0,02267519
ILMN_1772455	HDAC3	NA	NA	NA	NA	NA	NA	NA	NA	NA	0,02271464
ILMN_2094313	ZDHHC1	NA	0,000955253	NA	NA	NA	NA	NA	NA	NA	0,02272139
ILMN_2141452	RPL18A	NA	NA	NA	NA	NA	NA	NA	NA	NA	0,02272508
ILMN_1745049	UQCR	NA	NA	NA	NA	NA	NA	NA	NA	NA	0,02273234
ILMN_1680693	ZNF419	NA	NA	0,002430332	NA	0,006832678	NA	NA	NA	NA	0,02276081
ILMN_1683415	CAMK2D	NA	NA	NA	NA	NA	NA	NA	NA	NA	0,02277197
ILMN_2104696	ERICH1	NA	NA	NA	NA	NA	NA	NA	NA	NA	0,0228313
ILMN_2302716	ALDH18A1	NA	NA	NA	NA	NA	NA	NA	7,41E-06	NA	0,0228485

ILMN_1698139	TCEA1	NA	NA	NA	NA	NA	NA	NA	NA	NA	0,02294496
ILMN_2393296	GK	NA	NA	NA	NA	NA	NA	NA	0,003254498	NA	0,02301238
ILMN_1723969	PLCB1	NA	NA	NA	NA	NA	NA	NA	NA	NA	0,02314835
ILMN_1814247	TCFL5	NA	0,000501711	NA	NA	NA	NA	NA	NA	NA	0,02318988
ILMN_1692107	SUV420H1	NA	NA	NA	NA	NA	NA	NA	NA	NA	0,02323438
ILMN_1691526	MAPKAP1	NA	0,003427184	NA	NA	NA	NA	NA	NA	NA	0,02323455
ILMN_1792265	TRIM4	NA	NA	0,004692134	NA	NA	NA	NA	NA	NA	0,02330674
ILMN_2149946	FOXG1	NA	NA	NA	NA	NA	NA	NA	NA	NA	0,02331286
ILMN_1690289	DUOX1	NA	NA	NA	NA	NA	NA	NA	NA	NA	0,02333182
ILMN_1756326	CKS2	NA	0,008984818	NA	NA	NA	NA	NA	NA	NA	0,0233419
ILMN_2162799	AHR	NA	NA	NA	NA	NA	NA	NA	NA	NA	0,0233616
ILMN_1812503	PHKA1	NA	NA	NA	NA	NA	NA	NA	NA	NA	0,02337679
ILMN_1743319	PAFAH1B2	NA	NA	NA	NA	NA	0,005178266	NA	NA	NA	0,02340586
ILMN_2096719	GRK5	NA	NA	NA	NA	NA	NA	NA	0,000167365	NA	0,02342908
ILMN_1752213	TMEM60	NA	NA	NA	NA	NA	NA	NA	NA	NA	0,02346681
ILMN_2127605	LRP3	NA	0,003193133	NA	NA	NA	NA	NA	NA	NA	0,02348144
ILMN_1677305	PVR	NA	0,00136228	NA	NA	NA	NA	NA	NA	NA	0,02359694
ILMN_1775742	RNF128	NA	NA	0,003045291	NA	NA	NA	NA	NA	NA	0,023671
ILMN_1726659	THOP1	NA	0,005251756	NA	NA	NA	NA	NA	NA	NA	0,02369794
ILMN_3235477	RRP1	NA	NA	NA	NA	NA	NA	NA	0,00634014	NA	0,02375975
ILMN_2157951	STX6	NA	NA	NA	NA	NA	NA	NA	NA	NA	0,02379583
ILMN_1815130	MICALL1	NA	NA	NA	NA	NA	NA	NA	NA	NA	0,02383856
ILMN_2355665	MTP18	NA	NA	NA	NA	NA	NA	NA	NA	NA	0,02385524
ILMN_1717154	AQR	NA	NA	NA	NA	NA	NA	NA	NA	NA	0,02385874
ILMN_1656285	METTL7A	NA	NA	NA	NA	NA	NA	NA	NA	NA	0,02388945
ILMN_2377174	SYPL1	NA	NA	NA	NA	NA	NA	NA	NA	NA	0,02407031
ILMN_1888359		NA	NA	NA	NA	NA	NA	NA	0,001421739	NA	0,02409831
ILMN_1775508	CYLD	NA	NA	NA	NA	NA	NA	NA	NA	NA	0,02422188
ILMN_1706859	C22orf32	NA	NA	NA	NA	NA	NA	NA	NA	NA	0,02428204
ILMN_1726565	PIK3R2	NA	NA	NA	NA	NA	NA	NA	0,006797017	NA	0,02432029
ILMN_2103480	ZNF320	NA	NA	NA	NA	NA	NA	NA	NA	NA	0,02439584
ILMN_1684591	ZNF434	NA	NA	NA	NA	NA	NA	NA	NA	NA	0,02441808
ILMN_1670672	TMEM37	NA	NA	NA	NA	NA	NA	NA	0,002275647	NA	0,02445266
ILMN_1675612	BLCAP	NA	0,009453939	NA	NA	NA	NA	NA	NA	NA	0,02445921
ILMN_1760338	LOC643357	NA	NA	NA	NA	0,006631513	NA	NA	NA	NA	0,02449872
ILMN_2379393	ASL	NA	NA	NA	NA	NA	NA	NA	0,001530423	NA	0,02451025
ILMN_1802631	AGA	NA	NA	NA	NA	NA	NA	NA	NA	NA	0,02451695
ILMN_1691293	RNF185	NA	NA	0,005620925	NA	NA	NA	NA	NA	NA	0,02456441
ILMN_2357361	THYN1	NA	NA	NA	NA	NA	NA	NA	NA	NA	0,02463309
ILMN_3300353	LOC729920	NA	NA	NA	NA	NA	NA	NA	NA	NA	0,02476469
ILMN_1705213	TMBIM1	NA	NA	NA	NA	NA	NA	NA	NA	NA	0,02476836
ILMN_3260618	LOC1001280	NA	NA	NA	NA	NA	NA	NA	NA	NA	0,02491541
ILMN_1800311	HSF2	NA	NA	0,000454875	NA	0,004753845	NA	NA	NA	NA	0,0249621
ILMN_2041222	FLJ40504	NA	NA	0,00942585	NA	0,004653204	NA	NA	NA	NA	0,02496907
ILMN_1802203	ARF1	NA	NA	NA	NA	NA	NA	NA	NA	NA	0,02502247
ILMN_2147114	PSPH	NA	NA	NA	NA	NA	NA	NA	NA	NA	0,02503528
ILMN_1787248	SIVA	NA	NA	NA	NA	NA	NA	NA	NA	NA	0,02514385
ILMN_1813455	SP110	NA	NA	NA	NA	NA	NA	NA	NA	NA	0,02518399
ILMN_1708081	LCLAT1	NA	NA	NA	NA	NA	NA	NA	NA	NA	0,02521617
ILMN_2262044	PARP10	NA	NA	NA	NA	NA	NA	NA	NA	NA	0,02524817
ILMN_1750409	RAB9A	NA	NA	NA	NA	NA	NA	NA	NA	NA	0,02525485
ILMN_1767337	SFXN5	NA	0,001229074	NA	NA	NA	NA	0,002167946	NA	NA	0,02525667
ILMN_1659364	RFC5	NA	NA	NA	NA	NA	NA	NA	NA	NA	0,02526098
ILMN_1719202	ZNF174	NA	NA	NA	NA	NA	NA	NA	NA	NA	0,02528412
ILMN_3178302	FNDC3B	NA	0,00056567	NA	NA	NA	NA	NA	NA	NA	0,02534036
ILMN_2368773	FAM3C	NA	NA	NA	NA	NA	NA	NA	NA	NA	0,02538369
ILMN_1770811	PELO	NA	NA	NA	NA	NA	NA	NA	NA	NA	0,02542171
ILMN_1745779	TCTEX1D2	NA	NA	NA	NA	NA	NA	NA	NA	NA	0,02548976
ILMN_1721703	PNN	NA	NA	NA	NA	NA	NA	NA	NA	NA	0,02558477
ILMN_1808110	TCEB3	NA	NA	NA	NA	NA	NA	NA	0,000721431	NA	0,0255988
ILMN_3249240	C4orf46	NA	NA	NA	NA	NA	NA	NA	NA	NA	0,02562619
ILMN_2340565	ATP2C1	NA	NA	NA	NA	NA	NA	NA	NA	NA	0,0256716
ILMN_1752914	CDGAP	NA	NA	NA	NA	NA	NA	NA	NA	NA	0,02575171
ILMN_2086077	JUNB	NA	NA	NA	NA	NA	NA	NA	NA	NA	0,02577371
ILMN_1794863	CAMK2N1	NA	0,003693792	NA	NA	NA	NA	0,002723449	NA	NA	0,02586423
ILMN_2370976	FER1L3	NA	NA	NA	NA	NA	NA	NA	0,001889417	NA	0,02586902
ILMN_2390974	DNAJB2	NA	NA	NA	NA	0,006054012	NA	NA	NA	NA	0,02594285
ILMN_1759743	SLC38A10	NA	0,001391707	NA	NA	NA	NA	NA	NA	NA	0,02595348
ILMN_1669617	GRB10	NA	NA	NA	0,00827289	NA	NA	NA	0,004099456	NA	0,02597244
ILMN_2103397	ZNF234	NA	NA	0,002229391	NA	NA	NA	NA	NA	NA	0,02597427
ILMN_1807633	HRSP12	NA	NA	NA	NA	0,008880502	NA	NA	NA	NA	0,0259995
ILMN_2375879	VEGFA	NA	NA	NA	NA	NA	NA	NA	0,000950232	NA	0,0260327
ILMN_1784352	CCM2	NA	NA	NA	NA	NA	NA	NA	NA	NA	0,02604408
ILMN_1758146	SIRPA	NA	NA	NA	NA	NA	NA	NA	NA	NA	0,02611529
ILMN_1801124	KIAA1826	NA	NA	NA	NA	NA	NA	NA	NA	NA	0,02612186
ILMN_1666096	ACSL3	NA	0,00581551	NA	NA	NA	NA	NA	NA	NA	0,02612745
ILMN_3225432	MAPKSP1	NA	NA	NA	NA	NA	NA	NA	NA	NA	0,0261383
ILMN_1862180	MGC3032	NA	NA	NA	NA	NA	NA	NA	NA	NA	0,02614504
ILMN_2358647	FBXO21	NA	NA	NA	NA	NA	NA	NA	NA	NA	0,02614666
ILMN_1801934	SERTAD3	NA	NA	NA	NA	NA	NA	NA	NA	NA	0,02615595
ILMN_1729318	TOR1AIP1	NA	NA	NA	NA	NA	NA	NA	NA	0,003997298	0,02618957
ILMN_1729294	RNF130	NA	NA	NA	NA	NA	NA	NA	NA	NA	0,02619367
ILMN_1664610	LOC402057	NA	NA	NA	NA	NA	NA	NA	NA	NA	0,02630897
ILMN_2340908	SCML1	NA	NA	0,003858409	NA	0,000678723	NA	NA	NA	NA	0,0263494
ILMN_2111187	ELOVL6	NA	NA	NA	NA	NA	NA	NA	0,001382623	NA	0,02637011
ILMN_1714158	PON2	NA	NA	NA	NA	NA	NA	NA	0,007448006	NA	0,02644596
ILMN_1691156	MT1A	NA	NA	NA	NA	0,006123153	NA	NA	NA	NA	0,02651903
ILMN_1665357	EPS15	NA	NA	NA	NA	NA	NA	NA	NA	NA	0,0266174
ILMN_1729650	PEX7	0,000473525	NA	0,002940069	NA	0,00825083	NA	NA	NA	NA	0,02668317
ILMN_1685608	NPTX2	NA	0,002125772	NA	NA	NA	NA	NA	NA	NA	0,02669411
ILMN_1679640	FXR1	NA	NA	NA	NA	NA	NA	NA	NA	NA	0,0267622
ILMN_1794512	ADPRH	NA	NA	NA	NA	NA	NA	NA	NA	NA	0,02679166
ILMN_1811921	CSRP1	NA	NA	NA	NA	NA	NA	NA	NA	NA	0,02688806
ILMN_1696654	IFIT5	NA	NA	NA	NA	NA	NA	NA	NA	NA	0,02697684
ILMN_1763036	CLCN6	NA	NA	NA	NA	NA	NA	NA	NA	NA	0,02700266
ILMN_1760360	RNF160	NA	NA	NA	NA	NA	NA	NA	NA	NA	0,02701864
ILMN_1667577	LCMT2	NA	NA	NA	NA	NA	NA	NA	NA	NA	0,02711899
ILMN_2367258	SMOX	NA	NA	NA	NA	NA	NA	NA	NA	NA	0,02714613
ILMN_2171461	ZNF322A	0,005200093	NA	0,00103649	NA	0,004559252	NA	NA	NA	NA	0,02730979
ILMN_1795906	PCBD1	NA	NA	NA	NA	NA	NA	NA	NA	NA	0,02739013
ILMN_1772888	LOC645688	NA	NA	NA	NA	NA	NA	NA	NA	NA	0,02739551
ILMN_1806809	ZNF189	NA	NA	0,000980234	NA	0,002349882	NA	NA	NA	NA	0,02744356
ILMN_3203098	LOC400836	NA	NA	NA	NA	NA	NA	NA	NA	NA	0,02746581
ILMN_1709772	SNX5	NA	NA	NA	NA	NA	NA	NA	NA	NA	0,02748676
ILMN_2055156	PAG1	NA	0,007942789	NA	NA	NA	NA	NA	0,001482432	NA	0,02752842
ILMN_1701244	ITFG2	NA	NA	NA	NA	NA	NA	NA	NA	NA	0,02754542
ILMN_2247594	RPLP1	NA	NA	NA	NA	NA	NA	NA	NA	NA	0,02755868
ILMN_2384181	DHRS9	NA	NA	NA	NA	NA	NA	NA	NA	NA	0,0275903
ILMN_1722838	MRPL46	NA	NA	NA	NA	NA	NA	NA	0,009389115	NA	0,02760259

ILMN_1809818	PRCC	NA	NA	NA	NA	NA	NA	NA	NA	NA	0,02764858
ILMN_1692056	HS3ST3A1	NA	NA	NA	NA	NA	NA	NA	NA	NA	0,02766932
ILMN_3225938	LOC729402	0,000722289	NA	NA	NA	NA	NA	NA	NA	NA	0,02770748
ILMN_2341254	STARD13	NA	NA	NA	NA	NA	NA	NA	NA	NA	0,02770822
ILMN_1705676	MRAP	NA	NA	NA	NA	NA	NA	NA	0,00240089	NA	0,02771715
ILMN_1677262	LOC388621	NA	NA	0,002961804	NA	NA	NA	NA	NA	NA	0,02773186
ILMN_1704253	C6orf106	NA	NA	NA	NA	NA	NA	NA	NA	NA	0,02774655
ILMN_1792528	LOC401206	NA	NA	NA	NA	NA	NA	NA	NA	NA	0,0277535
ILMN_1768577	PCSK6	NA	NA	NA	NA	NA	NA	NA	NA	NA	0,02775887
ILMN_1671291	EIF3I	NA	NA	NA	NA	0,001039157	NA	NA	NA	NA	0,02778167
ILMN_2147078	PIWIL4	NA	NA	NA	NA	NA	NA	NA	NA	NA	0,02784053
ILMN_1751020	PACSIN1	NA	NA	NA	NA	NA	NA	NA	0,009380776	NA	0,02789719
ILMN_2303166	MBNL3	NA	0,000698871	NA	NA	NA	NA	0,004818348	NA	NA	0,02795121
ILMN_1753482	FAM86C	NA	NA	NA	NA	NA	NA	NA	NA	NA	0,02796398
ILMN_1713189	C12orf41	NA	NA	NA	NA	0,003557995	NA	NA	NA	NA	0,02797846
ILMN_1784948	SPOCD1	NA	NA	NA	NA	NA	NA	NA	NA	NA	0,0279796
ILMN_1737580	PPPDE2	NA	NA	NA	NA	NA	NA	NA	0,001772473	NA	0,02804317
ILMN_2214098	BIVM	NA	NA	NA	NA	NA	NA	NA	NA	0,008873775	0,0280943
ILMN_1679071	MTX3	NA	NA	NA	NA	NA	NA	NA	NA	NA	0,02809916
ILMN_1740430	SLC2A4RG	NA	NA	NA	NA	NA	NA	NA	NA	NA	0,02810067
ILMN_1788468	ARGLU1	NA	NA	NA	NA	NA	NA	NA	0,000182703	NA	0,02811779
ILMN_1656915	PSD3	NA	NA	NA	NA	NA	NA	NA	NA	NA	0,02815606
ILMN_1672496	DNAJA1	NA	NA	NA	NA	NA	NA	NA	NA	NA	0,0281914
ILMN_1652185	IL4R	NA	NA	NA	NA	NA	NA	NA	0,002165458	NA	0,02821961
ILMN_2411190	SMC2	NA	NA	NA	NA	NA	NA	NA	NA	NA	0,02824872
ILMN_1692760	DPAGT1	NA	NA	NA	NA	NA	NA	NA	NA	NA	0,02829758
ILMN_3243644	LOC1001325	NA	NA	NA	NA	NA	NA	NA	NA	NA	0,02830054
ILMN_1652754	ZNF428	NA	0,001863614	NA	NA	NA	NA	NA	NA	NA	0,02831488
ILMN_2087528	CPSF3	NA	NA	0,005811611	NA	0,004829169	NA	NA	NA	NA	0,02833513
ILMN_2124802	MT1H	NA	NA	NA	NA	NA	NA	NA	0,006537157	NA	0,0283593
ILMN_1767892	DUSP12	NA	NA	NA	NA	NA	NA	NA	NA	NA	0,02842086
ILMN_1748352	CTSL2	NA	NA	NA	NA	NA	NA	NA	NA	NA	0,02844899
ILMN_1671554	LPIN1	NA	NA	NA	NA	NA	NA	NA	NA	NA	0,02848211
ILMN_1755173	PLEKHA4	NA	0,001782933	NA	NA	NA	NA	NA	NA	NA	0,02848877
ILMN_2401769	PHF14	NA	NA	NA	NA	NA	NA	NA	0,005853728	NA	0,02856217
ILMN_1719286	CTSA	NA	NA	NA	NA	NA	NA	NA	NA	NA	0,02856992
ILMN_1730809	SLC29A2	NA	NA	NA	NA	NA	NA	NA	0,003770596	NA	0,02863206
ILMN_2196569	NUP93	NA	NA	NA	NA	NA	NA	NA	NA	NA	0,02867406
ILMN_3289171	LOC1001315	NA	NA	0,002182897	NA	NA	NA	NA	NA	0,002886224	0,02869272
ILMN_3248786	LOC647588	NA	NA	NA	NA	NA	NA	NA	NA	NA	0,02869871
ILMN_1664738	C11orf54	NA	NA	NA	NA	NA	NA	NA	NA	NA	0,02870514
ILMN_1672755	LOC649548	NA	NA	0,006529023	NA	0,00398904	NA	NA	NA	NA	0,02876214
ILMN_1687922	RP9	NA	NA	NA	NA	NA	NA	NA	3,36E-05	NA	0,02879109
ILMN_1651498	GADD45G	NA	NA	NA	NA	NA	NA	NA	NA	NA	0,02881892
ILMN_1738796	PITPNC1	NA	NA	NA	NA	NA	NA	NA	NA	NA	0,0288259
ILMN_2111294	NUDT10	NA	NA	NA	NA	NA	NA	NA	NA	NA	0,02889697
ILMN_1685109	POLR3D	NA	NA	NA	NA	NA	NA	NA	0,001368847	NA	0,02891004
ILMN_2154566	RPL10A	NA	NA	NA	NA	NA	NA	NA	NA	NA	0,02892483
ILMN_3199609	LOC400013	NA	NA	0,000297401	NA	0,00457108	NA	NA	NA	NA	0,02899513
ILMN_1656292	LOC388532	NA	NA	0,003230001	NA	NA	NA	NA	NA	0,008318824	0,02901332
ILMN_1727023	AMMECR1L	NA	NA	NA	NA	NA	NA	NA	NA	NA	0,02904977
ILMN_1702691	TNFAIP3	NA	NA	NA	NA	NA	NA	NA	NA	NA	0,02917119
ILMN_1659444	KIF1B	NA	NA	NA	NA	NA	NA	NA	NA	NA	0,02923299
ILMN_1720233	CCDC49	NA	NA	NA	NA	NA	NA	NA	NA	NA	0,02926157
ILMN_1795128	C13orf23	NA	NA	NA	NA	NA	NA	NA	NA	NA	0,02932762
ILMN_1708203	OTUD4	NA	NA	NA	NA	NA	NA	NA	NA	0,009785747	0,02938935
ILMN_1882590		NA	NA	6,03E-06	NA	0,000660956	NA	NA	NA	NA	0,02951812
ILMN_1673380	GNG12	NA	NA	NA	NA	NA	NA	NA	NA	0,003067976	0,02952632
ILMN_1767612	BBS2	NA	NA	0,000102527	NA	0,001445665	NA	NA	NA	NA	0,0295489
ILMN_2408102	TFIP11	NA	NA	NA	NA	NA	NA	NA	NA	NA	0,02955832
ILMN_1691798	ZNF26	NA	NA	0,005343527	NA	NA	NA	NA	NA	NA	0,02959386
ILMN_1745994	GAS7	NA	NA	NA	NA	NA	NA	NA	NA	NA	0,02967844
ILMN_1763436	SETX	NA	NA	NA	NA	NA	NA	NA	NA	NA	0,02974824
ILMN_2129388	FAM190B	NA	NA	NA	NA	NA	NA	NA	NA	NA	0,02977786
ILMN_1694177	PCNA	NA	NA	NA	NA	NA	NA	NA	NA	NA	0,02977916
ILMN_2062001	HOOK3	NA	0,007368117	NA	NA	NA	NA	NA	NA	NA	0,02978399
ILMN_2355033	KIAA1147	NA	NA	NA	NA	NA	NA	NA	NA	NA	0,02978882
ILMN_2152465	PIP5K2A	NA	NA	NA	NA	NA	NA	NA	NA	NA	0,02983553
ILMN_1656145	GOT1	NA	NA	NA	NA	NA	NA	NA	NA	NA	0,0298386
ILMN_2150352	CBWD5	NA	NA	NA	NA	NA	NA	NA	0,001413212	NA	0,02988285
ILMN_1679262	DPYSL3	NA	NA	NA	NA	NA	NA	NA	NA	NA	0,02989008
ILMN_1689037	LIPG	NA	NA	NA	NA	NA	NA	NA	NA	NA	0,02991227
ILMN_2401927	TTC8	NA	NA	NA	NA	0,007074365	NA	NA	NA	NA	0,02997776
ILMN_3234831	LOC645296	NA	NA	NA	NA	NA	NA	NA	NA	NA	0,0300452
ILMN_2207539	RPS17	NA	NA	0,003527818	NA	NA	NA	NA	NA	NA	0,03005894
ILMN_1725183	TBCE	NA	NA	NA	NA	NA	NA	NA	NA	NA	0,03015639
ILMN_1676946	AP3M2	NA	NA	NA	NA	NA	NA	NA	NA	NA	0,0301588
ILMN_2376859	PDGFD	NA	NA	0,000448201	NA	0,000100432	NA	NA	NA	NA	0,03017589
ILMN_2344373	MVP	NA	NA	0,00189941	NA	0,001435733	NA	NA	NA	NA	0,03019474
ILMN_1801421	EMD	NA	NA	NA	NA	NA	NA	NA	NA	NA	0,03020776
ILMN_1686985	MTM1	NA	NA	NA	NA	NA	NA	NA	0,000680335	NA	0,03031287
ILMN_1795719	RPA1	NA	NA	NA	NA	NA	NA	NA	NA	NA	0,03035711
ILMN_1809889	CCDC117	NA	NA	NA	NA	NA	NA	NA	NA	NA	0,03036079
ILMN_1728298	SBK1	NA	NA	NA	NA	0,006654283	NA	NA	NA	NA	0,0304177
ILMN_2135798	NR2C2AP	NA	NA	NA	NA	NA	NA	NA	NA	NA	0,0304552
ILMN_1664912	IL11RA	NA	NA	NA	NA	0,005552734	NA	NA	NA	NA	0,03046652
ILMN_1754145	CAPRIN1	NA	NA	NA	NA	0,004845481	NA	NA	NA	NA	0,03047428
ILMN_3235168	MUL1	NA	NA	NA	NA	NA	NA	NA	NA	NA	0,03055205
ILMN_2380237	C1QTNF1	0,008132847	NA	NA	NA	NA	NA	NA	NA	NA	0,03057958
ILMN_3248773	C7orf40	NA	NA	NA	NA	NA	NA	NA	0,003386098	NA	0,03063622
ILMN_1811063	LOC649447	NA	0,007813805	NA	NA	NA	NA	NA	NA	NA	0,0306869
ILMN_1655935	ADCY7	NA	NA	NA	NA	NA	NA	NA	NA	NA	0,03077061
ILMN_1794492	HOXC6	NA	NA	NA	NA	NA	NA	NA	NA	NA	0,03081012
ILMN_1744347	LOC127295	NA	NA	NA	NA	NA	NA	NA	NA	NA	0,03089201
ILMN_3186390	LOC1001285	NA	NA	0,000771528	NA	0,004095276	NA	NA	NA	NA	0,0309075
ILMN_1773470	ST5	NA	NA	NA	NA	NA	NA	NA	NA	NA	0,03091404
ILMN_1682910	SLC35B3	NA	NA	NA	NA	NA	NA	NA	NA	NA	0,03093461
ILMN_1768505	IL13RA1	NA	NA	NA	NA	NA	NA	NA	NA	NA	0,03094467
ILMN_1702124	LNX2	0,005417975	0,00440244	NA	NA	NA	NA	NA	NA	NA	0,03097547
ILMN_1810327	HNRPUL2	NA	NA	NA	NA	NA	NA	NA	NA	NA	0,03099177
ILMN_1865764		NA	NA	NA	NA	NA	NA	NA	NA	NA	0,03120013
ILMN_1808163	C11orf24	NA	NA	NA	NA	NA	NA	NA	NA	NA	0,03128663
ILMN_1798620	PQLC1	NA	NA	NA	NA	NA	NA	0,00361652	NA	NA	0,03131771
ILMN_3236858	NYNRIN	NA	NA	NA	NA	NA	NA	NA	NA	NA	0,03135885
ILMN_2392261	FABP6	NA	0,006382278	NA	NA	NA	NA	NA	NA	NA	0,03137693
ILMN_1702168	HSD17B12	NA	NA	NA	NA	NA	NA	NA	NA	NA	0,03147296
ILMN_1778464	TMEM1	NA	NA	NA	NA	NA	NA	NA	NA	NA	0,03149023
ILMN_1725992	RAPGEF6	NA	NA	0,002335328	NA	NA	NA	NA	NA	0,002200209	0,03151095
ILMN_2123431	RPS6KC1	NA	NA	NA	NA	NA	NA	NA	NA	NA	0,03171562

ILMN_1722292	AVL9	NA	NA	NA	NA	NA	NA	NA	NA	NA	0,03173147
ILMN_1677402	LOC387763	NA	NA	NA	NA	NA	NA	NA	NA	NA	0,03173187
ILMN_1728540	FUNDC1	NA	NA	NA	NA	NA	NA	NA	NA	NA	0,03175928
ILMN_1787628	NOP56	NA	NA	NA	NA	NA	NA	NA	NA	NA	0,03176772
ILMN_1659688	LGALS3BP	NA	NA	NA	NA	NA	NA	NA	NA	NA	0,03189115
ILMN_1762312	FOXRED1	NA	NA	NA	NA	NA	NA	NA	NA	NA	0,03194001
ILMN_2347798	IFI6	0,004852733	0,000584766	NA	NA	NA	NA	NA	NA	NA	0,03198178
ILMN_1793598	APIP	NA	NA	NA	NA	NA	NA	NA	NA	NA	0,03205829
ILMN_2412192	CFH	NA	NA	NA	NA	NA	NA	NA	NA	NA	0,03208552
ILMN_1669881	TSPAN13	NA	NA	NA	NA	NA	NA	NA	NA	NA	0,03210035
ILMN_3248263	CCDC93	NA	NA	0,001309191	NA	NA	NA	NA	NA	NA	0,03211437
ILMN_1728059	LOC645236	NA	NA	NA	NA	NA	NA	NA	NA	NA	0,03212276
ILMN_3252941	LOC1001279	NA	NA	NA	NA	NA	NA	NA	NA	NA	0,0321292
ILMN_1659350	CASP6	NA	NA	NA	NA	NA	NA	NA	NA	NA	0,03214971
ILMN_1711878	ENOPH1	NA	0,007728028	NA	NA	NA	NA	NA	NA	NA	0,03218244
ILMN_1770733	RIC8A	NA	NA	NA	NA	NA	NA	NA	NA	NA	0,03224741
ILMN_3226045	LOC728533	NA	NA	NA	NA	NA	NA	NA	NA	0,000593803	0,03228123
ILMN_1810559	RHOQ	NA	NA	NA	NA	NA	NA	NA	0,007263775	NA	0,03228173
ILMN_1807448	FAM8A1	NA	NA	NA	NA	NA	NA	NA	NA	NA	0,03231039
ILMN_1796738	SPAST	NA	NA	NA	NA	NA	NA	NA	0,000142622	NA	0,03231041
ILMN_1797828	DDRGK1	NA	1,21E-06	NA	NA	NA	NA	NA	NA	NA	0,032361
ILMN_1713454	ZNF671	NA	NA	NA	NA	NA	NA	NA	NA	NA	0,03255084
ILMN_1769606	PIWIL4	NA	NA	NA	NA	NA	NA	NA	NA	NA	0,03262569
ILMN_1699022	ENDOD1	NA	0,005398195	NA	NA	NA	NA	NA	NA	NA	0,03277813
ILMN_2127379	ST6GALNAC3	NA	NA	NA	NA	NA	NA	NA	NA	NA	0,03280638
ILMN_1656691	FBXO4	NA	NA	NA	NA	NA	NA	NA	NA	NA	0,032825
ILMN_2038775	TUBB2A	NA	NA	NA	NA	NA	NA	NA	NA	NA	0,03296633
ILMN_1673798	PPOX	0,000387977	0,000357432	NA	NA	NA	NA	NA	NA	NA	0,03296849
ILMN_2336595	ACSS2	NA	NA	NA	NA	NA	NA	NA	0,001315614	NA	0,03300449
ILMN_3247587	SLC48A1	NA	NA	NA	NA	NA	NA	NA	NA	NA	0,03300989
ILMN_1798108	C6orf211	NA	NA	NA	NA	0,009219464	NA	NA	NA	NA	0,03306124
ILMN_3225534	RNF216L	NA	NA	NA	NA	NA	NA	NA	NA	NA	0,0330831
ILMN_1663489	UBR2	0,003231092	0,003813953	NA	NA	NA	NA	NA	NA	NA	0,03308929
ILMN_2154654	PTP4A1	NA	NA	NA	NA	NA	NA	NA	0,007671318	NA	0,03312093
ILMN_3298694	TYW1B	NA	NA	NA	NA	NA	NA	NA	0,0041281	NA	0,03318051
ILMN_1672022	EPHA4	0,004426314	0,007888292	NA	NA	NA	NA	NA	NA	NA	0,03326056
ILMN_2104877	CMPK1	NA	NA	NA	NA	NA	NA	NA	NA	NA	0,03330136
ILMN_1740351	KIAA0174	NA	NA	NA	NA	NA	NA	NA	NA	5,16E-05	0,03337298
ILMN_2370365	RFC4	NA	NA	NA	NA	0,002261614	NA	NA	NA	NA	0,03341883
ILMN_1758918	BRD2	NA	NA	NA	NA	NA	NA	NA	NA	NA	0,03356376
ILMN_1750596	CLUAP1	NA	NA	NA	NA	NA	NA	NA	NA	NA	0,03358462
ILMN_1746588	TALDO1	NA	NA	NA	NA	NA	NA	NA	NA	NA	0,03364914
ILMN_1695034	LOC642817	NA	NA	NA	NA	NA	NA	NA	NA	NA	0,0337095
ILMN_1800276	RCN1	NA	NA	NA	NA	NA	NA	NA	0,002256783	NA	0,03372807
ILMN_3238123	ZNF844	0,004966341	NA	0,005471245	NA	NA	NA	NA	NA	0,005331509	0,0337288
ILMN_2049536	TRPV2	NA	NA	NA	NA	NA	NA	NA	NA	NA	0,03375569
ILMN_3244461	LRRC16A	NA	NA	NA	NA	NA	NA	NA	0,006105869	NA	0,03375591
ILMN_1768097	RPGR	NA	NA	NA	NA	NA	NA	NA	0,003137461	NA	0,03377788
ILMN_1683494	TMEM154	NA	NA	NA	NA	NA	NA	NA	0,006544786	NA	0,03380578
ILMN_1852384		NA	NA	NA	NA	NA	NA	NA	NA	NA	0,03383462
ILMN_2043845	IMPACT	NA	NA	NA	0,00290415	0,00964553	NA	NA	NA	NA	0,03391744
ILMN_1811648	DCAKD	NA	NA	NA	NA	NA	NA	NA	NA	NA	0,03394154
ILMN_1682599	GPRC5A	NA	NA	NA	NA	NA	NA	NA	0,008061931	NA	0,0339577
ILMN_2145670	TNC	NA	NA	NA	NA	NA	NA	NA	NA	NA	0,03397495
ILMN_1687384	IFI6	0,000600956	0,003190962	NA	NA	NA	NA	NA	NA	NA	0,03404616
ILMN_1789508	GTF3C3	NA	NA	NA	NA	NA	NA	NA	0,000765748	NA	0,03405055
ILMN_3188984	C20orf199	NA	NA	NA	NA	NA	NA	NA	NA	NA	0,03408052
ILMN_3242205	GMPS	NA	NA	0,003297901	NA	0,002178798	NA	NA	NA	NA	0,03418874
ILMN_1730294	INO80C	NA	NA	NA	NA	NA	NA	NA	0,009543326	NA	0,03423657
ILMN_2082893	LOC440157	NA	NA	NA	NA	NA	NA	NA	NA	NA	0,0342537
ILMN_3269719	ARAP1	NA	NA	NA	NA	NA	NA	NA	NA	NA	0,03428237
ILMN_2063925	CTNNBL1	NA	0,008333635	NA	NA	NA	NA	NA	NA	NA	0,03434112
ILMN_1678579	CPT2	NA	NA	NA	NA	NA	NA	NA	NA	NA	0,03439129
ILMN_2385161	CUL4B	NA	NA	NA	NA	NA	NA	NA	NA	NA	0,03440214
ILMN_3251303	NR2C2	NA	NA	NA	NA	NA	NA	NA	NA	NA	0,0344277
ILMN_1729237	CYB5R1	NA	NA	NA	NA	NA	NA	NA	NA	NA	0,03445109
ILMN_1751345	AP1S1	NA	NA	NA	NA	NA	NA	NA	NA	NA	0,03447366
ILMN_1718353	LOC652458	NA	NA	NA	NA	NA	NA	NA	NA	NA	0,03476805
ILMN_2128128	SHFM1	NA	NA	NA	NA	NA	NA	NA	NA	0,004219488	0,03478349
ILMN_1714567	AHNAK	NA	NA	NA	NA	NA	NA	NA	NA	NA	0,03480259
ILMN_1726666	GPX3	NA	0,00873895	0,00368575	NA	0,00788155	NA	NA	NA	NA	0,03480346
ILMN_2325347	B3GALNT1	NA	NA	0,006163224	NA	0,005949662	NA	NA	NA	NA	0,03488467
ILMN_2377185	TCEB2	0,009196122	NA	NA	NA	NA	NA	NA	NA	NA	0,03494179
ILMN_3244096	ZFC3H1	NA	NA	NA	NA	NA	NA	NA	NA	NA	0,03507095
ILMN_1651513	SKIV2L2	NA	NA	NA	NA	0,000531504	NA	NA	NA	NA	0,0350805
ILMN_1814808	BFAR	NA	NA	NA	NA	NA	NA	NA	NA	NA	0,03509585
ILMN_3199798	LOC389342	NA	NA	0,002134589	NA	0,002468291	NA	NA	NA	NA	0,03516618
ILMN_1715384	B3GNT6	NA	NA	NA	NA	NA	NA	NA	NA	NA	0,03516764
ILMN_1824665		NA	NA	NA	NA	NA	NA	NA	NA	NA	0,03519407
ILMN_1776088	NAT9	NA	NA	NA	NA	NA	NA	NA	NA	NA	0,03519452
ILMN_2378868	SFRS5	NA	NA	NA	NA	NA	NA	NA	NA	NA	0,03520215
ILMN_1769245	GLIPR1	NA	NA	NA	NA	NA	NA	NA	NA	NA	0,03521302
ILMN_1689869	GPR89C	NA	NA	NA	NA	NA	NA	NA	NA	NA	0,03524625
ILMN_3186473	LCLAT1	NA	NA	NA	NA	NA	NA	NA	NA	NA	0,0352792
ILMN_2072140	BTF3L4	NA	NA	NA	NA	NA	NA	NA	NA	NA	0,03529138
ILMN_1714809	RPIA	NA	NA	NA	NA	NA	NA	NA	0,006891652	NA	0,03529664
ILMN_1703754	CEP192	NA	NA	NA	NA	NA	NA	NA	NA	NA	0,03530347
ILMN_1703955	FBXO32	NA	NA	NA	NA	NA	NA	NA	NA	NA	0,03534172
ILMN_3259146	LOC1001296	NA	NA	NA	NA	NA	NA	NA	8,84E-05	NA	0,03534442
ILMN_2408039	EEF1D	NA	NA	NA	NA	NA	NA	NA	0,003255574	NA	0,03541892
ILMN_2345739	CAPRIN2	NA	NA	NA	NA	NA	NA	NA	NA	NA	0,03552412
ILMN_1725471	GK	NA	NA	NA	NA	NA	NA	NA	0,004177732	NA	0,03557183
ILMN_2198408	MFF	NA	9,75E-05	NA	NA	NA	NA	NA	NA	NA	0,03560028
ILMN_1810910	CFH	NA	NA	NA	NA	NA	NA	NA	NA	NA	0,03561863
ILMN_1774800	KTELC1	NA	NA	NA	NA	NA	NA	NA	NA	NA	0,03567807
ILMN_1693338	CYP1B1	NA	NA	0,003171738	NA	NA	NA	NA	NA	NA	0,0356979
ILMN_1751351	FLJ90086	NA	NA	NA	NA	NA	NA	NA	NA	NA	0,03571317
ILMN_1739297	GALNT4	NA	NA	NA	NA	NA	NA	NA	NA	0,003698189	0,03575228
ILMN_2215211	ZNF514	0,001616089	NA	NA	NA	NA	NA	NA	NA	NA	0,03580843
ILMN_1653504	EDG1	NA	NA	NA	NA	NA	NA	NA	NA	0,008804628	0,03594097
ILMN_1707551	AFMID	NA	NA	NA	NA	NA	NA	NA	NA	NA	0,03595835
ILMN_1668411	FHL2	NA	NA	NA	NA	NA	NA	NA	NA	NA	0,03600436
ILMN_1803995	TM7SF3	NA	NA	NA	NA	NA	NA	NA	0,00769609	NA	0,03601911
ILMN_2112049	DNLZ	0,000417667	NA	NA	NA	NA	NA	NA	NA	NA	0,03602956
ILMN_1724230	LOC642236	NA	NA	NA	NA	NA	NA	NA	NA	NA	0,03606793
ILMN_2359907	CD68	NA	NA	NA	NA	0,002275459	NA	NA	NA	NA	0,03607294
ILMN_1683096	ASB1	NA	NA	NA	NA	0,009732765	NA	NA	NA	NA	0,03620451
ILMN_3293367	LOC391370	NA	NA	NA	NA	NA	NA	NA	NA	NA	0,03627888
ILMN_1795856	LOC644935	NA	NA	0,007142024	NA	NA	NA	NA	NA	NA	0,03630973

ILMN_1785107	NXT2	NA	NA	NA	NA	NA	NA	NA	0,009877767	NA	0,03635678
ILMN_1792415	C3orf39	NA	NA	NA	NA	NA	NA	NA	NA	NA	0,03635714
ILMN_3304042	LOC729051	NA	NA	NA	NA	NA	NA	NA	NA	NA	0,03637048
ILMN_1731224	PARP9	NA	NA	0,001599309	NA	NA	NA	NA	NA	0,004271992	0,03642034
ILMN_1660426	CPSF4	NA	NA	NA	NA	NA	NA	NA	NA	NA	0,03642843
ILMN_2341467	PARL	NA	NA	NA	NA	NA	NA	NA	NA	NA	0,03643731
ILMN_1660451	STARD13	NA	NA	NA	NA	0,000688299	NA	NA	NA	NA	0,03646163
ILMN_1805148	C2orf63	NA	NA	NA	NA	0,009994435	NA	NA	NA	NA	0,03649431
ILMN_1696699	FAM184A	NA	NA	NA	NA	NA	NA	NA	NA	NA	0,03651875
ILMN_1741096	FDFT1	NA	NA	NA	NA	NA	NA	NA	0,00737658	NA	0,036568
ILMN_1665554	BRF2	NA	NA	NA	NA	NA	NA	NA	NA	NA	0,03664792
ILMN_1698706	NID2	NA	NA	NA	NA	NA	NA	NA	NA	NA	0,03665342
ILMN_1687303	ACAD10	NA	0,002430187	NA	NA	NA	NA	NA	NA	NA	0,03668907
ILMN_1790309	PINX1	NA	NA	NA	NA	NA	NA	NA	NA	NA	0,03669297
ILMN_1732534	CHMP5	NA	NA	NA	NA	NA	NA	NA	NA	NA	0,03670155
ILMN_1761722	ZNF579	NA	NA	NA	NA	NA	NA	NA	NA	NA	0,03673127
ILMN_2404063	APP	NA	NA	NA	NA	NA	NA	NA	0,000911685	NA	0,03680111
ILMN_2373177	PANK2	NA	NA	NA	NA	NA	NA	NA	NA	NA	0,03685069
ILMN_1714159	LUZP1	NA	NA	0,000310171	NA	0,00762767	NA	NA	NA	NA	0,03686236
ILMN_1870085		NA	0,008590189	NA	NA	NA	NA	NA	NA	NA	0,03692302
ILMN_3292244	LOC1001328	NA	NA	NA	NA	NA	NA	NA	NA	NA	0,03700039
ILMN_1739674	PDE4DIP	NA	NA	NA	NA	NA	NA	NA	NA	NA	0,03701086
ILMN_1796244	CD2BP2	NA	NA	NA	NA	NA	NA	NA	NA	NA	0,03701501
ILMN_3239946	LOC729375	NA	NA	NA	NA	NA	NA	NA	NA	NA	0,03702016
ILMN_2201678	FSTL1	NA	NA	NA	NA	0,006940751	NA	NA	NA	NA	0,03703422
ILMN_1683313	ST3GAL1	NA	NA	NA	NA	NA	NA	NA	NA	NA	0,03710082
ILMN_1699644	MARCH3	NA	NA	NA	NA	NA	NA	NA	NA	NA	0,03712627
ILMN_1815184	ASPM	NA	NA	NA	NA	NA	NA	NA	NA	NA	0,03713276
ILMN_1706502	EIF2AK2	0,0052006	NA	0,009580373	NA	NA	NA	NA	NA	0,002044879	0,03717931
ILMN_1798975	EGFR	0,009169964	NA	NA	NA	NA	NA	NA	NA	NA	0,03718211
ILMN_2331266	NUMB	NA	NA	NA	NA	NA	NA	NA	NA	NA	0,03725839
ILMN_1751726	LOC649049	NA	NA	NA	NA	NA	NA	NA	NA	NA	0,03743612
ILMN_2202747	ELA2A	NA	NA	NA	NA	NA	NA	NA	0,00125891	NA	0,03757432
ILMN_2053345	KCNT2	NA	NA	NA	NA	NA	NA	NA	NA	NA	0,03760118
ILMN_1779264	PSMG1	NA	NA	0,002745924	NA	0,001466878	NA	NA	NA	NA	0,03761863
ILMN_2067607	TMEM106B	NA	NA	NA	NA	NA	NA	NA	NA	NA	0,03767057
ILMN_1773650	LRRN3	NA	NA	NA	NA	NA	NA	NA	NA	NA	0,03791093
ILMN_1655904	ROR1	NA	NA	NA	NA	NA	NA	NA	NA	NA	0,03793451
ILMN_1672004	TOB1	NA	NA	0,002119052	NA	NA	NA	NA	NA	0,009681675	0,03795733
ILMN_2355831	FHL2	NA	NA	NA	NA	NA	NA	NA	NA	NA	0,03797743
ILMN_1712918	NQO2	NA	NA	NA	NA	NA	NA	NA	NA	NA	0,0379991
ILMN_2407879	SORBS2	NA	NA	NA	NA	NA	NA	NA	NA	NA	0,03800743
ILMN_3272378	EZR	NA	NA	0,000104329	NA	0,003731102	NA	NA	NA	NA	0,0380968
ILMN_1767766	PRDX2	NA	NA	NA	NA	NA	NA	NA	NA	NA	0,03816241
ILMN_2245305	ABHD12	NA	NA	NA	NA	0,006893285	NA	NA	NA	NA	0,03821365
ILMN_2181125	NAPB	NA	NA	NA	NA	NA	NA	NA	0,003591877	NA	0,03828233
ILMN_1865013		NA	NA	NA	NA	NA	NA	NA	NA	NA	0,03830993
ILMN_1653274	NUDT10	NA	NA	NA	NA	NA	NA	NA	NA	NA	0,03837499
ILMN_2072622	FLJ12684	NA	NA	NA	NA	NA	NA	NA	NA	NA	0,03839684
ILMN_2134110	SELK	NA	NA	NA	NA	NA	NA	NA	0,001300175	NA	0,03841183
ILMN_2401826	FTSJ1	NA	NA	NA	NA	NA	NA	NA	NA	NA	0,03843146
ILMN_1732885	BTBD12	NA	NA	NA	NA	NA	NA	NA	0,002547774	NA	0,03843456
ILMN_2157240	MNS1	NA	NA	NA	NA	NA	NA	NA	NA	NA	0,03845909
ILMN_1813275	DUSP22	NA	NA	NA	NA	NA	NA	NA	NA	NA	0,03847011
ILMN_1716407	SORBS2	NA	NA	NA	NA	NA	NA	NA	NA	NA	0,03856334
ILMN_2361185	PHF20L1	NA	NA	0,005401231	NA	NA	NA	NA	NA	NA	0,03863946
ILMN_1705746	LSG1	NA	NA	NA	NA	NA	NA	NA	NA	NA	0,03865511
ILMN_1771734	LOC728564	NA	NA	0,000188112	NA	0,001864435	NA	NA	NA	NA	0,03868336
ILMN_1802808	LOC654103	NA	NA	NA	NA	NA	NA	NA	NA	NA	0,03881072
ILMN_1718961	BNIP3L	NA	NA	NA	NA	NA	NA	NA	0,000640074	NA	0,03881497
ILMN_1784946	ORC3L	NA	NA	NA	NA	NA	NA	NA	NA	NA	0,03884266
ILMN_1676280	NSUN3	NA	NA	NA	NA	NA	NA	NA	NA	NA	0,03885495
ILMN_1784037	ZBTB40	NA	NA	NA	NA	NA	NA	NA	NA	NA	0,03888854
ILMN_1675939	IFNGR1	NA	NA	NA	NA	NA	NA	NA	NA	NA	0,03892181
ILMN_1750167	PRR3	NA	NA	NA	NA	NA	NA	NA	NA	NA	0,03894663
ILMN_2068104	TFPI2	NA	NA	NA	NA	NA	NA	NA	NA	NA	0,03908159
ILMN_1671791	PCK2	NA	NA	NA	NA	NA	NA	NA	NA	NA	0,0390836
ILMN_1759252	ADD1	NA	NA	NA	NA	NA	NA	NA	NA	NA	0,03911809
ILMN_1740512	MGC39900	NA	NA	0,003173431	NA	NA	NA	NA	NA	NA	0,03916845
ILMN_1729217	FAM131A	NA	NA	NA	NA	NA	NA	NA	NA	NA	0,03917917
ILMN_2409395	CCNC	NA	NA	NA	NA	NA	NA	NA	NA	NA	0,03919026
ILMN_2354391	EGLN2	NA	NA	NA	NA	NA	NA	NA	NA	0,006981602	0,03925645
ILMN_2063500	IFT52	NA	NA	NA	NA	NA	NA	NA	NA	NA	0,03925938
ILMN_2055477	EXOSC7	NA	NA	NA	NA	NA	NA	NA	NA	NA	0,03933219
ILMN_2380494	ANXA11	NA	NA	NA	NA	NA	NA	NA	0,000536555	NA	0,03937474
ILMN_1674650	C9orf95	NA	NA	NA	NA	NA	NA	NA	NA	NA	0,03939191
ILMN_1751051	C7orf25	NA	0,002877386	NA	NA	NA	NA	NA	NA	NA	0,03940007
ILMN_1793025	OSAP	NA	NA	NA	NA	NA	NA	0,003619819	NA	NA	0,0394267
ILMN_3236970	LOC339290	NA	NA	NA	NA	NA	NA	NA	NA	NA	0,03944189
ILMN_1655796	MARCH3	NA	NA	NA	NA	NA	NA	NA	NA	NA	0,03944588
ILMN_2196335	C12orf11	NA	NA	NA	NA	NA	NA	NA	NA	NA	0,03946328
ILMN_3298167	ZSWIM7	NA	0,000321275	NA	NA	NA	NA	NA	NA	NA	0,03946367
ILMN_3301324	LOC729301	NA	NA	NA	NA	NA	NA	NA	NA	NA	0,03949037
ILMN_2268068	MAPKAP1	NA	NA	NA	NA	NA	NA	NA	0,005345114	NA	0,03952023
ILMN_2038776	TXN	NA	0,000272515	NA	NA	NA	NA	NA	NA	NA	0,03952165
ILMN_1741171	TM2D2	NA	NA	0,008828582	NA	NA	NA	NA	NA	NA	0,03965321
ILMN_1808811	SBNO2	NA	NA	0,009865936	NA	NA	NA	NA	NA	NA	0,03973926
ILMN_2409167	ANXA2	NA	NA	NA	NA	NA	NA	NA	NA	NA	0,03975577
ILMN_2334296	IL18BP	NA	NA	NA	NA	NA	NA	NA	NA	NA	0,03982127
ILMN_1714622	TNRC6A	NA	NA	NA	NA	NA	NA	NA	NA	NA	0,03987374
ILMN_1710124	CMTM8	NA	NA	0,007003537	NA	NA	NA	NA	NA	NA	0,039946
ILMN_1670901	COX10	0,001498784	NA	NA	NA	NA	NA	NA	NA	NA	0,04003924
ILMN_2182120	SF3B14	NA	NA	NA	NA	NA	NA	NA	NA	NA	0,04004604
ILMN_1699631	GATS	NA	0,000653479	NA	NA	NA	NA	NA	NA	NA	0,04005667
ILMN_2172174	NP	NA	NA	NA	NA	NA	NA	NA	NA	NA	0,04008829
ILMN_1676984	DDIT3	NA	NA	NA	NA	NA	NA	NA	NA	NA	0,04014539
ILMN_1745119	C7orf11	0,000829648	0,003134289	0,005671228	NA	NA	NA	NA	NA	NA	0,04017253
ILMN_3231820	SIVA1	NA	NA	NA	NA	NA	NA	NA	NA	NA	0,04017378
ILMN_2067656	CCND2	NA	NA	NA	NA	NA	NA	NA	0,005433538	NA	0,04017871
ILMN_1725760	YTHDC2	NA	NA	0,003376096	NA	0,001100636	NA	NA	NA	NA	0,04019289
ILMN_1803775	HSPE1	NA	NA	0,003854003	NA	NA	NA	NA	NA	NA	0,04021693
ILMN_1766085	REL	NA	NA	NA	NA	NA	NA	NA	NA	NA	0,04024517
ILMN_1706583	DLAT	NA	0,001367688	NA	NA	NA	NA	NA	NA	NA	0,04038138
ILMN_3271122	LOC1001297	NA	NA	NA	NA	NA	NA	NA	NA	NA	0,04040474
ILMN_2217212	ZNF564	NA	NA	NA	NA	NA	NA	NA	NA	NA	0,04040671
ILMN_1782488	RNASEH2B	NA	NA	NA	NA	NA	NA	NA	NA	NA	0,04042685
ILMN_2073010	TMEM203	NA	0,004888031	NA	NA	NA	NA	0,002863979	NA	NA	0,04043801
ILMN_1811592	ARHGAP21	NA	0,004330822	NA	NA	NA	NA	NA	NA	NA	0,04048461
ILMN_2181883	C14orf129	NA	NA	NA	NA	NA	NA	NA	NA	NA	0,04049809

ILMN_1696276	ZNF653	NA	NA	NA	NA	NA	NA	NA	NA	NA	0,04053893
ILMN_1783815	COG7	0,003565724	NA	NA	NA	NA	NA	NA	NA	NA	0,04064108
ILMN_2369953	ARL4A	NA	NA	NA	NA	NA	NA	NA	NA	0,00746024	0,04071458
ILMN_1814044	CABIN1	NA	NA	NA	NA	NA	NA	NA	NA	NA	0,04075075
ILMN_1783593	CCL13	NA	0,004136116	NA	NA	NA	NA	NA	NA	NA	0,04075596
ILMN_2360307	SUSD4	NA	NA	NA	NA	NA	NA	NA	NA	NA	0,04076443
ILMN_1739805	NDE1	NA	NA	NA	NA	NA	NA	NA	NA	NA	0,04076907
ILMN_3233930	LOC390557	NA	NA	NA	NA	NA	NA	NA	NA	NA	0,040784
ILMN_2089175	SYAP1	NA	NA	NA	0,00545868	0,008447234	NA	NA	NA	NA	0,0408004
ILMN_3234513	LOC728416	NA	NA	NA	NA	NA	NA	NA	NA	NA	0,04083175
ILMN_1695290	FERMT2	NA	NA	NA	NA	NA	NA	NA	NA	NA	0,04083658
ILMN_1715908	NCAPH2	NA	NA	NA	NA	NA	NA	NA	NA	NA	0,04088146
ILMN_1754051	RMI1	NA	NA	NA	NA	NA	NA	NA	NA	NA	0,04089249
ILMN_1786658	BOLA3	NA	NA	NA	NA	NA	NA	NA	0,002299057	NA	0,04095092
ILMN_1768751	MTA3	NA	NA	NA	NA	NA	NA	NA	NA	NA	0,04096712
ILMN_3202734	LOC1001327	NA	NA	NA	NA	NA	NA	NA	NA	NA	0,040999
ILMN_1721113	HLA-C	NA	4,43E-05	NA	NA	NA	NA	NA	NA	NA	0,04100426
ILMN_1739045	LOC647009	NA	NA	NA	NA	NA	NA	NA	NA	NA	0,04103276
ILMN_1777663	TOP2B	NA	NA	NA	NA	NA	NA	NA	NA	NA	0,04107046
ILMN_1807397	NDUFB5	NA	NA	0,008999104	NA	0,009370231	NA	NA	NA	NA	0,04109963
ILMN_3195203	C17orf101	NA	NA	NA	NA	NA	NA	NA	NA	NA	0,04116636
ILMN_1685124	TCTN1	NA	NA	NA	NA	NA	NA	NA	0,00387804	NA	0,04119972
ILMN_2073604	EBP	NA	NA	NA	NA	NA	NA	NA	0,000616962	NA	0,04121438
ILMN_1753472	SUMF1	NA	NA	NA	NA	NA	NA	NA	NA	NA	0,04123282
ILMN_1666885	ATRX	NA	NA	NA	NA	NA	NA	NA	NA	NA	0,0412673
ILMN_1662438	SOD1	NA	NA	NA	NA	NA	NA	NA	NA	NA	0,04131053
ILMN_1662243	ING1	NA	NA	NA	NA	NA	NA	NA	NA	NA	0,04131995
ILMN_2325574	CASC4	NA	NA	NA	NA	NA	NA	NA	1,64E-05	NA	0,04132831
ILMN_1723874	MRPS6	0,00335397	NA	0,000119165	NA	0,004828572	NA	NA	NA	NA	0,04133372
ILMN_1744118	ASTN2	NA	NA	NA	NA	NA	NA	NA	NA	NA	0,04139823
ILMN_1767320	MAPK1	0,00191621	NA	0,008585074	NA	NA	NA	NA	NA	0,000522922	0,04140166
ILMN_1733998	DHRS9	NA	NA	NA	NA	NA	NA	NA	NA	NA	0,0414086
ILMN_1900520		NA	NA	NA	NA	NA	NA	NA	NA	NA	0,04142404
ILMN_3287058	LOC1001320	NA	NA	NA	NA	NA	NA	NA	0,007330717	NA	0,04143945
ILMN_1800837	CFDP1	NA	NA	NA	NA	NA	NA	NA	NA	NA	0,041477
ILMN_1720965	TULP4	NA	NA	NA	NA	NA	NA	NA	0,006840556	NA	0,04153712
ILMN_1882248		NA	NA	NA	NA	NA	NA	NA	NA	NA	0,04155852
ILMN_1736730	LRRC16	NA	NA	NA	NA	NA	NA	NA	NA	NA	0,04157953
ILMN_1763409	LRRC8D	0,005246112	NA	NA	NA	NA	NA	NA	NA	0,001631935	0,04164481
ILMN_1671265	ING2	NA	NA	NA	NA	NA	NA	NA	NA	NA	0,04164573
ILMN_1667356	CCDC128	NA	0,000737641	NA	NA	NA	NA	NA	NA	NA	0,0416591
ILMN_2352097	GPR56	NA	NA	NA	NA	NA	NA	NA	NA	NA	0,04175186
ILMN_1777721	MAPRE1	NA	NA	NA	NA	NA	NA	NA	NA	NA	0,04184272
ILMN_2395926	MANBAL	NA	NA	NA	NA	NA	NA	NA	NA	NA	0,04187856
ILMN_1793290	WDR60	NA	NA	NA	NA	NA	NA	NA	0,003080463	NA	0,04189904
ILMN_1756982	CLIC1	NA	NA	NA	NA	NA	NA	NA	NA	NA	0,04192161
ILMN_2309180	SMARCD3	NA	NA	NA	NA	NA	NA	NA	0,005207499	NA	0,04192549
ILMN_1671932	SAMM50	NA	NA	NA	NA	NA	NA	NA	NA	NA	0,04193037
ILMN_1718354	INTS7	NA	0,005759926	NA	NA	NA	NA	NA	NA	NA	0,04193304
ILMN_1793563	DCTN1	NA	NA	NA	NA	NA	NA	NA	NA	NA	0,04193953
ILMN_1791568	CEP57	NA	NA	NA	NA	NA	NA	NA	NA	NA	0,04196851
ILMN_1769449	MBLAC2	0,007875905	0,002112575	NA	NA	NA	NA	NA	NA	NA	0,04206116
ILMN_1811104	KTELC1	NA	NA	NA	NA	NA	NA	NA	NA	NA	0,04208912
ILMN_1664303	HTATIP2	NA	NA	NA	NA	NA	NA	NA	NA	NA	0,04211565
ILMN_1792669	HLCS	NA	0,001766226	NA	NA	NA	NA	NA	NA	NA	0,04212563
ILMN_2339835	PTGS1	NA	NA	NA	NA	0,005975099	NA	NA	NA	NA	0,04214591
ILMN_1705876	NAP1L1	NA	NA	NA	NA	NA	NA	NA	0,005451203	NA	0,04215829
ILMN_1782086	AOC3	0,003573307	NA	0,00347485	NA	NA	NA	NA	NA	NA	0,04219351
ILMN_1898682		NA	NA	NA	NA	0,007670629	NA	NA	NA	NA	0,04219627
ILMN_1662989	PRB2	NA	NA	NA	NA	NA	NA	NA	NA	NA	0,0421982
ILMN_2360415	PRNP	NA	NA	NA	NA	NA	NA	NA	NA	NA	0,04223288
ILMN_1654151	COX6C	NA	NA	NA	NA	NA	NA	NA	NA	NA	0,04226259
ILMN_2094776	CCNL1	NA	NA	NA	NA	NA	NA	NA	NA	NA	0,04233176
ILMN_3288529	LOC645630	NA	NA	NA	NA	NA	NA	NA	NA	NA	0,04235338
ILMN_1667016	FAF1	NA	NA	NA	NA	NA	NA	NA	NA	NA	0,04240897
ILMN_2395204	SLTM	NA	NA	NA	NA	NA	NA	NA	NA	NA	0,04242181
ILMN_2407824	ATP1B1	NA	NA	NA	NA	NA	NA	NA	NA	NA	0,0424468
ILMN_1709233	F5	NA	NA	0,008001482	NA	NA	NA	NA	NA	0,007547027	0,04247903
ILMN_1810836	PDE5A	2,58E-05	NA	0,004191093	NA	NA	NA	NA	NA	NA	0,04255081
ILMN_1670490	PDPN	0,009517879	NA	0,008784488	NA	0,007255007	NA	NA	NA	NA	0,04257953
ILMN_2081988	LANCL1	NA	NA	NA	NA	NA	NA	NA	NA	NA	0,04262279
ILMN_1664826	FBXO33	NA	NA	NA	NA	NA	NA	NA	NA	NA	0,04281585
ILMN_1733110	RASSF7	NA	NA	NA	NA	NA	NA	NA	NA	NA	0,04287691
ILMN_1677567	EPYC	NA	NA	NA	NA	NA	NA	NA	0,001399048	NA	0,04291004
ILMN_1680673	NT5DC1	NA	NA	NA	NA	NA	NA	NA	NA	NA	0,04295901
ILMN_1669747	UNC13B	NA	NA	NA	NA	NA	NA	NA	NA	NA	0,04297685
ILMN_1807152	GOLGA1	NA	NA	NA	NA	NA	NA	NA	NA	NA	0,04303517
ILMN_1782749	ZNF468	0,000924715	NA	0,000975018	NA	0,006166918	NA	NA	NA	NA	0,0430711
ILMN_2167616	NACA	NA	NA	NA	NA	NA	NA	NA	NA	NA	0,04308393
ILMN_1752728	FUCA1	NA	NA	NA	NA	NA	NA	NA	0,004380424	NA	0,0431443
ILMN_1843585		NA	NA	NA	NA	NA	NA	NA	NA	NA	0,04314571
ILMN_1778381	LOC650029	NA	NA	NA	NA	NA	NA	NA	NA	0,008130021	0,04317324
ILMN_1703335	LACTB	NA	NA	0,00581803	NA	NA	NA	NA	NA	NA	0,04317608
ILMN_1664698	UNC119	NA	NA	NA	NA	NA	NA	NA	NA	NA	0,04318834
ILMN_1751958	NSUN5	NA	NA	NA	NA	NA	NA	NA	NA	NA	0,04319046
ILMN_1694847	TERF1	0,00995259	NA	0,000138226	NA	0,000945048	NA	NA	NA	NA	0,04328113
ILMN_1786920	JARID1A	NA	0,003425802	NA	NA	NA	NA	NA	NA	NA	0,04336808
ILMN_1720083	EHD4	NA	NA	0,003741831	NA	NA	NA	NA	NA	0,001617891	0,04346589
ILMN_1804327	NAP1L4	NA	NA	NA	NA	NA	NA	NA	NA	NA	0,04350418
ILMN_3224126	LOC729222	NA	NA	NA	NA	NA	NA	NA	NA	NA	0,04351902
ILMN_1769911	SLC38A1	NA	NA	0,005167982	NA	NA	NA	NA	NA	NA	0,04356401
ILMN_2309245	BIN1	NA	NA	NA	NA	NA	NA	NA	NA	NA	0,04358065
ILMN_1685493	TMCC3	NA	NA	NA	NA	0,006786078	NA	NA	NA	NA	0,04361234
ILMN_2154455	ZNF330	NA	NA	NA	NA	NA	NA	NA	NA	NA	0,04361692
ILMN_1759084	INTS8	NA	NA	NA	NA	NA	NA	NA	NA	NA	0,04361724
ILMN_3245517	LOC1001342	NA	NA	0,005723272	NA	NA	NA	NA	NA	NA	0,0436314
ILMN_1698144	CFH	NA	NA	NA	NA	NA	NA	NA	NA	NA	0,04368123
ILMN_2400922	OPRL1	NA	NA	NA	NA	NA	NA	NA	NA	NA	0,04375928
ILMN_2141453	RPL18A	NA	NA	0,009416364	NA	0,00792634	NA	NA	NA	NA	0,04380456
ILMN_1729112	CHPT1	NA	NA	NA	NA	NA	NA	NA	NA	NA	0,04382588
ILMN_2146648	PRB3	NA	NA	NA	NA	NA	NA	NA	NA	NA	0,04383327
ILMN_1654689	ZNF197	NA	NA	NA	NA	NA	NA	NA	NA	NA	0,04391016
ILMN_1770290	CNN2	NA	NA	NA	NA	NA	NA	NA	NA	NA	0,04391445
ILMN_2117987	TFDP1	NA	NA	NA	NA	NA	NA	NA	NA	NA	0,04402821
ILMN_3264073	LOC1001300	NA	NA	0,009346088	NA	NA	NA	NA	NA	NA	0,04403067
ILMN_1712088	CLYBL	NA	NA	0,006591898	NA	NA	NA	NA	NA	NA	0,04403913
ILMN_1651832	EHD1	NA	NA	NA	NA	NA	NA	NA	NA	NA	0,04406707
ILMN_1730260	N6AMT2	NA	NA	NA	NA	NA	NA	NA	NA	NA	0,04407222
ILMN_3297880	SEC13	NA	NA	NA	NA	NA	NA	NA	NA	NA	0,04414394

ILMN_2137066	ZNF7	NA	NA	NA	NA	NA	NA	NA	NA	NA	0,04415454
ILMN_1807201	FAM104A	NA	NA	NA	NA	NA	NA	NA	0,003275648	NA	0,0441695
ILMN_2321634	RAD17	NA	NA	NA	NA	NA	NA	NA	NA	NA	0,04417029
ILMN_1739521	NLGN1	NA	NA	NA	NA	NA	NA	NA	NA	NA	0,04423063
ILMN_3254322	LOC1001297	NA	NA	NA	NA	NA	NA	NA	NA	0,007063828	0,04423793
ILMN_2312906	MIA3	NA	NA	NA	NA	NA	NA	NA	NA	NA	0,04439665
ILMN_1697469	SFRS6	NA	NA	NA	NA	NA	NA	NA	NA	NA	0,04446654
ILMN_1651705	CAT	NA	NA	NA	NA	NA	NA	NA	NA	NA	0,04446765
ILMN_1725556	LOC730130	NA	NA	NA	NA	NA	NA	NA	NA	NA	0,04448368
ILMN_1674160	BIN1	NA	NA	NA	NA	NA	NA	NA	NA	NA	0,04461158
ILMN_1691575	SNX2	NA	NA	NA	NA	NA	NA	NA	NA	NA	0,04468876
ILMN_1776038	UBXN2A	NA	NA	NA	NA	NA	NA	NA	NA	NA	0,04473305
ILMN_1702609	B3GNT5	NA	0,000310396	NA	NA	NA	NA	NA	NA	NA	0,04474611
ILMN_1698968	ASXL2	NA	NA	NA	NA	NA	NA	NA	NA	NA	0,04476249
ILMN_1758104	PRPS2	NA	NA	NA	NA	0,00465725	NA	NA	NA	NA	0,04479523
ILMN_2194828	C16orf53	NA	0,003835224	NA	NA	NA	NA	NA	NA	NA	0,04480325
ILMN_1803197	RAB3IP	NA	NA	NA	NA	NA	NA	NA	NA	NA	0,04481068
ILMN_2304624	EIF4H	NA	NA	NA	NA	NA	NA	NA	NA	NA	0,04492314
ILMN_1872404		NA	3,68E-06	NA	NA	NA	NA	NA	NA	NA	0,04494202
ILMN_1729749	HERC5	NA	NA	NA	NA	NA	NA	NA	NA	NA	0,04496016
ILMN_2193325	MMP23B	NA	0,002943766	NA	NA	NA	NA	NA	NA	NA	0,04497387
ILMN_1716797	CD302	NA	NA	NA	NA	NA	NA	NA	0,003566265	NA	0,04513475
ILMN_1778611	GBAS	NA	NA	NA	NA	NA	NA	NA	NA	NA	0,04517365
ILMN_1757646	UFM1	NA	0,003983548	NA	NA	NA	NA	NA	NA	NA	0,04522234
ILMN_2059452	SLC12A2	NA	NA	NA	NA	NA	NA	NA	NA	NA	0,04523134
ILMN_1677607	SC5DL	NA	NA	NA	NA	NA	NA	NA	NA	NA	0,04525239
ILMN_2263718	SPAG9	NA	NA	NA	NA	NA	NA	NA	NA	0,00660543	0,04528996
ILMN_2395055	ATPAF1	NA	NA	NA	NA	NA	NA	NA	NA	NA	0,04532498
ILMN_1738759	PIGT	NA	NA	NA	NA	0,000322773	NA	NA	NA	NA	0,04540865
ILMN_1703316	LOC255783	0,002453918	NA	NA	NA	NA	NA	NA	NA	NA	0,04544582
ILMN_3243274	NCRNA0020(	NA	NA	NA	NA	NA	NA	NA	NA	NA	0,04549271
ILMN_1736441	PDXP	NA	NA	NA	NA	NA	NA	NA	NA	0,009766508	0,04553018
ILMN_1714083	KLHL8	NA	NA	NA	NA	NA	NA	NA	NA	NA	0,04557384
ILMN_1755822	SYDE1	NA	NA	NA	NA	NA	NA	NA	NA	NA	0,04561316
ILMN_2296697	MAP3K3	NA	NA	NA	NA	NA	NA	NA	0,006643272	NA	0,04566766
ILMN_1796417	ASNS	NA	NA	NA	NA	NA	NA	NA	NA	NA	0,04567865
ILMN_1669905	DCP2	NA	NA	NA	NA	NA	NA	NA	NA	NA	0,04568189
ILMN_1779997	MEIS3	NA	NA	NA	NA	NA	NA	NA	NA	NA	0,04569313
ILMN_1686555	FYN	NA	NA	NA	NA	NA	NA	NA	NA	NA	0,04576691
ILMN_1787576	CLCNKA	6,90E-05	NA	0,001111908	NA	NA	NA	NA	NA	0,00227569	0,04576761
ILMN_2386100	BUB3	NA	NA	NA	NA	NA	NA	NA	NA	NA	0,04582039
ILMN_1880885		NA	NA	0,002798843	NA	0,001944059	NA	NA	NA	NA	0,04582153
ILMN_1762932	CHMP2A	NA	NA	NA	NA	NA	NA	NA	NA	NA	0,04591628
ILMN_2166831	RPS4X	NA	NA	NA	NA	NA	NA	NA	NA	NA	0,04592434
ILMN_3232894	CNRIP1	NA	NA	NA	NA	NA	NA	NA	NA	NA	0,04595781
ILMN_1698213	RBM3	NA	NA	NA	NA	NA	NA	NA	NA	NA	0,04595803
ILMN_1772731	HAGH	NA	NA	NA	NA	NA	NA	NA	NA	NA	0,04596163
ILMN_1785071	SEPP1	NA	NA	NA	NA	NA	NA	NA	NA	NA	0,04597853
ILMN_1766222	LARP4B	NA	NA	NA	NA	NA	NA	NA	NA	NA	0,045982
ILMN_1768271	SMAP1	NA	NA	NA	NA	NA	NA	NA	0,008848675	NA	0,04601805
ILMN_1776993	COG2	NA	NA	NA	NA	NA	NA	NA	NA	NA	0,04602504
ILMN_1659017	C11orf74	NA	0,004800505	NA	NA	NA	NA	NA	NA	NA	0,04605396
ILMN_1694504	RNF220	NA	NA	NA	NA	NA	NA	NA	NA	NA	0,04606063
ILMN_1698934	CMTM7	NA	NA	NA	NA	NA	NA	NA	NA	NA	0,04606192
ILMN_1774828	VEZT	NA	NA	NA	NA	NA	NA	NA	NA	NA	0,04608845
ILMN_1698554	AACS	NA	NA	NA	NA	NA	NA	NA	0,001207866	NA	0,04611733
ILMN_1764207	RPRD1A	NA	NA	NA	NA	NA	NA	NA	NA	NA	0,04618472
ILMN_1758679	TMEM168	NA	NA	NA	NA	NA	NA	NA	NA	NA	0,04628299
ILMN_3290800	LOC647081	NA	NA	NA	NA	NA	NA	NA	NA	NA	0,04629894
ILMN_1788689	PHIP	NA	NA	NA	NA	NA	NA	NA	NA	NA	0,04629959
ILMN_1741954	SMYD3	NA	0,005752928	NA	NA	NA	NA	NA	NA	NA	0,04631988
ILMN_1774086	CBX3	NA	NA	NA	NA	NA	NA	NA	0,008464783	NA	0,04632564
ILMN_1764573	XIST	NA	NA	0,002173461	NA	0,001713966	NA	NA	NA	NA	0,04639392
ILMN_1710220	LOC729985	NA	NA	NA	NA	0,009309216	NA	NA	NA	NA	0,04646159
ILMN_1765557	OLFML2B	NA	NA	NA	NA	NA	NA	NA	0,002072425	NA	0,04650871
ILMN_1689189	ZBTB3	NA	NA	0,007102367	NA	NA	NA	NA	NA	NA	0,04650942
ILMN_1794260	FBXL10	NA	NA	NA	NA	NA	NA	NA	NA	NA	0,04655667
ILMN_1728799	FBP1	NA	NA	NA	NA	NA	NA	0,003086495	NA	NA	0,04658255
ILMN_1859863		NA	NA	NA	NA	NA	NA	NA	NA	NA	0,04661825
ILMN_3289262	LOC1001312	NA	NA	NA	NA	NA	NA	NA	NA	NA	0,04662912
ILMN_1772627	D4S234E	NA	NA	NA	NA	NA	NA	NA	NA	NA	0,0466881
ILMN_1712413	RPL39L	NA	NA	NA	NA	NA	NA	NA	NA	NA	0,0467968
ILMN_1758846	ANKRD40	NA	NA	NA	NA	NA	NA	NA	NA	NA	0,04682835
ILMN_1701413	PIGQ	NA	NA	NA	NA	NA	NA	NA	NA	NA	0,04684087
ILMN_3233589	LOC728467	NA	NA	NA	NA	NA	NA	NA	NA	NA	0,04687232
ILMN_1725510	DHCR24	NA	NA	NA	NA	NA	NA	NA	NA	NA	0,04687796
ILMN_1702783	LOC652595	NA	NA	NA	NA	NA	NA	NA	NA	NA	0,04691543
ILMN_1763144	NEU1	NA	NA	0,002997366	NA	NA	NA	NA	NA	NA	0,04700667
ILMN_1708101	LMNB2	NA	NA	NA	NA	NA	NA	NA	NA	NA	0,04703993
ILMN_2363231	SMAP1	NA	NA	NA	NA	NA	NA	NA	NA	NA	0,0470951
ILMN_1796801	ABCA8	NA	NA	NA	NA	NA	NA	NA	NA	NA	0,0471738
ILMN_1664608	INPP5A	NA	NA	NA	NA	NA	NA	NA	NA	NA	0,04719736
ILMN_3236112	TP53TG1	NA	NA	NA	NA	NA	NA	NA	NA	NA	0,04720215
ILMN_1700306	OCIAD2	NA	NA	NA	NA	NA	NA	NA	NA	NA	0,0473418
ILMN_1664776	EFR3A	0,004225186	NA	0,001144014	NA	NA	NA	NA	NA	0,001967907	0,04736165
ILMN_2047885	PCDHB9	NA	NA	NA	NA	0,008396919	NA	NA	NA	NA	0,04738741
ILMN_1798123	ELOVL1	NA	NA	NA	NA	NA	NA	NA	3,15E-06	NA	0,04739938
ILMN_1672940	ZNF562	NA	NA	NA	NA	NA	NA	NA	NA	NA	0,04750123
ILMN_3235013	LOC730288	NA	NA	NA	NA	0,009622385	NA	NA	NA	NA	0,04750241
ILMN_2394552	WNK3	NA	NA	NA	NA	NA	NA	NA	NA	NA	0,04750503
ILMN_1679929	KLF13	NA	NA	NA	NA	NA	NA	NA	NA	NA	0,04753618
ILMN_1663538	CLYBL	NA	NA	0,003429523	NA	0,009445019	NA	NA	NA	NA	0,04761373
ILMN_1673522	MOCOS	NA	0,00347918	NA	NA	NA	NA	NA	NA	NA	0,04770199
ILMN_1654690	CECR5	NA	NA	NA	NA	NA	NA	NA	NA	NA	0,04776018
ILMN_1741431	AOC2	NA	NA	NA	NA	NA	NA	NA	0,002357859	NA	0,04780495
ILMN_1658576	PTPRN	NA	NA	NA	NA	NA	NA	NA	9,27E-05	NA	0,04781514
ILMN_3193306	C14orf109	NA	NA	NA	NA	NA	NA	NA	NA	NA	0,04781524
ILMN_2378100	FBXL5	NA	NA	NA	NA	NA	NA	NA	NA	NA	0,04781587
ILMN_3251298	THNSL1	NA	0,00122017	NA	NA	NA	NA	NA	NA	NA	0,04784293
ILMN_2069593	SFRS2IP	NA	NA	NA	NA	NA	NA	NA	NA	NA	0,04787521
ILMN_1666739	RBM15	NA	NA	NA	NA	NA	NA	NA	NA	NA	0,04789313
ILMN_1681203	TWF1	NA	NA	NA	NA	NA	NA	NA	NA	NA	0,04796731
ILMN_1676899	YEATS2	NA	NA	NA	NA	NA	NA	NA	0,0056397	NA	0,04806012
ILMN_1691731	PARP14	NA	NA	NA	NA	NA	NA	NA	NA	NA	0,0481391
ILMN_2138745	C14orf37	NA	NA	NA	NA	NA	NA	NA	NA	NA	0,04815947
ILMN_1813399	ATP2B1	NA	NA	NA	NA	NA	NA	NA	NA	NA	0,04826396
ILMN_1683598	ACSL4	NA	NA	NA	NA	NA	NA	NA	NA	NA	0,04829944
ILMN_1791106	HEATR6	NA	NA	0,004782587	NA	NA	NA	NA	NA	NA	0,04832291
ILMN_1782890	SLC25A3	NA	NA	NA	NA	NA	NA	NA	0,008906163	NA	0,04837833

ILMN_1714197	ACSS2	NA	NA	NA	NA	NA	NA	NA	0,00093911	NA	0,04840395
ILMN_1710937	IFI16	NA	NA	0,008583934	NA	0,003635528	NA	NA	NA	NA	0,04845703
ILMN_1781764	HNRNPH2	NA	NA	NA	NA	NA	NA	NA	NA	NA	0,04846971
ILMN_1786335	DUOX2	NA	NA	NA	NA	NA	NA	NA	NA	NA	0,04849567
ILMN_1751743	XRCC1	NA	NA	NA	NA	NA	NA	NA	NA	NA	0,04850063
ILMN_2115154	NUPL2	NA	NA	NA	NA	NA	NA	NA	NA	NA	0,04857869
ILMN_1803953	LOC388789	NA	NA	0,008827749	NA	NA	NA	NA	NA	NA	0,04862567
ILMN_2049727	C20orf111	NA	NA	NA	NA	NA	NA	NA	NA	NA	0,04863332
ILMN_2119486	DMTF1	0,001358215	NA	NA	NA	NA	NA	NA	NA	NA	0,04872354
ILMN_1672605	C7orf41	NA	0,005499195	NA	NA	NA	NA	NA	NA	NA	0,04875523
ILMN_3223181	SEC13	NA	NA	NA	NA	NA	NA	NA	NA	NA	0,04881339
ILMN_1807833	HM13	NA	NA	NA	NA	NA	NA	NA	NA	NA	0,04886731
ILMN_1761450	DHRS4L2	NA	0,000899704	NA	NA	0,005910461	NA	NA	NA	NA	0,04900284
ILMN_2358919	TP53I3	NA	NA	NA	NA	NA	NA	NA	NA	NA	0,0490659
ILMN_2364072	CLCNKA	3,30E-05	NA	0,000160665	NA	0,009584198	NA	NA	NA	NA	0,0490707
ILMN_1683498	LOC441034	NA	NA	0,002993789	NA	NA	NA	NA	NA	NA	0,04912321
ILMN_1719199	TULP3	NA	NA	NA	NA	NA	NA	NA	0,005761957	NA	0,04913415
ILMN_1804248	FDPS	NA	NA	NA	NA	NA	NA	NA	0,009738036	NA	0,04917983
ILMN_1709674	GFPT2	NA	NA	NA	NA	NA	NA	NA	NA	NA	0,04921588
ILMN_1765409	STAM	NA	NA	NA	NA	NA	NA	NA	NA	0,00882295	0,04930491
ILMN_1670134	FADS1	NA	0,004724742	NA	NA	NA	NA	NA	NA	NA	0,04931182
ILMN_1732923	SIPA1L2	0,008377476	NA	0,00163723	NA	NA	NA	NA	NA	0,009747398	0,04931587
ILMN_1790202	C1orf35	NA	NA	NA	NA	NA	NA	NA	NA	NA	0,04932225
ILMN_1697544	SLC25A29	NA	NA	NA	NA	NA	NA	NA	NA	NA	0,04940491
ILMN_3247906	RNF114	NA	NA	NA	NA	NA	NA	NA	NA	NA	0,04941996
ILMN_2128750	PTTG1IP	NA	NA	NA	NA	NA	NA	NA	NA	NA	0,0494912
ILMN_2077886	C1orf109	NA	NA	NA	NA	NA	NA	NA	NA	NA	0,04953208
ILMN_1762262	PKIA	NA	NA	NA	NA	NA	NA	NA	0,005951025	NA	0,0496287
ILMN_2054297	PTGS2	NA	NA	NA	NA	NA	NA	NA	NA	NA	0,04969943
ILMN_2355463	CYFIP1	NA	NA	NA	NA	NA	NA	NA	NA	NA	0,04987571
ILMN_1696494	CMTM6	NA	0,00048299	NA	NA	NA	NA	NA	NA	NA	0,0499248
ILMN_1692163	NSDHL	NA	NA	NA	NA	NA	NA	NA	0,005553569	NA	0,04993906
ILMN_1746516	RPS25	NA	NA	NA	NA	NA	NA	NA	NA	NA	0,04998737
ILMN_2307903	VCAM1	0,003788619	NA	NA	NA	NA	NA	NA	NA	0,000323887	NA
ILMN_2306565	MTX2	NA	NA	NA	NA	NA	NA	NA	NA	0,000347964	NA
ILMN_1791057	IFNAR2	0,004635332	NA	NA	NA	NA	NA	NA	NA	0,000362612	NA
ILMN_1758673	SLC44A1	NA	NA	0,008760304	NA	NA	NA	NA	NA	0,000367841	NA
ILMN_3201658	LOC642585	NA	NA	NA	NA	NA	NA	NA	NA	0,000610237	NA
ILMN_1728256	SPAG9	NA	NA	NA	NA	NA	NA	NA	NA	0,000659952	NA
ILMN_2230998	TMEM186	0,000135454	NA	NA	NA	NA	NA	NA	NA	0,000759917	NA
ILMN_1715680	NEIL2	NA	NA	NA	NA	NA	NA	NA	NA	0,000777548	NA
ILMN_1803590	ZFC3H1	NA	NA	NA	NA	NA	NA	NA	NA	0,000812449	NA
ILMN_3272996	LOC1001291	0,00035918	NA	0,008721078	NA	NA	NA	NA	NA	0,000821361	NA
ILMN_2189424	MRPL20	NA	NA	0,003800544	NA	NA	NA	NA	NA	0,00087049	NA
ILMN_3191636	LOC1001287	0,003149267	NA	NA	NA	NA	NA	NA	NA	0,000916662	NA
ILMN_1713966	MRPL52	0,000203428	NA	NA	NA	NA	NA	NA	NA	0,000941125	NA
ILMN_2336109	L3MBTL2	NA	NA	NA	NA	NA	NA	NA	NA	0,0010647	NA
ILMN_1800912	ZFP106	NA	NA	NA	NA	NA	NA	NA	NA	0,001135073	NA
ILMN_1766955	VCAM1	0,003004835	NA	NA	NA	NA	NA	NA	NA	0,00114619	NA
ILMN_2152429	C12orf76	0,005284538	NA	NA	NA	NA	NA	NA	NA	0,001216319	NA
ILMN_1678680	DBR1	0,007722395	NA	NA	NA	NA	NA	NA	NA	0,00133423	NA
ILMN_1722674	MGC3196	NA	NA	NA	NA	NA	NA	NA	NA	0,001404179	NA
ILMN_1793673	ZNF766	0,00382984	NA	NA	NA	NA	NA	NA	NA	0,001517347	NA
ILMN_1717099	DSCR3	0,004101738	NA	NA	NA	NA	NA	NA	NA	0,001549379	NA
ILMN_3307786	L3MBTL2	NA	NA	NA	NA	NA	NA	NA	NA	0,001589942	NA
ILMN_1664010	ELF1	0,004397248	NA	NA	NA	NA	NA	NA	NA	0,001597308	NA
ILMN_1681675	RBM16	0,003273838	NA	NA	NA	NA	NA	NA	NA	0,001663469	NA
ILMN_2204876	FLVCR2	3,79E-05	NA	0,003764852	NA	NA	NA	NA	NA	0,001809905	NA
ILMN_3247082	FAM150B	0,000489161	NA	0,000591684	NA	NA	NA	NA	NA	0,001822129	NA
ILMN_1802519	VPS36	NA	NA	NA	NA	NA	NA	NA	NA	0,001964406	NA
ILMN_1723689	RANBP3	NA	NA	NA	NA	NA	NA	NA	NA	0,002027162	NA
ILMN_1718042	ZNF549	0,000401978	NA	NA	NA	NA	NA	NA	NA	0,002104181	NA
ILMN_1774659	LOC652388	NA	NA	NA	NA	NA	NA	NA	NA	0,002156672	NA
ILMN_2089752	LOC285016	0,000346671	NA	0,000298151	NA	NA	NA	NA	NA	0,002243985	NA
ILMN_1741841	KIAA1429	0,002230442	NA	NA	NA	NA	NA	NA	NA	0,00235861	NA
ILMN_1770623	FAM58A	0,006952771	NA	NA	NA	NA	NA	NA	NA	0,002478307	NA
ILMN_1776352	MUTED	0,000401417	NA	NA	NA	NA	NA	NA	NA	0,002483943	NA
ILMN_1754114	FLJ20021	NA	NA	NA	NA	NA	NA	NA	NA	0,00252788	NA
ILMN_1735552	KIF1B	0,000785626	NA	NA	NA	NA	NA	NA	NA	0,002566616	NA
ILMN_1738704	TRIM26	0,002698893	NA	NA	NA	NA	NA	NA	NA	0,002624054	NA
ILMN_3183517	LOC1001308	NA	NA	0,005665098	NA	NA	NA	NA	NA	0,002662428	NA
ILMN_3262439	FLJ46552	NA	NA	NA	NA	NA	NA	NA	NA	0,00280724	NA
ILMN_1667893	TNS3	2,51E-05	NA	0,005250785	NA	NA	NA	NA	NA	0,002868496	NA
ILMN_2083469	IRS2	NA	NA	0,002721065	NA	NA	NA	NA	NA	0,002931878	NA
ILMN_1814859	DDX47	NA	NA	NA	NA	NA	NA	NA	NA	0,002971648	NA
ILMN_1795228	ZFAND5	NA	NA	NA	NA	NA	NA	NA	NA	0,003028059	NA
ILMN_1773427	KANK1	0,007985058	NA	NA	NA	NA	NA	NA	NA	0,003183426	NA
ILMN_2149053	RIF1	NA	NA	NA	NA	NA	NA	NA	NA	0,003195624	NA
ILMN_1676844	NR1D2	5,53E-05	NA	NA	NA	NA	NA	NA	NA	0,003311991	NA
ILMN_2221564	LYAR	0,002811329	NA	NA	NA	NA	NA	NA	NA	0,003494409	NA
ILMN_1651538	NUMBL	NA	NA	NA	NA	NA	NA	NA	NA	0,003562365	NA
ILMN_1742618	XAF1	NA	NA	NA	NA	NA	NA	NA	NA	0,003571903	NA
ILMN_1664855	PPP1R14C	0,0003351	NA	NA	NA	NA	NA	NA	NA	0,003585896	NA
ILMN_1738523	MYD88	NA	NA	0,007151236	0,00805264	NA	NA	NA	NA	0,003630012	NA
ILMN_1780582	CD83	NA	NA	NA	NA	NA	NA	NA	NA	0,003715885	NA
ILMN_1681703	FOXO3	NA	NA	0,007051065	NA	NA	NA	NA	NA	0,003871124	NA
ILMN_1738326	EIF4E2	0,001207778	NA	NA	NA	NA	NA	NA	NA	0,003873423	NA
ILMN_1688346	ZNF800	NA	NA	NA	NA	NA	NA	NA	NA	0,003904846	NA
ILMN_1653404	NKIRAS2	0,004189916	NA	0,006910691	NA	NA	NA	NA	NA	0,003936644	NA
ILMN_1678075	CDYL	NA	NA	NA	NA	NA	NA	NA	NA	0,003968344	NA
ILMN_1713964	BTBD3	NA	NA	NA	NA	NA	NA	NA	NA	0,003985567	NA
ILMN_1718303	PVRL2	2,11E-05	NA	0,002680597	NA	NA	NA	NA	NA	0,004003118	NA
ILMN_1698402	NFX1	0,00297259	NA	0,004736607	NA	NA	NA	NA	NA	0,004035322	NA
ILMN_1821724		NA	NA	NA	NA	NA	NA	NA	NA	0,004046579	NA
ILMN_1725312	RASA1	NA	NA	NA	NA	NA	NA	NA	NA	0,004072981	NA
ILMN_1862217		NA	NA	NA	NA	NA	NA	NA	NA	0,004268852	NA
ILMN_1792072	FUT4	0,006248176	NA	NA	NA	NA	NA	NA	NA	0,004383879	NA
ILMN_1739454	USP34	0,008128585	NA	NA	NA	NA	NA	NA	NA	0,004407672	NA
ILMN_1751803	LSM10	NA	NA	NA	NA	NA	NA	NA	NA	0,004444781	NA
ILMN_2106449	PLEKHF2	NA	NA	0,001764911	NA	NA	NA	NA	NA	0,004598767	NA
ILMN_1657470	YTHDF3	NA	NA	NA	NA	NA	NA	NA	NA	0,004621624	NA
ILMN_1787539	ZNF177	NA	NA	NA	NA	NA	NA	NA	NA	0,004640058	NA
ILMN_1856609		NA	NA	NA	NA	NA	NA	NA	NA	0,004709698	NA
ILMN_1740505	SGMS1	0,001079282	NA	0,000808127	NA	NA	NA	NA	NA	0,004809289	NA
ILMN_1737947	LSM5	NA	NA	NA	NA	NA	NA	NA	NA	0,004833992	NA
ILMN_1736015	PHF17	0,001126289	NA	NA	NA	NA	NA	NA	NA	0,004893473	NA
ILMN_1677432	SRGAP1	0,009992629	NA	NA	NA	NA	NA	NA	NA	0,004918137	NA
ILMN_1784860	RFC3	0,007778587	NA	NA	NA	NA	NA	NA	NA	0,005090137	NA
ILMN_2061185	SH2B1	NA	NA	NA	NA	NA	NA	NA	NA	0,00509054	NA

ILMN_2344047	ZNF398	NA	NA	NA	NA	NA	NA	NA	NA	0,0051552	NA
ILMN_1815141	LOC149448	NA	NA	NA	NA	NA	NA	NA	NA	0,00549245	NA
ILMN_2374778	DUT	0,003509706	NA	NA	NA	NA	NA	NA	NA	0,005577802	NA
ILMN_1681949	PDGFRA	NA	NA	NA	NA	NA	NA	NA	NA	0,005609908	NA
ILMN_1682147	HOOK2	NA	NA	NA	0,00068879	NA	NA	NA	NA	0,005651352	NA
ILMN_1849013		NA	NA	NA	NA	NA	NA	NA	NA	0,005742508	NA
ILMN_1689831	FLJ11235	NA	NA	NA	NA	NA	NA	NA	NA	0,005756322	NA
ILMN_1723632	PIGC	NA	NA	NA	NA	NA	NA	NA	NA	0,005824286	NA
ILMN_1652749	ERF	NA	NA	NA	NA	NA	NA	NA	NA	0,00587324	NA
ILMN_1741985	BBS10	NA	NA	NA	NA	NA	NA	NA	NA	0,006047268	NA
ILMN_1804117	FAM89B	0,00014051	NA	NA	NA	NA	NA	NA	NA	0,006047458	NA
ILMN_1755303	ZNF217	NA	NA	NA	NA	NA	NA	NA	NA	0,006131273	NA
ILMN_1756942	SP3	NA	NA	NA	NA	NA	NA	NA	NA	0,006209489	NA
ILMN_1735594	CDC42SE2	0,001171667	NA	NA	NA	NA	NA	NA	NA	0,006344507	NA
ILMN_1737164	TM9SF1	0,007311074	NA	NA	NA	NA	NA	NA	NA	0,006346777	NA
ILMN_1728349	TMEM63B	0,004779652	NA	NA	NA	NA	NA	NA	NA	0,006406288	NA
ILMN_1777322	FAM91A1	NA	NA	NA	NA	NA	NA	NA	NA	0,006576567	NA
ILMN_3240721	LOC645233	0,000304693	NA	NA	NA	NA	NA	NA	NA	0,006661162	NA
ILMN_1656335	RIT1	0,000525349	NA	0,002456062	NA	NA	NA	NA	NA	0,006756131	NA
ILMN_2046024	DUSP11	NA	NA	NA	NA	NA	NA	NA	NA	0,006798702	NA
ILMN_1782331	TDG	0,000678732	NA	NA	NA	NA	NA	NA	NA	0,006861585	NA
ILMN_1704656	PPP2R1B	NA	NA	NA	NA	NA	NA	NA	NA	0,006922512	NA
ILMN_1801121	SENP2	NA	NA	NA	NA	NA	NA	NA	NA	0,006941251	NA
ILMN_1700834	SLK	NA	NA	NA	NA	NA	NA	NA	NA	0,00696505	NA
ILMN_1732336	RFC2	NA	NA	NA	NA	NA	NA	NA	NA	0,007046537	NA
ILMN_1867188		NA	NA	NA	NA	NA	NA	NA	NA	0,00708105	NA
ILMN_1789596	ETV6	0,00147087	NA	NA	NA	NA	NA	NA	NA	0,007134452	NA
ILMN_1709611	PSMA1	0,008441585	NA	NA	NA	NA	NA	NA	NA	0,007178498	NA
ILMN_2380605	MTMR3	NA	NA	NA	NA	NA	NA	NA	NA	0,007193593	NA
ILMN_1734316	YME1L1	0,005048842	NA	NA	NA	NA	NA	NA	NA	0,007239237	NA
ILMN_2405305	ARNTL	NA	NA	NA	NA	NA	NA	NA	NA	0,007313562	NA
ILMN_3287266	LOC1001333	NA	NA	NA	NA	NA	NA	NA	NA	0,007426315	NA
ILMN_1737992	CRAT	0,004246068	NA	NA	NA	NA	NA	NA	NA	0,007448846	NA
ILMN_1652024	CSNK1G3	NA	NA	NA	NA	NA	NA	NA	NA	0,007545928	NA
ILMN_1733615	MTF2	0,004238483	NA	NA	NA	NA	NA	NA	NA	0,007549835	NA
ILMN_2086417	NDUFV2	0,001574118	NA	NA	NA	NA	NA	NA	NA	0,007562907	NA
ILMN_1755727	KDM5B	0,004214984	NA	NA	NA	NA	NA	NA	NA	0,007568785	NA
ILMN_3239531	C4orf48	NA	NA	NA	NA	NA	NA	NA	NA	0,007711741	NA
ILMN_1682495	FOXP1	NA	NA	NA	NA	NA	NA	NA	NA	0,007861724	NA
ILMN_1731412	UBTD2	NA	NA	NA	NA	NA	NA	NA	NA	0,007933197	NA
ILMN_1684289	PNPO	5,16E-05	NA	NA	NA	NA	NA	NA	NA	0,007986868	NA
ILMN_3275575	LOC648980	0,000196502	NA	NA	NA	NA	NA	NA	NA	0,008193019	NA
ILMN_2392143	TRAF6	NA	NA	NA	NA	NA	NA	NA	NA	0,008232831	NA
ILMN_1664216	NKIRAS1	NA	NA	0,006442264	NA	NA	NA	NA	NA	0,008269769	NA
ILMN_1677113	RNF8	0,003682998	NA	NA	NA	NA	NA	NA	NA	0,008342731	NA
ILMN_1749466	VAT1L	0,002664137	NA	NA	NA	NA	NA	NA	NA	0,008378225	NA
ILMN_2339284	CHD2	0,00465294	NA	NA	NA	NA	NA	NA	NA	0,008476871	NA
ILMN_1768798	SPAG9	NA	NA	NA	NA	NA	NA	NA	NA	0,008507773	NA
ILMN_3238712	SNRNP25	NA	NA	NA	NA	NA	NA	NA	NA	0,008570747	NA
ILMN_1695509	PTPN12	NA	NA	NA	NA	NA	NA	NA	NA	0,008583189	NA
ILMN_2246661	FAHD1	0,007795614	NA	NA	NA	NA	NA	NA	NA	0,008597652	NA
ILMN_1675844	WDR1	0,009384874	NA	NA	NA	NA	NA	NA	NA	0,0086393	NA
ILMN_1747162	DDX47	0,002385659	NA	NA	NA	NA	NA	NA	NA	0,008662143	NA
ILMN_1660498	RPS27	0,004059585	NA	NA	NA	NA	NA	NA	NA	0,008746442	NA
ILMN_1748432	ZNF525	6,00E-05	NA	NA	NA	NA	NA	NA	NA	0,00878192	NA
ILMN_1776375	PIN1	0,00025767	NA	0,006589013	NA	NA	NA	NA	NA	0,009027096	NA
ILMN_1669094	ZNF672	0,005194352	NA	NA	NA	NA	NA	NA	NA	0,009066516	NA
ILMN_1798602	PCF11	NA	NA	NA	NA	NA	NA	NA	NA	0,009123389	NA
ILMN_1709124	ANO8	NA	NA	NA	NA	NA	NA	NA	NA	0,009191977	NA
ILMN_1752741	TRIM23	NA	NA	NA	NA	NA	NA	NA	NA	0,009235642	NA
ILMN_1700477	MRPL43	NA	NA	NA	NA	NA	NA	NA	NA	0,009261422	NA
ILMN_1777118	INTS9	0,006240359	NA	0,003450612	NA	NA	NA	NA	NA	0,009441053	NA
ILMN_1763688	C17orf49	0,00448761	NA	NA	NA	NA	NA	NA	NA	0,009539023	NA
ILMN_1773763	MTA2	0,004025913	NA	0,009796435	NA	NA	NA	NA	NA	0,009577929	NA
ILMN_2332267	CSNK1G3	0,000772323	NA	0,007950565	NA	NA	NA	NA	NA	0,009603872	NA
ILMN_2313856	OSBPL9	NA	0,009315292	NA	NA	NA	NA	NA	NA	0,009607179	NA
ILMN_1714444	KLF12	NA	NA	NA	NA	NA	NA	NA	NA	0,009627378	NA
ILMN_3275489	LOC1001319	0,004895005	NA	0,002874072	NA	NA	NA	NA	NA	0,009629929	NA
ILMN_1819805		NA	NA	NA	NA	NA	NA	NA	NA	0,00966271	NA
ILMN_2328666	CD83	NA	NA	NA	NA	NA	NA	NA	NA	0,009779532	NA
ILMN_1695432	TPST2	NA	NA	NA	NA	NA	NA	NA	NA	0,009879951	NA
ILMN_1750401	C17orf62	NA	NA	NA	NA	NA	NA	NA	NA	0,009927211	NA
ILMN_2374692	WAC	NA	NA	NA	NA	NA	NA	NA	3,90E-06	NA	NA
ILMN_1795715	DPYD	NA	NA	NA	NA	NA	NA	NA	5,88E-06	NA	NA
ILMN_2386973	PKP2	NA	NA	NA	NA	NA	NA	NA	9,59E-06	NA	NA
ILMN_2404407	ABAT	NA	NA	NA	NA	NA	NA	NA	1,33E-05	NA	NA
ILMN_1711807	LPHN3	NA	NA	NA	NA	NA	NA	NA	1,77E-05	NA	NA
ILMN_1733164	FBXO11	NA	NA	NA	NA	NA	NA	NA	3,08E-05	NA	NA
ILMN_2202948	BUB1	NA	NA	NA	NA	NA	NA	NA	4,27E-05	NA	NA
ILMN_1653466	HES4	NA	NA	NA	NA	NA	NA	NA	4,66E-05	NA	NA
ILMN_2053679	ACADM	NA	NA	NA	NA	NA	NA	NA	7,73E-05	NA	NA
ILMN_1738491	SNX30	NA	NA	NA	NA	NA	NA	NA	8,90E-05	NA	NA
ILMN_1684038	CLIP1	NA	NA	NA	NA	NA	NA	NA	0,000108438	NA	NA
ILMN_1724333	TOR1B	NA	NA	NA	NA	NA	NA	NA	0,000113207	NA	NA
ILMN_1806692	HEXB	NA	NA	NA	NA	NA	NA	NA	0,000131452	NA	NA
ILMN_1706957	BMPR1A	NA	NA	NA	NA	NA	NA	NA	0,000142201	NA	NA
ILMN_1802888	ZNF185	NA	NA	NA	NA	NA	NA	NA	0,00017178	NA	NA
ILMN_1659885	ACSS3	NA	NA	NA	NA	NA	NA	NA	0,000186671	NA	NA
ILMN_1716913	TRAPPC1	NA	NA	NA	NA	NA	NA	NA	0,000187561	NA	NA
ILMN_2091347	IDH1	NA	NA	NA	NA	NA	NA	NA	0,000188132	NA	NA
ILMN_2123567	SENP2	NA	NA	NA	NA	NA	NA	NA	0,000195168	NA	NA
ILMN_1887357		NA	NA	NA	NA	NA	NA	NA	0,000199643	NA	NA
ILMN_2174884	XPO7	NA	NA	NA	NA	NA	NA	NA	0,000204647	NA	NA
ILMN_1715173	RPS6KA1	NA	NA	NA	NA	NA	NA	NA	0,000219949	NA	NA
ILMN_1667330	LOC113386	NA	NA	NA	NA	NA	NA	NA	0,000244485	NA	NA
ILMN_2390609	ANK3	NA	NA	NA	NA	NA	NA	NA	0,000245975	NA	NA
ILMN_1672176	SMPD2	NA	NA	NA	NA	NA	NA	NA	0,000293169	NA	NA
ILMN_1806463	CTAGE6	NA	NA	NA	NA	NA	NA	NA	0,000326037	NA	NA
ILMN_1708502	AFF4	NA	NA	NA	NA	NA	NA	NA	0,000327559	NA	NA
ILMN_1746598	SCNM1	NA	NA	NA	NA	NA	NA	NA	0,000328202	NA	NA
ILMN_1716816	TMEM87A	NA	NA	NA	NA	NA	NA	NA	0,000342231	NA	NA
ILMN_1761281	LOC441019	NA	NA	NA	NA	NA	NA	NA	0,000363468	NA	NA
ILMN_1743432	DGUOK	NA	NA	NA	NA	NA	NA	NA	0,000369677	NA	NA
ILMN_2376502	RHOBTB1	NA	NA	NA	NA	NA	NA	NA	0,000381573	NA	NA
ILMN_2207363	RABAC1	NA	NA	NA	NA	NA	NA	NA	0,000382136	NA	NA
ILMN_1784753	PAIP2	NA	NA	NA	NA	NA	NA	NA	0,000382161	NA	NA
ILMN_1669033	NCOA1	NA	NA	NA	NA	NA	NA	NA	0,000395451	NA	NA
ILMN_1802553	MRPS24	NA	NA	NA	NA	NA	NA	NA	0,000405403	NA	NA
ILMN_2306066	FUS	NA	NA	NA	NA	NA	NA	NA	0,000418513	NA	NA

ILMN_1664006	AGL	NA	NA	NA	NA	NA	NA	NA	0,000422968	NA	NA
ILMN_1679797	ADARB1	NA	NA	NA	NA	NA	NA	NA	0,000424196	NA	NA
ILMN_1667551	GUSBL1	NA	NA	NA	NA	NA	NA	NA	0,000428063	NA	NA
ILMN_1701855	PPP1CC	NA	NA	NA	NA	NA	NA	NA	0,000437494	NA	NA
ILMN_2404085	CLIP1	NA	NA	NA	NA	NA	NA	NA	0,000451466	NA	NA
ILMN_1779558	GAS6	NA	NA	NA	NA	NA	NA	NA	0,000457538	NA	NA
ILMN_1664371	HIATL1	NA	NA	NA	NA	NA	NA	NA	0,000480626	NA	NA
ILMN_1652846	PCYT2	NA	NA	NA	NA	NA	NA	NA	0,000483849	NA	NA
ILMN_1806787	CSDC2	NA	NA	NA	NA	NA	NA	NA	0,000506436	NA	NA
ILMN_1703441	ZNF593	NA	NA	NA	NA	NA	NA	NA	0,000516978	NA	NA
ILMN_1755741	DACH1	NA	NA	NA	NA	NA	NA	NA	0,000518505	NA	NA
ILMN_1687556	DAPL1	NA	NA	NA	NA	NA	NA	NA	0,000529537	NA	NA
ILMN_2043615	C17orf90	NA	NA	NA	NA	NA	NA	NA	0,000544041	NA	NA
ILMN_1693410	BRI3BP	NA	NA	NA	NA	NA	NA	NA	0,000548619	NA	NA
ILMN_1659913	ISG20	NA	NA	NA	NA	NA	NA	NA	0,000557142	NA	NA
ILMN_2352121	NT5C3	NA	NA	NA	NA	NA	NA	NA	0,000564087	NA	NA
ILMN_3264466	FAM54B	NA	NA	NA	NA	NA	NA	NA	0,000583844	NA	NA
ILMN_2352036	RTN4	NA	NA	NA	NA	NA	NA	NA	0,000589451	NA	NA
ILMN_1703379	VPS41	NA	NA	NA	NA	NA	NA	NA	0,000603646	NA	NA
ILMN_1749792	SORBS1	NA	NA	NA	NA	NA	NA	NA	0,000603836	NA	NA
ILMN_1694799	PIAS2	NA	NA	NA	NA	NA	NA	NA	0,000607801	NA	NA
ILMN_1772719	GPN1	NA	NA	NA	NA	NA	NA	NA	0,000627077	NA	NA
ILMN_1746265	SARM1	NA	NA	NA	NA	NA	NA	NA	0,000627106	NA	NA
ILMN_1805104	ABAT	NA	NA	NA	NA	NA	NA	NA	0,000711473	NA	NA
ILMN_3291352	LOC643778	NA	NA	NA	NA	NA	NA	NA	0,000749023	NA	NA
ILMN_1726466	HDHD3	NA	NA	NA	NA	NA	NA	NA	0,000751244	NA	NA
ILMN_2404657	ALAD	NA	NA	NA	NA	NA	NA	NA	0,00077496	NA	NA
ILMN_1690342	LTA4H	NA	NA	NA	NA	NA	NA	NA	0,000777223	NA	NA
ILMN_2129877	PARP11	NA	NA	NA	NA	NA	NA	NA	0,000780752	NA	NA
ILMN_1736834	SKIL	NA	NA	NA	NA	NA	NA	NA	0,000789803	NA	NA
ILMN_2299843	ATP5S	NA	NA	NA	NA	NA	NA	NA	0,000798077	NA	NA
ILMN_1669273	PPT1	NA	NA	NA	NA	NA	NA	NA	0,000798739	NA	NA
ILMN_2307025	CPNE1	NA	NA	NA	NA	NA	NA	NA	0,000805766	NA	NA
ILMN_1742025	OLFM1	NA	NA	NA	NA	NA	NA	NA	0,000819708	NA	NA
ILMN_1801941	C1orf50	NA	NA	NA	NA	NA	NA	NA	0,000828133	NA	NA
ILMN_1665737	UFD1L	NA	NA	NA	NA	NA	NA	NA	0,000831065	NA	NA
ILMN_2152502	MGC72080	NA	NA	NA	NA	NA	NA	NA	0,000832651	NA	NA
ILMN_2306955	ACPL2	NA	NA	NA	NA	NA	NA	NA	0,000862185	NA	NA
ILMN_1715401	MT1G	NA	NA	NA	NA	NA	NA	NA	0,000878937	NA	NA
ILMN_1731609	CHMP6	NA	NA	NA	NA	NA	NA	NA	0,000920092	NA	NA
ILMN_2336803	RPGR	NA	NA	NA	NA	NA	NA	NA	0,000924491	NA	NA
ILMN_2370872	GRINA	NA	NA	NA	NA	NA	NA	NA	0,000957711	NA	NA
ILMN_1766054	ABCA1	NA	NA	NA	NA	NA	NA	NA	0,001020163	NA	NA
ILMN_1754235	SLC35C2	NA	NA	NA	NA	NA	NA	NA	0,00102859	NA	NA
ILMN_2136089	MTE	NA	NA	NA	NA	NA	NA	NA	0,001034879	NA	NA
ILMN_2350183	ST5	NA	NA	NA	NA	NA	NA	NA	0,001063841	NA	NA
ILMN_1810782	SH3KBP1	NA	NA	NA	NA	NA	NA	NA	0,001080114	NA	NA
ILMN_1653039	LOC642934	NA	NA	NA	NA	NA	NA	NA	0,001086163	NA	NA
ILMN_2415949	MRRF	NA	NA	NA	NA	NA	NA	NA	0,001090442	NA	NA
ILMN_2128770	CDR2L	NA	NA	NA	NA	NA	NA	NA	0,001096154	NA	NA
ILMN_3211746	LOC1001311	NA	NA	NA	NA	NA	NA	NA	0,00113	NA	NA
ILMN_1754864	SLC25A18	NA	NA	NA	NA	NA	NA	NA	0,001134161	NA	NA
ILMN_1678037	HIRIP3	NA	NA	NA	NA	NA	NA	NA	0,001148622	NA	NA
ILMN_2411963	RBM39	NA	NA	NA	NA	NA	NA	NA	0,001157728	NA	NA
ILMN_2178587	ANKRD6	NA	NA	NA	NA	NA	NA	NA	0,001179865	NA	NA
ILMN_1705468	PIK3CA	NA	NA	NA	NA	NA	NA	NA	0,001190934	NA	NA
ILMN_1742187	MAN1A1	NA	NA	NA	NA	NA	NA	NA	0,001202752	NA	NA
ILMN_1653718	ZFAND2B	NA	NA	NA	NA	NA	NA	NA	0,001223221	NA	NA
ILMN_1687864	POP5	NA	NA	NA	NA	NA	NA	NA	0,001235238	NA	NA
ILMN_1680091	POP7	NA	NA	NA	NA	NA	NA	NA	0,001247123	NA	NA
ILMN_1794468	QPCTL	NA	NA	NA	NA	NA	NA	NA	0,001284474	NA	NA
ILMN_1731064	CABC1	NA	NA	NA	NA	NA	NA	NA	0,001353711	NA	NA
ILMN_1794950	RGAG1	NA	NA	NA	NA	NA	NA	NA	0,001355545	NA	NA
ILMN_2104924	C21orf70	NA	NA	NA	NA	NA	NA	NA	0,001389608	NA	NA
ILMN_1678678	SLC37A4	NA	NA	NA	NA	NA	NA	NA	0,001405778	NA	NA
ILMN_2228035	HS3ST1	NA	NA	NA	NA	NA	NA	NA	0,00140834	NA	NA
ILMN_1793384	JAK1	NA	NA	NA	NA	NA	NA	NA	0,001415851	NA	NA
ILMN_1765641	SEMA3A	NA	NA	NA	NA	NA	NA	NA	0,001428723	NA	NA
ILMN_1736847	MED8	NA	NA	NA	NA	NA	NA	NA	0,001433298	NA	NA
ILMN_1659769	MRAP	NA	NA	NA	NA	NA	NA	NA	0,001435397	NA	NA
ILMN_1687626	ZDHHC24	NA	NA	NA	NA	NA	NA	NA	0,001448711	NA	NA
ILMN_1725079	TSPAN31	NA	NA	NA	NA	NA	NA	NA	0,001469154	NA	NA
ILMN_3234081	LOC1001290	NA	NA	NA	NA	NA	NA	NA	0,001490494	NA	NA
ILMN_1800874	MRO	NA	NA	NA	NA	NA	NA	NA	0,001504526	NA	NA
ILMN_1659990	C7orf68	NA	NA	NA	NA	NA	NA	NA	0,001522079	NA	NA
ILMN_1777483	APOOL	NA	NA	NA	NA	NA	NA	NA	0,0015263	NA	NA
ILMN_1756999	RBL2	NA	NA	NA	NA	NA	NA	NA	0,001553798	NA	NA
ILMN_1658302	PIAS2	NA	NA	NA	NA	NA	NA	NA	0,001565282	NA	NA
ILMN_1795507	ABCA6	NA	NA	NA	NA	NA	NA	NA	0,001571247	NA	NA
ILMN_2280911	VPS41	NA	NA	NA	NA	NA	NA	NA	0,001572322	NA	NA
ILMN_3225894	LOC729774	NA	NA	NA	NA	NA	NA	NA	0,001584069	NA	NA
ILMN_1677198	C1R	NA	NA	NA	NA	NA	NA	NA	0,001616875	NA	NA
ILMN_1748911	SNAP23	NA	NA	NA	NA	NA	NA	NA	0,001622975	NA	NA
ILMN_2391264	DMKN	NA	NA	NA	NA	NA	NA	NA	0,001646945	NA	NA
ILMN_1800739	SPINT2	NA	NA	NA	0,00829686	NA	NA	NA	0,001648349	NA	NA
ILMN_1670638	PITPNC1	NA	NA	NA	NA	NA	NA	NA	0,001655632	NA	NA
ILMN_2231608	LOC441294	NA	NA	NA	NA	NA	NA	NA	0,001678965	NA	NA
ILMN_1664068	ERGIC1	NA	NA	NA	NA	NA	NA	NA	0,001684232	NA	NA
ILMN_2246083	C7orf28B	NA	NA	NA	NA	NA	NA	NA	0,001699119	NA	NA
ILMN_1718070	CASP9	NA	NA	NA	NA	NA	NA	NA	0,00170478	NA	NA
ILMN_1701681	SEC11C	NA	NA	NA	NA	NA	NA	NA	0,001706012	NA	NA
ILMN_2323385	TRIM4	NA	NA	NA	NA	NA	NA	NA	0,001706862	NA	NA
ILMN_1775170	MT1X	NA	NA	NA	NA	NA	NA	NA	0,00172653	NA	NA
ILMN_1673880	EFEMP1	NA	NA	NA	NA	NA	NA	NA	0,001739516	NA	NA
ILMN_1898404	CACNA1C	NA	NA	NA	NA	NA	NA	NA	0,001810362	NA	NA
ILMN_1738736	SNX4	NA	NA	NA	NA	NA	NA	NA	0,001836092	NA	NA
ILMN_1764109	C1R	NA	NA	NA	NA	NA	NA	NA	0,001855793	NA	NA
ILMN_1772821	KIAA1671	NA	NA	NA	NA	NA	NA	NA	0,001862149	NA	NA
ILMN_3248627	CHRM3	0,001093781	NA	NA	NA	NA	NA	NA	0,001874142	NA	NA
ILMN_3237329	FNIP2	0,006350097	NA	NA	NA	NA	NA	NA	0,001901356	NA	NA
ILMN_2113490	NTN4	NA	NA	NA	NA	NA	NA	NA	0,001902625	NA	NA
ILMN_1702759	TMX4	NA	NA	NA	NA	NA	NA	NA	0,001939768	NA	NA
ILMN_1781721	DDX31	NA	NA	NA	NA	NA	NA	NA	0,001942261	NA	NA
ILMN_1665775	MOSC2	NA	NA	NA	NA	NA	NA	NA	0,001949336	NA	NA
ILMN_1782257	METT11D1	NA	NA	NA	NA	NA	NA	NA	0,001960491	NA	NA
ILMN_1686679	ZNF462	NA	NA	NA	NA	NA	NA	NA	0,00196691	NA	NA
ILMN_1733869	OGDH	NA	NA	NA	NA	NA	NA	NA	0,001970623	NA	NA
ILMN_1778319	DMKN	NA	NA	NA	NA	NA	NA	NA	0,001982194	NA	NA
ILMN_1710410	LOC730413	0,000542488	NA	NA	NA	NA	NA	NA	0,001984325	NA	NA

ILMN_1671257	DKC1	NA	NA	NA	NA	NA	NA	NA	0,001999267	NA	NA
ILMN_2145997	SP4	NA	NA	NA	NA	NA	NA	NA	0,002007615	NA	NA
ILMN_3270411	LOC1001298	NA	NA	NA	NA	NA	NA	NA	0,002011118	NA	NA
ILMN_1674661	CIRBP	NA	NA	NA	NA	NA	NA	NA	0,0020157	NA	NA
ILMN_3263694	LOC1001287	NA	NA	NA	NA	NA	NA	NA	0,002017094	NA	NA
ILMN_1683576	MAGED2	NA	NA	NA	NA	NA	NA	NA	0,002031306	NA	NA
ILMN_1684446	SPAG7	NA	NA	NA	NA	NA	NA	NA	0,002066431	NA	NA
ILMN_1771949	TAF4B	NA	NA	NA	NA	NA	NA	NA	0,002071087	NA	NA
ILMN_1681304	PAN3	NA	NA	NA	NA	NA	NA	NA	0,002075472	NA	NA
ILMN_1713058	PSTPIP2	NA	NA	NA	NA	NA	NA	NA	0,002097169	NA	NA
ILMN_2366710	UCRC	NA	NA	NA	NA	NA	NA	NA	0,002101734	NA	NA
ILMN_1725594	FAM188A	NA	NA	NA	NA	NA	NA	NA	0,002108507	NA	NA
ILMN_1677765	LRP8	NA	NA	NA	NA	NA	NA	NA	0,002121022	NA	NA
ILMN_1777811	URG4	NA	NA	NA	NA	NA	NA	NA	0,002171803	NA	NA
ILMN_1724009	SETD6	NA	NA	NA	NA	NA	NA	NA	0,002179126	NA	NA
ILMN_1709162	SHCBP1	0,007694847	NA	NA	NA	NA	NA	NA	0,002180879	NA	NA
ILMN_1807662	IGF2R	NA	NA	NA	NA	NA	NA	NA	0,002184277	NA	NA
ILMN_2055700	SLBP	NA	NA	NA	NA	NA	NA	NA	0,002185858	NA	NA
ILMN_2232166	CCDC90B	NA	NA	NA	NA	NA	NA	NA	0,002199488	NA	NA
ILMN_1669523	FOS	NA	NA	NA	NA	NA	NA	NA	0,002224132	NA	NA
ILMN_1728049	S100A16	NA	NA	NA	NA	NA	NA	NA	0,002237032	NA	NA
ILMN_1742611	C1orf52	NA	NA	NA	0,00889081	NA	NA	NA	0,002253561	NA	NA
ILMN_1741176	CHMP2B	NA	NA	NA	NA	NA	NA	NA	0,002265802	NA	NA
ILMN_1656186	SLC41A1	NA	NA	NA	NA	NA	NA	NA	0,002278016	NA	NA
ILMN_1784749	GAS6	NA	NA	NA	NA	NA	NA	NA	0,002287823	NA	NA
ILMN_1663751	CYCSL1	NA	NA	NA	NA	NA	NA	NA	0,002309153	NA	NA
ILMN_2333367	FKBP1A	NA	NA	NA	NA	NA	NA	NA	0,00233031	NA	NA
ILMN_1797822	SEL1L3	NA	NA	NA	NA	NA	NA	NA	0,002330762	NA	NA
ILMN_2312296	PCBP2	NA	NA	NA	NA	NA	NA	NA	0,00235076	NA	NA
ILMN_2145250	NACAP1	NA	NA	NA	NA	NA	NA	NA	0,002356327	NA	NA
ILMN_2223130	SMARCA5	NA	NA	NA	NA	NA	NA	NA	0,002367298	NA	NA
ILMN_2383934	ITGB1	NA	NA	NA	NA	NA	NA	NA	0,002369657	NA	NA
ILMN_1697440	PRPF4	NA	NA	NA	NA	NA	NA	NA	0,002376617	NA	NA
ILMN_1754103	CLDN11	NA	NA	NA	NA	NA	NA	NA	0,002423151	NA	NA
ILMN_2209766	RHBDD1	NA	NA	NA	NA	NA	NA	NA	0,002471814	NA	NA
ILMN_1658356	PAMR1	0,000872772	NA	NA	NA	NA	NA	NA	0,002476675	NA	NA
ILMN_1702237	FKBP1A	0,004696872	NA	NA	NA	NA	NA	NA	0,002485611	NA	NA
ILMN_1707448	CRKRS	NA	NA	NA	NA	NA	NA	NA	0,002493326	NA	NA
ILMN_1699767	PDE8A	NA	NA	NA	NA	NA	NA	NA	0,002507091	NA	NA
ILMN_2232712	MYO10	0,00933849	NA	NA	NA	NA	NA	NA	0,002515619	NA	NA
ILMN_1722239	TIMM8A	NA	NA	NA	NA	NA	NA	NA	0,002517649	NA	NA
ILMN_2330371	TATDN3	NA	NA	NA	NA	NA	NA	NA	0,002570434	NA	NA
ILMN_2355586	ATRN	NA	NA	NA	NA	NA	NA	NA	0,002571398	NA	NA
ILMN_2413251	EWSR1	NA	NA	NA	NA	NA	NA	NA	0,002586846	NA	NA
ILMN_2242937	ARSB	NA	NA	NA	NA	NA	NA	NA	0,002599014	NA	NA
ILMN_1692896	JMJD4	NA	NA	NA	NA	NA	NA	NA	0,002604313	NA	NA
ILMN_1747577	ALAD	NA	NA	NA	NA	NA	NA	NA	0,002627535	NA	NA
ILMN_1734229	SPPL2A	NA	NA	NA	NA	NA	NA	NA	0,002650113	NA	NA
ILMN_1772690	OGFOD1	NA	NA	NA	NA	NA	NA	NA	0,002660538	NA	NA
ILMN_1782069	TRAK1	0,00495574	NA	NA	NA	NA	NA	NA	0,002666219	NA	NA
ILMN_1698243	C1orf85	NA	NA	NA	NA	NA	NA	NA	0,002712908	NA	NA
ILMN_1778444	FKBP5	NA	NA	NA	NA	NA	NA	NA	0,002733736	NA	NA
ILMN_1779228	CDH2	NA	NA	NA	NA	NA	NA	NA	0,002741875	NA	NA
ILMN_3284119	LOC399804	NA	NA	NA	NA	NA	NA	NA	0,00275029	NA	NA
ILMN_1700001	TCTA	NA	NA	NA	NA	NA	NA	NA	0,002764221	NA	NA
ILMN_2203544	OR2A9P	NA	NA	NA	NA	NA	NA	NA	0,002783834	NA	NA
ILMN_1745904	CCDC6	NA	NA	NA	NA	NA	NA	NA	0,002790902	NA	NA
ILMN_1778957	PNRC2	NA	NA	NA	NA	NA	NA	NA	0,002806582	NA	NA
ILMN_1656287	SPOCK2	NA	0,00806504	NA	NA	NA	NA	NA	0,002813811	NA	NA
ILMN_1656129	SLC39A10	NA	NA	NA	NA	NA	NA	NA	0,00281779	NA	NA
ILMN_1793859	ALDH2	NA	NA	NA	NA	NA	NA	NA	0,002818217	NA	NA
ILMN_1789266	CCDC25	NA	NA	NA	NA	NA	NA	NA	0,002839786	NA	NA
ILMN_1682792	BYSL	NA	NA	NA	NA	NA	NA	NA	0,002859101	NA	NA
ILMN_3251312	TMEM69	NA	NA	NA	NA	NA	NA	NA	0,002880098	NA	NA
ILMN_1801658	BMPR1B	NA	NA	NA	NA	NA	NA	NA	0,002888055	NA	NA
ILMN_2337740	TULP4	NA	NA	NA	NA	NA	NA	NA	0,002899422	NA	NA
ILMN_1651429	SELM	NA	NA	NA	NA	NA	NA	NA	0,00290715	NA	NA
ILMN_1687546	HSP90AA1	NA	NA	NA	NA	NA	NA	NA	0,002918306	NA	NA
ILMN_2133675	SGSH	NA	NA	NA	NA	NA	NA	NA	0,002953474	NA	NA
ILMN_1815682	C3orf37	NA	NA	NA	NA	NA	NA	NA	0,002975232	NA	NA
ILMN_1652409	SPATA7	NA	NA	NA	NA	NA	NA	NA	0,002987114	NA	NA
ILMN_2178618	WDR44	NA	NA	NA	NA	NA	NA	NA	0,002994663	NA	NA
ILMN_1778321	SLC2A6	NA	NA	NA	NA	NA	NA	NA	0,00299541	NA	NA
ILMN_1708672	ACAT2	NA	NA	NA	NA	NA	NA	NA	0,003000615	NA	NA
ILMN_1659524	C6orf66	NA	NA	NA	NA	NA	NA	NA	0,003008778	NA	NA
ILMN_1781386	WIPI1	NA	NA	NA	NA	NA	NA	NA	0,003023044	NA	NA
ILMN_2043306	EPB41L5	NA	NA	NA	NA	NA	NA	NA	0,003046278	NA	NA
ILMN_1728844	PTPRN2	NA	NA	NA	NA	NA	NA	NA	0,00306371	NA	NA
ILMN_3238319	BEND6	0,005212516	NA	NA	NA	NA	NA	NA	0,00306766	NA	NA
ILMN_1772459	RPS23	NA	NA	NA	NA	NA	NA	NA	0,003070675	NA	NA
ILMN_1746917	LOC729843	0,003300528	NA	NA	NA	NA	NA	NA	0,003096568	NA	NA
ILMN_1773623	HSD17B1	NA	NA	NA	NA	NA	NA	NA	0,003098067	NA	NA
ILMN_1801923	ATF1	NA	NA	NA	NA	NA	NA	NA	0,003105208	NA	NA
ILMN_1675210	ADAM22	NA	0,006169238	NA	NA	NA	NA	NA	0,003116451	NA	NA
ILMN_1794063	ANKRD27	0,005437352	NA	NA	NA	NA	NA	NA	0,003156185	NA	NA
ILMN_1728168	C20orf45	NA	NA	NA	NA	NA	NA	NA	0,003171937	NA	NA
ILMN_1687840	ABCB7	NA	NA	NA	NA	NA	NA	NA	0,00317524	NA	NA
ILMN_1706825	PKN2	NA	NA	NA	NA	NA	NA	NA	0,003203116	NA	NA
ILMN_1766408	CBFB	NA	NA	NA	NA	NA	NA	NA	0,003232443	NA	NA
ILMN_3238785	SNHG9	NA	NA	NA	NA	NA	NA	NA	0,00323301	NA	NA
ILMN_1765578	TIPARP	NA	NA	NA	NA	NA	NA	NA	0,003273152	NA	NA
ILMN_1787897	CXCL1	NA	NA	NA	NA	NA	NA	NA	0,003303548	NA	NA
ILMN_1696767	SERP2	NA	NA	NA	NA	NA	NA	NA	0,003308872	NA	NA
ILMN_1707240	PTBP2	NA	NA	NA	NA	NA	NA	NA	0,003342983	NA	NA
ILMN_2373689	DIAPH3	NA	NA	NA	NA	NA	NA	NA	0,003376698	NA	NA
ILMN_1740407	CHSY3	NA	NA	NA	NA	NA	NA	NA	0,003382349	NA	NA
ILMN_1677953	OGFOD1	NA	NA	NA	NA	NA	NA	NA	0,003384647	NA	NA
ILMN_1797074	EMG1	NA	NA	NA	NA	NA	NA	NA	0,003384945	NA	NA
ILMN_1715188	USP8	NA	NA	NA	NA	NA	NA	NA	0,003417603	NA	NA
ILMN_2328280	ACTL6A	NA	NA	NA	NA	NA	NA	NA	0,003424467	NA	NA
ILMN_1683969	FKBP1A	NA	NA	NA	NA	NA	NA	NA	0,003433194	NA	NA
ILMN_1655469	TSPAN3	NA	NA	NA	NA	NA	NA	NA	0,003433454	NA	NA
ILMN_1664440	TP53BP1	NA	NA	NA	NA	NA	NA	NA	0,003435096	NA	NA
ILMN_1651385	MFN2	NA	NA	NA	NA	NA	NA	NA	0,003438968	NA	NA
ILMN_1810228	TTF2	NA	NA	NA	NA	NA	NA	NA	0,003439651	NA	NA
ILMN_2161577	CXCL6	NA	NA	NA	NA	NA	NA	NA	0,003442466	NA	NA
ILMN_2356111	SLC41A3	NA	NA	NA	NA	NA	NA	NA	0,003457637	NA	NA
ILMN_1756793	POLS	NA	NA	NA	NA	NA	NA	NA	0,003458601	NA	NA
ILMN_1819783		0,003923427	NA	NA	0,00215588	NA	NA	NA	0,003477688	NA	NA

ILMN_1731984	HMGN3	NA	NA	NA	NA	NA	NA	NA	0,003508583	NA	NA
ILMN_3237156	LOC1000096	NA	NA	NA	NA	NA	NA	NA	0,003545202	NA	NA
ILMN_1776157	Sep 04	NA	NA	NA	NA	NA	NA	NA	0,00354768	NA	NA
ILMN_1691053	LOC91561	NA	NA	NA	NA	NA	NA	NA	0,003575512	NA	NA
ILMN_3306019	LOC729535	NA	NA	NA	NA	NA	NA	NA	0,003579891	NA	NA
ILMN_1691097	HSP90AA1	NA	NA	NA	NA	NA	NA	NA	0,00359353	NA	NA
ILMN_1774974	CLUAP1	NA	NA	NA	NA	NA	NA	NA	0,003623497	NA	NA
ILMN_3260017	HNRNPK	NA	NA	NA	NA	NA	NA	NA	0,003659221	NA	NA
ILMN_1739885	SLC41A3	NA	NA	NA	NA	NA	NA	NA	0,003663281	NA	NA
ILMN_3280565	LOC389342	NA	NA	NA	NA	NA	NA	NA	0,003698077	NA	NA
ILMN_2208158	GTF2IP1	NA	NA	NA	NA	NA	NA	NA	0,003698571	NA	NA
ILMN_1662741	EDG4	NA	NA	NA	NA	NA	NA	NA	0,003708834	NA	NA
ILMN_1789505	ITPR1	NA	NA	NA	NA	NA	NA	NA	0,003716145	NA	NA
ILMN_2400292	MAPK9	NA	NA	NA	NA	NA	NA	NA	0,003737391	NA	NA
ILMN_1708328	RAB11FIP3	NA	NA	NA	NA	NA	NA	NA	0,003741431	NA	NA
ILMN_1672596	BCAR1	NA	NA	NA	NA	NA	NA	NA	0,003750344	NA	NA
ILMN_1767459	POLR3B	NA	NA	NA	NA	NA	NA	NA	0,003751049	NA	NA
ILMN_2198812	FDX1	NA	NA	NA	NA	NA	NA	NA	0,003776587	NA	NA
ILMN_1688877	DIRAS3	NA	NA	NA	NA	NA	NA	NA	0,003779026	NA	NA
ILMN_3245869	LOC440957	NA	NA	NA	NA	NA	NA	NA	0,003805014	NA	NA
ILMN_1664369	DHTKD1	NA	NA	NA	NA	NA	NA	NA	0,003806455	NA	NA
ILMN_1808299	IQSEC1	NA	NA	NA	NA	NA	NA	NA	0,003811388	NA	NA
ILMN_1738750	TFCP2	NA	NA	NA	NA	NA	NA	NA	0,003840755	NA	NA
ILMN_2397188	CACNA1H	NA	NA	NA	NA	NA	NA	NA	0,003842316	NA	NA
ILMN_2280731	C17orf95	NA	NA	NA	NA	NA	NA	NA	0,003847391	NA	NA
ILMN_2275533	DIAPH3	NA	NA	NA	0,00640957	NA	NA	NA	0,003874117	NA	NA
ILMN_1684746	IPO11	NA	NA	NA	NA	NA	NA	NA	0,003882953	NA	NA
ILMN_1808501	SH3KBP1	NA	NA	NA	NA	NA	NA	NA	0,003888588	NA	NA
ILMN_1740169	TYRO3	NA	NA	NA	NA	NA	NA	NA	0,003897602	NA	NA
ILMN_1812995	CTSL1	NA	NA	NA	NA	NA	NA	NA	0,003925475	NA	NA
ILMN_1814726	SCARB2	NA	NA	NA	NA	NA	NA	NA	0,003954918	NA	NA
ILMN_1752591	LEPROTL1	NA	NA	NA	NA	NA	NA	NA	0,003962679	NA	NA
ILMN_1780940	PPP2R5D	NA	NA	NA	NA	NA	NA	NA	0,003993765	NA	NA
ILMN_2061732	YRDC	NA	NA	NA	NA	NA	NA	NA	0,003993843	NA	NA
ILMN_1675186	ME2	NA	NA	NA	NA	NA	NA	NA	0,004012249	NA	NA
ILMN_1730416	CYCS	NA	NA	NA	NA	NA	NA	NA	0,00401248	NA	NA
ILMN_1700915	BMI1	NA	NA	NA	NA	NA	NA	NA	0,004026363	NA	NA
ILMN_1802458	AGTRAP	NA	NA	NA	NA	NA	NA	NA	0,004034494	NA	NA
ILMN_1652459	LOC654161	NA	NA	NA	NA	NA	NA	NA	0,004053325	NA	NA
ILMN_2166972	BBS12	NA	NA	NA	NA	NA	NA	NA	0,004067777	NA	NA
ILMN_1666597	PI4KB	NA	NA	NA	NA	NA	NA	NA	0,004075468	NA	NA
ILMN_1792092	ZCCHC8	NA	NA	NA	NA	NA	NA	NA	0,004081777	NA	NA
ILMN_1745772	ASCC3	NA	NA	NA	0,00756764	NA	NA	NA	0,004094044	NA	NA
ILMN_1686664	MT2A	NA	NA	NA	NA	NA	NA	NA	0,004106561	NA	NA
ILMN_1679358	ZDHHC5	NA	NA	NA	NA	NA	NA	NA	0,004135443	NA	NA
ILMN_2058795	PGCP	NA	NA	NA	NA	NA	NA	NA	0,004136007	NA	NA
ILMN_1655117	WDR19	NA	NA	NA	NA	NA	NA	NA	0,004164875	NA	NA
ILMN_1689059	ZNF329	NA	NA	NA	NA	NA	NA	NA	0,004173267	NA	NA
ILMN_2375651	SCNM1	NA	NA	NA	NA	NA	NA	NA	0,00418648	NA	NA
ILMN_2311166	ITGB5	NA	NA	NA	NA	NA	NA	NA	0,004213375	NA	NA
ILMN_1696591	RB1	NA	NA	NA	NA	NA	NA	NA	0,004227809	NA	NA
ILMN_1651767	MKL1	NA	NA	NA	NA	NA	NA	NA	0,004331874	NA	NA
ILMN_1680831	BAZ1B	NA	NA	NA	NA	NA	NA	NA	0,004332176	NA	NA
ILMN_1701551	ABCA6	NA	NA	NA	NA	NA	NA	NA	0,004366373	NA	NA
ILMN_1698323	PLEKHB2	NA	NA	NA	NA	NA	NA	NA	0,004369874	NA	NA
ILMN_1692962	CTDSP2	NA	NA	NA	NA	NA	NA	NA	0,004377288	NA	NA
ILMN_1673843	CST2	NA	NA	NA	NA	NA	NA	NA	0,004395004	NA	NA
ILMN_1701603	ALPL	NA	NA	NA	NA	NA	NA	NA	0,004413365	NA	NA
ILMN_2343563	ANAPC11	NA	NA	NA	NA	NA	NA	NA	0,004417248	NA	NA
ILMN_1703132	LYRM2	NA	NA	NA	NA	NA	NA	NA	0,004433684	NA	NA
ILMN_1808792	ALKBH6	NA	NA	NA	NA	NA	NA	NA	0,00445726	NA	NA
ILMN_1702009	SV2A	NA	NA	NA	NA	NA	NA	NA	0,004482079	NA	NA
ILMN_2204754	TMX4	NA	NA	NA	NA	NA	NA	NA	0,004485345	NA	NA
ILMN_1742260	SSFA2	NA	NA	NA	NA	NA	NA	NA	0,004526308	NA	NA
ILMN_2156267	EIF2AK1	NA	NA	NA	NA	NA	NA	NA	0,004581211	NA	NA
ILMN_3250257	ACVRL1	NA	NA	NA	NA	NA	NA	NA	0,004602245	NA	NA
ILMN_1712389	CKLF	0,007114236	NA	NA	NA	NA	NA	NA	0,004603257	NA	NA
ILMN_1687519	SNAP23	NA	NA	NA	NA	NA	NA	NA	0,004650703	NA	NA
ILMN_1675472	LOC644799	NA	NA	NA	NA	NA	NA	NA	0,004656055	NA	NA
ILMN_1760245	TMEM42	NA	NA	NA	NA	NA	NA	NA	0,00466046	NA	NA
ILMN_1737398	PTPLAD1	NA	NA	NA	NA	NA	NA	NA	0,004667676	NA	NA
ILMN_1761363	VAMP4	NA	NA	NA	NA	NA	NA	NA	0,004690259	NA	NA
ILMN_1765204	ST13	0,009779077	NA	NA	NA	NA	NA	NA	0,004704067	NA	NA
ILMN_2325008	DHX40	NA	NA	NA	NA	NA	NA	NA	0,004706185	NA	NA
ILMN_2388547	EPSTI1	NA	NA	NA	NA	NA	NA	NA	0,004712744	NA	NA
ILMN_1811121	CXorf39	NA	NA	NA	NA	NA	NA	NA	0,004721504	NA	NA
ILMN_2339779	ATP6V1E1	NA	NA	NA	NA	NA	NA	NA	0,004723718	NA	NA
ILMN_1739847	EIF3D	NA	NA	NA	NA	NA	NA	NA	0,004732617	NA	NA
ILMN_2060086	ADAM23	NA	NA	NA	NA	NA	NA	NA	0,004753724	NA	NA
ILMN_2087941	ENTPD3	NA	NA	NA	NA	NA	NA	NA	0,004754914	NA	NA
ILMN_1779177	U2AF1L4	NA	NA	NA	NA	NA	NA	NA	0,004785245	NA	NA
ILMN_1656886	LIN37	NA	NA	NA	NA	NA	NA	NA	0,004809841	NA	NA
ILMN_1774596	BSCL2	NA	NA	NA	NA	NA	NA	NA	0,00481449	NA	NA
ILMN_1670037	POLR2L	NA	NA	NA	NA	NA	NA	NA	0,004818584	NA	NA
ILMN_1718766	MT1F	NA	NA	NA	NA	NA	NA	NA	0,00486598	NA	NA
ILMN_1736939	UGCG	NA	NA	NA	NA	NA	NA	NA	0,004903745	NA	NA
ILMN_1724826	DNM1L	NA	NA	NA	NA	NA	NA	NA	0,004925653	NA	NA
ILMN_3204890	LOC345041	NA	NA	NA	NA	NA	NA	NA	0,004938083	NA	NA
ILMN_1721623	APOO	NA	NA	NA	NA	NA	NA	NA	0,004938551	NA	NA
ILMN_2348090	MRPL55	NA	NA	NA	NA	NA	NA	NA	0,004940316	NA	NA
ILMN_1732575	SEC14L1	NA	NA	NA	NA	NA	NA	NA	0,005042991	NA	NA
ILMN_1772712	UBE2J2	NA	NA	NA	NA	NA	NA	NA	0,005046571	NA	NA
ILMN_1695226	DIAPH3	NA	NA	NA	NA	NA	NA	NA	0,005051057	NA	NA
ILMN_1706598	ACPL2	NA	NA	NA	NA	NA	NA	NA	0,005053423	NA	NA
ILMN_2197846	HADHB	NA	NA	NA	NA	NA	NA	NA	0,005072982	NA	NA
ILMN_1778523	KLF9	0,007847211	NA	NA	0,00086623	NA	NA	NA	0,005098757	NA	NA
ILMN_1762766	PTPRA	NA	NA	NA	NA	NA	NA	NA	0,005112827	NA	NA
ILMN_1804357	GNG4	NA	NA	NA	NA	NA	NA	NA	0,005113358	NA	NA
ILMN_2358626	ADK	NA	NA	NA	NA	NA	NA	NA	0,005127484	NA	NA
ILMN_1757877	HCFC1R1	NA	NA	NA	NA	NA	NA	NA	0,005137155	NA	NA
ILMN_1815668	GTF2IP1	NA	NA	NA	NA	NA	NA	NA	0,005186312	NA	NA
ILMN_1707858	H2AFZ	NA	NA	NA	NA	NA	NA	NA	0,005194354	NA	NA
ILMN_1788053	SLC25A12	NA	NA	NA	NA	NA	NA	NA	0,005199757	NA	NA
ILMN_1688178	RRP7A	NA	NA	NA	NA	NA	NA	NA	0,005227627	NA	NA
ILMN_2411559	PUS1	NA	NA	NA	0,00264398	NA	NA	NA	0,005227929	NA	NA
ILMN_3236160	C4orf49	NA	NA	NA	NA	NA	NA	NA	0,005243409	NA	NA
ILMN_1782633	BOLA2	NA	NA	NA	NA	NA	NA	NA	0,005293493	NA	NA
ILMN_2087989	ZFAND1	NA	NA	NA	NA	NA	NA	NA	0,005304976	NA	NA
ILMN_1808333	PPP1R7	NA	NA	NA	NA	NA	NA	NA	0,005314942	NA	NA

ILMN_1788778	Sep 11	NA	NA	NA	NA	NA	NA	NA	0,005315057	NA	NA
ILMN_1690282	CRADD	NA	NA	NA	NA	NA	NA	NA	0,005341043	NA	NA
ILMN_1786015	CTCF	NA	NA	NA	NA	NA	NA	NA	0,005378481	NA	NA
ILMN_1690392	COMMD3	NA	NA	NA	NA	NA	NA	NA	0,005411272	NA	NA
ILMN_2382906	PLCG1	NA	NA	NA	NA	NA	NA	NA	0,005429567	NA	NA
ILMN_1731353	CHPF	NA	NA	NA	NA	NA	NA	NA	0,005444626	NA	NA
ILMN_1709728	SLC30A5	NA	NA	NA	NA	NA	NA	NA	0,005445569	NA	NA
ILMN_1679391	MAMDC2	NA	NA	NA	NA	NA	NA	NA	0,005514299	NA	NA
ILMN_1713163	SMARCA5	NA	NA	NA	NA	NA	NA	NA	0,005516378	NA	NA
ILMN_1660817	DDB2	NA	NA	NA	NA	NA	NA	NA	0,005537694	NA	NA
ILMN_1738229	NDRG3	NA	NA	NA	NA	NA	NA	NA	0,005539085	NA	NA
ILMN_1779040	INO80B	NA	NA	NA	NA	NA	NA	NA	0,00555362	NA	NA
ILMN_3254481	LOC1001288	NA	NA	NA	NA	NA	NA	NA	0,005556633	NA	NA
ILMN_1813423	NAT15	NA	NA	NA	NA	NA	NA	NA	0,005573032	NA	NA
ILMN_3247645	LOC550643	NA	NA	NA	NA	NA	NA	NA	0,005607017	NA	NA
ILMN_2365595	GMPR2	NA	NA	NA	NA	NA	NA	NA	0,005637455	NA	NA
ILMN_1688725	UTP14A	NA	NA	NA	NA	NA	NA	NA	0,00566833	NA	NA
ILMN_2113728	FLJ31568	NA	0,009495879	NA	NA	NA	NA	NA	0,005687403	NA	NA
ILMN_1698209	AGPS	NA	NA	NA	NA	NA	NA	NA	0,005723378	NA	NA
ILMN_1781280	SRD5A2L2	NA	NA	NA	NA	NA	NA	NA	0,005801132	NA	NA
ILMN_1781700	IL18R1	NA	NA	NA	NA	NA	NA	NA	0,005836015	NA	NA
ILMN_3236135	FAM86D	NA	NA	NA	NA	NA	NA	NA	0,005857219	NA	NA
ILMN_2380999	RECQL	NA	NA	NA	NA	NA	NA	NA	0,005875511	NA	NA
ILMN_1758232	GGCX	NA	NA	NA	NA	NA	NA	NA	0,005886542	NA	NA
ILMN_1682738	SMAD3	NA	NA	NA	NA	NA	NA	NA	0,005919511	NA	NA
ILMN_1773764	CECR7	NA	NA	NA	NA	NA	NA	NA	0,005926492	NA	NA
ILMN_2186806	HLA-F	NA	NA	NA	NA	NA	NA	NA	0,005927509	NA	NA
ILMN_1767934	PCSK5	NA	NA	NA	NA	NA	NA	NA	0,005939171	NA	NA
ILMN_1700024	UST	NA	NA	NA	NA	NA	NA	NA	0,005949058	NA	NA
ILMN_2072101	C4orf49	NA	NA	NA	NA	NA	NA	0,005362702	0,005973838	NA	NA
ILMN_1809400	FAM49B	NA	NA	NA	NA	NA	NA	NA	0,005987244	NA	NA
ILMN_1692545	LOC646849	NA	NA	NA	NA	NA	NA	NA	0,00600326	NA	NA
ILMN_1701331	UBE2M	0,000849737	NA	NA	NA	NA	NA	NA	0,006009074	NA	NA
ILMN_1696432	IDH1	NA	NA	NA	NA	NA	NA	NA	0,006012801	NA	NA
ILMN_2388177	SPATA7	NA	NA	NA	NA	NA	NA	NA	0,006022097	NA	NA
ILMN_3238048	LOC730324	NA	NA	NA	NA	NA	NA	NA	0,006044223	NA	NA
ILMN_1748546	Sep 02	NA	NA	NA	NA	NA	NA	NA	0,006072825	NA	NA
ILMN_1671263	CACNA1H	NA	NA	NA	NA	NA	NA	NA	0,006091409	NA	NA
ILMN_3306997	METTL1	NA	NA	NA	NA	NA	NA	NA	0,006108023	NA	NA
ILMN_1726989	C1orf86	NA	NA	NA	NA	NA	NA	NA	0,006108878	NA	NA
ILMN_1762103	WDR48	NA	NA	NA	NA	NA	NA	NA	0,006144179	NA	NA
ILMN_1671207	IARS2	NA	NA	NA	NA	NA	NA	NA	0,006161956	NA	NA
ILMN_3217285	LOC389322	NA	NA	NA	NA	NA	NA	NA	0,006165992	NA	NA
ILMN_1693233	KIAA0513	NA	NA	NA	NA	NA	NA	NA	0,006167413	NA	NA
ILMN_1662954	CCT7	NA	NA	NA	NA	NA	NA	NA	0,006193955	NA	NA
ILMN_2229940	C2orf7	NA	NA	NA	NA	NA	NA	NA	0,006237976	NA	NA
ILMN_2079386	RPL22	NA	NA	NA	NA	NA	NA	NA	0,006238114	NA	NA
ILMN_1702501	RPS6KA2	0,002382897	NA	NA	NA	NA	NA	NA	0,006248118	NA	NA
ILMN_1874530	DIAPH3	NA	NA	NA	NA	NA	NA	NA	0,006256926	NA	NA
ILMN_1722753	GJA5	NA	NA	NA	NA	NA	NA	NA	0,006319227	NA	NA
ILMN_2410038	FAM111A	NA	NA	NA	NA	NA	NA	NA	0,006346343	NA	NA
ILMN_3246900	LOC92249	NA	NA	NA	NA	NA	NA	NA	0,006350916	NA	NA
ILMN_1712975	YIF1A	NA	0,008856108	NA	NA	NA	NA	NA	0,00635857	NA	NA
ILMN_1737462	OXR1	NA	NA	NA	NA	NA	NA	NA	0,006372836	NA	NA
ILMN_1720344	NIPA2	0,000445447	NA	NA	NA	NA	NA	NA	0,006382252	NA	NA
ILMN_1659343	BOLA2	NA	NA	NA	NA	NA	NA	NA	0,006384669	NA	NA
ILMN_2350634	EFEMP1	NA	NA	NA	NA	NA	NA	NA	0,00638644	NA	NA
ILMN_1677534	SCAP	NA	0,009933713	NA	NA	NA	NA	NA	0,00639779	NA	NA
ILMN_1685156	ADCY6	NA	NA	NA	NA	NA	NA	NA	0,006513108	NA	NA
ILMN_1684321	CYB5B	NA	NA	NA	NA	NA	NA	NA	0,006528651	NA	NA
ILMN_1662198	RANGAP1	NA	NA	NA	NA	NA	NA	NA	0,006530364	NA	NA
ILMN_1715273	PICALM	NA	NA	NA	NA	NA	NA	NA	0,006565201	NA	NA
ILMN_2414027	CKLF	NA	NA	NA	NA	NA	NA	NA	0,006569115	NA	NA
ILMN_1757732	OSGIN2	NA	NA	NA	NA	NA	NA	NA	0,006570894	NA	NA
ILMN_1675406	PPAPDC1B	NA	NA	NA	NA	NA	NA	NA	0,006572698	NA	NA
ILMN_1651987	C6orf129	NA	NA	NA	NA	NA	NA	NA	0,006590008	NA	NA
ILMN_1720703	SLC25A3	NA	NA	NA	NA	NA	NA	NA	0,006591528	NA	NA
ILMN_1692267	B4GALT3	NA	NA	NA	NA	NA	NA	NA	0,006613756	NA	NA
ILMN_1688637	TMEM198	NA	NA	NA	NA	NA	NA	NA	0,006636582	NA	NA
ILMN_2327203	POT1	NA	NA	NA	NA	NA	NA	NA	0,00665733	NA	NA
ILMN_1660436	HSPA1B	NA	NA	NA	NA	NA	NA	NA	0,006670594	NA	NA
ILMN_1813836	DARS	NA	NA	NA	NA	NA	NA	NA	0,006689871	NA	NA
ILMN_1658835	CAV2	NA	NA	NA	NA	NA	NA	NA	0,006697624	NA	NA
ILMN_2412172	APOBEC3F	NA	NA	NA	NA	NA	NA	NA	0,006807579	NA	NA
ILMN_1800008	ACAT1	NA	NA	NA	NA	NA	NA	NA	0,006809454	NA	NA
ILMN_1765371	LUC7L	NA	NA	NA	NA	NA	NA	NA	0,006815597	NA	NA
ILMN_1810977	CDV3	NA	NA	NA	NA	NA	NA	NA	0,006821748	NA	NA
ILMN_1764410	C22orf13	NA	NA	NA	NA	NA	NA	NA	0,006867472	NA	NA
ILMN_2312897	DGUOK	NA	NA	NA	NA	NA	NA	NA	0,006874649	NA	NA
ILMN_1724700	RIOK3	NA	NA	NA	NA	NA	NA	NA	0,006937555	NA	NA
ILMN_2358041	NBN	NA	NA	NA	NA	NA	NA	NA	0,006982204	NA	NA
ILMN_1669831	C6orf192	NA	NA	NA	0,00849114	NA	NA	NA	0,006997702	NA	NA
ILMN_2214473	ARHGEF5L	NA	NA	NA	NA	NA	NA	NA	0,007030208	NA	NA
ILMN_1728779	NGLY1	NA	NA	NA	NA	NA	NA	NA	0,007042615	NA	NA
ILMN_1667707	SPCS3	NA	NA	NA	NA	NA	NA	NA	0,007052749	NA	NA
ILMN_1674522	HIGD1A	NA	NA	NA	NA	NA	NA	NA	0,007081951	NA	NA
ILMN_2183409	SCARB1	NA	NA	NA	NA	NA	NA	NA	0,007102555	NA	NA
ILMN_1657550	MVD	NA	NA	NA	NA	NA	NA	NA	0,007144997	NA	NA
ILMN_1657435	MT1M	NA	NA	NA	NA	NA	NA	NA	0,007173081	NA	NA
ILMN_1739751	SLC26A11	NA	NA	NA	NA	NA	NA	NA	0,007216634	NA	NA
ILMN_1698745	CLRN1	NA	NA	NA	NA	NA	NA	NA	0,007270254	NA	NA
ILMN_2048793	CIAO1	NA	NA	NA	NA	NA	NA	NA	0,007276666	NA	NA
ILMN_1654540	SEMA6D	NA	NA	NA	NA	NA	NA	NA	0,007302978	NA	NA
ILMN_1652163	DVL2	NA	NA	NA	NA	NA	NA	NA	0,007313979	NA	NA
ILMN_1696643	TLN1	NA	NA	NA	NA	NA	NA	NA	0,00733002	NA	NA
ILMN_1733690	AKAP7	NA	NA	NA	NA	NA	NA	NA	0,007388963	NA	NA
ILMN_1801504	RUNX1	NA	NA	NA	NA	NA	NA	NA	0,007429013	NA	NA
ILMN_2363489	BRE	NA	NA	NA	NA	NA	NA	0,001292269	0,007429479	NA	NA
ILMN_1680196	LAPTM4B	NA	NA	NA	NA	NA	NA	NA	0,007467368	NA	NA
ILMN_3245983	NEURL1B	0,000949136	NA	NA	NA	NA	NA	NA	0,007519324	NA	NA
ILMN_1777058	HIATL1	NA	NA	NA	NA	NA	NA	NA	0,007535776	NA	NA
ILMN_1785765	TM9SF2	NA	NA	NA	NA	NA	NA	NA	0,007549915	NA	NA
ILMN_1729868	SPRYD4	NA	NA	NA	NA	NA	NA	NA	0,007560759	NA	NA
ILMN_3190972	LOC1001302	NA	NA	NA	NA	NA	NA	NA	0,007625942	NA	NA
ILMN_1721225	C20orf4	NA	NA	NA	NA	NA	NA	NA	0,007697769	NA	NA
ILMN_1769546	RIN2	NA	NA	NA	NA	NA	NA	NA	0,007702935	NA	NA
ILMN_1708416	ARL6IP1	NA	NA	NA	NA	NA	NA	NA	0,007715318	NA	NA
ILMN_1784655	TLCD1	NA	NA	NA	NA	NA	NA	NA	0,007734953	NA	NA
ILMN_1688089	PEBP1	NA	NA	NA	NA	NA	NA	NA	0,007804309	NA	NA

ILMN_3277332	LOC441453	NA	NA	NA	NA	NA	NA	NA	0,007824546	NA	NA
ILMN_2343010	BOLA3	NA	NA	NA	NA	NA	NA	NA	0,007861209	NA	NA
ILMN_1735495	TBC1D8	NA	NA	NA	NA	NA	NA	NA	0,007861467	NA	NA
ILMN_2113362	ARL6IP1	NA	NA	NA	NA	NA	NA	NA	0,007864884	NA	NA
ILMN_1741224	GPR137C	NA	NA	NA	NA	NA	NA	NA	0,007874235	NA	NA
ILMN_1795026	FAM189B	NA	NA	NA	NA	NA	NA	NA	0,007879754	NA	NA
ILMN_1778173	AK3	NA	NA	NA	NA	NA	NA	NA	0,007881993	NA	NA
ILMN_1778557	CDC2L5	NA	NA	NA	NA	NA	NA	NA	0,007914326	NA	NA
ILMN_1758717	SUPT6H	NA	NA	NA	NA	NA	NA	NA	0,007922731	NA	NA
ILMN_3268914	LOC1001284	NA	NA	NA	NA	NA	NA	NA	0,007945591	NA	NA
ILMN_1728965	C11orf57	NA	NA	NA	NA	NA	NA	NA	0,007968666	NA	NA
ILMN_1788239	AMDHD1	NA	NA	NA	NA	NA	NA	NA	0,008024738	NA	NA
ILMN_3180989	DSTYK	NA	NA	NA	NA	NA	NA	NA	0,008083164	NA	NA
ILMN_1763524	LZTS2	NA	NA	NA	NA	NA	NA	NA	0,008127531	NA	NA
ILMN_1738346	EFHA1	NA	0,009618685	NA	NA	NA	NA	0,0071198	0,008178075	NA	NA
ILMN_1659894	SLC25A26	0,007265487	NA	NA	NA	NA	NA	NA	0,008197637	NA	NA
ILMN_2230892	IL10RB	NA	NA	NA	NA	NA	NA	NA	0,008208935	NA	NA
ILMN_3217276	LOC644517	NA	NA	NA	NA	NA	NA	NA	0,008264166	NA	NA
ILMN_1752923	IFNAR1	NA	NA	NA	NA	NA	NA	NA	0,008271138	NA	NA
ILMN_3239263	RPL23AP53	NA	NA	NA	NA	NA	NA	0,008923812	0,00830033	NA	NA
ILMN_1796589	TRIP13	NA	NA	NA	NA	NA	NA	NA	0,008302675	NA	NA
ILMN_1666376	TRIM56	NA	NA	NA	NA	NA	NA	0,007422085	0,008304226	NA	NA
ILMN_1727332	ATPIF1	NA	NA	NA	NA	NA	NA	NA	0,008312076	NA	NA
ILMN_2095133	SPTAN1	NA	NA	NA	NA	NA	NA	NA	0,008326352	NA	NA
ILMN_1687821	C16orf45	NA	NA	NA	NA	NA	NA	NA	0,008359343	NA	NA
ILMN_2096083	PLEKHA5	NA	NA	NA	NA	NA	NA	NA	0,008365923	NA	NA
ILMN_2356786	ADD1	NA	NA	NA	NA	NA	NA	NA	0,008392846	NA	NA
ILMN_1689296	ARMC10	NA	NA	NA	NA	NA	NA	NA	0,00843104	NA	NA
ILMN_1753584	KRT8	NA	NA	NA	NA	NA	NA	NA	0,008434863	NA	NA
ILMN_1733462	HSD3B2	NA	NA	NA	NA	NA	NA	NA	0,008485865	NA	NA
ILMN_2369286	NME7	NA	NA	NA	NA	NA	NA	NA	0,00849071	NA	NA
ILMN_1707484	GEMIN6	NA	NA	NA	NA	NA	NA	NA	0,008520247	NA	NA
ILMN_1758034	ETFDH	NA	NA	NA	NA	NA	NA	NA	0,008522126	NA	NA
ILMN_1704621	WDR44	NA	NA	NA	NA	NA	NA	NA	0,008526082	NA	NA
ILMN_1779234	CXCL6	NA	NA	NA	NA	NA	NA	NA	0,008623236	NA	NA
ILMN_1717925	SIGMAR1	NA	NA	NA	NA	NA	NA	NA	0,008627624	NA	NA
ILMN_2209748	DERL1	NA	NA	NA	NA	NA	NA	NA	0,008667449	NA	NA
ILMN_2165867	DHCR7	NA	NA	NA	NA	NA	NA	NA	0,008670985	NA	NA
ILMN_1774432	DTD1	NA	NA	NA	NA	NA	NA	NA	0,008711324	NA	NA
ILMN_2409451	NCKAP1	NA	NA	NA	NA	NA	NA	NA	0,008803146	NA	NA
ILMN_1737074	LOC440589	NA	NA	NA	NA	NA	NA	NA	0,008841745	NA	NA
ILMN_1742866	F2R	NA	NA	NA	NA	NA	NA	NA	0,008884436	NA	NA
ILMN_1726417	MRPL33	NA	NA	NA	NA	NA	NA	NA	0,008889753	NA	NA
ILMN_2288070	FTO	NA	NA	NA	NA	NA	NA	NA	0,008891632	NA	NA
ILMN_3243297	LOC641814	NA	NA	NA	NA	NA	NA	NA	0,008914845	NA	NA
ILMN_1814606	GALNT2	NA	NA	NA	NA	NA	NA	NA	0,008919932	NA	NA
ILMN_1663032	FNDC4	NA	NA	NA	NA	NA	NA	NA	0,009008647	NA	NA
ILMN_1702899	PLEKHA7	NA	NA	NA	NA	NA	NA	NA	0,009022271	NA	NA
ILMN_1801845	DNAL4	NA	NA	NA	NA	NA	NA	NA	0,00905141	NA	NA
ILMN_3242048	ASAH2C	NA	NA	NA	NA	NA	NA	NA	0,009055375	NA	NA
ILMN_2047206	TMX3	NA	NA	NA	NA	NA	NA	NA	0,009060238	NA	NA
ILMN_3304850	LOC730098	NA	NA	NA	NA	NA	NA	NA	0,009064802	NA	NA
ILMN_1708059	USP13	NA	NA	NA	NA	NA	NA	NA	0,009113022	NA	NA
ILMN_1677768	POR	NA	NA	NA	NA	NA	NA	NA	0,00916436	NA	NA
ILMN_3282506	LOC728098	NA	NA	NA	NA	NA	NA	NA	0,009200319	NA	NA
ILMN_1699852	CES8	NA	NA	NA	NA	NA	NA	NA	0,009209758	NA	NA
ILMN_3224204	PSMG4	NA	NA	NA	NA	NA	NA	NA	0,00926672	NA	NA
ILMN_1779279	C14orf32	NA	NA	NA	NA	NA	NA	NA	0,009293345	NA	NA
ILMN_1758039	KRTAP13-2	NA	NA	NA	NA	NA	NA	NA	0,009332403	NA	NA
ILMN_1656410	TRPC6	NA	NA	NA	NA	NA	NA	NA	0,009376281	NA	NA
ILMN_2412294	GNB5	NA	NA	NA	NA	NA	NA	NA	0,009394195	NA	NA
ILMN_1709043	C9orf46	NA	NA	NA	NA	NA	NA	NA	0,009443417	NA	NA
ILMN_3247578	FAT1	NA	NA	NA	NA	NA	NA	NA	0,009448011	NA	NA
ILMN_1661424	THAP6	NA	NA	NA	NA	NA	NA	NA	0,009458153	NA	NA
ILMN_1810433	LOC652545	NA	NA	NA	NA	NA	NA	NA	0,009458773	NA	NA
ILMN_1693341	SNRPN	NA	NA	NA	NA	NA	NA	NA	0,009485994	NA	NA
ILMN_1694491	CCNG1	NA	NA	NA	NA	NA	NA	NA	0,009509741	NA	NA
ILMN_1713322	C7orf28B	NA	NA	NA	NA	NA	NA	NA	0,009521974	NA	NA
ILMN_1748650	MRPL45	NA	NA	NA	NA	NA	NA	NA	0,009534697	NA	NA
ILMN_1663667	FLAD1	NA	NA	NA	NA	NA	NA	NA	0,009550279	NA	NA
ILMN_2149226	CAV1	NA	NA	NA	NA	NA	NA	NA	0,009573288	NA	NA
ILMN_2184064	ARRDC4	NA	0,006086437	NA	NA	NA	NA	NA	0,009598032	NA	NA
ILMN_1692684	SMARCD2	NA	NA	NA	NA	NA	NA	NA	0,009612922	NA	NA
ILMN_1725862	USP3	NA	NA	NA	NA	NA	NA	NA	0,009623625	NA	NA
ILMN_3242586	RHOU	NA	NA	NA	NA	NA	NA	NA	0,009658084	NA	NA
ILMN_1737426	PCMTD1	NA	NA	NA	NA	NA	NA	NA	0,009664735	NA	NA
ILMN_3192316	LOC1001292	NA	NA	NA	NA	NA	NA	NA	0,009683194	NA	NA
ILMN_1732509	LRRC40	NA	NA	NA	NA	NA	NA	NA	0,009697412	NA	NA
ILMN_2342793	FBXW8	NA	0,006579712	NA	NA	NA	NA	NA	0,009719085	NA	NA
ILMN_1777565	TAP2	NA	NA	NA	NA	NA	NA	NA	0,009741768	NA	NA
ILMN_2117330	NDUFB2	0,009738497	NA	NA	NA	NA	NA	NA	0,009774464	NA	NA
ILMN_1778836	SFRS7	NA	NA	NA	NA	NA	NA	NA	0,009793289	NA	NA
ILMN_1728660	PMPCB	NA	NA	NA	NA	NA	NA	NA	0,009795949	NA	NA
ILMN_1804929	OXTR	0,00451481	NA	NA	NA	NA	NA	NA	0,009796248	NA	NA
ILMN_2372082	SNRPN	NA	NA	NA	NA	NA	NA	NA	0,00980369	NA	NA
ILMN_1757497	VGF	NA	NA	NA	NA	NA	NA	NA	0,009823503	NA	NA
ILMN_2189936	RPL36AL	NA	NA	NA	NA	NA	NA	NA	0,009828714	NA	NA
ILMN_2360730	CAV2	NA	NA	NA	NA	NA	NA	NA	0,009860461	NA	NA
ILMN_1679188	ATP5S	NA	NA	NA	NA	NA	NA	NA	0,009915778	NA	NA
ILMN_1669508	PAF1	NA	NA	NA	NA	NA	NA	NA	0,009961845	NA	NA
ILMN_1786328	WDR40A	NA	NA	NA	NA	NA	NA	NA	0,009974953	NA	NA
ILMN_2389273	FXR1	NA	NA	NA	NA	NA	NA	NA	0,009985972	NA	NA
ILMN_1783156	LOC650832	NA	0,004923092	NA	NA	NA	NA	2,15E-05	NA	NA	NA
ILMN_1805251	LOC641983	NA	NA	NA	NA	NA	NA	2,15E-05	NA	NA	NA
ILMN_2344455	G3BP1	NA	NA	NA	NA	NA	NA	2,67E-05	NA	NA	NA
ILMN_2379560	CDC14B	NA	NA	NA	NA	NA	NA	3,13E-05	NA	NA	NA
ILMN_1745116	ABHD12	NA	NA	NA	NA	NA	NA	3,15E-05	NA	NA	NA
ILMN_1790534	MAP2K3	NA	NA	NA	NA	NA	NA	3,34E-05	NA	NA	NA
ILMN_2201580	GSTM2	NA	0,003472635	NA	NA	NA	NA	3,89E-05	NA	NA	NA
ILMN_1687074	CDC14B	NA	NA	NA	NA	NA	NA	4,10E-05	NA	NA	NA
ILMN_1765851	TRADD	NA	NA	NA	NA	NA	NA	4,28E-05	NA	NA	NA
ILMN_1780132	PELI2	NA	0,002080744	NA	NA	NA	NA	4,43E-05	NA	NA	NA
ILMN_1691111	SPATA2L	NA	0,008846755	NA	NA	NA	NA	4,50E-05	NA	NA	NA
ILMN_1707748	PIM3	NA	NA	NA	NA	NA	NA	4,69E-05	NA	NA	NA
ILMN_1804822	SRXN1	NA	NA	NA	NA	NA	NA	4,80E-05	NA	NA	NA
ILMN_1786039	RNF34	NA	NA	NA	NA	NA	NA	5,59E-05	NA	NA	NA
ILMN_1916256		NA	0,008650896	NA	NA	NA	NA	5,61E-05	NA	NA	NA
ILMN_1735353	IRX1	NA	NA	NA	NA	NA	NA	5,72E-05	NA	NA	NA
ILMN_1672591	SCN9A	NA	NA	NA	NA	NA	NA	6,33E-05	NA	NA	NA

ILMN_1777061	ZSWIM6	NA	0,004032479	NA	NA	NA	NA	6,68E-05	NA	NA	NA
ILMN_1683149	LOC388882	NA	NA	NA	NA	NA	NA	8,90E-05	NA	NA	NA
ILMN_1740035	POU4F2	NA	NA	NA	NA	NA	NA	0,000101828	NA	NA	NA
ILMN_2391861	GSTM1	NA	0,008196933	NA	NA	NA	NA	0,000102886	NA	NA	NA
ILMN_1815086	NINJ1	NA	NA	NA	NA	NA	NA	0,000104659	NA	NA	NA
ILMN_3305475	LOC729708	NA	0,000318552	NA	NA	NA	NA	0,00010473	NA	NA	NA
ILMN_1752046	SH2B3	NA	NA	NA	NA	NA	NA	0,000106738	NA	NA	NA
ILMN_1735584	ST8SIA5	NA	NA	NA	NA	NA	NA	0,000114945	NA	NA	NA
ILMN_1792910	MNT	NA	NA	NA	NA	NA	NA	0,000180679	NA	NA	NA
ILMN_1808374	SNTB2	NA	NA	NA	NA	NA	NA	0,000194562	NA	NA	NA
ILMN_1679428	CHIC2	NA	0,003495622	NA	NA	NA	NA	0,000206583	NA	NA	NA
ILMN_1703531	S1PR3	NA	NA	NA	NA	NA	NA	0,000209417	NA	NA	NA
ILMN_1651617	CTNNB1	NA	NA	NA	NA	NA	NA	0,000225502	NA	NA	NA
ILMN_1807873	SNX6	NA	0,008115886	NA	NA	NA	NA	0,000228726	NA	NA	NA
ILMN_3240586	PLD6	NA	NA	NA	NA	NA	NA	0,000229726	NA	NA	NA
ILMN_1661432	NUP43	NA	NA	0,001896182	NA	NA	NA	0,000238244	NA	NA	NA
ILMN_1773643	SLC38A9	NA	0,009724856	NA	NA	NA	NA	0,000252054	NA	NA	NA
ILMN_2321485	PPP1R8	NA	NA	NA	NA	NA	NA	0,000259312	NA	NA	NA
ILMN_1760303	PIK3R1	NA	NA	NA	NA	NA	NA	0,000294914	NA	NA	NA
ILMN_1811181	FLJ20444	NA	NA	NA	NA	NA	NA	0,000316315	NA	NA	NA
ILMN_2282077	MIB2	NA	NA	NA	NA	NA	NA	0,000318056	NA	NA	NA
ILMN_1681160	CCT6P1	NA	NA	NA	NA	NA	NA	0,000320415	NA	NA	NA
ILMN_2308903	WFDC3	NA	NA	NA	NA	NA	NA	0,000323648	NA	NA	NA
ILMN_2406586	CC2D2A	NA	NA	NA	NA	NA	NA	0,000339483	NA	NA	NA
ILMN_1744381	SERPINE1	NA	NA	NA	NA	NA	NA	0,000341117	NA	NA	NA
ILMN_3243606	C14orf174	NA	NA	NA	NA	NA	NA	0,000354707	NA	NA	NA
ILMN_2311537	HMGA1	NA	NA	NA	NA	NA	NA	0,000355213	NA	NA	NA
ILMN_1771841	FOSL1	NA	0,000839412	NA	NA	NA	NA	0,000361202	NA	NA	NA
ILMN_1653794	C6orf160	NA	NA	NA	NA	NA	NA	0,000376392	NA	NA	NA
ILMN_3204908	LOC1001314	NA	NA	NA	NA	NA	NA	0,000398852	NA	NA	NA
ILMN_2095506	SPOPL	NA	NA	NA	NA	NA	NA	0,000440941	NA	NA	NA
ILMN_1670875	PPM1D	NA	NA	NA	NA	NA	NA	0,000444766	NA	NA	NA
ILMN_1662358	MX1	NA	NA	NA	NA	NA	NA	0,000460884	NA	NA	NA
ILMN_1757388	OCEL1	NA	NA	NA	NA	NA	NA	0,00047152	NA	NA	NA
ILMN_3204210	LOC284393	NA	NA	NA	NA	NA	NA	0,000473792	NA	NA	NA
ILMN_1743078	LOC643031	NA	NA	NA	NA	NA	NA	0,00050382	NA	NA	NA
ILMN_1756784	FREQ	NA	NA	NA	NA	NA	NA	0,000516027	NA	NA	NA
ILMN_3248781	SDHAP2	NA	NA	NA	NA	NA	NA	0,000516504	NA	NA	NA
ILMN_1671478	CKB	NA	NA	NA	NA	NA	NA	0,000521544	NA	NA	NA
ILMN_1687247	SPATA20	NA	NA	NA	NA	NA	NA	0,000521921	NA	NA	NA
ILMN_1730101	GSPT2	NA	NA	NA	NA	NA	NA	0,000522697	NA	NA	NA
ILMN_1681456	FOXL2	NA	NA	NA	NA	NA	NA	0,00053388	NA	NA	NA
ILMN_1762134	ECHDC1	NA	NA	NA	NA	NA	NA	0,000585298	NA	NA	NA
ILMN_2073012	TMEM203	NA	NA	NA	NA	NA	NA	0,000614648	NA	NA	NA
ILMN_1675979	RBMS3	NA	0,000868834	NA	NA	NA	NA	0,000629393	NA	NA	NA
ILMN_1740415	WFDC3	NA	NA	NA	NA	NA	NA	0,000645245	NA	NA	NA
ILMN_1665033	NPR3	NA	NA	NA	NA	NA	NA	0,000650173	NA	NA	NA
ILMN_2314169	PTHLH	NA	NA	0,006491719	NA	NA	NA	0,000650182	NA	NA	NA
ILMN_1679401	TRPM4	NA	NA	NA	NA	NA	NA	0,00068798	NA	NA	NA
ILMN_2096191	AASDHPPT	NA	0,007168958	NA	NA	NA	NA	0,000760538	NA	NA	NA
ILMN_2195821	C5orf41	NA	NA	NA	NA	NA	NA	0,000778341	NA	NA	NA
ILMN_1714473	ZNF436	NA	NA	NA	NA	NA	NA	0,000788009	NA	NA	NA
ILMN_1778684	BRE	NA	NA	NA	NA	NA	NA	0,000793367	NA	NA	NA
ILMN_1821517		NA	NA	NA	NA	NA	NA	0,000818605	NA	NA	NA
ILMN_1726368	ZNF135	NA	NA	NA	NA	NA	NA	0,000841057	NA	NA	NA
ILMN_1735788	TRIOBP	NA	NA	NA	NA	NA	NA	0,000854668	NA	NA	NA
ILMN_1685679	RNF146	NA	NA	NA	NA	NA	NA	0,000873748	NA	NA	NA
ILMN_2410929	PAPSS2	NA	NA	NA	NA	NA	NA	0,000875282	NA	NA	NA
ILMN_1730084	COMT	NA	NA	NA	NA	NA	NA	0,000879935	NA	NA	NA
ILMN_2317457	HSD11B1L	NA	NA	NA	NA	NA	NA	0,000915879	NA	NA	NA
ILMN_2131523	SACS	NA	NA	NA	NA	NA	NA	0,000931699	NA	NA	NA
ILMN_1694213	PLEKHO1	NA	NA	NA	NA	NA	NA	0,000945995	NA	NA	NA
ILMN_1769092	FAM176B	NA	NA	NA	NA	NA	NA	0,000951101	NA	NA	NA
ILMN_1757350	CTNNB1	NA	NA	NA	NA	NA	NA	0,000953817	NA	NA	NA
ILMN_1763328	GZF1	NA	NA	NA	NA	NA	NA	0,00095499	NA	NA	NA
ILMN_2118910	TPRKB	NA	NA	0,001598233	NA	NA	NA	0,000980779	NA	NA	NA
ILMN_2151056	C10orf32	NA	NA	NA	NA	NA	NA	0,000996672	NA	NA	NA
ILMN_2183216	TTRAP	NA	NA	NA	NA	NA	NA	0,001000611	NA	NA	NA
ILMN_1681984	GALNT10	NA	NA	NA	NA	NA	NA	0,001073028	NA	NA	NA
ILMN_1796835	RWDD3	NA	NA	NA	NA	NA	NA	0,001084401	NA	NA	NA
ILMN_2344002	SIP1	NA	NA	NA	NA	NA	NA	0,001098991	NA	NA	NA
ILMN_1708345	DNTTIP2	NA	NA	NA	NA	NA	NA	0,001105136	NA	NA	NA
ILMN_1676197	LRP11	NA	NA	NA	NA	NA	NA	0,001109328	NA	NA	NA
ILMN_1785699	PTHLH	NA	NA	NA	NA	NA	NA	0,001115518	NA	NA	NA
ILMN_3242603	GTF2H2B	NA	NA	NA	NA	NA	NA	0,001140279	NA	NA	NA
ILMN_1701308	COL1A1	NA	NA	NA	NA	NA	NA	0,001158473	NA	NA	NA
ILMN_3235176	C2orf89	NA	NA	NA	NA	NA	NA	0,001199215	NA	NA	NA
ILMN_1795251	SPARCL1	NA	NA	NA	NA	NA	NA	0,001211228	NA	NA	NA
ILMN_1672884	MTFMT	NA	NA	NA	NA	NA	NA	0,001256209	NA	NA	NA
ILMN_1779034	NADSYN1	NA	NA	NA	NA	NA	NA	0,001301337	NA	NA	NA
ILMN_1801378	COQ3	NA	NA	NA	NA	NA	NA	0,001304448	NA	NA	NA
ILMN_1761560	PHF13	NA	NA	NA	NA	NA	NA	0,001308236	NA	NA	NA
ILMN_3247783	MGC21881	NA	NA	NA	NA	NA	NA	0,001333705	NA	NA	NA
ILMN_2097185	PUS3	NA	NA	NA	NA	NA	NA	0,001347469	NA	NA	NA
ILMN_3285616	LOC1001334	NA	NA	NA	NA	NA	NA	0,001367619	NA	NA	NA
ILMN_2356991	CD47	NA	0,003281415	NA	NA	NA	NA	0,001389994	NA	NA	NA
ILMN_1672554	C17orf81	NA	0,004736101	NA	NA	NA	NA	0,001394875	NA	NA	NA
ILMN_1692785	KLHL21	NA	NA	NA	NA	NA	NA	0,001400421	NA	NA	NA
ILMN_1770293	KLF5	NA	NA	NA	NA	NA	NA	0,001410088	NA	NA	NA
ILMN_1729234	TPP1	NA	NA	NA	NA	NA	NA	0,00141694	NA	NA	NA
ILMN_1807136	LOC729559	NA	NA	0,005086429	NA	NA	NA	0,001444999	NA	NA	NA
ILMN_1689378	CCRN4L	NA	NA	NA	NA	NA	NA	0,001467548	NA	NA	NA
ILMN_2075927	STK40	NA	NA	NA	NA	NA	NA	0,001510943	NA	NA	NA
ILMN_1773080	OAZ1	NA	NA	NA	NA	NA	NA	0,001520817	NA	NA	NA
ILMN_1709809	NHP2L1	NA	NA	NA	NA	NA	NA	0,00154719	NA	NA	NA
ILMN_1747460	TMEM184B	NA	NA	NA	NA	NA	NA	0,001568635	NA	NA	NA
ILMN_1830462	XYLT1	NA	NA	0,009693748	NA	NA	NA	0,001580067	NA	NA	NA
ILMN_1666713	LYPLA1	NA	NA	NA	NA	NA	NA	0,001636946	NA	NA	NA
ILMN_2169692	SCN9A	NA	NA	NA	NA	NA	NA	0,001638764	NA	NA	NA
ILMN_1803779	AP2A2	NA	NA	NA	NA	NA	NA	0,0016477	NA	NA	NA
ILMN_2176955	CUL5	NA	NA	NA	NA	NA	NA	0,001680881	NA	NA	NA
ILMN_3249331	LOC1001334	NA	NA	NA	NA	NA	NA	0,001701295	NA	NA	NA
ILMN_2338921	C4orf41	NA	NA	NA	NA	NA	NA	0,001705316	NA	NA	NA
ILMN_1685714	INHBB	NA	NA	NA	NA	NA	NA	0,001754635	NA	NA	NA
ILMN_1680738	C5orf13	NA	NA	NA	NA	NA	NA	0,001766506	NA	NA	NA
ILMN_1655921	GTF2E1	NA	NA	NA	NA	NA	NA	0,001779138	NA	NA	NA
ILMN_3224758	LOC92755	NA	NA	NA	NA	NA	NA	0,001786665	NA	NA	NA
ILMN_2218208	SPARCL1	NA	NA	NA	NA	NA	NA	0,001794783	NA	NA	NA
ILMN_1722066	ARMC1	NA	NA	NA	NA	NA	NA	0,001808045	NA	NA	NA

ILMN_2093500	ZBED5	NA	NA	NA	NA	NA	NA	0,001857661	NA	NA	NA
ILMN_2332558	ARL5A	NA	NA	NA	NA	NA	NA	0,00186381	NA	NA	NA
ILMN_2169089	C18orf54	NA	NA	0,000130953	NA	NA	NA	0,001876329	NA	NA	NA
ILMN_2362293	FBXO38	NA	NA	NA	NA	NA	NA	0,001893988	NA	NA	NA
ILMN_1783627	CAST	NA	0,00012194	NA	NA	NA	NA	0,001922693	NA	NA	NA
ILMN_1665736	LOC648024	NA	NA	NA	NA	NA	NA	0,001928126	NA	NA	NA
ILMN_1665107	ITGB1BP1	NA	0,000432228	NA	NA	NA	NA	0,001952233	NA	NA	NA
ILMN_1718069	MIS12	NA	0,004841596	NA	NA	NA	NA	0,0019769	NA	NA	NA
ILMN_1672389	CRYZ	NA	NA	NA	NA	NA	NA	0,001983107	NA	NA	NA
ILMN_1724040	ANKRD57	NA	0,004660763	NA	NA	NA	NA	0,002019305	NA	NA	NA
ILMN_1667748	ANKRD33	NA	NA	NA	NA	NA	NA	0,002025896	NA	NA	NA
ILMN_1786197	NR2F1	NA	NA	NA	NA	NA	NA	0,002053054	NA	NA	NA
ILMN_1701512	KIAA0391	NA	NA	NA	NA	NA	NA	0,002055281	NA	NA	NA
ILMN_2101928	HNRPH1	NA	NA	NA	NA	NA	NA	0,002096561	NA	NA	NA
ILMN_1741727	QPCT	NA	NA	NA	NA	NA	NA	0,002099191	NA	NA	NA
ILMN_1827211		NA	0,008195344	NA	NA	NA	NA	0,002100723	NA	NA	NA
ILMN_1705093	ORC5L	NA	NA	NA	NA	NA	NA	0,002102202	NA	NA	NA
ILMN_1779213	TFG	NA	NA	NA	NA	NA	NA	0,002102985	NA	NA	NA
ILMN_2167011	ECHDC1	NA	NA	NA	NA	NA	NA	0,002113047	NA	NA	NA
ILMN_1739032	TMEM70	NA	NA	NA	NA	NA	NA	0,002117766	NA	NA	NA
ILMN_1668134	GSTM1	NA	NA	NA	NA	NA	NA	0,002128648	NA	NA	NA
ILMN_1777066	NIF3L1	NA	NA	NA	NA	NA	NA	0,002133151	NA	NA	NA
ILMN_1660270	C7orf28A	NA	NA	NA	NA	NA	NA	0,002167608	NA	NA	NA
ILMN_2085862	SLC15A3	NA	NA	NA	NA	NA	NA	0,002175653	NA	NA	NA
ILMN_1758658	FADD	NA	NA	NA	NA	NA	NA	0,002192141	NA	NA	NA
ILMN_3308335	RNU6-1	NA	NA	NA	NA	NA	NA	0,002192388	NA	NA	NA
ILMN_1705774	TIGD5	NA	NA	NA	NA	NA	NA	0,002208221	NA	NA	NA
ILMN_1803686	ADA	NA	NA	NA	NA	NA	NA	0,002220988	NA	NA	NA
ILMN_1688515	ZNF195	NA	NA	NA	NA	NA	NA	0,002225436	NA	NA	NA
ILMN_1700923	ATG4C	NA	NA	NA	NA	NA	NA	0,00222589	NA	NA	NA
ILMN_1725791	PTPLA	NA	NA	NA	NA	NA	NA	0,00223123	NA	NA	NA
ILMN_2370464	ATRIP	NA	NA	NA	NA	NA	NA	0,002250329	NA	NA	NA
ILMN_1702447	IGF2BP2	NA	NA	NA	NA	NA	NA	0,00225564	NA	NA	NA
ILMN_1734880	LOC644128	NA	NA	NA	NA	NA	NA	0,002256594	NA	NA	NA
ILMN_1755411	RBMS2	NA	NA	NA	NA	NA	NA	0,002268815	NA	NA	NA
ILMN_1803510	LOC650803	NA	NA	NA	NA	NA	NA	0,002275655	NA	NA	NA
ILMN_1725387	TMEM200A	NA	NA	NA	NA	NA	NA	0,002280958	NA	NA	NA
ILMN_2357134	SPHK1	NA	NA	NA	NA	NA	NA	0,002302772	NA	NA	NA
ILMN_2411731	HPS5	NA	NA	NA	NA	NA	NA	0,002316057	NA	NA	NA
ILMN_1783170	ING3	NA	NA	NA	NA	NA	NA	0,002334057	NA	NA	NA
ILMN_1703803	ADAMTS3	NA	NA	NA	NA	NA	NA	0,00243727	NA	NA	NA
ILMN_1775692	EIF4G3	NA	NA	0,007331719	NA	NA	NA	0,00244953	NA	NA	NA
ILMN_1719449	DCLK2	NA	0,006395504	NA	NA	NA	NA	0,002469618	NA	NA	NA
ILMN_2154052	FVT1	NA	NA	0,009621997	NA	NA	NA	0,002491222	NA	NA	NA
ILMN_1701854	GNG5	NA	NA	NA	NA	NA	NA	0,002500381	NA	NA	NA
ILMN_2357382	TERF1	NA	NA	NA	NA	NA	NA	0,002522861	NA	NA	NA
ILMN_1813834	PRMT6	NA	NA	NA	NA	NA	NA	0,002530759	NA	NA	NA
ILMN_1797875	ALOX5AP	NA	NA	0,002011559	NA	NA	NA	0,002572464	NA	NA	NA
ILMN_2318685	ABHD12	NA	NA	NA	NA	NA	NA	0,00260726	NA	NA	NA
ILMN_1769556	C5orf23	NA	NA	NA	NA	NA	NA	0,002607493	NA	NA	NA
ILMN_2162989	TMEM189	NA	NA	NA	NA	NA	NA	0,00264167	NA	NA	NA
ILMN_1755926	DBI	NA	0,005925108	NA	NA	NA	NA	0,002666565	NA	NA	NA
ILMN_1751378	RARS	NA	NA	NA	NA	NA	NA	0,002694238	NA	NA	NA
ILMN_2397028	SERPINB8	NA	NA	NA	NA	NA	NA	0,002761031	NA	NA	NA
ILMN_2352921	BPGM	NA	NA	NA	NA	NA	NA	0,00276694	NA	NA	NA
ILMN_1721651	LOC646463	NA	0,007233013	NA	NA	NA	NA	0,002769569	NA	NA	NA
ILMN_1721922	NAB2	NA	NA	NA	NA	NA	NA	0,002810694	NA	NA	NA
ILMN_1732967	KIAA1949	NA	NA	NA	NA	NA	NA	0,002813164	NA	NA	NA
ILMN_1796925	CXADR	NA	NA	NA	NA	NA	NA	0,002820449	NA	NA	NA
ILMN_1725787	RFX1	NA	NA	NA	NA	NA	NA	0,002823049	NA	NA	NA
ILMN_2415235	CSNK1E	NA	NA	NA	NA	NA	NA	0,002836388	NA	NA	NA
ILMN_1768282	SNX21	NA	NA	NA	NA	NA	NA	0,00284528	NA	NA	NA
ILMN_1664424	ZBED5	NA	NA	NA	NA	NA	NA	0,002874418	NA	NA	NA
ILMN_2140510	KLHL13	NA	NA	0,004685065	NA	NA	NA	0,002888459	NA	NA	NA
ILMN_1689004	TNFRSF12A	NA	NA	NA	NA	NA	NA	0,003020571	NA	NA	NA
ILMN_1651347	SERTAD2	NA	NA	NA	NA	NA	NA	0,003054779	NA	NA	NA
ILMN_1768595	DLG4	NA	NA	NA	NA	NA	NA	0,003059599	NA	NA	NA
ILMN_1768640	CRNKL1	NA	NA	NA	NA	NA	NA	0,003091432	NA	NA	NA
ILMN_1671427	FBXW4	NA	0,000216099	NA	NA	NA	NA	0,003108444	NA	NA	NA
ILMN_2405129	ARHGEF1	NA	NA	NA	NA	NA	NA	0,003141318	NA	NA	NA
ILMN_1699545	PCSK7	NA	NA	NA	NA	NA	NA	0,003172517	NA	NA	NA
ILMN_1775937	DDB1	NA	NA	NA	NA	NA	NA	0,003173968	NA	NA	NA
ILMN_1739987	KCNH2	NA	NA	NA	NA	NA	NA	0,003216153	NA	NA	NA
ILMN_1763638	BCAR3	NA	NA	NA	NA	NA	NA	0,003235004	NA	NA	NA
ILMN_2160210	TACSTD1	NA	NA	NA	NA	NA	NA	0,003235697	NA	NA	NA
ILMN_1808356	FAM3A	NA	NA	NA	NA	NA	NA	0,003258417	NA	NA	NA
ILMN_1718712	C20orf177	NA	NA	NA	NA	NA	NA	0,003273094	NA	NA	NA
ILMN_2344007	SIP1	NA	NA	NA	NA	NA	NA	0,003279515	NA	NA	NA
ILMN_1714756	YIPF5	NA	0,004814888	NA	NA	NA	NA	0,003300525	NA	NA	NA
ILMN_1791232	SPRED2	NA	NA	NA	NA	NA	NA	0,003309428	NA	NA	NA
ILMN_1733956	IARS	NA	NA	0,005478514	NA	NA	NA	0,00331844	NA	NA	NA
ILMN_1750876	SLC4A1AP	NA	NA	NA	NA	NA	NA	0,00332974	NA	NA	NA
ILMN_1795474	LOC644131	NA	NA	NA	NA	NA	NA	0,003346689	NA	NA	NA
ILMN_1756874	FBXO42	NA	NA	NA	NA	NA	NA	0,003368561	NA	NA	NA
ILMN_1769390	ABHD15	NA	NA	NA	NA	NA	NA	0,003368625	NA	NA	NA
ILMN_2053178	ACTG1	NA	NA	NA	NA	NA	NA	0,003375064	NA	NA	NA
ILMN_1689774	MRFAP1L1	NA	NA	NA	NA	NA	NA	0,003417241	NA	NA	NA
ILMN_1702763	ZMYM1	NA	NA	NA	NA	NA	NA	0,003483142	NA	NA	NA
ILMN_1746561	BCL2L2	NA	NA	NA	NA	NA	NA	0,003540415	NA	NA	NA
ILMN_2415536	SIPA1	NA	0,002694801	NA	NA	NA	NA	0,003558832	NA	NA	NA
ILMN_1728228	ZNF548	NA	NA	NA	NA	NA	NA	0,003583387	NA	NA	NA
ILMN_1707649	MPDZ	NA	NA	NA	NA	NA	NA	0,003596713	NA	NA	NA
ILMN_1801869	WDR75	NA	NA	NA	NA	NA	NA	0,003598788	NA	NA	NA
ILMN_1764754	RAMP1	NA	NA	NA	NA	NA	NA	0,003661524	NA	NA	NA
ILMN_2268026	C15orf44	NA	NA	NA	NA	NA	NA	0,003682413	NA	NA	NA
ILMN_1800935	ARV1	NA	NA	NA	NA	NA	NA	0,003704956	NA	NA	NA
ILMN_3270641	HNRNPH3	NA	NA	NA	NA	NA	NA	0,003736812	NA	NA	NA
ILMN_2177090	LOC200030	NA	NA	NA	NA	NA	NA	0,003810288	NA	NA	NA
ILMN_1811574	MAPK8IP3	NA	NA	NA	NA	NA	NA	0,003832302	NA	NA	NA
ILMN_1721575	VPS18	NA	NA	NA	NA	NA	NA	0,003836655	NA	NA	NA
ILMN_2086222	BRWD2	NA	0,000309316	NA	NA	NA	NA	0,003837464	NA	NA	NA
ILMN_2198185	CXorf12	NA	NA	NA	NA	NA	NA	0,003858353	NA	NA	NA
ILMN_1701875	ZYX	NA	NA	NA	NA	NA	NA	0,003892698	NA	NA	NA
ILMN_2297765	KCNMA1	NA	NA	NA	NA	NA	NA	0,003914953	NA	NA	NA
ILMN_3206827	LOC1001317	NA	NA	NA	NA	NA	NA	0,003938623	NA	NA	NA
ILMN_1668540	ZNHIT6	NA	0,007283991	NA	NA	NA	NA	0,00394059	NA	NA	NA
ILMN_1680644	MIOS	NA	NA	NA	NA	NA	NA	0,00394235	NA	NA	NA
ILMN_1673069	DPP9	NA	NA	NA	NA	NA	NA	0,00394483	NA	NA	NA
ILMN_2322499	RORA	NA	NA	NA	NA	NA	NA	0,003987077	NA	NA	NA

ILMN_1657950	RPS26P10	NA	NA	NA	NA	NA	NA	0,004003997	NA	NA	NA
ILMN_3238889	RPRD2	NA	NA	0,000999161	NA	0,003460461	NA	0,004036569	NA	NA	NA
ILMN_1806328	PDIK1L	NA	NA	NA	NA	NA	NA	0,004043792	NA	NA	NA
ILMN_1744611	WDSOF1	NA	NA	NA	NA	NA	NA	0,004092563	NA	NA	NA
ILMN_3235065	ZNHIT6	NA	NA	NA	NA	NA	NA	0,004194519	NA	NA	NA
ILMN_2136455	C3orf64	NA	NA	NA	NA	NA	NA	0,004197504	NA	NA	NA
ILMN_2356654	LGALS8	NA	0,009052308	NA	NA	NA	NA	0,004212901	NA	NA	NA
ILMN_1711994	TCIRG1	NA	NA	NA	NA	NA	NA	0,004225817	NA	NA	NA
ILMN_1652907	CDC5L	NA	NA	NA	NA	NA	NA	0,004246333	NA	NA	NA
ILMN_1730670	FSTL3	NA	NA	NA	NA	NA	NA	0,004255528	NA	NA	NA
ILMN_3188180	LOC1001282	NA	NA	NA	NA	NA	NA	0,004274323	NA	NA	NA
ILMN_1720267	TARSL2	NA	0,003719906	NA	NA	NA	NA	0,004293309	NA	NA	NA
ILMN_1685112	TACO1	NA	0,000419939	NA	NA	NA	NA	0,004302632	NA	NA	NA
ILMN_1685567	TGDS	NA	0,001394905	NA	NA	NA	NA	0,004351277	NA	NA	NA
ILMN_1736448	VWC2	NA	NA	NA	NA	NA	NA	0,004365902	NA	NA	NA
ILMN_3225121	LOC728484	NA	NA	NA	NA	NA	NA	0,004384742	NA	NA	NA
ILMN_1680814	EDN2	NA	NA	NA	NA	NA	NA	0,00440139	NA	NA	NA
ILMN_3278754	LOC649445	NA	NA	NA	NA	NA	NA	0,004481061	NA	NA	NA
ILMN_2359287	ITGA6	NA	NA	NA	NA	NA	NA	0,004485696	NA	NA	NA
ILMN_2322498	RORA	NA	NA	NA	NA	NA	NA	0,004489138	NA	NA	NA
ILMN_1653514	RBBP4	NA	NA	NA	NA	NA	NA	0,004510448	NA	NA	NA
ILMN_1653730	OXCT2	NA	NA	NA	NA	NA	NA	0,004513472	NA	NA	NA
ILMN_2105983	XRCC5	NA	NA	NA	NA	NA	NA	0,004551018	NA	NA	NA
ILMN_1654920	HNRPH3	NA	NA	NA	NA	NA	NA	0,004569909	NA	NA	NA
ILMN_1724059	GAS2L1	NA	NA	NA	NA	NA	NA	0,004653746	NA	NA	NA
ILMN_3246097	LOC649839	NA	NA	NA	NA	NA	NA	0,004655704	NA	NA	NA
ILMN_3310351	RNU6-15	NA	NA	NA	NA	NA	NA	0,004711737	NA	NA	NA
ILMN_2115490	NBPF20	NA	NA	NA	NA	NA	NA	0,004745554	NA	NA	NA
ILMN_1741200	RFX5	NA	NA	NA	NA	NA	NA	0,004767977	NA	NA	NA
ILMN_1804396	C14orf4	NA	NA	0,003088849	NA	NA	NA	0,004780838	NA	NA	NA
ILMN_2059211	KIAA0195	NA	NA	NA	NA	NA	NA	0,004782943	NA	NA	NA
ILMN_1722583	COX4NB	NA	0,00197023	NA	NA	NA	NA	0,00486611	NA	NA	NA
ILMN_1725620	GGNBP2	NA	NA	NA	NA	NA	NA	0,004876501	NA	NA	NA
ILMN_3265895	HNRNPR	NA	NA	NA	NA	NA	NA	0,004925142	NA	NA	NA
ILMN_1769757	C14orf104	NA	NA	0,001215674	NA	NA	NA	0,004962129	NA	NA	NA
ILMN_1744912	CTTN	NA	0,00387243	NA	NA	NA	NA	0,004998355	NA	NA	NA
ILMN_1764230	GNPTG	NA	NA	NA	NA	NA	NA	0,004998359	NA	NA	NA
ILMN_2143822	ZNF148	NA	NA	NA	NA	NA	NA	0,005003568	NA	NA	NA
ILMN_1793433	RAB10	NA	NA	NA	NA	NA	NA	0,005021633	NA	NA	NA
ILMN_1739222	ETV5	NA	NA	NA	NA	NA	NA	0,005081864	NA	NA	NA
ILMN_2159859	LYSMD4	NA	NA	NA	NA	NA	NA	0,005118817	NA	NA	NA
ILMN_2141807	C15orf23	NA	NA	0,003241707	NA	NA	NA	0,0052368	NA	NA	NA
ILMN_2135232	SFRS3	NA	NA	NA	NA	NA	NA	0,005243439	NA	NA	NA
ILMN_1693538	STK36	NA	NA	NA	NA	NA	NA	0,005249702	NA	NA	NA
ILMN_1797950	EXTL2	NA	NA	NA	NA	NA	NA	0,00532251	NA	NA	NA
ILMN_1790136	C20orf20	NA	NA	NA	NA	NA	NA	0,005380249	NA	NA	NA
ILMN_1655312	HPS5	NA	NA	NA	NA	NA	NA	0,005382092	NA	NA	NA
ILMN_3199896	LOC442153	NA	NA	NA	NA	NA	NA	0,005399532	NA	NA	NA
ILMN_1836218		NA	NA	NA	NA	NA	NA	0,005446416	NA	NA	NA
ILMN_3185709	IP6K2	NA	NA	NA	NA	NA	NA	0,005528807	NA	NA	NA
ILMN_1764571	ARHGAP23	NA	NA	NA	NA	NA	NA	0,005531069	NA	NA	NA
ILMN_3201216	LOC441550	NA	NA	NA	NA	NA	NA	0,005545512	NA	NA	NA
ILMN_1814009	LOC283157	NA	NA	NA	NA	NA	NA	0,005595407	NA	NA	NA
ILMN_1792986	RFC1	NA	NA	NA	NA	NA	NA	0,005616985	NA	NA	NA
ILMN_1671765	TRIM35	NA	NA	NA	NA	NA	NA	0,005627609	NA	NA	NA
ILMN_1779828	EDEM1	NA	NA	NA	NA	NA	NA	0,005635461	NA	NA	NA
ILMN_2308849	MYADM	NA	NA	NA	NA	NA	NA	0,005639792	NA	NA	NA
ILMN_2316278	MAGED4B	NA	NA	NA	NA	NA	NA	0,005646815	NA	NA	NA
ILMN_1666933	ASH2L	NA	NA	NA	NA	NA	NA	0,005702728	NA	NA	NA
ILMN_1755882	C13orf7	NA	NA	NA	NA	NA	NA	0,005717411	NA	NA	NA
ILMN_1727617	XRN2	NA	NA	NA	NA	NA	NA	0,005718841	NA	NA	NA
ILMN_1651776	FHOD1	NA	NA	0,001248875	NA	NA	NA	0,005728068	NA	NA	NA
ILMN_2335198	NCOA1	NA	NA	NA	NA	NA	NA	0,005728394	NA	NA	NA
ILMN_1716006	C18orf54	NA	NA	8,63E-05	NA	0,008469938	NA	0,005740563	NA	NA	NA
ILMN_1695357	CCDC99	NA	NA	NA	NA	NA	NA	0,005804685	NA	NA	NA
ILMN_1651684	KIAA0586	NA	NA	NA	NA	NA	NA	0,005831367	NA	NA	NA
ILMN_1777318	C9orf64	NA	NA	NA	NA	NA	NA	0,005845199	NA	NA	NA
ILMN_1761486	C13orf34	NA	NA	NA	NA	NA	NA	0,005863665	NA	NA	NA
ILMN_2400326	DYRK3	NA	NA	NA	NA	NA	NA	0,005923587	NA	NA	NA
ILMN_1739792	RHOG	NA	NA	NA	NA	NA	NA	0,005924889	NA	NA	NA
ILMN_1751328	FAM83H	NA	NA	NA	NA	NA	NA	0,005960729	NA	NA	NA
ILMN_1758323	ACPP	NA	NA	NA	NA	NA	NA	0,005980874	NA	NA	NA
ILMN_1729645	BBC3	NA	NA	NA	NA	NA	NA	0,005983532	NA	NA	NA
ILMN_1728724	IL17RA	NA	NA	NA	NA	NA	NA	0,006054693	NA	NA	NA
ILMN_1815673	DKK3	NA	NA	NA	NA	NA	NA	0,006110537	NA	NA	NA
ILMN_1697409	TNFRSF14	NA	NA	NA	NA	NA	NA	0,006130521	NA	NA	NA
ILMN_1732609	KIAA1539	NA	0,001131121	NA	NA	NA	NA	0,006162128	NA	NA	NA
ILMN_1778242	CALM1	NA	NA	0,003769161	NA	NA	NA	0,00616847	NA	NA	NA
ILMN_1654289	ELK1	NA	NA	NA	NA	NA	NA	0,006189734	NA	NA	NA
ILMN_1716488	PACS1	NA	0,001977693	NA	NA	NA	NA	0,006191494	NA	NA	NA
ILMN_2359742	CTSB	NA	NA	NA	NA	NA	NA	0,006228832	NA	NA	NA
ILMN_1717868	CCT8	NA	NA	NA	NA	NA	NA	0,006281989	NA	NA	NA
ILMN_2403006	TJP1	NA	NA	NA	NA	NA	NA	0,006305626	NA	NA	NA
ILMN_1698668	NR1D2	NA	NA	0,004218496	NA	NA	NA	0,006328662	NA	NA	NA
ILMN_2130525	TSPAN13	NA	0,002547361	NA	NA	NA	NA	0,006363877	NA	NA	NA
ILMN_1782688	THNSL1	NA	0,0080258	NA	NA	NA	NA	0,006371547	NA	NA	NA
ILMN_1819854		NA	NA	NA	NA	NA	NA	0,006375085	NA	NA	NA
ILMN_2051867	PTCD2	NA	0,006008304	NA	NA	NA	NA	0,006395677	NA	NA	NA
ILMN_1752111	SMARCAL1	NA	NA	NA	NA	NA	NA	0,006416752	NA	NA	NA
ILMN_1722698	RCHY1	NA	NA	NA	NA	NA	NA	0,006430326	NA	NA	NA
ILMN_1812461	WISP2	NA	NA	NA	NA	NA	NA	0,006437767	NA	NA	NA
ILMN_1664859	ST8SIA1	NA	NA	0,002073738	NA	NA	NA	0,006476085	NA	NA	NA
ILMN_1809139	AHCTF1	NA	NA	NA	NA	NA	NA	0,006486054	NA	NA	NA
ILMN_3200597	LOC441154	NA	NA	NA	NA	NA	NA	0,006507635	NA	NA	NA
ILMN_3283470	LOC644937	NA	NA	NA	NA	NA	NA	0,006558133	NA	NA	NA
ILMN_1731137	TXNDC9	NA	NA	NA	NA	NA	NA	0,00656086	NA	NA	NA
ILMN_1755222	C9orf82	NA	NA	0,001047114	NA	NA	NA	0,006566976	NA	NA	NA
ILMN_1673409	MGC16121	NA	NA	NA	NA	NA	NA	0,006695283	NA	NA	NA
ILMN_1803745	SUOX	NA	NA	NA	NA	NA	NA	0,0067239	NA	NA	NA
ILMN_1655924	TRNT1	NA	NA	NA	NA	NA	NA	0,006766025	NA	NA	NA
ILMN_1656254	NOTCH2NL	NA	NA	NA	NA	NA	NA	0,006773951	NA	NA	NA
ILMN_2307450	ZNF302	NA	0,002594666	NA	NA	NA	NA	0,006774948	NA	NA	NA
ILMN_3301052	LOC728791	NA	NA	0,008821289	NA	NA	NA	0,006794701	NA	NA	NA
ILMN_3249546	RASA4P	NA	NA	NA	NA	NA	NA	0,006803356	NA	NA	NA
ILMN_1686401	LOC728739	NA	NA	NA	NA	NA	NA	0,006812178	NA	NA	NA
ILMN_1781104	MAP2K7	NA	NA	NA	NA	NA	NA	0,006854973	NA	NA	NA
ILMN_2365549	BRPF1	NA	NA	NA	NA	NA	NA	0,006895973	NA	NA	NA
ILMN_1785177	DNAJC14	NA	NA	NA	NA	NA	NA	0,007007811	NA	NA	NA
ILMN_1786766	SNTB2	NA	NA	NA	NA	NA	NA	0,007018639	NA	NA	NA

ILMN_2103720	MRPL15	NA	NA	NA	NA	NA	NA	0,007035983	NA	NA	NA
ILMN_1724139	TMEM123	NA	NA	NA	NA	NA	NA	0,007056433	NA	NA	NA
ILMN_1749838	MZF1	NA	NA	NA	NA	NA	NA	0,007104625	NA	NA	NA
ILMN_1690884	APOA1	NA	NA	NA	NA	NA	NA	0,007133357	NA	NA	NA
ILMN_1793934	PISD	NA	NA	NA	NA	NA	NA	0,007137058	NA	NA	NA
ILMN_1739325	LOC284023	NA	NA	NA	NA	NA	NA	0,007266888	NA	NA	NA
ILMN_1746396	CTNNB1	NA	NA	NA	NA	NA	NA	0,007352734	NA	NA	NA
ILMN_1795911	TTC37	NA	NA	NA	NA	NA	NA	0,007369447	NA	NA	NA
ILMN_1703408	FZD3	NA	NA	NA	NA	NA	NA	0,007393377	NA	NA	NA
ILMN_1780937	MUS81	NA	NA	NA	NA	NA	NA	0,007403728	NA	NA	NA
ILMN_1801491	SLC26A2	NA	NA	NA	NA	NA	NA	0,007413957	NA	NA	NA
ILMN_1800425	SLC9A1	NA	NA	0,001577274	NA	NA	NA	0,007446092	NA	NA	NA
ILMN_1683243	VPS45	NA	NA	NA	NA	NA	NA	0,00744707	NA	NA	NA
ILMN_2237428	SCD5	NA	NA	NA	NA	NA	NA	0,007574282	NA	NA	NA
ILMN_1659777	RUFY1	NA	NA	NA	NA	NA	NA	0,007594315	NA	NA	NA
ILMN_1742167	TUBA1C	NA	NA	NA	NA	NA	NA	0,007601341	NA	NA	NA
ILMN_1769135	DPP7	NA	NA	NA	NA	NA	NA	0,007610528	NA	NA	NA
ILMN_1683328	IP6K2	NA	NA	NA	NA	NA	NA	0,007657611	NA	NA	NA
ILMN_1903568		NA	NA	NA	NA	NA	NA	0,007689446	NA	NA	NA
ILMN_1769734	NT5C3	NA	NA	NA	NA	NA	NA	0,007694258	NA	NA	NA
ILMN_2158781	LOC650717	NA	NA	NA	NA	NA	NA	0,007695525	NA	NA	NA
ILMN_1705907	NUP153	NA	NA	NA	NA	NA	NA	0,007706859	NA	NA	NA
ILMN_2328835	IP6K2	NA	NA	NA	NA	NA	NA	0,007802368	NA	NA	NA
ILMN_1673769	KCNG1	NA	NA	NA	NA	NA	NA	0,007837799	NA	NA	NA
ILMN_1707062	REV1	NA	NA	NA	NA	NA	NA	0,00794429	NA	NA	NA
ILMN_1658498	SLC44A3	NA	NA	NA	NA	NA	NA	0,007945243	NA	NA	NA
ILMN_1703844	DYNC1LI1	NA	NA	NA	NA	NA	NA	0,007945616	NA	NA	NA
ILMN_2311826	USP6NL	NA	NA	0,001290853	NA	NA	NA	0,008068579	NA	NA	NA
ILMN_1694147	PUS3	NA	NA	NA	NA	NA	NA	0,008115689	NA	NA	NA
ILMN_2357781	ZNF436	NA	NA	NA	NA	NA	NA	0,008118035	NA	NA	NA
ILMN_3237368	LOC644132	NA	NA	0,009833143	NA	NA	NA	0,008139391	NA	NA	NA
ILMN_1676213	SRPX2	NA	NA	NA	NA	NA	NA	0,008275446	NA	NA	NA
ILMN_1714700	TRIB2	NA	NA	NA	NA	NA	NA	0,008316452	NA	NA	NA
ILMN_2405023	PPP1CB	NA	NA	NA	NA	NA	NA	0,008374261	NA	NA	NA
ILMN_1723048	GJC2	NA	0,007277544	NA	NA	NA	NA	0,008408054	NA	NA	NA
ILMN_1729368	FZD8	NA	NA	NA	NA	NA	NA	0,008414728	NA	NA	NA
ILMN_1773369	MRPL48	NA	NA	NA	NA	NA	NA	0,008431283	NA	NA	NA
ILMN_2217513	GLB1L	NA	NA	NA	NA	NA	NA	0,008441323	NA	NA	NA
ILMN_2230577	RABGEF1	NA	NA	NA	NA	NA	NA	0,008466878	NA	NA	NA
ILMN_3203515	LOC401717	NA	NA	NA	NA	NA	NA	0,008555031	NA	NA	NA
ILMN_1731113	ZBTB43	NA	NA	NA	NA	NA	NA	0,008640191	NA	NA	NA
ILMN_1771233	CHMP4B	NA	NA	NA	NA	NA	NA	0,008686342	NA	NA	NA
ILMN_1659206	RARA	NA	NA	NA	NA	NA	NA	0,008713832	NA	NA	NA
ILMN_1658494	C13orf15	NA	NA	NA	NA	NA	NA	0,008778887	NA	NA	NA
ILMN_2170595	RRM2B	NA	NA	0,007711891	NA	NA	NA	0,008809823	NA	NA	NA
ILMN_3264543	LOC1001280	NA	NA	NA	NA	NA	NA	0,008841095	NA	NA	NA
ILMN_1714170	SPSB1	NA	NA	NA	NA	NA	NA	0,008854908	NA	NA	NA
ILMN_2350574	MYADM	NA	NA	NA	NA	NA	NA	0,008870754	NA	NA	NA
ILMN_1671260	GPR177	NA	NA	NA	NA	NA	NA	0,008876039	NA	NA	NA
ILMN_1718977	GADD45B	NA	NA	NA	NA	NA	NA	0,008936943	NA	NA	NA
ILMN_1805662	FBXW5	NA	NA	NA	NA	NA	NA	0,008965674	NA	NA	NA
ILMN_1744147	CEBPZ	NA	NA	NA	0,00787527	NA	NA	0,008969848	NA	NA	NA
ILMN_1812355	WDR42C	NA	NA	NA	NA	0,006231069	NA	0,009026772	NA	NA	NA
ILMN_3249032	EPCAM	NA	NA	NA	NA	NA	NA	0,009047746	NA	NA	NA
ILMN_2322375	MAFF	NA	NA	NA	NA	NA	NA	0,009123668	NA	NA	NA
ILMN_3242211	TMEM187	NA	NA	NA	NA	NA	NA	0,009129101	NA	NA	NA
ILMN_2168747	GSTA2	NA	NA	0,009674112	NA	NA	NA	0,009143196	NA	NA	NA
ILMN_2315694	STRADA	NA	NA	NA	NA	NA	NA	0,009211839	NA	NA	NA
ILMN_2243553	ZNF275	NA	NA	NA	NA	NA	NA	0,009232451	NA	NA	NA
ILMN_1655595	SERPINE2	NA	NA	NA	NA	NA	NA	0,009290826	NA	NA	NA
ILMN_2386355	CSNK2A1	NA	NA	NA	NA	NA	NA	0,00930304	NA	NA	NA
ILMN_2334303	SEC24B	NA	NA	NA	NA	NA	NA	0,009374449	NA	NA	NA
ILMN_1680139	MAFF	NA	NA	NA	NA	NA	NA	0,009374752	NA	NA	NA
ILMN_1712556	ZW10	NA	NA	NA	NA	NA	NA	0,009387864	NA	NA	NA
ILMN_1667839	UBR7	NA	NA	NA	NA	NA	NA	0,009400185	NA	NA	NA
ILMN_2305225	NDRG4	NA	NA	NA	NA	NA	NA	0,009461313	NA	NA	NA
ILMN_1741331	MUDENG	NA	NA	NA	NA	NA	NA	0,009477795	NA	NA	NA
ILMN_1707727	ANGPTL4	NA	NA	NA	NA	NA	NA	0,009502936	NA	NA	NA
ILMN_2385178	MIB2	NA	NA	NA	NA	NA	NA	0,009511039	NA	NA	NA
ILMN_2050813	CLGN	NA	NA	NA	NA	NA	NA	0,009545867	NA	NA	NA
ILMN_1769409	C9orf123	NA	NA	NA	NA	NA	NA	0,00958517	NA	NA	NA
ILMN_1660079	RNF44	NA	NA	NA	NA	NA	NA	0,009608336	NA	NA	NA
ILMN_1681591	PTPN1	NA	NA	NA	NA	NA	NA	0,009609379	NA	NA	NA
ILMN_2106931	PPP1R15B	NA	NA	NA	NA	NA	NA	0,009675724	NA	NA	NA
ILMN_1738095	PER2	NA	NA	NA	NA	NA	NA	0,009677117	NA	NA	NA
ILMN_2222768	REPS1	NA	NA	0,007475238	NA	NA	NA	0,00968391	NA	NA	NA
ILMN_1751393	ZNF684	NA	NA	NA	NA	NA	NA	0,009707307	NA	NA	NA
ILMN_3244574	KIAA1430	NA	NA	NA	NA	NA	NA	0,009772966	NA	NA	NA
ILMN_1798354	PAPOLA	NA	NA	NA	NA	NA	NA	0,009826859	NA	NA	NA
ILMN_2383419	GMEB1	NA	NA	NA	NA	NA	NA	0,009898072	NA	NA	NA
ILMN_1753582	RPA2	NA	NA	NA	NA	NA	NA	0,009952652	NA	NA	NA
ILMN_1755120	MAN1A2	NA	NA	NA	NA	NA	0,000723273	NA	NA	NA	NA
ILMN_1698330	NIT1	NA	NA	NA	NA	NA	0,001553421	NA	NA	NA	NA
ILMN_2343105	LIPT1	NA	NA	NA	NA	NA	0,002232785	NA	NA	NA	NA
ILMN_2077623	RRAS2	NA	NA	NA	NA	NA	0,002427912	NA	NA	NA	NA
ILMN_1658717	DEPDC5	0,004682404	NA	NA	NA	NA	0,003313421	NA	NA	NA	NA
ILMN_1711327	TRIM37	NA	NA	NA	NA	NA	0,005577934	NA	NA	NA	NA
ILMN_2326713	CD151	NA	NA	NA	NA	NA	0,005666148	NA	NA	NA	NA
ILMN_1753190	C9orf102	NA	NA	NA	NA	NA	0,00671622	NA	NA	NA	NA
ILMN_1695422	NCL	NA	NA	0,009825709	NA	NA	0,008693858	NA	NA	NA	NA
ILMN_1790282	NUDT19	NA	NA	NA	NA	NA	0,008768117	NA	NA	NA	NA
ILMN_3301478	ZNF716	NA	NA	NA	NA	NA	0,009295247	NA	NA	NA	NA
ILMN_1790978	ATG7	NA	NA	NA	NA	NA	0,009396659	NA	NA	NA	NA
ILMN_1672564	FMO6P	NA	NA	NA	NA	NA	0,009709681	NA	NA	NA	NA
ILMN_1789879	WDR35	NA	NA	NA	NA	NA	0,009842296	NA	NA	NA	NA
ILMN_1903914		NA	NA	0,001597236	NA	0,000309822	NA	NA	NA	NA	NA
ILMN_1803094	PDGFD	NA	NA	0,000165418	NA	0,000357698	NA	NA	NA	NA	NA
ILMN_1750661	FBXW9	NA	NA	NA	NA	0,001183554	NA	NA	NA	NA	NA
ILMN_2352303	RASSF2	NA	NA	0,003667944	NA	0,001200259	NA	NA	NA	NA	NA
ILMN_1805449	TAPBPL	0,000928341	NA	0,000242019	NA	0,001330356	NA	NA	NA	NA	NA
ILMN_1667319	LPPR2	NA	NA	NA	NA	0,001432819	NA	NA	NA	NA	NA
ILMN_1750395	MBD2	NA	NA	0,008711798	NA	0,001551298	NA	NA	NA	NA	NA
ILMN_1715647	VANGL2	NA	NA	NA	NA	0,0016548	NA	NA	NA	NA	NA
ILMN_1798030	XPR1	NA	NA	0,002378208	NA	0,001693964	NA	NA	NA	NA	NA
ILMN_2405684	BIRC3	NA	NA	0,000777549	NA	0,001796471	NA	NA	NA	NA	NA
ILMN_1748755	KCND2	NA	NA	4,71E-05	NA	0,002033137	NA	NA	NA	NA	NA
ILMN_2366388	PRDX1	NA	NA	NA	NA	0,002570044	NA	NA	NA	NA	NA
ILMN_1797025	LOC651380	NA	NA	0,001605359	NA	0,002812278	NA	NA	NA	NA	NA
ILMN_3304111	LOC729978	NA	NA	0,002603582	NA	0,002817654	NA	NA	NA	NA	NA

ILMN_3247325	LOC1001909	NA	NA	NA	NA	0,002847587	NA	NA	NA	NA	NA
ILMN_3245752	LOC1001340	NA	NA	NA	NA	0,003153187	NA	NA	NA	NA	NA
ILMN_2337336	PVRL2	0,000346119	NA	0,000199172	NA	0,003205078	NA	NA	NA	NA	NA
ILMN_1707503	C1orf144	NA	NA	NA	NA	0,003287791	NA	NA	NA	NA	NA
ILMN_1761941	C4orf18	NA	NA	0,000895237	NA	0,003310704	NA	NA	NA	NA	NA
ILMN_1815924	NUP107	NA	NA	NA	NA	0,003463557	NA	NA	NA	NA	NA
ILMN_1715969	SLC25A37	NA	NA	NA	NA	0,00357024	NA	NA	NA	NA	NA
ILMN_1774427	CALCOCO1	NA	NA	0,002890269	NA	0,003682329	NA	NA	NA	NA	NA
ILMN_1808779	CSTF3	NA	NA	0,000166678	NA	0,003960071	NA	NA	NA	NA	NA
ILMN_1800354	CST3	NA	NA	0,005353789	NA	0,003965555	NA	NA	NA	NA	NA
ILMN_1767747	HDAC2	NA	NA	0,001861246	NA	0,004030885	NA	NA	NA	NA	NA
ILMN_1810759	C2orf25	NA	NA	2,39E-05	NA	0,004061403	NA	NA	NA	NA	NA
ILMN_1731043	TRA2A	NA	NA	0,000202574	NA	0,00408841	NA	NA	NA	NA	NA
ILMN_1803939	YIPF6	NA	NA	0,004288431	NA	0,004175447	NA	NA	NA	NA	NA
ILMN_1674985	TMEM51	NA	NA	0,003459566	NA	0,004242855	NA	NA	NA	NA	NA
ILMN_1765558	NPAS2	NA	NA	0,004815778	NA	0,004340055	NA	NA	NA	NA	NA
ILMN_2070052	LOC613037	NA	NA	0,001009453	NA	0,004419763	NA	NA	NA	NA	NA
ILMN_1675085	UBA6	NA	NA	0,00414643	NA	0,004636084	NA	NA	NA	NA	NA
ILMN_1847402		NA	NA	NA	NA	0,004663299	NA	NA	NA	NA	NA
ILMN_1654385	ASB13	NA	NA	0,002169683	NA	0,004691389	NA	NA	NA	NA	NA
ILMN_3268697	LOC1001298	0,004871879	NA	2,32E-05	NA	0,004742885	NA	NA	NA	NA	NA
ILMN_1795937	VIL2	NA	NA	6,23E-05	NA	0,004763345	NA	NA	NA	NA	NA
ILMN_3248026	RBM27	NA	NA	0,0019742	NA	0,004768489	NA	NA	NA	NA	NA
ILMN_2175474	MTRF1L	NA	NA	0,009511022	NA	0,004866887	NA	NA	NA	NA	NA
ILMN_1716730	FAM44B	NA	NA	0,00020371	NA	0,005148212	NA	NA	NA	NA	NA
ILMN_1701052	TUBG2	NA	NA	NA	NA	0,005155261	NA	NA	NA	NA	NA
ILMN_1776653	SCML1	NA	NA	0,003779261	NA	0,005186593	NA	NA	NA	NA	NA
ILMN_1653856	STS-1	NA	NA	NA	NA	0,005449757	NA	NA	NA	NA	NA
ILMN_3239225	RNY3	NA	NA	0,004865085	NA	0,005696579	NA	NA	NA	NA	NA
ILMN_3214893	LOC1001327	NA	NA	0,002321793	NA	0,00587386	NA	NA	NA	NA	NA
ILMN_1730054	GSTT1	NA	NA	0,000189291	NA	0,005986335	NA	NA	NA	NA	NA
ILMN_1670305	SERPING1	NA	NA	0,001350943	NA	0,006013348	NA	NA	NA	NA	NA
ILMN_3239426	GPN3	NA	NA	NA	NA	0,006021496	NA	NA	NA	NA	NA
ILMN_1813846	P2RX4	NA	NA	NA	NA	0,006096937	NA	NA	NA	NA	NA
ILMN_1754538	C10orf58	0,000226791	0,008136686	0,000922165	NA	0,006173431	NA	NA	NA	NA	NA
ILMN_1656868	LOC23117	NA	NA	0,002053595	NA	0,006284631	NA	NA	NA	NA	NA
ILMN_2346460	NARG2	NA	NA	0,00236151	NA	0,006521124	NA	NA	NA	NA	NA
ILMN_3245659	ERI1	NA	NA	NA	NA	0,006672758	NA	NA	NA	NA	NA
ILMN_1787657	CLDN12	0,002129017	0,009072421	0,001185132	NA	0,006704714	NA	NA	NA	NA	NA
ILMN_2349393	MDK	NA	NA	NA	NA	0,006832811	NA	NA	NA	NA	NA
ILMN_2362858	PILRA	NA	NA	NA	NA	0,006894376	NA	NA	NA	NA	NA
ILMN_1687107	RFWD3	0,003525675	NA	0,000355583	NA	0,006990303	NA	NA	NA	NA	NA
ILMN_1664921	PPP6C	NA	NA	0,004764143	NA	0,00700301	NA	NA	NA	NA	NA
ILMN_1771732	AS3MT	NA	NA	0,001433568	NA	0,00700375	NA	NA	NA	NA	NA
ILMN_1758672	FAM107B	NA	NA	0,00014478	NA	0,007097981	NA	NA	NA	NA	NA
ILMN_2351930	TRIM33	NA	NA	NA	NA	0,007120042	NA	NA	NA	NA	NA
ILMN_1711566	TIMP1	NA	NA	NA	NA	0,007194054	NA	NA	NA	NA	NA
ILMN_1676358	RALB	NA	NA	0,001417878	NA	0,007207442	NA	NA	NA	NA	NA
ILMN_1670379	ANTXR1	NA	NA	NA	NA	0,007216551	NA	NA	NA	NA	NA
ILMN_2108823	FZD7	NA	NA	0,005989971	NA	0,007248842	NA	NA	NA	NA	NA
ILMN_1664602	LOC143543	NA	NA	0,001884556	NA	0,007285583	NA	NA	NA	NA	NA
ILMN_1654099	LRIG2	NA	NA	0,007931312	NA	0,007328557	NA	NA	NA	NA	NA
ILMN_2339266	LAMA2	NA	NA	0,001975374	NA	0,007406172	NA	NA	NA	NA	NA
ILMN_1744048	LOC652455	NA	NA	NA	NA	0,007499488	NA	NA	NA	NA	NA
ILMN_2259633	MLL5	NA	NA	NA	NA	0,007500463	NA	NA	NA	NA	NA
ILMN_1694888	TAF2	NA	NA	NA	NA	0,007535153	NA	NA	NA	NA	NA
ILMN_1675124	DDX17	0,005685628	NA	0,002085268	NA	0,007542724	NA	NA	NA	NA	NA
ILMN_1776516	ITPKA	0,000663893	NA	9,52E-05	NA	0,007555643	NA	NA	NA	NA	NA
ILMN_2052208	GADD45A	NA	NA	0,00010968	NA	0,007623395	NA	NA	NA	NA	NA
ILMN_2120072	FLJ13305	0,001805131	NA	0,004243406	NA	0,00769321	NA	NA	NA	NA	NA
ILMN_2288483	C3orf34	NA	NA	0,003977905	NA	0,008498021	NA	NA	NA	NA	NA
ILMN_1811702	GRN	NA	NA	NA	NA	0,008510855	NA	NA	NA	NA	NA
ILMN_1672717	C10orf57	NA	NA	NA	NA	0,008511721	NA	NA	NA	NA	NA
ILMN_2383707	ALDH1A2	NA	NA	0,001372766	NA	0,0085862	NA	NA	NA	NA	NA
ILMN_1705928	SNRNP200	NA	NA	NA	NA	0,008707521	NA	NA	NA	NA	NA
ILMN_2403730	ATP6V1H	NA	NA	NA	NA	0,00875132	NA	NA	NA	NA	NA
ILMN_2053527	PARP9	NA	NA	0,004099339	NA	0,008944918	NA	NA	NA	NA	NA
ILMN_1810486	RAB34	NA	NA	NA	NA	0,009147437	NA	NA	NA	NA	NA
ILMN_2052790	NONO	NA	NA	NA	NA	0,009193153	NA	NA	NA	NA	NA
ILMN_1678423	SPA17	NA	NA	NA	NA	0,009224815	NA	NA	NA	NA	NA
ILMN_2209578	CTGLF3	NA	NA	NA	NA	0,009251537	NA	NA	NA	NA	NA
ILMN_1652073	LOC653658	NA	NA	NA	NA	0,009253541	NA	NA	NA	NA	NA
ILMN_1748770	CKAP5	NA	NA	NA	NA	0,009271331	NA	NA	NA	NA	NA
ILMN_1694399	ICA1	NA	NA	NA	NA	0,009314	NA	NA	NA	NA	NA
ILMN_1738383	EEF2	NA	NA	NA	NA	0,009333023	NA	NA	NA	NA	NA
ILMN_1685496	RGS7	NA	NA	0,003096455	NA	0,009426134	NA	NA	NA	NA	NA
ILMN_1824362		NA	NA	NA	NA	0,009469145	NA	NA	NA	NA	NA
ILMN_1665510	ERRFI1	NA	NA	0,006920217	NA	0,009481104	NA	NA	NA	NA	NA
ILMN_3294074	LOC338870	NA	NA	0,000873825	NA	0,009583909	NA	NA	NA	NA	NA
ILMN_1678494	ZNF438	NA	NA	0,008031201	NA	0,009646218	NA	NA	NA	NA	NA
ILMN_1685371	SUMF2	NA	NA	NA	NA	0,009650749	NA	NA	NA	NA	NA
ILMN_1696087	PHB2	0,002393249	NA	0,002709621	NA	0,009661615	NA	NA	NA	NA	NA
ILMN_2077130	MSH3	NA	NA	0,002689833	NA	0,009858998	NA	NA	NA	NA	NA
ILMN_1671217	LOC440733	0,005523467	NA	0,002915634	NA	0,009870269	NA	NA	NA	NA	NA
ILMN_3236680	LOC1001343	NA	NA	0,008177742	NA	0,009892267	NA	NA	NA	NA	NA
ILMN_2350970	SOCS5	NA	NA	0,001333306	NA	0,009898216	NA	NA	NA	NA	NA
ILMN_1699503	UBE2D2	NA	NA	NA	5,52E-05	NA	NA	NA	NA	NA	NA
ILMN_2184789	HSCB	NA	NA	NA	8,52E-05	NA	NA	NA	NA	NA	NA
ILMN_1659976	CLP1	NA	NA	NA	0,00016717	NA	NA	NA	NA	NA	NA
ILMN_1804351	FZD7	NA	NA	NA	0,00024912	NA	NA	NA	NA	NA	NA
ILMN_1732216	NARS	NA	NA	NA	0,00025051	NA	NA	NA	NA	NA	NA
ILMN_1821280		0,006712114	NA	NA	0,00026256	NA	NA	NA	NA	NA	NA
ILMN_1727740	SYNCRIP	NA	NA	NA	0,00037868	NA	NA	NA	NA	NA	NA
ILMN_1711199	ZNF331	NA	NA	NA	0,00040147	NA	NA	NA	NA	NA	NA
ILMN_1791306	C9orf103	NA	NA	NA	0,00043915	NA	NA	NA	NA	NA	NA
ILMN_1739210	NSL1	NA	NA	NA	0,00045849	NA	NA	NA	NA	NA	NA
ILMN_2362232	ZNF331	NA	NA	NA	0,00053179	NA	NA	NA	NA	NA	NA
ILMN_1768293	NUP155	NA	NA	NA	0,0006544	NA	NA	NA	NA	NA	NA
ILMN_1659937	ZBTB24	NA	NA	NA	0,00136922	NA	NA	NA	NA	NA	NA
ILMN_3246004	LOC1001336	NA	NA	NA	0,0014459	NA	NA	NA	NA	NA	NA
ILMN_1897864		NA	NA	NA	0,00147705	NA	NA	NA	NA	NA	NA
ILMN_1696065	SDF4	NA	NA	NA	0,00163071	NA	NA	NA	NA	NA	NA
ILMN_1652371	KIAA1324L	NA	NA	NA	0,0016475	NA	NA	NA	NA	NA	NA
ILMN_1656386	SEC24D	NA	NA	NA	0,00166236	NA	NA	NA	NA	NA	NA
ILMN_2115633	CD320	NA	NA	NA	0,00174985	NA	NA	NA	NA	NA	NA
ILMN_1730329	CRSP6	0,009559629	NA	NA	0,00175925	NA	NA	NA	NA	NA	NA
ILMN_1669201	ABCF2	NA	NA	NA	0,00175945	NA	NA	NA	NA	NA	NA
ILMN_1772261	GLG1	NA	NA	NA	0,00185141	NA	NA	NA	NA	NA	NA
ILMN_1765801	GAA	NA	NA	NA	0,00185797	NA	NA	NA	NA	NA	NA

ILMN_2388142	CD99L2	NA	NA	NA		0,00187778	NA	NA	NA	NA	NA	NA
ILMN_1697694	ATP6AP1	NA	NA	NA		0,00196212	NA	NA	NA	NA	NA	NA
ILMN_1690911	C16orf87	NA	NA	NA		0,00203055	NA	NA	NA	NA	NA	NA
ILMN_2220187	GFPT1	NA	NA	NA		0,00226342	NA	NA	NA	NA	NA	NA
ILMN_1718171	MED26	NA	NA	NA		0,00232004	NA	NA	NA	NA	NA	NA
ILMN_2220184	GFPT1	NA	NA	NA		0,00243869	NA	NA	NA	NA	NA	NA
ILMN_1737343	FNIP1	NA	NA		0,00815387	0,00250418	NA	NA	NA	NA	NA	NA
ILMN_1806032	SLC25A16	NA	NA	NA		0,0029129	NA	NA	NA	NA	NA	NA
ILMN_2077905	PTGFRN	NA	NA	NA		0,00301753	NA	NA	NA	NA	NA	NA
ILMN_1718629	NRIP1	NA	NA	NA		0,00310397	NA	NA	NA	NA	NA	NA
ILMN_1736481	SECISBP2	NA	NA	NA		0,00322563	NA	NA	NA	NA	NA	NA
ILMN_3237319	LOC678655	NA	NA	NA		0,00340617	NA	NA	NA	NA	NA	NA
ILMN_1755383	LRRC1	NA	NA	NA		0,00351311	NA	NA	NA	NA	NA	NA
ILMN_1769282	FRMD6	NA	NA	NA		0,00364139	NA	NA	NA	NA	NA	NA
ILMN_2415572	GALC	NA	NA	NA		0,00389691	NA	NA	NA	NA	NA	NA
ILMN_1686750	MGEA5	NA	NA	NA		0,00393419	NA	NA	NA	NA	NA	NA
ILMN_1800602	GCA	NA	NA	NA		0,00400729	NA	NA	NA	NA	NA	NA
ILMN_1706873	RPL34	NA	NA	NA		0,0041065	NA	NA	NA	NA	NA	NA
ILMN_1745021	SLC30A1	NA	NA		0,001556077	0,00419762	NA	NA	NA	NA	NA	NA
ILMN_1812474	TFG	NA	NA	NA		0,00429012	NA	NA	NA	NA	NA	NA
ILMN_1743747	RUSC1	NA	NA	NA		0,00429519	NA	NA	NA	NA	NA	NA
ILMN_1665164	CTR9	NA	NA	NA		0,0043217	NA	NA	NA	NA	NA	NA
ILMN_1677376	CHD7	NA	NA	NA		0,00443793	NA	NA	NA	NA	NA	NA
ILMN_2410783	GAA	NA	NA	NA		0,00447068	NA	NA	NA	NA	NA	NA
ILMN_1757408	ZNF256	NA	NA	NA		0,00453346	NA	NA	NA	NA	NA	NA
ILMN_1788416	FAM108C1	NA	NA	NA		0,00454972	NA	NA	NA	NA	NA	NA
ILMN_2347943	ZNF384	NA	NA	NA		0,00458608	NA	NA	NA	NA	NA	NA
ILMN_1732410	SLC16A9	NA	NA	NA		0,00462383	NA	NA	NA	NA	NA	NA
ILMN_1678781	SNX26	NA	NA	NA		0,00464636	NA	NA	NA	NA	NA	NA
ILMN_1899903		NA	NA	NA		0,00472504	NA	NA	NA	NA	NA	NA
ILMN_2388363	IMPDH1	NA	NA	NA		0,00479112	NA	NA	NA	NA	NA	NA
ILMN_2186369	NCOR1	0,003359208	NA	NA		0,00490948	NA	NA	NA	NA	NA	NA
ILMN_1766123	FHIT	NA	NA	NA		0,00493985	NA	NA	NA	NA	NA	NA
ILMN_1797893	PFAAP5	NA	NA	NA		0,00500097	NA	NA	NA	NA	NA	NA
ILMN_2090397	ISG20L2	NA	NA	NA		0,00505935	NA	NA	NA	NA	NA	NA
ILMN_2067852	SLC30A1	NA	NA		0,007115705	0,00507667	NA	NA	NA	NA	NA	NA
ILMN_2087575	ZC3H4	NA	NA	NA		0,00507949	NA	NA	NA	NA	NA	NA
ILMN_1683932	ZNF425	NA	NA	NA		0,0050807	NA	NA	NA	NA	NA	NA
ILMN_1800540	CD55	NA	NA	NA		0,00510536	NA	NA	NA	NA	NA	NA
ILMN_1773741	GOLGA5	NA	NA	NA		0,00517193	NA	NA	NA	NA	NA	NA
ILMN_1693421	RPN2	NA	NA	NA		0,00520011	NA	NA	NA	NA	NA	NA
ILMN_2112402	PHF5A	NA	NA	NA		0,00526104	NA	NA	NA	NA	NA	NA
ILMN_1666545	GCNT1	NA	NA	NA		0,00526956	NA	NA	NA	NA	NA	NA
ILMN_1809583	CREBBP	NA	NA	NA		0,00527592	NA	NA	NA	NA	NA	NA
ILMN_2052871	TMEM116	NA	NA	NA		0,00530226	NA	NA	NA	NA	NA	NA
ILMN_1669376	DRAM1	NA	NA	NA		0,00537047	NA	NA	NA	NA	NA	NA
ILMN_2228732	CCNG2	0,001515727	NA		0,005919213	0,00543266	NA	NA	NA	NA	NA	NA
ILMN_3225591	RPL14L	NA	NA	NA		0,00547669	NA	NA	NA	NA	NA	NA
ILMN_1806023	JUN	NA	NA	NA		0,00554765	NA	NA	NA	NA	NA	NA
ILMN_1679971	CCDC123	NA	NA	NA		0,00567743	NA	NA	NA	NA	NA	NA
ILMN_1730631	C2orf44	NA	NA		0,008481403	0,00584575	NA	NA	NA	NA	NA	NA
ILMN_1750974	S100A9	NA	NA	NA		0,00592714	NA	NA	NA	NA	NA	NA
ILMN_1652989	NUP160	NA	NA	NA		0,00594743	NA	NA	NA	NA	NA	NA
ILMN_1662232	DCTN2	NA	NA	NA		0,00601617	NA	NA	NA	NA	NA	NA
ILMN_1751708	ITM2B	NA	NA	NA		0,00612467	NA	NA	NA	NA	NA	NA
ILMN_2360229	MAPKAP1	NA	NA	NA		0,00618775	NA	NA	NA	NA	NA	NA
ILMN_3236866	ULK4	0,007134852	NA	NA		0,00620297	NA	NA	NA	NA	NA	NA
ILMN_1722186	C2orf56	NA	NA	NA		0,00622145	NA	NA	NA	NA	NA	NA
ILMN_1788988	THAP1	NA	NA	NA		0,00623803	NA	NA	NA	NA	NA	NA
ILMN_1733538	RGS10	NA	NA	NA		0,00631162	NA	NA	NA	NA	NA	NA
ILMN_3188124	LOC1001305	NA	NA	NA		0,00631586	NA	NA	NA	NA	NA	NA
ILMN_1669070	MIPEP	NA	NA	NA		0,00644745	NA	NA	NA	NA	NA	NA
ILMN_1701111	RP5-1022P6.	NA	NA	NA		0,0066174	NA	NA	NA	NA	NA	NA
ILMN_1788213	FRAT2	NA	NA	NA		0,00685235	NA	NA	NA	NA	NA	NA
ILMN_1781290	RHOA	NA	NA	NA		0,0069723	NA	NA	NA	NA	NA	NA
ILMN_1729639	CLDN20	NA	NA	NA		0,00709497	NA	NA	NA	NA	NA	NA
ILMN_1755077	HEBP2	NA	NA		0,006546342	0,00713581	NA	NA	NA	NA	NA	NA
ILMN_3237850	C5orf51	NA	NA	NA		0,00726559	NA	NA	NA	NA	NA	NA
ILMN_1675674	UBE4B	NA	NA	NA		0,00727949	NA	NA	NA	NA	NA	NA
ILMN_1733562	TFB1M	0,002277565	NA		0,008448513	0,00730262	NA	NA	NA	NA	NA	NA
ILMN_1749109	PSAP	NA	NA	NA		0,00767235	NA	NA	NA	NA	NA	NA
ILMN_1788607	RPL23A	NA	NA	NA		0,00788025	NA	NA	NA	NA	NA	NA
ILMN_1717524	LIPT1	NA	NA	NA		0,00812852	NA	NA	NA	NA	NA	NA
ILMN_1782292	LAMP1	NA	NA	NA		0,00814769	NA	NA	NA	NA	NA	NA
ILMN_3240177	LLPH	NA	NA	NA		0,00825089	NA	NA	NA	NA	NA	NA
ILMN_1695362	ZNF32	NA	NA	NA		0,00842616	NA	NA	NA	NA	NA	NA
ILMN_1678235	KIAA1267	NA	NA	NA		0,00848209	NA	NA	NA	NA	NA	NA
ILMN_1707720	SLC1A5	NA	NA	NA		0,0085134	NA	NA	NA	NA	NA	NA
ILMN_1730391	MRPS18A	NA	NA	NA		0,00869854	NA	NA	NA	NA	NA	NA
ILMN_1702806	PDCL3	NA	NA	NA		0,00886425	NA	NA	NA	NA	NA	NA
ILMN_1797522	DUSP3	NA	NA	NA		0,00886985	NA	NA	NA	NA	NA	NA
ILMN_1749875	LOC728715	0,001603202	NA		0,001599218	0,00907525	NA	NA	NA	NA	NA	NA
ILMN_1857113		0,007191754	NA	NA		0,00914535	NA	NA	NA	NA	NA	NA
ILMN_2216838	DKFZP586I14	NA	NA	NA		0,0092214	NA	NA	NA	NA	NA	NA
ILMN_1695276	MAPRE2	NA	NA		0,003632946	0,00926276	NA	NA	NA	NA	NA	NA
ILMN_1662263	MDP1	0,006345852	NA	NA		0,00927901	NA	NA	NA	NA	NA	NA
ILMN_1696702	NEO1	NA	NA	NA		0,00937632	NA	NA	NA	NA	NA	NA
ILMN_1700231	IP6K1	NA	NA	NA		0,00946188	NA	NA	NA	NA	NA	NA
ILMN_2354269	FAM164C	NA	NA	NA		0,00947234	NA	NA	NA	NA	NA	NA
ILMN_1671728	CARD14	NA	NA	NA		0,0095369	NA	NA	NA	NA	NA	NA
ILMN_2251978	C21orf66	NA	NA	NA		0,00969212	NA	NA	NA	NA	NA	NA
ILMN_1662318	CCDC59	NA	NA	NA		0,00982175	NA	NA	NA	NA	NA	NA
ILMN_1788180	RAB13	NA	NA	NA		0,00992949	NA	NA	NA	NA	NA	NA
ILMN_2327994	AZIN1	0,001714715	NA		4,63E-06	NA	NA	NA	NA	NA	NA	NA
ILMN_1769601	MGC16169	NA	NA		8,58E-06	NA	NA	NA	NA	NA	NA	NA
ILMN_1712082	GCNT3	0,000297729	NA		8,72E-06	NA	NA	NA	NA	NA	NA	NA
ILMN_1657624	NSFL1C	0,005727712	NA		2,51E-05	NA	NA	NA	NA	NA	NA	NA
ILMN_2366719	NSFL1C	0,00018019	NA		5,23E-05	NA	NA	NA	NA	NA	NA	NA
ILMN_3266894	LOC1001288	NA	NA		5,87E-05	NA	NA	NA	NA	NA	NA	NA
ILMN_1694686	KIAA0194	0,007049787	NA		6,10E-05	NA	NA	NA	NA	NA	NA	NA
ILMN_1693293	LOC648176	NA	NA		7,40E-05	NA	NA	NA	NA	NA	NA	NA
ILMN_2335718	HNRNPAB	0,002977959	NA		0,000111162	NA	NA	NA	NA	NA	NA	NA
ILMN_1706687	KLHL5	0,003407203	NA		0,000137955	NA	NA	NA	NA	NA	NA	NA
ILMN_1685012	EAF1	NA	NA		0,000163356	NA	NA	NA	NA	NA	NA	NA
ILMN_1701457	FAHD1	0,000830022	NA		0,000168444	NA	NA	NA	NA	NA	NA	NA
ILMN_1760121	RRAGC	NA	NA		0,000168497	NA	NA	NA	NA	NA	NA	NA
ILMN_1815885	ZFP112	6,23E-05	NA		0,000178913	NA	NA	NA	NA	NA	NA	NA
ILMN_2356672	EIF2B4	NA	NA		0,000208134	NA	NA	NA	NA	NA	NA	NA
ILMN_1721106	C14orf159	0,000513571	NA		0,000210372	NA	NA	NA	NA	NA	NA	NA

ILMN_3178792	HNRNPA2B1	0,002282001	NA	0,000225088	NA	NA	NA	NA	NA	NA	NA
ILMN_1756071	MFGE8	NA	NA	0,000274994	NA	NA	NA	NA	NA	NA	NA
ILMN_1722156	RWDD2A	0,00072046	NA	0,000288977	NA	NA	NA	NA	NA	NA	NA
ILMN_1694075	GADD45A	0,006496358	NA	0,000310611	NA	NA	NA	NA	NA	NA	NA
ILMN_1722774	VPS72	0,009926045	NA	0,000321675	NA	NA	NA	NA	NA	NA	NA
ILMN_1762095	TMTC4	NA	NA	0,000324059	NA	NA	NA	NA	NA	NA	NA
ILMN_1742332	KCTD12	NA	NA	0,000324111	NA	NA	NA	NA	NA	NA	NA
ILMN_1771139	FBXO31	0,005906107	NA	0,000331962	NA	NA	NA	NA	NA	NA	NA
ILMN_1771385	GBP4	NA	NA	0,000370838	NA	NA	NA	NA	NA	NA	NA
ILMN_1803564	YIPF1	NA	NA	0,000380562	NA	NA	NA	NA	NA	NA	NA
ILMN_2387731	NDUFV3	0,002827087	NA	0,000425162	NA	NA	NA	NA	NA	NA	NA
ILMN_1886493		NA	NA	0,000449461	NA	NA	NA	NA	NA	NA	NA
ILMN_1663664	MRPS10	NA	NA	0,000452694	NA	NA	NA	NA	NA	NA	NA
ILMN_2177460	AQR	NA	NA	0,000457671	NA	NA	NA	NA	NA	NA	NA
ILMN_1789830	CFLAR	NA	NA	0,000494009	NA	NA	NA	NA	NA	NA	NA
ILMN_3251207	TMEM19	0,007677162	NA	0,000533711	NA	NA	NA	NA	NA	NA	NA
ILMN_1758906	GNA13	0,000697401	NA	0,000538667	NA	NA	NA	NA	NA	NA	NA
ILMN_1660345	NGRN	0,005130292	NA	0,00053983	NA	NA	NA	NA	NA	NA	NA
ILMN_2044617	MTERFD1	NA	NA	0,000539926	NA	NA	NA	NA	NA	NA	NA
ILMN_2342437	KLHL5	0,003297511	NA	0,000541209	NA	NA	NA	NA	NA	NA	NA
ILMN_2079285	ATP5L	0,003121944	NA	0,000543628	NA	NA	NA	NA	NA	NA	NA
ILMN_1744442	TTPAL	0,00034335	NA	0,000563361	NA	NA	NA	NA	NA	NA	NA
ILMN_1784269	AASDH	NA	NA	0,000641698	NA	NA	NA	NA	NA	NA	NA
ILMN_2070300	LSM2	0,001931691	NA	0,000667867	NA	NA	NA	NA	NA	NA	NA
ILMN_2348788	CD44	0,002939743	NA	0,000674873	NA	NA	NA	NA	NA	NA	NA
ILMN_1665797	CSE1L	NA	NA	0,000676223	NA	NA	NA	NA	NA	NA	NA
ILMN_1744725	BTBD6	NA	NA	0,000694323	NA	NA	NA	NA	NA	NA	NA
ILMN_1655765	MRPS21	NA	NA	0,000696441	NA	NA	NA	NA	NA	NA	NA
ILMN_1803652	C9orf91	NA	NA	0,000702845	NA	NA	NA	NA	NA	NA	NA
ILMN_1811195	ZNF211	0,009514288	NA	0,000703376	NA	NA	NA	NA	NA	NA	NA
ILMN_2137789	KLF4	NA	NA	0,000705561	NA	NA	NA	NA	NA	NA	NA
ILMN_3271244	LOC1001307	0,001710887	NA	0,000721345	NA	NA	NA	NA	NA	NA	NA
ILMN_2118472	C10orf58	0,00129723	NA	0,00082515	NA	NA	NA	NA	NA	NA	NA
ILMN_2155516	QTRTD1	0,003639404	NA	0,000825761	NA	NA	NA	NA	NA	NA	NA
ILMN_1699772	RRAGD	NA	NA	0,000850822	NA	NA	NA	NA	NA	NA	NA
ILMN_2097421	MRPL51	0,000525116	NA	0,000887104	NA	NA	NA	NA	NA	NA	NA
ILMN_2047430	MARCH5	NA	NA	0,000901858	NA	NA	NA	NA	NA	NA	NA
ILMN_1687430	EIF2B4	NA	NA	0,000904482	NA	NA	NA	NA	NA	NA	NA
ILMN_1694479	WDR18	0,003312119	NA	0,000916127	NA	NA	NA	NA	NA	NA	NA
ILMN_1670079	OMA1	NA	NA	0,000943553	NA	NA	NA	NA	NA	NA	NA
ILMN_1768784	PSMC2	NA	NA	0,000947195	NA	NA	NA	NA	NA	NA	NA
ILMN_1749521	SLC35E3	NA	NA	0,00095957	NA	NA	NA	NA	NA	NA	NA
ILMN_1753980	LOC731950	NA	NA	0,000959605	NA	NA	NA	NA	NA	NA	NA
ILMN_1691506	NGRN	NA	NA	0,000966128	NA	NA	NA	NA	NA	NA	NA
ILMN_1707350	TUSC1	NA	NA	0,000984288	NA	NA	NA	NA	NA	NA	NA
ILMN_1731107	CCDC92	NA	NA	0,000994775	NA	NA	NA	NA	NA	NA	NA
ILMN_1691570	METTL5	0,006782273	NA	0,001029545	NA	NA	NA	NA	NA	NA	NA
ILMN_1691432	PRDM4	0,003229659	NA	0,001042016	NA	NA	NA	NA	NA	NA	NA
ILMN_1722522	CCNT2	0,009400956	NA	0,001053036	NA	NA	NA	NA	NA	NA	NA
ILMN_2102951	FAHD2A	0,000358297	NA	0,001056249	NA	NA	NA	NA	NA	NA	NA
ILMN_1718619	TRIM11	0,003033867	NA	0,001057391	NA	NA	NA	NA	NA	NA	NA
ILMN_1659415	MAP2K1IP1	0,002613608	NA	0,001073872	NA	NA	NA	NA	NA	NA	NA
ILMN_2367239	RCAN1	NA	NA	0,001084655	NA	NA	NA	NA	NA	NA	NA
ILMN_1791069	ZNF513	0,007621227	NA	0,0010999	NA	NA	NA	NA	NA	NA	NA
ILMN_1810901	RNASEH2A	NA	NA	0,001108313	NA	NA	NA	NA	NA	NA	NA
ILMN_1659523	USP39	NA	NA	0,001131911	NA	NA	NA	NA	NA	NA	NA
ILMN_2180239	DOPEY2	NA	NA	0,001143904	NA	NA	NA	NA	NA	NA	NA
ILMN_1788783	TRAM2	NA	NA	0,001152313	NA	NA	NA	NA	NA	NA	NA
ILMN_2301725	PDE8B	NA	NA	0,001168826	NA	NA	NA	NA	NA	NA	NA
ILMN_1703016	CACNA1G	NA	NA	0,001172026	NA	NA	NA	NA	NA	NA	NA
ILMN_1705433	CBLL1	0,002192794	NA	0,001172579	NA	NA	NA	NA	NA	NA	NA
ILMN_1770030	C7orf43	0,007788724	NA	0,001179378	NA	NA	NA	NA	NA	NA	NA
ILMN_1657632	ZMYM6	0,002121343	NA	0,001208916	NA	NA	NA	NA	NA	NA	NA
ILMN_1754990	LOC388720	0,000374745	NA	0,001249054	NA	NA	NA	NA	NA	NA	NA
ILMN_1777096	TDG	0,008398578	NA	0,001259315	NA	NA	NA	NA	NA	NA	NA
ILMN_1726981	VEGFB	NA	NA	0,001290014	NA	NA	NA	NA	NA	NA	NA
ILMN_1767658	RRS1	NA	NA	0,001291715	NA	NA	NA	NA	NA	NA	NA
ILMN_2198376	PSMA4	NA	NA	0,001297918	NA	NA	NA	NA	NA	NA	NA
ILMN_1740197	ZNF234	0,005574328	NA	0,00131978	NA	NA	NA	NA	NA	NA	NA
ILMN_1754969	LMCD1	NA	NA	0,001355733	NA	NA	NA	NA	NA	NA	NA
ILMN_1706593	PPHLN1	0,009314937	NA	0,001359405	NA	NA	NA	NA	NA	NA	NA
ILMN_2057399	ZBTB8OS	0,003412396	NA	0,001378931	NA	NA	NA	NA	NA	NA	NA
ILMN_1773847	DYNC1I2	NA	NA	0,001384226	NA	NA	NA	NA	NA	NA	NA
ILMN_3280943	LOC389156	NA	NA	0,001390678	NA	NA	NA	NA	NA	NA	NA
ILMN_1813117	ITGA9	NA	NA	0,001410531	NA	NA	NA	NA	NA	NA	NA
ILMN_1711766	SKP1A	NA	NA	0,001413508	NA	NA	NA	NA	NA	NA	NA
ILMN_1732688	DUT	0,002958911	NA	0,001420507	NA	NA	NA	NA	NA	NA	NA
ILMN_2403555	DHX30	NA	NA	0,001457875	NA	NA	NA	NA	NA	NA	NA
ILMN_1707464	MST1	NA	NA	0,001469143	NA	NA	NA	NA	NA	NA	NA
ILMN_3234116	LOC730382	NA	NA	0,001483072	NA	NA	NA	NA	NA	NA	NA
ILMN_1715607	CHMP4A	0,009716403	NA	0,001517054	NA	NA	NA	NA	NA	NA	NA
ILMN_1755808	LOC654194	NA	NA	0,001567963	NA	NA	NA	NA	NA	NA	NA
ILMN_1729179	SPATA5L1	0,007533946	NA	0,001569485	NA	NA	NA	NA	NA	NA	NA
ILMN_1791296	DPY19L1	0,005358032	NA	0,001571738	NA	NA	NA	NA	NA	NA	NA
ILMN_1689237	BVES	NA	NA	0,001596251	NA	NA	NA	NA	NA	NA	NA
ILMN_2090607	IRF2	0,001183228	NA	0,001621834	NA	NA	NA	NA	NA	NA	NA
ILMN_1665877	RNF149	NA	NA	0,00165712	NA	NA	NA	NA	NA	NA	NA
ILMN_1651262	HNRNPAB	0,003316521	NA	0,001693757	NA	NA	NA	NA	NA	NA	NA
ILMN_1815169	MCM5	NA	NA	0,001709512	NA	NA	NA	NA	NA	NA	NA
ILMN_3251672	DSG2	NA	NA	0,001715927	NA	NA	NA	NA	NA	NA	NA
ILMN_1784256	HDGFRP3	NA	NA	0,001729631	NA	NA	NA	NA	NA	NA	NA
ILMN_1752340	ARF5	0,006175997	NA	0,001741283	NA	NA	NA	NA	NA	NA	NA
ILMN_1780141	TMEM66	0,00549593	NA	0,001745554	NA	NA	NA	NA	NA	NA	NA
ILMN_1706118	HN1L	0,000150448	NA	0,001764524	NA	NA	NA	NA	NA	NA	NA
ILMN_2381296	GSTZ1	NA	NA	0,001777803	NA	NA	NA	NA	NA	NA	NA
ILMN_2079803	LSM14A	NA	NA	0,001782259	NA	NA	NA	NA	NA	NA	NA
ILMN_1664231	TIMM23	0,006025021	NA	0,001806321	NA	NA	NA	NA	NA	NA	NA
ILMN_1734826	NUP88	0,008661061	NA	0,001811382	NA	NA	NA	NA	NA	NA	NA
ILMN_1861057		NA	NA	0,001811868	NA	NA	NA	NA	NA	NA	NA
ILMN_1798826	MRPS25	0,000537127	NA	0,001831329	NA	NA	NA	NA	NA	NA	NA
ILMN_3232219	LOC1001341	0,002147215	NA	0,001838067	NA	NA	NA	NA	NA	NA	NA
ILMN_2405009	NBL1	NA	NA	0,001855466	NA	NA	NA	NA	NA	NA	NA
ILMN_2313158	MBNL1	0,000587338	NA	0,001895469	NA	NA	NA	NA	NA	NA	NA
ILMN_1781691	TRAK2	NA	NA	0,001909598	NA	NA	NA	NA	NA	NA	NA
ILMN_2072178	ECHDC3	NA	NA	0,001917473	NA	NA	NA	NA	NA	NA	NA
ILMN_1793990	ID2	0,008946071	NA	0,001920142	NA	NA	NA	NA	NA	NA	NA
ILMN_2121068	ADAM17	0,004791008	NA	0,001934838	NA	NA	NA	NA	NA	NA	NA
ILMN_2344216	STX2	0,000394907	NA	0,00196394	NA	NA	NA	NA	NA	NA	NA
ILMN_2301722	PDE8B	NA	NA	0,001969726	NA	NA	NA	NA	NA	NA	NA

ILMN_2202481	UBLCP1	NA	NA	0,001990924	NA	NA	NA	NA	NA	NA	NA
ILMN_1664750	TMBIM4	0,000721008	NA	0,002002548	NA	NA	NA	NA	NA	NA	NA
ILMN_2149766	APPBP2	NA	NA	0,002010717	NA	NA	NA	NA	NA	NA	NA
ILMN_1768812	FXYD6	NA	NA	0,002019339	NA	NA	NA	NA	NA	NA	NA
ILMN_1764964	IFNGR2	NA	NA	0,002026692	NA	NA	NA	NA	NA	NA	NA
ILMN_1756767	EIF5B	NA	NA	0,002045293	NA	NA	NA	NA	NA	NA	NA
ILMN_2319825	ACYP1	0,004393362	NA	0,002049364	NA	NA	NA	NA	NA	NA	NA
ILMN_2136635	ISCA2	0,001919774	NA	0,002068347	NA	NA	NA	NA	NA	NA	NA
ILMN_1844692	FOXO3	0,00234161	NA	0,002100301	NA	NA	NA	NA	NA	NA	NA
ILMN_1810334	COMMD7	0,000346855	NA	0,002101426	NA	NA	NA	NA	NA	NA	NA
ILMN_1694274	NDUFC2	0,000371934	NA	0,002107068	NA	NA	NA	NA	NA	NA	NA
ILMN_1743067	ZNF701	0,006565784	NA	0,002115593	NA	NA	NA	NA	NA	NA	NA
ILMN_1771964	GSTA4	NA	NA	0,002127415	NA	NA	NA	NA	NA	NA	NA
ILMN_1746465	FJX1	NA	NA	0,002132123	NA	NA	NA	NA	NA	NA	NA
ILMN_1736002	COPS5	NA	NA	0,002143461	NA	NA	NA	NA	NA	NA	NA
ILMN_1800975	PSME3	0,003162083	NA	0,002163167	NA	NA	NA	NA	NA	NA	NA
ILMN_1699610	CCT6B	NA	NA	0,002169829	NA	NA	NA	NA	NA	NA	NA
ILMN_1696485	HNRNPAB	0,004543361	NA	0,002172008	NA	NA	NA	NA	NA	NA	NA
ILMN_1700316	LOC440055	0,006743023	NA	0,002176864	NA	NA	NA	NA	NA	NA	NA
ILMN_3251445	MED27	NA	NA	0,002225301	NA	NA	NA	NA	NA	NA	NA
ILMN_3293146	LOC642975	NA	NA	0,002227082	NA	NA	NA	NA	NA	NA	NA
ILMN_2174394	MMS19L	0,001716134	NA	0,002238757	NA	NA	NA	NA	NA	NA	NA
ILMN_1803429	CD44	0,007330603	NA	0,002239083	NA	NA	NA	NA	NA	NA	NA
ILMN_1750075	DMTF1	0,001415909	NA	0,002280513	NA	NA	NA	NA	NA	NA	NA
ILMN_1803376	AEBP2	NA	NA	0,002281167	NA	NA	NA	NA	NA	NA	NA
ILMN_1778226	EXTL3	NA	NA	0,002282718	NA	NA	NA	NA	NA	NA	NA
ILMN_1740319	IFI27L2	5,53E-05	NA	0,002312082	NA	NA	NA	NA	NA	NA	NA
ILMN_1704793	MYPOP	0,00927543	NA	0,002321112	NA	NA	NA	NA	NA	NA	NA
ILMN_1787705	ATP6V1B2	NA	NA	0,002321593	NA	NA	NA	NA	NA	NA	NA
ILMN_1761594	FAM35A	6,74E-05	NA	0,002454164	NA	NA	NA	NA	NA	NA	NA
ILMN_1697117	TBP	NA	NA	0,002481031	NA	NA	NA	NA	NA	NA	NA
ILMN_3202673	LOC1001311	NA	NA	0,00256818	NA	NA	NA	NA	NA	NA	NA
ILMN_1651769	ZFYVE16	NA	NA	0,002625534	NA	NA	NA	NA	NA	NA	NA
ILMN_1689274	NIPA1	0,000174312	NA	0,002627607	NA	NA	NA	NA	NA	NA	NA
ILMN_2182335	ARID2	NA	NA	0,002630203	NA	NA	NA	NA	NA	NA	NA
ILMN_1758626	IDS	NA	NA	0,00263192	NA	NA	NA	NA	NA	NA	NA
ILMN_1767448	LHFP	NA	NA	0,002636702	NA	NA	NA	NA	NA	NA	NA
ILMN_1810922	PCNT	NA	NA	0,002668589	NA	NA	NA	NA	NA	NA	NA
ILMN_2319424	GYG2	NA	NA	0,002692628	NA	NA	NA	NA	NA	NA	NA
ILMN_1699665	CLIC6	0,009590089	NA	0,002693263	NA	NA	NA	NA	NA	NA	NA
ILMN_1688818	LOC652864	NA	NA	0,002716408	NA	NA	NA	NA	NA	NA	NA
ILMN_3260070	LOC1001282	NA	NA	0,002726211	NA	NA	NA	NA	NA	NA	NA
ILMN_2399310	MLLT10	0,000782154	NA	0,002730792	NA	NA	NA	NA	NA	NA	NA
ILMN_1755850	ZNF350	NA	NA	0,002752804	NA	NA	NA	NA	NA	NA	NA
ILMN_1698803	ZAK	0,002695119	NA	0,002775173	NA	NA	NA	NA	NA	NA	NA
ILMN_1760280	NXT1	0,000205406	NA	0,002804736	NA	NA	NA	NA	NA	NA	NA
ILMN_2130180	RPL13L	NA	NA	0,002804836	NA	NA	NA	NA	NA	NA	NA
ILMN_2210837	CDC26	NA	NA	0,002823365	NA	NA	NA	NA	NA	NA	NA
ILMN_1807304	MBNL1	0,001319501	NA	0,002892046	NA	NA	NA	NA	NA	NA	NA
ILMN_1772605	FRS3	NA	NA	0,002894071	NA	NA	NA	NA	NA	NA	NA
ILMN_1709227	CCDC84	NA	NA	0,002918534	NA	NA	NA	NA	NA	NA	NA
ILMN_1804737	RAVER2	0,000253672	NA	0,002931337	NA	NA	NA	NA	NA	NA	NA
ILMN_2061435	MEG3	NA	NA	0,00294063	NA	NA	NA	NA	NA	NA	NA
ILMN_2125562	MOBK1B	NA	NA	0,003006132	NA	NA	NA	NA	NA	NA	NA
ILMN_2348093	ATP5J	0,009147421	NA	0,003012042	NA	NA	NA	NA	NA	NA	NA
ILMN_1748651	PSMB3	NA	NA	0,003041784	NA	NA	NA	NA	NA	NA	NA
ILMN_2086095	ID2	NA	NA	0,003052751	NA	NA	NA	NA	NA	NA	NA
ILMN_2301624	MACF1	NA	NA	0,003057646	NA	NA	NA	NA	NA	NA	NA
ILMN_1775074	TUBGCP2	NA	NA	0,003086974	NA	NA	NA	NA	NA	NA	NA
ILMN_1672124	C4orf18	NA	NA	0,003103204	NA	NA	NA	NA	NA	NA	NA
ILMN_1698038	FAM188B	NA	NA	0,003141213	NA	NA	NA	NA	NA	NA	NA
ILMN_3178252	LOC1001293	NA	NA	0,003198035	NA	NA	NA	NA	NA	NA	NA
ILMN_1774027	ZNF37A	NA	NA	0,003205777	NA	NA	NA	NA	NA	NA	NA
ILMN_1679133	SERPINB1	NA	NA	0,003208325	NA	NA	NA	NA	NA	NA	NA
ILMN_1753393	OSGEP	NA	NA	0,003242239	NA	NA	NA	NA	NA	NA	NA
ILMN_1747775	STX2	0,002194988	NA	0,003272497	NA	NA	NA	NA	NA	NA	NA
ILMN_1719256	CKS1B	NA	NA	0,003291403	NA	NA	NA	NA	NA	NA	NA
ILMN_1748538	ALDH1A2	NA	NA	0,003296525	NA	NA	NA	NA	NA	NA	NA
ILMN_1752899	BCL11A	NA	NA	0,003354531	NA	NA	NA	NA	NA	NA	NA
ILMN_1757736	IRX5	NA	NA	0,003355302	NA	NA	NA	NA	NA	NA	NA
ILMN_1779147	ENC1	NA	NA	0,003363728	NA	NA	NA	NA	NA	NA	NA
ILMN_1768197	ROD1	0,00753112	NA	0,003383049	NA	NA	NA	NA	NA	NA	NA
ILMN_1692956	LOC643949	NA	NA	0,003385364	NA	NA	NA	NA	NA	NA	NA
ILMN_2344204	PRR13	0,000318445	NA	0,003390106	NA	NA	NA	NA	NA	NA	NA
ILMN_1811188	BCCIP	NA	NA	0,003408019	NA	NA	NA	NA	NA	NA	NA
ILMN_1722855	VEGFB	NA	NA	0,003436022	NA	NA	NA	NA	NA	NA	NA
ILMN_1706238	CSE1L	NA	NA	0,003449387	NA	NA	NA	NA	NA	NA	NA
ILMN_1745471	IRF9	0,000129539	NA	0,003457356	NA	NA	NA	NA	NA	NA	NA
ILMN_3242448	SNORA11D	0,000768419	NA	0,003470298	NA	NA	NA	NA	NA	NA	NA
ILMN_1751079	TAP1	0,00039907	NA	0,00347038	NA	NA	NA	NA	NA	NA	NA
ILMN_3247781	LOC1001323	NA	NA	0,003480282	NA	NA	NA	NA	NA	NA	NA
ILMN_2311674	ADCY6	NA	NA	0,003482255	NA	NA	NA	NA	NA	NA	NA
ILMN_1694530	DDA1	9,30E-05	NA	0,003492705	NA	NA	NA	NA	NA	NA	NA
ILMN_1872419		0,005988983	NA	0,003520873	NA	NA	NA	NA	NA	NA	NA
ILMN_1785756	LOC731314	0,009427437	NA	0,003551343	NA	NA	NA	NA	NA	NA	NA
ILMN_1724686	CLDN1	NA	NA	0,003568681	NA	NA	NA	NA	NA	NA	NA
ILMN_1696870	TGFBRAP1	NA	NA	0,003574385	NA	NA	NA	NA	NA	NA	NA
ILMN_2387696	CCR6	NA	NA	0,003580692	NA	NA	NA	NA	NA	NA	NA
ILMN_2096322	ADIPOR1	NA	NA	0,003600457	NA	NA	NA	NA	NA	NA	NA
ILMN_1721770	PAPPA	NA	NA	0,003605668	NA	NA	NA	NA	NA	NA	NA
ILMN_2278653	ZNF493	0,000258898	NA	0,003611573	NA	NA	NA	NA	NA	NA	NA
ILMN_3246805	LOC1001343	NA	NA	0,003618681	NA	NA	NA	NA	NA	NA	NA
ILMN_2357386	FKTN	NA	NA	0,003669493	NA	NA	NA	NA	NA	NA	NA
ILMN_1774733	SOCS1	NA	NA	0,003670698	NA	NA	NA	NA	NA	NA	NA
ILMN_3263329	GAR1	NA	NA	0,003714335	NA	NA	NA	NA	NA	NA	NA
ILMN_2046730	S100A10	NA	NA	0,003729053	NA	NA	NA	NA	NA	NA	NA
ILMN_3246766	LOC1001322	NA	NA	0,00375406	NA	NA	NA	NA	NA	NA	NA
ILMN_2089616	FBXO10	NA	NA	0,003775099	NA	NA	NA	NA	NA	NA	NA
ILMN_3238560	IFI27L2	0,000102184	NA	0,003833877	NA	NA	NA	NA	NA	NA	NA
ILMN_2041190	F2RL1	NA	NA	0,003846036	NA	NA	NA	NA	NA	NA	NA
ILMN_1786470	C1orf74	NA	NA	0,003873878	NA	NA	NA	NA	NA	NA	NA
ILMN_1741632	RAB3IL1	NA	NA	0,003887147	NA	NA	NA	NA	NA	NA	NA
ILMN_1659544	STX3	0,002783157	NA	0,003897127	NA	NA	NA	NA	NA	NA	NA
ILMN_1664577	DLD	NA	NA	0,003910072	NA	NA	NA	NA	NA	NA	NA
ILMN_1661306	LOC643433	NA	NA	0,003911588	NA	NA	NA	NA	NA	NA	NA
ILMN_1706734	ZNF451	0,002753508	NA	0,003933084	NA	NA	NA	NA	NA	NA	NA
ILMN_1714278	C9orf30	NA	NA	0,003960587	NA	NA	NA	NA	NA	NA	NA
ILMN_3241164	KDSR	NA	NA	0,003972862	NA	NA	NA	NA	NA	NA	NA
ILMN_3251686	UBTD2	0,009286662	NA	0,004066343	NA	NA	NA	NA	NA	NA	NA

ILMN_1748908	PROSC	NA	NA	0,004070538	NA	NA	NA	NA	NA	NA	NA
ILMN_1724207	IVD	NA	NA	0,004113431	NA	NA	NA	NA	NA	NA	NA
ILMN_1762835	HELZ	0,007394822	NA	0,004141628	NA	NA	NA	NA	NA	NA	NA
ILMN_2278908	GGA1	NA	NA	0,004142886	NA	NA	NA	NA	NA	NA	NA
ILMN_1750507	RPL9	0,008063302	NA	0,00415175	NA	NA	NA	NA	NA	NA	NA
ILMN_1722390	CHRAC1	0,002016749	NA	0,004161653	NA	NA	NA	NA	NA	NA	NA
ILMN_1771149	MRPL19	NA	NA	0,004163631	NA	NA	NA	NA	NA	NA	NA
ILMN_1796851	FOXL2	NA	NA	0,004191958	NA	NA	NA	NA	NA	NA	NA
ILMN_1662952	ZNF687	NA	NA	0,004215602	NA	NA	NA	NA	NA	NA	NA
ILMN_1762002	CSTF3	NA	NA	0,004290007	NA	NA	NA	NA	NA	NA	NA
ILMN_1788363	MLH1	NA	NA	0,004305194	NA	NA	NA	NA	NA	NA	NA
ILMN_1739876	RAB3GAP1	0,001776609	NA	0,004337068	NA	NA	NA	NA	NA	NA	NA
ILMN_1787808	CEP63	NA	NA	0,004339695	NA	NA	NA	NA	NA	NA	NA
ILMN_1774604	PNKD	0,000148794	NA	0,004398071	NA	NA	NA	NA	NA	NA	NA
ILMN_1718863	KCNK1	NA	NA	0,004398337	NA	NA	NA	NA	NA	NA	NA
ILMN_1671839	TAF1C	NA	NA	0,004400722	NA	NA	NA	NA	NA	NA	NA
ILMN_3243664	LOC440353	NA	NA	0,004403599	NA	NA	NA	NA	NA	NA	NA
ILMN_1708660	RWDD4A	NA	NA	0,004443987	NA	NA	NA	NA	NA	NA	NA
ILMN_2098743	THEM2	NA	NA	0,004449693	NA	NA	NA	NA	NA	NA	NA
ILMN_2337058	PORCN	0,007737822	NA	0,004451304	NA	NA	NA	NA	NA	NA	NA
ILMN_2117223	ROD1	NA	NA	0,004466877	NA	NA	NA	NA	NA	NA	NA
ILMN_3307930	RAN	NA	NA	0,004566285	NA	NA	NA	NA	NA	NA	NA
ILMN_3284114	LOC399748	0,002831952	NA	0,004595201	NA	NA	NA	NA	NA	NA	NA
ILMN_1730201	DTNA	NA	NA	0,00464986	NA	NA	NA	NA	NA	NA	NA
ILMN_3241091	LOC1001308	NA	NA	0,004661786	NA	NA	NA	NA	NA	NA	NA
ILMN_1799082	ZNF609	0,001098501	NA	0,004674754	NA	NA	NA	NA	NA	NA	NA
ILMN_1677843	RAB24	NA	NA	0,004678846	NA	NA	NA	NA	NA	NA	NA
ILMN_1808591	LOC731049	0,001781449	NA	0,004692869	NA	NA	NA	NA	NA	NA	NA
ILMN_3307791	FAM69A	NA	NA	0,00476722	NA	NA	NA	NA	NA	NA	NA
ILMN_1777895	LRRC37B	NA	NA	0,004777735	NA	NA	NA	NA	NA	NA	NA
ILMN_1661002	RFWD2	NA	NA	0,004782848	NA	NA	NA	NA	NA	NA	NA
ILMN_3227912	LOC728576	0,002837607	NA	0,004785237	NA	NA	NA	NA	NA	NA	NA
ILMN_1809285	DCP1A	NA	NA	0,004798299	NA	NA	NA	NA	NA	NA	NA
ILMN_1795905	ZBTB4	NA	NA	0,004798472	NA	NA	NA	NA	NA	NA	NA
ILMN_3288161	LOC1001324	0,004205605	NA	0,004881789	NA	NA	NA	NA	NA	NA	NA
ILMN_1756937	ST8SIA4	NA	NA	0,004900714	NA	NA	NA	NA	NA	NA	NA
ILMN_1768311	LOC728888	NA	NA	0,004931648	NA	NA	NA	NA	NA	NA	NA
ILMN_1703140	NEDD4	NA	NA	0,004954412	NA	NA	NA	NA	NA	NA	NA
ILMN_1652955	LOC648622	NA	NA	0,004971662	NA	NA	NA	NA	NA	NA	NA
ILMN_1789599	NBL1	NA	NA	0,004971842	NA	NA	NA	NA	NA	NA	NA
ILMN_2364131	TTPAL	0,000378168	NA	0,004982076	NA	NA	NA	NA	NA	NA	NA
ILMN_1666502	SOBP	0,000118517	NA	0,005004169	NA	NA	NA	NA	NA	NA	NA
ILMN_2146372	KCTD6	NA	NA	0,005015944	NA	NA	NA	NA	NA	NA	NA
ILMN_1695899	LOC648659	NA	NA	0,005016807	NA	NA	NA	NA	NA	NA	NA
ILMN_1739798	C7orf30	0,000775167	NA	0,005120591	NA	NA	NA	NA	NA	NA	NA
ILMN_1748438	POLR2G	NA	NA	0,005130504	NA	NA	NA	NA	NA	NA	NA
ILMN_3265343	LOC1001306	0,000386368	NA	0,005131571	NA	NA	NA	NA	NA	NA	NA
ILMN_1812638	ATP5L	0,00042651	NA	0,005147489	NA	NA	NA	NA	NA	NA	NA
ILMN_2064898	CCDC56	4,65E-05	NA	0,005176146	NA	NA	NA	NA	NA	NA	NA
ILMN_1805658	LTV1	NA	NA	0,00522497	NA	NA	NA	NA	NA	NA	NA
ILMN_1660718	GABBR2	NA	NA	0,005240197	NA	NA	NA	NA	NA	NA	NA
ILMN_2098325	C8orf33	NA	NA	0,00527014	NA	NA	NA	NA	NA	NA	NA
ILMN_1765122	MAP3K2	NA	NA	0,005299782	NA	NA	NA	NA	NA	NA	NA
ILMN_2385647	ALAS1	0,001539049	NA	0,005354357	NA	NA	NA	NA	NA	NA	NA
ILMN_1753500	ARHGAP12	0,001599162	NA	0,005372705	NA	NA	NA	NA	NA	NA	NA
ILMN_2160819	RPS27	NA	NA	0,005381757	NA	NA	NA	NA	NA	NA	NA
ILMN_1786834	PRKX	0,001594206	NA	0,005401567	NA	NA	NA	NA	NA	NA	NA
ILMN_1757552	PTRF	NA	NA	0,005402041	NA	NA	NA	NA	NA	NA	NA
ILMN_1744059	DCTN6	0,005369735	NA	0,005404117	NA	NA	NA	NA	NA	NA	NA
ILMN_2124471	SLC36A1	0,007871211	NA	0,005433806	NA	NA	NA	NA	NA	NA	NA
ILMN_1737635	RAD1	NA	NA	0,005435962	NA	NA	NA	NA	NA	NA	NA
ILMN_1736548	PHACTR4	NA	NA	0,00548115	NA	NA	NA	NA	NA	NA	NA
ILMN_1668090	STK25	NA	NA	0,005483013	NA	NA	NA	NA	NA	NA	NA
ILMN_1719599	SYTL4	NA	NA	0,005490864	NA	NA	NA	NA	NA	NA	NA
ILMN_1787345	FKBP11	NA	NA	0,005491111	NA	NA	NA	NA	NA	NA	NA
ILMN_2192385	TTC19	6,04E-06	NA	0,00550313	NA	NA	NA	NA	NA	NA	NA
ILMN_1795697	PTP4A2	0,009840656	NA	0,005515799	NA	NA	NA	NA	NA	NA	NA
ILMN_1760490	ACVR1	NA	NA	0,005518732	NA	NA	NA	NA	NA	NA	NA
ILMN_1792978	HAS2	NA	NA	0,005519964	NA	NA	NA	NA	NA	NA	NA
ILMN_1741564	DCTN4	NA	NA	0,005553304	NA	NA	NA	NA	NA	NA	NA
ILMN_1714364	PTK2	NA	NA	0,005562692	NA	NA	NA	NA	NA	NA	NA
ILMN_1754195	RPL31	NA	NA	0,005578987	NA	NA	NA	NA	NA	NA	NA
ILMN_1759792	CLIP4	NA	NA	0,005584675	NA	NA	NA	NA	NA	NA	NA
ILMN_2389876	TGFB1I1	NA	NA	0,005608073	NA	NA	NA	NA	NA	NA	NA
ILMN_1869087		NA	NA	0,005608162	NA	NA	NA	NA	NA	NA	NA
ILMN_1780026	ZNF430	NA	NA	0,005621621	NA	NA	NA	NA	NA	NA	NA
ILMN_1744713	PARK7	0,00463403	NA	0,005632388	NA	NA	NA	NA	NA	NA	NA
ILMN_1751338	NUP133	NA	NA	0,005638586	NA	NA	NA	NA	NA	NA	NA
ILMN_3248707	KCNQ1OT1	NA	NA	0,005652747	NA	NA	NA	NA	NA	NA	NA
ILMN_1666564	LOC652489	NA	NA	0,005669977	NA	NA	NA	NA	NA	NA	NA
ILMN_1811991	C17orf101	0,008961255	NA	0,005698846	NA	NA	NA	NA	NA	NA	NA
ILMN_1805826	BIVM	NA	NA	0,005700797	NA	NA	NA	NA	NA	NA	NA
ILMN_1673113	F2RL1	NA	NA	0,005725168	NA	NA	NA	NA	NA	NA	NA
ILMN_1770548	CLASP2	NA	NA	0,005735658	NA	NA	NA	NA	NA	NA	NA
ILMN_2093427	SHF	NA	NA	0,005738343	NA	NA	NA	NA	NA	NA	NA
ILMN_2374633	ZWILCH	NA	NA	0,0057926	NA	NA	NA	NA	NA	NA	NA
ILMN_1689446	EIF3G	0,004171817	NA	0,005835384	NA	NA	NA	NA	NA	NA	NA
ILMN_1661174	LOC731640	NA	NA	0,005884231	NA	NA	NA	NA	NA	NA	NA
ILMN_2107004	GPR1	NA	NA	0,005925111	NA	NA	NA	NA	NA	NA	NA
ILMN_1772278	TANC1	0,004219842	NA	0,005938857	NA	NA	NA	NA	NA	NA	NA
ILMN_2139100	SHISA5	NA	NA	0,005941683	NA	NA	NA	NA	NA	NA	NA
ILMN_3263918	LOC1001292	0,003428622	NA	0,005942073	NA	NA	NA	NA	NA	NA	NA
ILMN_1680782	PATL1	NA	NA	0,005948423	NA	NA	NA	NA	NA	NA	NA
ILMN_2396956	AKAP13	0,006739189	NA	0,005958795	NA	NA	NA	NA	NA	NA	NA
ILMN_1740493	TRAF5	0,004395147	NA	0,005963584	NA	NA	NA	NA	NA	NA	NA
ILMN_1716264	ANKRD1	NA	NA	0,005997347	NA	NA	NA	NA	NA	NA	NA
ILMN_2376625	VHL	0,001733168	NA	0,00600648	NA	NA	NA	NA	NA	NA	NA
ILMN_2197030	ZFYVE21	NA	NA	0,00602616	NA	NA	NA	NA	NA	NA	NA
ILMN_2412521	KIAA0101	NA	NA	0,006027748	NA	NA	NA	NA	NA	NA	NA
ILMN_1671452	MRPL44	0,009678446	NA	0,006037856	NA	NA	NA	NA	NA	NA	NA
ILMN_1679727	CLK1	NA	NA	0,006045387	NA	NA	NA	NA	NA	NA	NA
ILMN_1758728	FANCG	NA	NA	0,006081852	NA	NA	NA	NA	NA	NA	NA
ILMN_2404539	C20orf30	NA	NA	0,006091743	NA	NA	NA	NA	NA	NA	NA
ILMN_1745034	SLC11A2	0,004260303	NA	0,006119883	NA	NA	NA	NA	NA	NA	NA
ILMN_1798081	PTPRF	NA	NA	0,006135643	NA	NA	NA	NA	NA	NA	NA
ILMN_1797082	SNX13	9,54E-05	NA	0,006149273	NA	NA	NA	NA	NA	NA	NA
ILMN_2207328	C18orf10	0,000482739	NA	0,006168416	NA	NA	NA	NA	NA	NA	NA
ILMN_1688702	PJA2	NA	NA	0,006213715	NA	NA	NA	NA	NA	NA	NA
ILMN_1713756	GLUD1	NA	NA	0,00623381	NA	NA	NA	NA	NA	NA	NA

ILMN_1805737	PFKP	NA	NA		0,006248518	NA	NA	NA	NA	NA	NA	NA
ILMN_1739943	SBNO1	0,000292681	NA		0,006255705	NA	NA	NA	NA	NA	NA	NA
ILMN_2317580	SHANK3	NA	NA		0,006277985	NA	NA	NA	NA	NA	NA	NA
ILMN_3225669	RPS10P3	0,009460588	NA		0,006283149	NA	NA	NA	NA	NA	NA	NA
ILMN_1671250	CLIC4	NA	NA		0,006283604	NA	NA	NA	NA	NA	NA	NA
ILMN_2102330	COL8A2	NA	NA		0,006313645	NA	NA	NA	NA	NA	NA	NA
ILMN_1800898	ARG2	0,001187332	NA		0,006324739	NA	NA	NA	NA	NA	NA	NA
ILMN_3307742	CAPS2	NA	NA		0,006392146	NA	NA	NA	NA	NA	NA	NA
ILMN_1686968	ZNF362	NA	NA		0,00641942	NA	NA	NA	NA	NA	NA	NA
ILMN_1784678	PBX1	0,007974078	NA		0,006464851	NA	NA	NA	NA	NA	NA	NA
ILMN_1779633	PSMD6	0,006270991	NA		0,006500196	NA	NA	NA	NA	NA	NA	NA
ILMN_1788122	GSTA5	NA	NA		0,006518249	NA	NA	NA	NA	NA	NA	NA
ILMN_1734915	C1orf156	0,006892258	NA		0,006552444	NA	NA	NA	NA	NA	NA	NA
ILMN_3236713	SNHG1	NA	NA		0,006574376	NA	NA	NA	NA	NA	NA	NA
ILMN_2206812	CCNJ	NA	NA		0,006588786	NA	NA	NA	NA	NA	NA	NA
ILMN_1782761	ARHGAP20	NA	NA		0,00660157	NA	NA	NA	NA	NA	NA	NA
ILMN_3275590	LOC647302	0,000885449	NA		0,00660358	NA	NA	NA	NA	NA	NA	NA
ILMN_1674050	COL8A2	NA	NA		0,006607862	NA	NA	NA	NA	NA	NA	NA
ILMN_1797005	PGLS	NA	NA		0,006620206	NA	NA	NA	NA	NA	NA	NA
ILMN_1666967	BRP44L	NA	NA		0,006664695	NA	NA	NA	NA	NA	NA	NA
ILMN_1728467	GHITM	NA	NA		0,006670818	NA	NA	NA	NA	NA	NA	NA
ILMN_1682428	C1orf59	NA	NA		0,006684413	NA	NA	NA	NA	NA	NA	NA
ILMN_1760315	VWCE	NA	NA		0,006687728	NA	NA	NA	NA	NA	NA	NA
ILMN_1784661	TMEM2	NA	NA		0,006741054	NA	NA	NA	NA	NA	NA	NA
ILMN_2371055	EFNA1	NA	NA		0,006761475	NA	NA	NA	NA	NA	NA	NA
ILMN_1754531	AP4E1	NA	NA		0,006768809	NA	NA	NA	NA	NA	NA	NA
ILMN_1716862	PPM1B	NA	NA		0,006802375	NA	NA	NA	NA	NA	NA	NA
ILMN_1730685	MRPL16	NA	NA		0,006807143	NA	NA	NA	NA	NA	NA	NA
ILMN_2394381	CLN3	NA	NA		0,006813639	NA	NA	NA	NA	NA	NA	NA
ILMN_3202396	LOC390735	NA	NA		0,006832207	NA	NA	NA	NA	NA	NA	NA
ILMN_1758806	C21orf2	NA	NA		0,006834814	NA	NA	NA	NA	NA	NA	NA
ILMN_2182647	PINX1	NA	NA		0,006857856	NA	NA	NA	NA	NA	NA	NA
ILMN_1788099	LSM4	0,00362476	NA		0,006900898	NA	NA	NA	NA	NA	NA	NA
ILMN_1782504	MTERFD1	NA	NA		0,006978871	NA	NA	NA	NA	NA	NA	NA
ILMN_2363106	RBM23	NA	NA		0,0069846	NA	NA	NA	NA	NA	NA	NA
ILMN_1761858	MID1	NA	NA		0,006991827	NA	NA	NA	NA	NA	NA	NA
ILMN_2380163	PTPRF	NA	NA		0,007027594	NA	NA	NA	NA	NA	NA	NA
ILMN_3301740	LOC729887	0,003883969	NA		0,007090214	NA	NA	NA	NA	NA	NA	NA
ILMN_1791375	STAG3L2	NA	NA		0,00710795	NA	NA	NA	NA	NA	NA	NA
ILMN_1771599	PLOD2	NA	NA		0,007210923	NA	NA	NA	NA	NA	NA	NA
ILMN_1654851	FAM134A	0,000659666	NA		0,007252783	NA	NA	NA	NA	NA	NA	NA
ILMN_2059535	PPM1F	0,00024729	NA		0,007281034	NA	NA	NA	NA	NA	NA	NA
ILMN_1687084	C3orf64	NA	NA		0,007314047	NA	NA	NA	NA	NA	NA	NA
ILMN_1761804	ALDH9A1	NA	NA		0,007334245	NA	NA	NA	NA	NA	NA	NA
ILMN_1778890	PPIL5	NA	NA		0,007336994	NA	NA	NA	NA	NA	NA	NA
ILMN_3265143	LOC1001295	NA	NA		0,007349375	NA	NA	NA	NA	NA	NA	NA
ILMN_1725528	LOC400657	NA	NA		0,007359189	NA	NA	NA	NA	NA	NA	NA
ILMN_1803277	MVP	NA	NA		0,007443457	NA	NA	NA	NA	NA	NA	NA
ILMN_1716445	LOC727761	0,009161243	NA		0,007459081	NA	NA	NA	NA	NA	NA	NA
ILMN_1775901	PHF5A	NA	NA		0,007463187	NA	NA	NA	NA	NA	NA	NA
ILMN_1709164	RUNDC2C	0,008963589	NA		0,007471188	NA	NA	NA	NA	NA	NA	NA
ILMN_2225548	ZNF521	0,001539598	NA		0,007491798	NA	NA	NA	NA	NA	NA	NA
ILMN_2073732	CBLL1	NA	NA		0,007493906	NA	NA	NA	NA	NA	NA	NA
ILMN_1727540	C1orf112	NA	NA		0,007500938	NA	NA	NA	NA	NA	NA	NA
ILMN_3272500	IFI27L1	NA	NA		0,007501323	NA	NA	NA	NA	NA	NA	NA
ILMN_1657993	ADNP	0,002970988	NA		0,007507578	NA	NA	NA	NA	NA	NA	NA
ILMN_2104106	XPR1	NA	NA		0,007535643	NA	NA	NA	NA	NA	NA	NA
ILMN_1662686	USPL1	NA	NA		0,007543718	NA	NA	NA	NA	NA	NA	NA
ILMN_2116556	LSM5	NA	NA		0,00754881	NA	NA	NA	NA	NA	NA	NA
ILMN_1721713	EXOSC9	NA	NA		0,007662203	NA	NA	NA	NA	NA	NA	NA
ILMN_2410924	PLOD2	NA	NA		0,007679898	NA	NA	NA	NA	NA	NA	NA
ILMN_1754489	FBXL20	NA	NA		0,007692553	NA	NA	NA	NA	NA	NA	NA
ILMN_3310840	MIR21	NA	NA		0,007723441	NA	NA	NA	NA	NA	NA	NA
ILMN_1720708	CSNK1D	NA	NA		0,00772394	NA	NA	NA	NA	NA	NA	NA
ILMN_1726755	COPS4	NA	NA		0,0077261	NA	NA	NA	NA	NA	NA	NA
ILMN_1706344	ABHD6	NA	NA		0,007812419	NA	NA	NA	NA	NA	NA	NA
ILMN_3235637	LOC1001282	NA	NA		0,007825734	NA	NA	NA	NA	NA	NA	NA
ILMN_1746658	RORB	NA	NA		0,007831343	NA	NA	NA	NA	NA	NA	NA
ILMN_1692707	C2orf79	1,42E-05	NA		0,007843767	NA	NA	NA	NA	NA	NA	NA
ILMN_1672366	PIGY	NA	NA		0,007893467	NA	NA	NA	NA	NA	NA	NA
ILMN_1730504	AGPAT4	NA	NA		0,007905476	NA	NA	NA	NA	NA	NA	NA
ILMN_1797604	CAP1	NA	NA		0,007909606	NA	NA	NA	NA	NA	NA	NA
ILMN_1718932	MTRR	NA	NA		0,007928073	NA	NA	NA	NA	NA	NA	NA
ILMN_1730572	HNRPDL	NA	NA		0,007970691	NA	NA	NA	NA	NA	NA	NA
ILMN_1662618	SQSTM1	NA	NA		0,007977004	NA	NA	NA	NA	NA	NA	NA
ILMN_3210171	LOC389156	NA	NA		0,007996889	NA	NA	NA	NA	NA	NA	NA
ILMN_1766247	ZBTB2	0,000493181	NA		0,008036265	NA	NA	NA	NA	NA	NA	NA
ILMN_1740587	RPS11	NA	NA		0,008053393	NA	NA	NA	NA	NA	NA	NA
ILMN_2041368	RN7SL1	NA	NA		0,008084191	NA	NA	NA	NA	NA	NA	NA
ILMN_1693836	LOC653344	0,000230111	NA		0,008124632	NA	NA	NA	NA	NA	NA	NA
ILMN_1656904	SLC1A4	NA	NA		0,008163322	NA	NA	NA	NA	NA	NA	NA
ILMN_1744240	WDR67	NA	NA		0,008195533	NA	NA	NA	NA	NA	NA	NA
ILMN_1749327	MAPK13	NA	NA		0,008225905	NA	NA	NA	NA	NA	NA	NA
ILMN_1651506	NCOA6IP	NA	NA		0,008258676	NA	NA	NA	NA	NA	NA	NA
ILMN_3249244	TMEM106A	NA	NA		0,008314155	NA	NA	NA	NA	NA	NA	NA
ILMN_1798380	UBQLN1	NA	NA		0,008383691	NA	NA	NA	NA	NA	NA	NA
ILMN_3182762	LOC1001296	NA	NA		0,008472574	NA	NA	NA	NA	NA	NA	NA
ILMN_2384513	C2CD2	NA	NA		0,00848909	NA	NA	NA	NA	NA	NA	NA
ILMN_2101526	GGCT	NA	NA		0,008489251	NA	NA	NA	NA	NA	NA	NA
ILMN_2140559	IRX5	0,0078715	NA		0,008489503	NA	NA	NA	NA	NA	NA	NA
ILMN_1746257	DAZAP1	0,004161038		0,009226229	0,008489815	NA	NA	NA	NA	NA	NA	NA
ILMN_2048326	RPS27A	NA	NA		0,008494196	NA	NA	NA	NA	NA	NA	NA
ILMN_2321931	DCLRE1C	NA	NA		0,008528449	NA	NA	NA	NA	NA	NA	NA
ILMN_3290100	LOC645157	NA	NA		0,008533258	NA	NA	NA	NA	NA	NA	NA
ILMN_1659058	PPP1R10	NA	NA		0,008560453	NA	NA	NA	NA	NA	NA	NA
ILMN_2218450	LSM1	0,009610295	NA		0,008619788	NA	NA	NA	NA	NA	NA	NA
ILMN_3249578	LOC1001323	NA	NA		0,008654398	NA	NA	NA	NA	NA	NA	NA
ILMN_1794392	DDX3X	0,009747558	NA		0,008660029	NA	NA	NA	NA	NA	NA	NA
ILMN_2336094	ODZ3	NA	NA		0,008669745	NA	NA	NA	NA	NA	NA	NA
ILMN_2333107	AES	NA	NA		0,008674257	NA	NA	NA	NA	NA	NA	NA
ILMN_2402805	TRPC4AP	0,001071122	NA		0,00873719	NA	NA	NA	NA	NA	NA	NA
ILMN_1748018	GORASP2	NA	NA		0,008851987	NA	NA	NA	NA	NA	NA	NA
ILMN_1784822	PPP1R3F	NA	NA		0,008859478	NA	NA	NA	NA	NA	NA	NA
ILMN_3183544	LOC1001301	NA	NA		0,00887896	NA	NA	NA	NA	NA	NA	NA
ILMN_3295419	LOC644363	NA	NA		0,008888238	NA	NA	NA	NA	NA	NA	NA
ILMN_1804945	ZNF667	NA	NA		0,008962399	NA	NA	NA	NA	NA	NA	NA
ILMN_1708778	ASS1	NA	NA		0,008973436	NA	NA	NA	NA	NA	NA	NA
ILMN_1741003	ANXA5	NA	NA		0,008976259	NA	NA	NA	NA	NA	NA	NA
ILMN_2316236	HOPX	NA	NA		0,008992012	NA	NA	NA	NA	NA	NA	NA

ILMN_1791792	C12orf5	NA	NA	0,009024949	NA	NA	NA	NA	NA	NA	NA
ILMN_1791726	TUBB3	NA	NA	0,009045867	NA	NA	NA	NA	NA	NA	NA
ILMN_1741422	FUT8	NA	NA	0,009058845	NA	NA	NA	NA	NA	NA	NA
ILMN_1748291	C1orf55	NA	NA	0,009123514	NA	NA	NA	NA	NA	NA	NA
ILMN_1660698	GTPBP8	NA	NA	0,009164726	NA	NA	NA	NA	NA	NA	NA
ILMN_1692844	TBC1D19	NA	NA	0,009182247	NA	NA	NA	NA	NA	NA	NA
ILMN_1716524	RAB7A	NA	NA	0,009224909	NA	NA	NA	NA	NA	NA	NA
ILMN_1724406	INO80E	0,000395012	NA	0,009237446	NA	NA	NA	NA	NA	NA	NA
ILMN_1702636	TUBB6	NA	NA	0,009415263	NA	NA	NA	NA	NA	NA	NA
ILMN_1782611	LOC643870	NA	NA	0,009418429	NA	NA	NA	NA	NA	NA	NA
ILMN_1653712	UAP1L1	NA	NA	0,00944591	NA	NA	NA	NA	NA	NA	NA
ILMN_1659273	LOC441408	NA	NA	0,009447969	NA	NA	NA	NA	NA	NA	NA
ILMN_1800659	PGM1	NA	NA	0,009466075	NA	NA	NA	NA	NA	NA	NA
ILMN_2222984	RDH14	NA	NA	0,009467526	NA	NA	NA	NA	NA	NA	NA
ILMN_3206390	LOC727808	NA	NA	0,009475969	NA	NA	NA	NA	NA	NA	NA
ILMN_1691395	ZBTB11	NA	NA	0,009529845	NA	NA	NA	NA	NA	NA	NA
ILMN_3256742	LOC1001299	0,001241138	NA	0,009540202	NA	NA	NA	NA	NA	NA	NA
ILMN_2346573	PSME3	0,006698272	NA	0,009553507	NA	NA	NA	NA	NA	NA	NA
ILMN_1809931	NDRG1	NA	NA	0,009554413	NA	NA	NA	NA	NA	NA	NA
ILMN_1655613	GSTA2	NA	NA	0,009558244	NA	NA	NA	NA	NA	NA	NA
ILMN_2372639	TRAPPC5	0,00534669	NA	0,009561518	NA	NA	NA	NA	NA	NA	NA
ILMN_1720270	CDR2	0,000900497	NA	0,009570299	NA	NA	NA	NA	NA	NA	NA
ILMN_1700168	LARS2	0,000767747	NA	0,009581451	NA	NA	NA	NA	NA	NA	NA
ILMN_3213531	LOC653737	NA	NA	0,009592451	NA	NA	NA	NA	NA	NA	NA
ILMN_1700044	SAP130	NA	NA	0,009637227	NA	NA	NA	NA	NA	NA	NA
ILMN_2090558	C2orf25	NA	NA	0,009665831	NA	NA	NA	NA	NA	NA	NA
ILMN_3251634	SENP5	NA	NA	0,009673045	NA	NA	NA	NA	NA	NA	NA
ILMN_1732725	SAPS3	NA	NA	0,009761676	NA	NA	NA	NA	NA	NA	NA
ILMN_3251565	RNASEH2C	NA	NA	0,009828457	NA	NA	NA	NA	NA	NA	NA
ILMN_1679195	C20orf24	0,004357302	NA	0,009844799	NA	NA	NA	NA	NA	NA	NA
ILMN_1765043	RPL38	0,007492746	NA	0,009960781	NA	NA	NA	NA	NA	NA	NA
ILMN_2214997	LRRFIP1	NA	NA	0,009989247	NA	NA	NA	NA	NA	NA	NA
ILMN_1691578	GTF3C6	NA	2,65E-05	NA	NA	NA	NA	NA	NA	NA	NA
ILMN_1781966	OSBP2	NA	2,76E-05	NA	NA	NA	NA	NA	NA	NA	NA
ILMN_1684873	ARSD	0,004683747	6,69E-05	NA	NA	NA	NA	NA	NA	NA	NA
ILMN_1686920	CCDC58	NA	9,43E-05	NA	NA	NA	NA	NA	NA	NA	NA
ILMN_1723743	ROM1	NA	0,000142925	NA	NA	NA	NA	NA	NA	NA	NA
ILMN_1654068	NUDT1	NA	0,000306228	NA	NA	NA	NA	NA	NA	NA	NA
ILMN_1759766	CTXN1	NA	0,000337217	NA	NA	NA	NA	NA	NA	NA	NA
ILMN_1697639	OGT	NA	0,000349281	NA	NA	NA	NA	NA	NA	NA	NA
ILMN_2094061	IMPA2	NA	0,000358397	NA	NA	NA	NA	NA	NA	NA	NA
ILMN_1776586	RPL26L1	NA	0,000358818	NA	NA	NA	NA	NA	NA	NA	NA
ILMN_1792885	CTSC	NA	0,00047083	NA	NA	NA	NA	NA	NA	NA	NA
ILMN_1768510	MAN2B2	NA	0,000492132	NA	NA	NA	NA	NA	NA	NA	NA
ILMN_1757730	TTC27	NA	0,000505788	NA	NA	NA	NA	NA	NA	NA	NA
ILMN_1667510	C12orf65	NA	0,000522468	NA	NA	NA	NA	NA	NA	NA	NA
ILMN_1696003	GNAI3	NA	0,000630879	NA	NA	NA	NA	NA	NA	NA	NA
ILMN_3253456	FNDC3B	NA	0,000703346	NA	NA	NA	NA	NA	NA	NA	NA
ILMN_2192281	CARD8	NA	0,000829781	NA	NA	NA	NA	NA	NA	NA	NA
ILMN_1694027	SESN3	NA	0,000838385	NA	NA	NA	NA	NA	NA	NA	NA
ILMN_1665428	GSDMD	NA	0,000840692	NA	NA	NA	NA	NA	NA	NA	NA
ILMN_1743646	VASP	NA	0,00084847	NA	NA	NA	NA	NA	NA	NA	NA
ILMN_1795949	CORO7	NA	0,00088772	NA	NA	NA	NA	NA	NA	NA	NA
ILMN_2041327	MRPL37	NA	0,000887969	NA	NA	NA	NA	NA	NA	NA	NA
ILMN_1811696	FDXR	NA	0,000958379	NA	NA	NA	NA	NA	NA	NA	NA
ILMN_1675542	LOC729148	NA	0,00117864	NA	NA	NA	NA	NA	NA	NA	NA
ILMN_1715113	HARS2	NA	0,001257473	NA	NA	NA	NA	NA	NA	NA	NA
ILMN_1735438	GPM6B	NA	0,001257628	NA	NA	NA	NA	NA	NA	NA	NA
ILMN_1683279	PEX6	NA	0,00126178	NA	NA	NA	NA	NA	NA	NA	NA
ILMN_2150019	SUCLA2	NA	0,001379869	NA	NA	NA	NA	NA	NA	NA	NA
ILMN_1735151	EIF5A2	NA	0,001521239	NA	NA	NA	NA	NA	NA	NA	NA
ILMN_1714896	SART3	NA	0,001547592	NA	NA	NA	NA	NA	NA	NA	NA
ILMN_1714296	DNAH2	NA	0,001588241	NA	NA	NA	NA	NA	NA	NA	NA
ILMN_1791147	YPEL3	NA	0,001623243	NA	NA	NA	NA	NA	NA	NA	NA
ILMN_1815134	PI4K2B	0,000175015	0,001666795	NA	NA	NA	NA	NA	NA	NA	NA
ILMN_1678934	POLR1E	NA	0,001700746	NA	NA	NA	NA	NA	NA	NA	NA
ILMN_1798181	IRF7	NA	0,001745154	NA	NA	NA	NA	NA	NA	NA	NA
ILMN_1674633	UBE2H	NA	0,00174556	NA	NA	NA	NA	NA	NA	NA	NA
ILMN_2221006	RAD21	NA	0,001751175	NA	NA	NA	NA	NA	NA	NA	NA
ILMN_1850238		NA	0,001782651	NA	NA	NA	NA	NA	NA	NA	NA
ILMN_1736103	ITPR2	NA	0,001860956	NA	NA	NA	NA	NA	NA	NA	NA
ILMN_1750693	SSR1	NA	0,001867905	NA	NA	NA	NA	NA	NA	NA	NA
ILMN_1707336	ARPC4	NA	0,001996657	NA	NA	NA	NA	NA	NA	NA	NA
ILMN_1788149	NEK11	NA	0,002091577	NA	NA	NA	NA	NA	NA	NA	NA
ILMN_1685170	ANXA11	NA	0,002188811	NA	NA	NA	NA	NA	NA	NA	NA
ILMN_1680314	TXN	NA	0,002297759	NA	NA	NA	NA	NA	NA	NA	NA
ILMN_1684205	CIB1	NA	0,002391067	NA	NA	NA	NA	NA	NA	NA	NA
ILMN_1675788	ZNF175	0,005610972	0,002462744	NA	NA	NA	NA	NA	NA	NA	NA
ILMN_1801246	IFITM1	NA	0,002556452	NA	NA	NA	NA	NA	NA	NA	NA
ILMN_1745374	IFI35	NA	0,002590354	NA	NA	NA	NA	NA	NA	NA	NA
ILMN_1666924	PINK1	NA	0,002691355	NA	NA	NA	NA	NA	NA	NA	NA
ILMN_2138435	MRPS27	NA	0,002706972	NA	NA	NA	NA	NA	NA	NA	NA
ILMN_1769299	MTMR11	NA	0,002777022	NA	NA	NA	NA	NA	NA	NA	NA
ILMN_1671005	IRF2BP2	NA	0,002803763	NA	NA	NA	NA	NA	NA	NA	NA
ILMN_2396982	BCL2L12	NA	0,00289187	NA	NA	NA	NA	NA	NA	NA	NA
ILMN_1810844	RARRES2	NA	0,002896257	NA	NA	NA	NA	NA	NA	NA	NA
ILMN_1769751	PIGG	NA	0,002934051	NA	NA	NA	NA	NA	NA	NA	NA
ILMN_1691864	Sep 10	NA	0,003084912	NA	NA	NA	NA	NA	NA	NA	NA
ILMN_1679917	ARL16	NA	0,003113359	NA	NA	NA	NA	NA	NA	NA	NA
ILMN_1811426	TMTC1	NA	0,003176359	NA	NA	NA	NA	NA	NA	NA	NA
ILMN_1774261	DOK4	NA	0,003198613	NA	NA	NA	NA	NA	NA	NA	NA
ILMN_1750549	PI4K2A	0,003869517	0,003219228	NA	NA	NA	NA	NA	NA	NA	NA
ILMN_1796177	GIPC1	NA	0,003230994	NA	NA	NA	NA	NA	NA	NA	NA
ILMN_2307744	CD46	0,004681494	0,003319382	NA	NA	NA	NA	NA	NA	NA	NA
ILMN_1730303	KBTBD7	NA	0,003331874	NA	NA	NA	NA	NA	NA	NA	NA
ILMN_3233388	RELL1	NA	0,003518782	NA	NA	NA	NA	NA	NA	NA	NA
ILMN_1750178	LOC653450	NA	0,003825671	NA	NA	NA	NA	NA	NA	NA	NA
ILMN_1668605	NAAA	NA	0,003885843	NA	NA	NA	NA	NA	NA	NA	NA
ILMN_1696347	CTSC	NA	0,003980373	NA	NA	NA	NA	NA	NA	NA	NA
ILMN_1737972	TSPYL5	NA	0,004021626	NA	NA	NA	NA	NA	NA	NA	NA
ILMN_1796749	KIF1C	NA	0,004027696	NA	NA	NA	NA	NA	NA	NA	NA
ILMN_1736048	ELL	0,00454263	0,004074608	NA	NA	NA	NA	NA	NA	NA	NA
ILMN_1661599	DDIT4	NA	0,004099447	NA	NA	NA	NA	NA	NA	NA	NA
ILMN_1689431	APCDD1L	NA	0,004139944	NA	NA	NA	NA	NA	NA	NA	NA
ILMN_1796397	CISD2	NA	0,004168231	NA	NA	NA	NA	NA	NA	NA	NA
ILMN_3250268	LOC643911	0,000285893	0,004181824	NA	NA	NA	NA	NA	NA	NA	NA
ILMN_1798619	KCTD17	NA	0,004276679	NA	NA	NA	NA	NA	NA	NA	NA
ILMN_2172202	NUDT15	NA	0,004312828	NA	NA	NA	NA	NA	NA	NA	NA
ILMN_1807283	NDST1	NA	0,00456102	NA	NA	NA	NA	NA	NA	NA	NA

ILMN_1667476	LTBR	NA	0,004569547	NA	NA	NA	NA	NA	NA	NA	NA
ILMN_3289745	LOC339352	0,004105502	0,004701635	NA	NA	NA	NA	NA	NA	NA	NA
ILMN_1761068	MGC52000	NA	0,004744751	NA	NA	NA	NA	NA	NA	NA	NA
ILMN_1708858	CSNK1E	NA	0,004809251	NA	NA	NA	NA	NA	NA	NA	NA
ILMN_1796113	BCDIN3D	NA	0,004902364	NA	NA	NA	NA	NA	NA	NA	NA
ILMN_1684042	BET1	NA	0,004906853	NA	NA	NA	NA	NA	NA	NA	NA
ILMN_1662049	AGPAT5	NA	0,004922886	NA	NA	NA	NA	NA	NA	NA	NA
ILMN_1754553	MED19	NA	0,005041351	NA	NA	NA	NA	NA	NA	NA	NA
ILMN_1716057	CAPN2	NA	0,005112415	NA	NA	NA	NA	NA	NA	NA	NA
ILMN_1691143	RAB18	NA	0,005114844	NA	NA	NA	NA	NA	NA	NA	NA
ILMN_1781987	CDK5	NA	0,00513167	NA	NA	NA	NA	NA	NA	NA	NA
ILMN_1715661	TFAM	NA	0,005247039	NA	NA	NA	NA	NA	NA	NA	NA
ILMN_1764609	PWWP2B	NA	0,005277521	NA	NA	NA	NA	NA	NA	NA	NA
ILMN_2351241	RNF14	0,000201149	0,005307325	NA	NA	NA	NA	NA	NA	NA	NA
ILMN_1732176	AGPAT2	NA	0,005328758	NA	NA	NA	NA	NA	NA	NA	NA
ILMN_1820226		NA	0,005572367	NA	NA	NA	NA	NA	NA	NA	NA
ILMN_1796180	CRY2	NA	0,005613538	NA	NA	NA	NA	NA	NA	NA	NA
ILMN_2317923	TMEM132A	NA	0,005709385	NA	NA	NA	NA	NA	NA	NA	NA
ILMN_1682930	SIPA1	NA	0,005784613	NA	NA	NA	NA	NA	NA	NA	NA
ILMN_1660727	ENPP5	0,000319085	0,005909204	NA	NA	NA	NA	NA	NA	NA	NA
ILMN_1705407	NOP56	NA	0,005960292	NA	NA	NA	NA	NA	NA	NA	NA
ILMN_3238623	LYRM7	NA	0,005963462	NA	NA	NA	NA	NA	NA	NA	NA
ILMN_1698001	NCK1	NA	0,005973329	NA	NA	NA	NA	NA	NA	NA	NA
ILMN_3203444	LOC1001325	NA	0,006114412	NA	NA	NA	NA	NA	NA	NA	NA
ILMN_1812552	PHCA	NA	0,006123139	NA	NA	NA	NA	NA	NA	NA	NA
ILMN_2134974	RAB38	NA	0,006273193	NA	NA	NA	NA	NA	NA	NA	NA
ILMN_1760400	C8orf41	NA	0,006353346	NA	NA	NA	NA	NA	NA	NA	NA
ILMN_2088172	POLR2B	NA	0,006397855	NA	NA	NA	NA	NA	NA	NA	NA
ILMN_2106167	RAP1GDS1	NA	0,006473316	NA	NA	NA	NA	NA	NA	NA	NA
ILMN_1764596	MPST	NA	0,00653349	NA	NA	NA	NA	NA	NA	NA	NA
ILMN_2139396	IGDCC4	NA	0,006542065	NA	NA	NA	NA	NA	NA	NA	NA
ILMN_1812262	DDR1	NA	0,006700069	NA	NA	NA	NA	NA	NA	NA	NA
ILMN_2394498	SYF2	NA	0,006808698	NA	NA	NA	NA	NA	NA	NA	NA
ILMN_2323048	ERP29	NA	0,006812488	NA	NA	NA	NA	NA	NA	NA	NA
ILMN_1668312	SLC2A9	NA	0,006886803	NA	NA	NA	NA	NA	NA	NA	NA
ILMN_1716382	LOC387882	NA	0,006937665	NA	NA	NA	NA	NA	NA	NA	NA
ILMN_1714417	PSEN2	NA	0,00710717	NA	NA	NA	NA	NA	NA	NA	NA
ILMN_1690099	ITGB1BP1	NA	0,007324095	NA	NA	NA	NA	NA	NA	NA	NA
ILMN_1723536	USP33	NA	0,007492981	NA	NA	NA	NA	NA	NA	NA	NA
ILMN_1792078	RNF114	NA	0,007744053	NA	NA	NA	NA	NA	NA	NA	NA
ILMN_1739154	LSAMP	NA	0,007905588	NA	NA	NA	NA	NA	NA	NA	NA
ILMN_1666122	HEG1	NA	0,007924263	NA	NA	NA	NA	NA	NA	NA	NA
ILMN_1800096	MPST	NA	0,007936716	NA	NA	NA	NA	NA	NA	NA	NA
ILMN_3238859	FAM120AOS	NA	0,007940514	NA	NA	NA	NA	NA	NA	NA	NA
ILMN_1796710	PHC1	NA	0,007953338	NA	NA	NA	NA	NA	NA	NA	NA
ILMN_1785284	ALDH6A1	NA	0,008034739	NA	NA	NA	NA	NA	NA	NA	NA
ILMN_2058782	IFI27	NA	0,008070358	NA	NA	NA	NA	NA	NA	NA	NA
ILMN_3201088	LOC644620	NA	0,008189397	NA	NA	NA	NA	NA	NA	NA	NA
ILMN_2318733	BNIP1	NA	0,008232452	NA	NA	NA	NA	NA	NA	NA	NA
ILMN_1680774	LOC730994	NA	0,008421724	NA	NA	NA	NA	NA	NA	NA	NA
ILMN_1792837	CIAO1	NA	0,008428103	NA	NA	NA	NA	NA	NA	NA	NA
ILMN_2096654	COMMD4	NA	0,008441917	NA	NA	NA	NA	NA	NA	NA	NA
ILMN_1779182	TMEM98	NA	0,008476237	NA	NA	NA	NA	NA	NA	NA	NA
ILMN_3270972	ASAP2	NA	0,008477378	NA	NA	NA	NA	NA	NA	NA	NA
ILMN_1718988	DAZAP2	NA	0,008567503	NA	NA	NA	NA	NA	NA	NA	NA
ILMN_2362982	ARID4B	NA	0,008577741	NA	NA	NA	NA	NA	NA	NA	NA
ILMN_1684461	CADPS2	NA	0,008616998	NA	NA	NA	NA	NA	NA	NA	NA
ILMN_1688625	AIM1	NA	0,008631526	NA	NA	NA	NA	NA	NA	NA	NA
ILMN_1657977	MSRB2	0,000531636	0,008666566	NA	NA	NA	NA	NA	NA	NA	NA
ILMN_1669497	OSBPL10	NA	0,008850834	NA	NA	NA	NA	NA	NA	NA	NA
ILMN_1738885	WDR40B	NA	0,008890872	NA	NA	NA	NA	NA	NA	NA	NA
ILMN_1758214	RARS2	NA	0,008915244	NA	NA	NA	NA	NA	NA	NA	NA
ILMN_1725612	NUP50	NA	0,008951882	NA	NA	NA	NA	NA	NA	NA	NA
ILMN_1671191	UQCRC1	NA	0,008969451	NA	NA	NA	NA	NA	NA	NA	NA
ILMN_1732089	MRI1	NA	0,00901455	NA	NA	NA	NA	NA	NA	NA	NA
ILMN_1659462	DUSP23	NA	0,009028355	NA	NA	NA	NA	NA	NA	NA	NA
ILMN_3240222	PRAGMIN	NA	0,009062887	NA	NA	NA	NA	NA	NA	NA	NA
ILMN_1678086	CCDC74A	NA	0,009142005	NA	NA	NA	NA	NA	NA	NA	NA
ILMN_1708482	TMEM80	NA	0,009162997	NA	NA	NA	NA	NA	NA	NA	NA
ILMN_1730516	TMEM133	NA	0,009174872	NA	NA	NA	NA	NA	NA	NA	NA
ILMN_1764383	MCOLN1	NA	0,009308609	NA	NA	NA	NA	NA	NA	NA	NA
ILMN_1711799	C9orf40	NA	0,009333056	NA	NA	NA	NA	NA	NA	NA	NA
ILMN_1699856	RALGDS	NA	0,009577373	NA	NA	NA	NA	NA	NA	NA	NA
ILMN_1681679	TSPO	NA	0,00963633	NA	NA	NA	NA	NA	NA	NA	NA
ILMN_1863939		NA	0,009671006	NA	NA	NA	NA	NA	NA	NA	NA
ILMN_1672834	SSH2	NA	0,009689592	NA	NA	NA	NA	NA	NA	NA	NA
ILMN_3300313	P4HTM	NA	0,009847975	NA	NA	NA	NA	NA	NA	NA	NA
ILMN_1800626	SESN1	7,81E-06	NA	NA	NA	NA	NA	NA	NA	NA	NA
ILMN_1696701	LOC344595	2,17E-05	NA	NA	NA	NA	NA	NA	NA	NA	NA
ILMN_1721727	USP6	4,01E-05	NA	NA	NA	NA	NA	NA	NA	NA	NA
ILMN_1767360	IL10RB	4,56E-05	NA	NA	NA	NA	NA	NA	NA	NA	NA
ILMN_3241262	PABPC4L	4,87E-05	NA	NA	NA	NA	NA	NA	NA	NA	NA
ILMN_1696975	USP1	6,18E-05	NA	NA	NA	NA	NA	NA	NA	NA	NA
ILMN_1778013	PDPR	7,22E-05	NA	NA	NA	NA	NA	NA	NA	NA	NA
ILMN_1683023	PDGFC	7,49E-05	NA	NA	NA	NA	NA	NA	NA	NA	NA
ILMN_1723846	FAM119B	7,50E-05	NA	NA	NA	NA	NA	NA	NA	NA	NA
ILMN_1674367	SPRR2F	8,75E-05	NA	NA	NA	NA	NA	NA	NA	NA	NA
ILMN_1794740	CD151	0,000110369	NA	NA	NA	NA	NA	NA	NA	NA	NA
ILMN_2229379	KIT	0,000119855	NA	NA	NA	NA	NA	NA	NA	NA	NA
ILMN_2176037	GNA13	0,000120227	NA	NA	NA	NA	NA	NA	NA	NA	NA
ILMN_2257665	PARL	0,000121321	NA	NA	NA	NA	NA	NA	NA	NA	NA
ILMN_1781761	ENPP4	0,000123622	NA	NA	NA	NA	NA	NA	NA	NA	NA
ILMN_1695959	C21orf63	0,000147761	NA	NA	NA	NA	NA	NA	NA	NA	NA
ILMN_3176040	LOC1001304	0,00014848	NA	NA	NA	NA	NA	NA	NA	NA	NA
ILMN_2117904	ZNF22	0,000197919	NA	NA	NA	NA	NA	NA	NA	NA	NA
ILMN_1709101	AKAP13	0,000213696	NA	NA	NA	NA	NA	NA	NA	NA	NA
ILMN_1791728	SLC25A25	0,000250818	NA	NA	NA	NA	NA	NA	NA	NA	NA
ILMN_1683562	SNRPG	0,00025218	NA	NA	NA	NA	NA	NA	NA	NA	NA
ILMN_1736757	GNPTAB	0,000255412	NA	NA	NA	NA	NA	NA	NA	NA	NA
ILMN_1801403	DCUN1D4	0,000260913	NA	NA	NA	NA	NA	NA	NA	NA	NA
ILMN_3247261	RAPGEF2	0,000262046	NA	NA	NA	NA	NA	NA	NA	NA	NA
ILMN_3244929	LOC1001331	0,000274268	NA	NA	NA	NA	NA	NA	NA	NA	NA
ILMN_1713086	RPL27A	0,000318105	NA	NA	NA	NA	NA	NA	NA	NA	NA
ILMN_1779979	SLC37A3	0,000357968	NA	NA	NA	NA	NA	NA	NA	NA	NA
ILMN_1706426	DSTN	0,00036207	NA	NA	NA	NA	NA	NA	NA	NA	NA
ILMN_1751615	COQ10B	0,000371838	NA	NA	NA	NA	NA	NA	NA	NA	NA
ILMN_1686954	RPS10	0,000372751	NA	NA	NA	NA	NA	NA	NA	NA	NA
ILMN_2367233	ZNF654	0,000372867	NA	NA	NA	NA	NA	NA	NA	NA	NA
ILMN_1806415	TTLL1	0,000378267	NA	NA	NA	NA	NA	NA	NA	NA	NA

ILMN_1802257	PCTP	0,000386701	NA	NA	NA	NA	NA	NA	NA	NA	NA
ILMN_1658684	ATP9B	0,000401755	NA	NA	NA	NA	NA	NA	NA	NA	NA
ILMN_2207533	RPS17	0,000413649	NA	NA	NA	NA	NA	NA	NA	NA	NA
ILMN_1693045	TMED1	0,000417677	NA	NA	NA	NA	NA	NA	NA	NA	NA
ILMN_2390821	THOC2	0,000429756	NA	NA	NA	NA	NA	NA	NA	NA	NA
ILMN_2123871	TMEM18	0,000429822	NA	NA	NA	NA	NA	NA	NA	NA	NA
ILMN_1778237	FN1	0,000437746	NA	NA	NA	NA	NA	NA	NA	NA	NA
ILMN_1729973	ZC3HAV1	0,000461843	NA	NA	NA	NA	NA	NA	NA	NA	NA
ILMN_1708427	KPNA3	0,000469346	NA	NA	NA	NA	NA	NA	NA	NA	NA
ILMN_1695868	PRICKLE4	0,000494584	NA	NA	NA	NA	NA	NA	NA	NA	NA
ILMN_1806432	NT5C	0,000499552	NA	NA	NA	NA	NA	NA	NA	NA	NA
ILMN_2180677	ARMCX1	0,000519519	NA	NA	NA	NA	NA	NA	NA	NA	NA
ILMN_1734153	GDI1	0,000548221	NA	NA	NA	NA	NA	NA	NA	NA	NA
ILMN_3224952	LOC729903	0,000561066	NA	NA	NA	NA	NA	NA	NA	NA	NA
ILMN_1810376	C11orf87	0,000569095	NA	NA	NA	NA	NA	NA	NA	NA	NA
ILMN_1763842	PTRH1	0,000577979	NA	NA	NA	NA	NA	NA	NA	NA	NA
ILMN_1796407	SS18L2	0,000618929	NA	NA	NA	NA	NA	NA	NA	NA	NA
ILMN_2410986	STAT3	0,000632641	NA	NA	NA	NA	NA	NA	NA	NA	NA
ILMN_2376108	PSMB9	0,000639744	NA	NA	NA	NA	NA	NA	NA	NA	NA
ILMN_1682197	NFXL1	0,000647758	NA	NA	NA	NA	NA	NA	NA	NA	NA
ILMN_1775943	BRMS1L	0,000663701	NA	NA	NA	NA	NA	NA	NA	NA	NA
ILMN_3293676	LOC1001313	0,000669794	NA	NA	NA	NA	NA	NA	NA	NA	NA
ILMN_1751431	WIBG	0,000693402	NA	NA	NA	NA	NA	NA	NA	NA	NA
ILMN_1766245	SUPT4H1	0,000700252	NA	NA	NA	NA	NA	NA	NA	NA	NA
ILMN_1734096	DCLRE1A	0,00070586	NA	NA	NA	NA	NA	NA	NA	NA	NA
ILMN_1792435	STAG1	0,000712219	NA	NA	NA	NA	NA	NA	NA	NA	NA
ILMN_3235113	TOMM6	0,000751893	NA	NA	NA	NA	NA	NA	NA	NA	NA
ILMN_1712751	HADHA	0,000752953	NA	NA	NA	NA	NA	NA	NA	NA	NA
ILMN_1774823	RPL34	0,000761785	NA	NA	NA	NA	NA	NA	NA	NA	NA
ILMN_3243982	SNAP47	0,000768036	NA	NA	NA	NA	NA	NA	NA	NA	NA
ILMN_1691425	LDOC1L	0,000768776	NA	NA	NA	NA	NA	NA	NA	NA	NA
ILMN_1702946	THUMPD1	0,000797925	NA	NA	NA	NA	NA	NA	NA	NA	NA
ILMN_2101810	ARHGAP12	0,000806177	NA	NA	NA	NA	NA	NA	NA	NA	NA
ILMN_1740571	LOC647859	0,000821999	NA	NA	NA	NA	NA	NA	NA	NA	NA
ILMN_1777106	LRRC57	0,000825902	NA	NA	NA	NA	NA	NA	NA	NA	NA
ILMN_1736901	ZDHHC23	0,000840592	NA	NA	NA	NA	NA	NA	NA	NA	NA
ILMN_1673478	C5orf5	0,000842402	NA	NA	NA	NA	NA	NA	NA	NA	NA
ILMN_2369826	C16orf13	0,000865153	NA	NA	NA	NA	NA	NA	NA	NA	NA
ILMN_1803772	POLD4	0,000868093	NA	NA	NA	NA	NA	NA	NA	NA	NA
ILMN_2389590	PRKAR1A	0,000872331	NA	NA	NA	NA	NA	NA	NA	NA	NA
ILMN_1814305	SAMD9	0,000882594	NA	NA	NA	NA	NA	NA	NA	NA	NA
ILMN_1661945	C14orf156	0,000886007	NA	NA	NA	NA	NA	NA	NA	NA	NA
ILMN_1760414	AADAC	0,000896028	NA	NA	NA	NA	NA	NA	NA	NA	NA
ILMN_1666409	PSMB6	0,000925153	NA	NA	NA	NA	NA	NA	NA	NA	NA
ILMN_1763640	KIAA1602	0,000948479	NA	NA	NA	NA	NA	NA	NA	NA	NA
ILMN_1809467	VAMP5	0,000996786	NA	NA	NA	NA	NA	NA	NA	NA	NA
ILMN_1701464	ZNF776	0,000998611	NA	NA	NA	NA	NA	NA	NA	NA	NA
ILMN_3251728	MTMR10	0,001023089	NA	NA	NA	NA	NA	NA	NA	NA	NA
ILMN_1732151	COL6A1	0,001033073	NA	NA	NA	NA	NA	NA	NA	NA	NA
ILMN_3200717	LOC643387	0,001049661	NA	NA	NA	NA	NA	NA	NA	NA	NA
ILMN_1660063	POLE4	0,001078053	NA	NA	NA	NA	NA	NA	NA	NA	NA
ILMN_1676075	KLHL12	0,001086503	NA	NA	NA	NA	NA	NA	NA	NA	NA
ILMN_3237324	MMS19	0,00110463	NA	NA	NA	NA	NA	NA	NA	NA	NA
ILMN_1766762	DYNLRB1	0,001106403	NA	NA	NA	NA	NA	NA	NA	NA	NA
ILMN_2339202	KTN1	0,001106642	NA	NA	NA	NA	NA	NA	NA	NA	NA
ILMN_2102960	KIAA1370	0,00111675	NA	NA	NA	NA	NA	NA	NA	NA	NA
ILMN_1776216	MMGT1	0,001158	NA	NA	NA	NA	NA	NA	NA	NA	NA
ILMN_1714515	MRPS12	0,001165806	NA	NA	NA	NA	NA	NA	NA	NA	NA
ILMN_3261197	ATP5E	0,001174738	NA	NA	NA	NA	NA	NA	NA	NA	NA
ILMN_2371964	MRPS12	0,001175555	NA	NA	NA	NA	NA	NA	NA	NA	NA
ILMN_2230672	MRPL18	0,001192784	NA	NA	NA	NA	NA	NA	NA	NA	NA
ILMN_3185784	LOC1001255	0,001281894	NA	NA	NA	NA	NA	NA	NA	NA	NA
ILMN_1832208		0,001317681	NA	NA	NA	NA	NA	NA	NA	NA	NA
ILMN_1804656	C12orf62	0,001345285	NA	NA	NA	NA	NA	NA	NA	NA	NA
ILMN_1778764	BUB3	0,001345662	NA	NA	NA	NA	NA	NA	NA	NA	NA
ILMN_1768394	ARPC5	0,00135033	NA	NA	NA	NA	NA	NA	NA	NA	NA
ILMN_1693004	C20orf117	0,00137502	NA	NA	NA	NA	NA	NA	NA	NA	NA
ILMN_2401978	STAT3	0,001419476	NA	NA	NA	NA	NA	NA	NA	NA	NA
ILMN_3187680	ACCS	0,001454082	NA	NA	NA	NA	NA	NA	NA	NA	NA
ILMN_1685005	TNFRSF1A	0,001483638	NA	NA	NA	NA	NA	NA	NA	NA	NA
ILMN_1667034	PDPR	0,001506611	NA	NA	NA	NA	NA	NA	NA	NA	NA
ILMN_1654583	CHD1	0,001514176	NA	NA	NA	NA	NA	NA	NA	NA	NA
ILMN_2161746	TRIP10	0,001530158	NA	NA	NA	NA	NA	NA	NA	NA	NA
ILMN_3200414	LOC441131	0,001608166	NA	NA	NA	NA	NA	NA	NA	NA	NA
ILMN_1782459	OSBPL8	0,001635004	NA	NA	NA	NA	NA	NA	NA	NA	NA
ILMN_1800573	RPS21	0,001657317	NA	NA	NA	NA	NA	NA	NA	NA	NA
ILMN_1795170	SLITRK2	0,001662549	NA	NA	NA	NA	NA	NA	NA	NA	NA
ILMN_1701293	COX7A2	0,001670525	NA	NA	NA	NA	NA	NA	NA	NA	NA
ILMN_3229033	LOC732360	0,001676104	NA	NA	NA	NA	NA	NA	NA	NA	NA
ILMN_1800543	SCFD1	0,001714361	NA	NA	NA	NA	NA	NA	NA	NA	NA
ILMN_1784410	UBP1	0,001743189	NA	NA	NA	NA	NA	NA	NA	NA	NA
ILMN_2232936	UQCRH	0,001748814	NA	NA	NA	NA	NA	NA	NA	NA	NA
ILMN_1713394	LOC441876	0,001792902	NA	NA	NA	NA	NA	NA	NA	NA	NA
ILMN_1719032	LSM3	0,001794307	NA	NA	NA	NA	NA	NA	NA	NA	NA
ILMN_1738989	GOLSYN	0,00180602	NA	NA	NA	NA	NA	NA	NA	NA	NA
ILMN_1798533	ZNF22	0,001821172	NA	NA	NA	NA	NA	NA	NA	NA	NA
ILMN_1763433	TRIM9	0,001836545	NA	NA	NA	NA	NA	NA	NA	NA	NA
ILMN_1720431	ZNF420	0,00184994	NA	NA	NA	NA	NA	NA	NA	NA	NA
ILMN_1779547	HPSE	0,001860912	NA	NA	NA	NA	NA	NA	NA	NA	NA
ILMN_2391178	STAR	0,00187097	NA	NA	NA	NA	NA	NA	NA	NA	NA
ILMN_2226917	KIAA0247	0,00190197	NA	NA	NA	NA	NA	NA	NA	NA	NA
ILMN_1765606	YAF2	0,001907274	NA	NA	NA	NA	NA	NA	NA	NA	NA
ILMN_1767422	POLR1D	0,001953556	NA	NA	NA	NA	NA	NA	NA	NA	NA
ILMN_2391522	MRPL47	0,002031628	NA	NA	NA	NA	NA	NA	NA	NA	NA
ILMN_3300198	LOC729580	0,002055755	NA	NA	NA	NA	NA	NA	NA	NA	NA
ILMN_1739345	C11orf48	0,002077808	NA	NA	NA	NA	NA	NA	NA	NA	NA
ILMN_2347805	EXOC1	0,002088326	NA	NA	NA	NA	NA	NA	NA	NA	NA
ILMN_1694742	RPS29	0,002095631	NA	NA	NA	NA	NA	NA	NA	NA	NA
ILMN_2062370	NOL8	0,002102185	NA	NA	NA	NA	NA	NA	NA	NA	NA
ILMN_1673491	LRAT	0,002136629	NA	NA	NA	NA	NA	NA	NA	NA	NA
ILMN_1681269	DYRK4	0,002166481	NA	NA	NA	NA	NA	NA	NA	NA	NA
ILMN_3251629	EIF4A1	0,002205595	NA	NA	NA	NA	NA	NA	NA	NA	NA
ILMN_1662331	PDSS2	0,002220254	NA	NA	NA	NA	NA	NA	NA	NA	NA
ILMN_1743767	ZNF446	0,002221063	NA	NA	NA	NA	NA	NA	NA	NA	NA
ILMN_1677800	SGTA	0,002224188	NA	NA	NA	NA	NA	NA	NA	NA	NA
ILMN_1693014	CEBPB	0,002231556	NA	NA	NA	NA	NA	NA	NA	NA	NA
ILMN_1724789	CD59	0,002245208	NA	NA	NA	NA	NA	NA	NA	NA	NA
ILMN_1803136	RAB4B	0,002245241	NA	NA	NA	NA	NA	NA	NA	NA	NA
ILMN_2076250	GPBP1L1	0,002264003	NA	NA	NA	NA	NA	NA	NA	NA	NA

ILMN_1721093	TAF10	0,002273687	NA	NA	NA	NA	NA	NA	NA	NA	NA
ILMN_3286685	LOC1001333	0,002298762	NA	NA	NA	NA	NA	NA	NA	NA	NA
ILMN_1672331	MAP3K7IP2	0,00231085	NA	NA	NA	NA	NA	NA	NA	NA	NA
ILMN_3282503	LOC644101	0,002312125	NA	NA	NA	NA	NA	NA	NA	NA	NA
ILMN_1739622	PPP1R12A	0,002339396	NA	NA	NA	NA	NA	NA	NA	NA	NA
ILMN_2179018	NDUFAB1	0,002341623	NA	NA	NA	NA	NA	NA	NA	NA	NA
ILMN_1778488	WDR41	0,002356572	NA	NA	NA	NA	NA	NA	NA	NA	NA
ILMN_2065773	SCG5	0,002374969	NA	NA	NA	NA	NA	NA	NA	NA	NA
ILMN_1771620	SNRPB2	0,002395607	NA	NA	NA	NA	NA	NA	NA	NA	NA
ILMN_1745343	ZMAT2	0,002428774	NA	NA	NA	NA	NA	NA	NA	NA	NA
ILMN_1688479	LRRC42	0,002446416	NA	NA	NA	NA	NA	NA	NA	NA	NA
ILMN_1679912	DPH3	0,002468938	NA	NA	NA	NA	NA	NA	NA	NA	NA
ILMN_1689747	NOL8	0,002471438	NA	NA	NA	NA	NA	NA	NA	NA	NA
ILMN_1801633	C20orf52	0,002507594	NA	NA	NA	NA	NA	NA	NA	NA	NA
ILMN_2201533	C17orf61	0,002520609	NA	NA	NA	NA	NA	NA	NA	NA	NA
ILMN_1815320	NR5A1	0,002522454	NA	NA	NA	NA	NA	NA	NA	NA	NA
ILMN_1743538	MLLT10	0,002524472	NA	NA	NA	NA	NA	NA	NA	NA	NA
ILMN_1764500	C3orf10	0,002550112	NA	NA	NA	NA	NA	NA	NA	NA	NA
ILMN_1767365	PAK1	0,002551223	NA	NA	NA	NA	NA	NA	NA	NA	NA
ILMN_1658834	ZC3H18	0,002577911	NA	NA	NA	NA	NA	NA	NA	NA	NA
ILMN_2376408	DPM3	0,00263236	NA	NA	NA	NA	NA	NA	NA	NA	NA
ILMN_1752247	AKAP13	0,002635683	NA	NA	NA	NA	NA	NA	NA	NA	NA
ILMN_1753534	RPS18	0,00270526	NA	NA	NA	NA	NA	NA	NA	NA	NA
ILMN_1741491	ZNHIT1	0,002741545	NA	NA	NA	NA	NA	NA	NA	NA	NA
ILMN_1744302	LOC645489	0,002746723	NA	NA	NA	NA	NA	NA	NA	NA	NA
ILMN_2124155	ATP11B	0,002747288	NA	NA	NA	NA	NA	NA	NA	NA	NA
ILMN_2063586	CLIC4	0,002781747	NA	NA	NA	NA	NA	NA	NA	NA	NA
ILMN_3286067	LOC391578	0,002783695	NA	NA	NA	NA	NA	NA	NA	NA	NA
ILMN_3287583	LOC648390	0,002788432	NA	NA	NA	NA	NA	NA	NA	NA	NA
ILMN_1739161	PPAP2A	0,002796362	NA	NA	NA	NA	NA	NA	NA	NA	NA
ILMN_1667306	RANBP10	0,002815408	NA	NA	NA	NA	NA	NA	NA	NA	NA
ILMN_1693227	ZC3H7A	0,002817611	NA	NA	NA	NA	NA	NA	NA	NA	NA
ILMN_1705151	SF3A3	0,002820801	NA	NA	NA	NA	NA	NA	NA	NA	NA
ILMN_1839750	LOC729776	0,002824918	NA	NA	NA	NA	NA	NA	NA	NA	NA
ILMN_2139052	ZKSCAN1	0,002858408	NA	NA	NA	NA	NA	NA	NA	NA	NA
ILMN_1706305	C14orf112	0,00288718	NA	NA	NA	NA	NA	NA	NA	NA	NA
ILMN_1797342	FNBP1	0,002888151	NA	NA	NA	NA	NA	NA	NA	NA	NA
ILMN_1813260	TIMM17B	0,002917598	NA	NA	NA	NA	NA	NA	NA	NA	NA
ILMN_1735432	ISCU	0,002937107	NA	NA	NA	NA	NA	NA	NA	NA	NA
ILMN_2299612	TMEM150A	0,002976888	NA	NA	NA	NA	NA	NA	NA	NA	NA
ILMN_1771261	SYNC1	0,002988025	NA	NA	NA	NA	NA	NA	NA	NA	NA
ILMN_1751627	TRAPPC3	0,003000979	NA	NA	NA	NA	NA	NA	NA	NA	NA
ILMN_1730931	RUFY3	0,00300339	NA	NA	NA	NA	NA	NA	NA	NA	NA
ILMN_3251451	MED31	0,003037603	NA	NA	NA	NA	NA	NA	NA	NA	NA
ILMN_1785570	SUSD3	0,00304274	NA	NA	NA	NA	NA	NA	NA	NA	NA
ILMN_1736042	ME1	0,003064531	NA	NA	NA	NA	NA	NA	NA	NA	NA
ILMN_1774860	UTP23	0,003081316	NA	NA	NA	NA	NA	NA	NA	NA	NA
ILMN_2404135	RIOK3	0,003140169	NA	NA	NA	NA	NA	NA	NA	NA	NA
ILMN_3241218	ANKIB1	0,003152039	NA	NA	NA	NA	NA	NA	NA	NA	NA
ILMN_1769615	FLRT2	0,003152125	NA	NA	NA	NA	NA	NA	NA	NA	NA
ILMN_3246850	LOC648729	0,003162736	NA	NA	NA	NA	NA	NA	NA	NA	NA
ILMN_1666761	PPP2R5E	0,003180745	NA	NA	NA	NA	NA	NA	NA	NA	NA
ILMN_2311041	MRPL52	0,003207394	NA	NA	NA	NA	NA	NA	NA	NA	NA
ILMN_3291986	LOC642892	0,003223163	NA	NA	NA	NA	NA	NA	NA	NA	NA
ILMN_3246065	CCDC151	0,003249556	NA	NA	NA	NA	NA	NA	NA	NA	NA
ILMN_1737211	ZNF585A	0,003266704	NA	NA	NA	NA	NA	NA	NA	NA	NA
ILMN_1687351	ANKRA2	0,003282776	NA	NA	NA	NA	NA	NA	NA	NA	NA
ILMN_1672446	RPL11	0,00329955	NA	NA	NA	NA	NA	NA	NA	NA	NA
ILMN_1763460	NHP2L1	0,00331336	NA	NA	NA	NA	NA	NA	NA	NA	NA
ILMN_1704713	CSNK1G1	0,003314726	NA	NA	NA	NA	NA	NA	NA	NA	NA
ILMN_1774334	HIGD2A	0,003318842	NA	NA	NA	NA	NA	NA	NA	NA	NA
ILMN_1666670	RBX1	0,003376852	NA	NA	NA	NA	NA	NA	NA	NA	NA
ILMN_1785356	DENND5A	0,003399422	NA	NA	NA	NA	NA	NA	NA	NA	NA
ILMN_1653001	CABLES1	0,003438288	NA	NA	NA	NA	NA	NA	NA	NA	NA
ILMN_1686388	LOC644330	0,003457391	NA	NA	NA	NA	NA	NA	NA	NA	NA
ILMN_1652806	ATP5J	0,003468203	NA	NA	NA	NA	NA	NA	NA	NA	NA
ILMN_1730612	DBNDD2	0,003489131	NA	NA	NA	NA	NA	NA	NA	NA	NA
ILMN_1801710	APBB1IP	0,00348946	NA	NA	NA	NA	NA	NA	NA	NA	NA
ILMN_1712577	FAM174A	0,003495685	NA	NA	NA	NA	NA	NA	NA	NA	NA
ILMN_1716895	RPA3	0,003509966	NA	NA	NA	NA	NA	NA	NA	NA	NA
ILMN_1695461	PTPRA	0,003637169	NA	NA	NA	NA	NA	NA	NA	NA	NA
ILMN_1752837	ARL8B	0,003641646	NA	NA	NA	NA	NA	NA	NA	NA	NA
ILMN_1788462	AMD1	0,003648282	NA	NA	NA	NA	NA	NA	NA	NA	NA
ILMN_3243065	FRG2C	0,003666795	NA	NA	NA	NA	NA	NA	NA	NA	NA
ILMN_1675852	LOC650518	0,003668157	NA	NA	NA	NA	NA	NA	NA	NA	NA
ILMN_1765132	LACTB	0,003685758	NA	NA	NA	NA	NA	NA	NA	NA	NA
ILMN_3256004	LOC1001300	0,003703124	NA	NA	NA	NA	NA	NA	NA	NA	NA
ILMN_1733377	FASTKD5	0,003714764	NA	NA	NA	NA	NA	NA	NA	NA	NA
ILMN_3234384	LOC728953	0,003720561	NA	NA	NA	NA	NA	NA	NA	NA	NA
ILMN_1719064	KCTD10	0,003725755	NA	NA	NA	NA	NA	NA	NA	NA	NA
ILMN_3304130	LOC729236	0,003741079	NA	NA	NA	NA	NA	NA	NA	NA	NA
ILMN_2153373	LRBA	0,003742593	NA	NA	NA	NA	NA	NA	NA	NA	NA
ILMN_1670028	LPIN2	0,003744841	NA	NA	NA	NA	NA	NA	NA	NA	NA
ILMN_1686805	CRK	0,003747095	NA	NA	NA	NA	NA	NA	NA	NA	NA
ILMN_1733927	TCEB2	0,003754982	NA	NA	NA	NA	NA	NA	NA	NA	NA
ILMN_2307883	ATP5J2	0,003784956	NA	NA	NA	NA	NA	NA	NA	NA	NA
ILMN_3246722	LOC1001342	0,003788627	NA	NA	NA	NA	NA	NA	NA	NA	NA
ILMN_1652230	LOC647361	0,003790371	NA	NA	NA	NA	NA	NA	NA	NA	NA
ILMN_1739263	LOC643284	0,00379688	NA	NA	NA	NA	NA	NA	NA	NA	NA
ILMN_1670456	RBM42	0,003805429	NA	NA	NA	NA	NA	NA	NA	NA	NA
ILMN_1724609	SLC2A8	0,003863845	NA	NA	NA	NA	NA	NA	NA	NA	NA
ILMN_2055634	TIAF1	0,003943724	NA	NA	NA	NA	NA	NA	NA	NA	NA
ILMN_1677038	FLJ21986	0,003966695	NA	NA	NA	NA	NA	NA	NA	NA	NA
ILMN_1745885	POLR2F	0,003986764	NA	NA	NA	NA	NA	NA	NA	NA	NA
ILMN_2107933	ZNF285A	0,003989596	NA	NA	NA	NA	NA	NA	NA	NA	NA
ILMN_1683082	RPUSD1	0,003992215	NA	NA	NA	NA	NA	NA	NA	NA	NA
ILMN_1813344	C20orf7	0,004013194	NA	NA	NA	NA	NA	NA	NA	NA	NA
ILMN_1755883	RPS27A	0,004045039	NA	NA	NA	NA	NA	NA	NA	NA	NA
ILMN_1722102	ANAPC11	0,004113754	NA	NA	NA	NA	NA	NA	NA	NA	NA
ILMN_1801795	LOC347544	0,004128504	NA	NA	NA	NA	NA	NA	NA	NA	NA
ILMN_1813489	RAF1	0,004177092	NA	NA	NA	NA	NA	NA	NA	NA	NA
ILMN_1815705	LZTFL1	0,004195165	NA	NA	NA	NA	NA	NA	NA	NA	NA
ILMN_1783805	PNMA3	0,004214684	NA	NA	NA	NA	NA	NA	NA	NA	NA
ILMN_2182348	SMC3	0,004220983	NA	NA	NA	NA	NA	NA	NA	NA	NA
ILMN_1670870	ALCAM	0,004234686	NA	NA	NA	NA	NA	NA	NA	NA	NA
ILMN_1655311	LOC145853	0,004265507	NA	NA	NA	NA	NA	NA	NA	NA	NA
ILMN_2226955	VOPP1	0,004276795	NA	NA	NA	NA	NA	NA	NA	NA	NA
ILMN_3291709	LOC402175	0,004314839	NA	NA	NA	NA	NA	NA	NA	NA	NA
ILMN_1655563	KIAA0427	0,004355496	NA	NA	NA	NA	NA	NA	NA	NA	NA

ILMN_1789171	EEF2K	0,004356931	NA	NA	NA	NA	NA	NA	NA	NA	NA
ILMN_1703305	TWF2	0,004372001	NA	NA	NA	NA	NA	NA	NA	NA	NA
ILMN_1678766	DYNLT1	0,004372498	NA	NA	NA	NA	NA	NA	NA	NA	NA
ILMN_3227060	LOC728931	0,00439258	NA	NA	NA	NA	NA	NA	NA	NA	NA
ILMN_2051381	ALDH16A1	0,00441888	NA	NA	NA	NA	NA	NA	NA	NA	NA
ILMN_3247006	LOC1001343	0,004426909	NA	NA	NA	NA	NA	NA	NA	NA	NA
ILMN_1756126	STUB1	0,004429413	NA	NA	NA	NA	NA	NA	NA	NA	NA
ILMN_2403852	CYTH1	0,004438547	NA	NA	NA	NA	NA	NA	NA	NA	NA
ILMN_1674609	CLTB	0,004438555	NA	NA	NA	NA	NA	NA	NA	NA	NA
ILMN_2313926	CDC42SE2	0,00445918	NA	NA	NA	NA	NA	NA	NA	NA	NA
ILMN_1658802	KRTCAP2	0,004497602	NA	NA	NA	NA	NA	NA	NA	NA	NA
ILMN_1696190	STRN4	0,004509758	NA	NA	NA	NA	NA	NA	NA	NA	NA
ILMN_2162328	PTS	0,004543616	NA	NA	NA	NA	NA	NA	NA	NA	NA
ILMN_1766797	CCS	0,00454952	NA	NA	NA	NA	NA	NA	NA	NA	NA
ILMN_1788192	ARMCX1	0,004549998	NA	NA	NA	NA	NA	NA	NA	NA	NA
ILMN_2295518	TRO	0,004578447	NA	NA	NA	NA	NA	NA	NA	NA	NA
ILMN_1667657	TPP2	0,004626649	NA	NA	NA	NA	NA	NA	NA	NA	NA
ILMN_1669633	ACP1	0,00463662	NA	NA	NA	NA	NA	NA	NA	NA	NA
ILMN_2198515	ARRDC3	0,004648205	NA	NA	NA	NA	NA	NA	NA	NA	NA
ILMN_1801443	TSKU	0,004656429	NA	NA	NA	NA	NA	NA	NA	NA	NA
ILMN_2369924	NDUFB6	0,004736565	NA	NA	NA	NA	NA	NA	NA	NA	NA
ILMN_3292082	LOC642367	0,004756074	NA	NA	NA	NA	NA	NA	NA	NA	NA
ILMN_1663512	COX5B	0,004794305	NA	NA	NA	NA	NA	NA	NA	NA	NA
ILMN_3249949	C11orf58	0,004797421	NA	NA	NA	NA	NA	NA	NA	NA	NA
ILMN_1683277	KIAA0319L	0,004803576	NA	NA	NA	NA	NA	NA	NA	NA	NA
ILMN_1766195	ZNF558	0,004812051	NA	NA	NA	NA	NA	NA	NA	NA	NA
ILMN_1727809	STK35	0,00481827	NA	NA	NA	NA	NA	NA	NA	NA	NA
ILMN_2402674	DPM3	0,004839416	NA	NA	NA	NA	NA	NA	NA	NA	NA
ILMN_1772929	ATP5J	0,004867119	NA	NA	NA	NA	NA	NA	NA	NA	NA
ILMN_3301440	LOC730004	0,004867693	NA	NA	NA	NA	NA	NA	NA	NA	NA
ILMN_2053829	CBLN3	0,004932224	NA	NA	NA	NA	NA	NA	NA	NA	NA
ILMN_1752810	LARP6	0,004950084	NA	NA	NA	NA	NA	NA	NA	NA	NA
ILMN_1789176	PSMB1	0,0049764	NA	NA	NA	NA	NA	NA	NA	NA	NA
ILMN_1699676	C14orf147	0,004992629	NA	NA	NA	NA	NA	NA	NA	NA	NA
ILMN_1693862	MGC70857	0,005065062	NA	NA	NA	NA	NA	NA	NA	NA	NA
ILMN_1754303	RPL30	0,00507445	NA	NA	NA	NA	NA	NA	NA	NA	NA
ILMN_3181695	LOC1001301	0,005143183	NA	NA	NA	NA	NA	NA	NA	NA	NA
ILMN_1688464	MAP6D1	0,005156085	NA	NA	NA	NA	NA	NA	NA	NA	NA
ILMN_1673421	LOC440704	0,005165062	NA	NA	NA	NA	NA	NA	NA	NA	NA
ILMN_2224486	C3orf14	0,005167973	NA	NA	NA	NA	NA	NA	NA	NA	NA
ILMN_1697962	NSMCE1	0,005186588	NA	NA	NA	NA	NA	NA	NA	NA	NA
ILMN_1720322	PTS	0,005250123	NA	NA	NA	NA	NA	NA	NA	NA	NA
ILMN_1659845	KIAA0355	0,005340664	NA	NA	NA	NA	NA	NA	NA	NA	NA
ILMN_1765829	NUFIP2	0,005355587	NA	NA	NA	NA	NA	NA	NA	NA	NA
ILMN_2081335	C7orf44	0,005355691	NA	NA	NA	NA	NA	NA	NA	NA	NA
ILMN_1704079	RBM38	0,005361785	NA	NA	NA	NA	NA	NA	NA	NA	NA
ILMN_1656662	LOC731365	0,005373032	NA	NA	NA	NA	NA	NA	NA	NA	NA
ILMN_2279339	WTAP	0,005402008	NA	NA	NA	NA	NA	NA	NA	NA	NA
ILMN_1671731	AVPI1	0,005417397	NA	NA	NA	NA	NA	NA	NA	NA	NA
ILMN_3292678	LOC1001332	0,005467588	NA	NA	NA	NA	NA	NA	NA	NA	NA
ILMN_1781039	VPS26	0,005492469	NA	NA	NA	NA	NA	NA	NA	NA	NA
ILMN_1700733	KIAA1370	0,00549329	NA	NA	NA	NA	NA	NA	NA	NA	NA
ILMN_2221673	ASNSD1	0,005506528	NA	NA	NA	NA	NA	NA	NA	NA	NA
ILMN_2098446	PMAIP1	0,005517519	NA	NA	NA	NA	NA	NA	NA	NA	NA
ILMN_1675577	TRMT61A	0,005566174	NA	NA	NA	NA	NA	NA	NA	NA	NA
ILMN_1728514	BAG5	0,005586246	NA	NA	NA	NA	NA	NA	NA	NA	NA
ILMN_3279322	LOC645174	0,005605116	NA	NA	NA	NA	NA	NA	NA	NA	NA
ILMN_1772527	C12orf44	0,005607359	NA	NA	NA	NA	NA	NA	NA	NA	NA
ILMN_3235912	C7orf38	0,005634003	NA	NA	NA	NA	NA	NA	NA	NA	NA
ILMN_1790625	CBX3	0,005636642	NA	NA	NA	NA	NA	NA	NA	NA	NA
ILMN_1732149	AQP2	0,005716253	NA	NA	NA	NA	NA	NA	NA	NA	NA
ILMN_1787949	RPS15A	0,005742792	NA	NA	NA	NA	NA	NA	NA	NA	NA
ILMN_1671054	HLA-A	0,005763106	NA	NA	NA	NA	NA	NA	NA	NA	NA
ILMN_2338963	SLC29A1	0,005767472	NA	NA	NA	NA	NA	NA	NA	NA	NA
ILMN_1803302	CRK	0,005791783	NA	NA	NA	NA	NA	NA	NA	NA	NA
ILMN_1791388	ZNF787	0,005814024	NA	NA	NA	NA	NA	NA	NA	NA	NA
ILMN_2121437	NCL	0,00582684	NA	NA	NA	NA	NA	NA	NA	NA	NA
ILMN_1784286	NDUFA1	0,005837923	NA	NA	NA	NA	NA	NA	NA	NA	NA
ILMN_3273493	LOC1001300	0,005846702	NA	NA	NA	NA	NA	NA	NA	NA	NA
ILMN_3250209	CTBP2	0,005857172	NA	NA	NA	NA	NA	NA	NA	NA	NA
ILMN_3241169	C3orf75	0,00587152	NA	NA	NA	NA	NA	NA	NA	NA	NA
ILMN_1715574	ZC3H11A	0,005888888	NA	NA	NA	NA	NA	NA	NA	NA	NA
ILMN_2283388	C20orf24	0,00589788	NA	NA	NA	NA	NA	NA	NA	NA	NA
ILMN_1896406		0,005919268	NA	NA	NA	NA	NA	NA	NA	NA	NA
ILMN_3236373	MSL2	0,005969002	NA	NA	NA	NA	NA	NA	NA	NA	NA
ILMN_2361695	BAG5	0,005969969	NA	NA	NA	NA	NA	NA	NA	NA	NA
ILMN_1667257	SDHB	0,005983762	NA	NA	NA	NA	NA	NA	NA	NA	NA
ILMN_1654543	MED6	0,006003888	NA	NA	NA	NA	NA	NA	NA	NA	NA
ILMN_1653501	SEMA3B	0,00606113	NA	NA	NA	NA	NA	NA	NA	NA	NA
ILMN_2307598	SLC37A3	0,006101272	NA	NA	NA	NA	NA	NA	NA	NA	NA
ILMN_1787591	XPA	0,006138748	NA	NA	NA	NA	NA	NA	NA	NA	NA
ILMN_1729115	LOC651816	0,006161891	NA	NA	NA	NA	NA	NA	NA	NA	NA
ILMN_2212690	ZC3H7A	0,006182523	NA	NA	NA	NA	NA	NA	NA	NA	NA
ILMN_2221076	C17orf85	0,006210155	NA	NA	NA	NA	NA	NA	NA	NA	NA
ILMN_1790973	CDS2	0,006275623	NA	NA	NA	NA	NA	NA	NA	NA	NA
ILMN_1657317	POLR2J	0,006308762	NA	NA	NA	NA	NA	NA	NA	NA	NA
ILMN_2143487	TAF1B	0,006371437	NA	NA	NA	NA	NA	NA	NA	NA	NA
ILMN_1653220	PITPNM1	0,006403671	NA	NA	NA	NA	NA	NA	NA	NA	NA
ILMN_1755536	PFDN5	0,006409395	NA	NA	NA	NA	NA	NA	NA	NA	NA
ILMN_1726603	ATP5I	0,006432715	NA	NA	NA	NA	NA	NA	NA	NA	NA
ILMN_1706498	DSE	0,006465625	NA	NA	NA	NA	NA	NA	NA	NA	NA
ILMN_1788059	PCGF5	0,006468148	NA	NA	NA	NA	NA	NA	NA	NA	NA
ILMN_1738718	CYB561D2	0,006493081	NA	NA	NA	NA	NA	NA	NA	NA	NA
ILMN_1790230	ZNF181	0,006514291	NA	NA	NA	NA	NA	NA	NA	NA	NA
ILMN_1753719	GTF2A2	0,006519211	NA	NA	NA	NA	NA	NA	NA	NA	NA
ILMN_1790533	PHACTR2	0,006587941	NA	NA	NA	NA	NA	NA	NA	NA	NA
ILMN_1732489	SLC10A7	0,006594063	NA	NA	NA	NA	NA	NA	NA	NA	NA
ILMN_3248069	LOC653881	0,006655101	NA	NA	NA	NA	NA	NA	NA	NA	NA
ILMN_1769158	ISOC2	0,006670007	NA	NA	NA	NA	NA	NA	NA	NA	NA
ILMN_3234993	ROMO1	0,00669339	NA	NA	NA	NA	NA	NA	NA	NA	NA
ILMN_1733757	LOC374395	0,006694878	NA	NA	NA	NA	NA	NA	NA	NA	NA
ILMN_2300186	DYNLL1	0,00677187	NA	NA	NA	NA	NA	NA	NA	NA	NA
ILMN_2310621	ATP5J2	0,006772735	NA	NA	NA	NA	NA	NA	NA	NA	NA
ILMN_3253304	BRI3P1	0,006793193	NA	NA	NA	NA	NA	NA	NA	NA	NA
ILMN_1691846	G0S2	0,006796776	NA	NA	NA	NA	NA	NA	NA	NA	NA
ILMN_1716907	FLJ20254	0,006823337	NA	NA	NA	NA	NA	NA	NA	NA	NA
ILMN_1729816	VDAC3	0,006853822	NA	NA	NA	NA	NA	NA	NA	NA	NA
ILMN_1727183	ZNF763	0,006908959	NA	NA	NA	NA	NA	NA	NA	NA	NA
ILMN_1761072	LOC653631	0,006916801	NA	NA	NA	NA	NA	NA	NA	NA	NA

ILMN_3240754	FAM90A3	0,006947791	NA	NA	NA	NA	NA	NA	NA	NA	NA
ILMN_1659761	SNX29	0,006960316	NA	NA	NA	NA	NA	NA	NA	NA	NA
ILMN_1696622	SLC38A6	0,006990528	NA	NA	NA	NA	NA	NA	NA	NA	NA
ILMN_1716552	ENAH	0,006996552	NA	NA	NA	NA	NA	NA	NA	NA	NA
ILMN_2255310	RPS15A	0,007043194	NA	NA	NA	NA	NA	NA	NA	NA	NA
ILMN_1672149	CHCHD1	0,007059847	NA	NA	NA	NA	NA	NA	NA	NA	NA
ILMN_1659075	HLA-DOA	0,007062763	NA	NA	NA	NA	NA	NA	NA	NA	NA
ILMN_1779813	FAM96B	0,007084419	NA	NA	NA	NA	NA	NA	NA	NA	NA
ILMN_1680692	NUCKS1	0,007111477	NA	NA	NA	NA	NA	NA	NA	NA	NA
ILMN_1812312	NDUFS4	0,007159913	NA	NA	NA	NA	NA	NA	NA	NA	NA
ILMN_1718950	ZNF433	0,007223882	NA	NA	NA	NA	NA	NA	NA	NA	NA
ILMN_2052863	RNF5P1	0,007230387	NA	NA	NA	NA	NA	NA	NA	NA	NA
ILMN_3248975	PPP4C	0,007271312	NA	NA	NA	NA	NA	NA	NA	NA	NA
ILMN_3238006	LOC730323	0,007296847	NA	NA	NA	NA	NA	NA	NA	NA	NA
ILMN_1655225	C19orf25	0,007312006	NA	NA	NA	NA	NA	NA	NA	NA	NA
ILMN_1703153	N-PAC	0,00731951	NA	NA	NA	NA	NA	NA	NA	NA	NA
ILMN_1658992	DPM1	0,007332507	NA	NA	NA	NA	NA	NA	NA	NA	NA
ILMN_3247064	SNRNP40	0,007384849	NA	NA	NA	NA	NA	NA	NA	NA	NA
ILMN_3235392	LOC653590	0,007400081	NA	NA	NA	NA	NA	NA	NA	NA	NA
ILMN_2249473	SPTLC1	0,007455182	NA	NA	NA	NA	NA	NA	NA	NA	NA
ILMN_1787251	DAAM1	0,007455627	NA	NA	NA	NA	NA	NA	NA	NA	NA
ILMN_1700232	FBXO30	0,007479882	NA	NA	NA	NA	NA	NA	NA	NA	NA
ILMN_1768396	AURKAIP1	0,007496615	NA	NA	NA	NA	NA	NA	NA	NA	NA
ILMN_1708787	ZNF627	0,00750348	NA	NA	NA	NA	NA	NA	NA	NA	NA
ILMN_3305849	LOC728431	0,007510856	NA	NA	NA	NA	NA	NA	NA	NA	NA
ILMN_1701134	PTEN	0,007527556	NA	NA	NA	NA	NA	NA	NA	NA	NA
ILMN_1756355	NDUFS3	0,007546152	NA	NA	NA	NA	NA	NA	NA	NA	NA
ILMN_2157435	DYNLRB1	0,007557284	NA	NA	NA	NA	NA	NA	NA	NA	NA
ILMN_1763884	Magmas	0,007594812	NA	NA	NA	NA	NA	NA	NA	NA	NA
ILMN_1745242	PLSCR1	0,007678025	NA	NA	NA	NA	NA	NA	NA	NA	NA
ILMN_2159322	SLK	0,007748732	NA	NA	NA	NA	NA	NA	NA	NA	NA
ILMN_1679655	WDR82	0,007763775	NA	NA	NA	NA	NA	NA	NA	NA	NA
ILMN_1766115	PLEKHF2	0,007775235	NA	NA	NA	NA	NA	NA	NA	NA	NA
ILMN_1726729	C2orf76	0,007776398	NA	NA	NA	NA	NA	NA	NA	NA	NA
ILMN_1707137	C17orf97	0,00777806	NA	NA	NA	NA	NA	NA	NA	NA	NA
ILMN_1706326	MRPL33	0,007789696	NA	NA	NA	NA	NA	NA	NA	NA	NA
ILMN_1679178	ATP5D	0,007791934	NA	NA	NA	NA	NA	NA	NA	NA	NA
ILMN_1721034	ZNF227	0,007818831	NA	NA	NA	NA	NA	NA	NA	NA	NA
ILMN_1760493	LIMS2	0,007827012	NA	NA	NA	NA	NA	NA	NA	NA	NA
ILMN_1679891	NAF1	0,007851394	NA	NA	NA	NA	NA	NA	NA	NA	NA
ILMN_2367681	SPAG1	0,007856208	NA	NA	NA	NA	NA	NA	NA	NA	NA
ILMN_1788457	ABCC4	0,00788276	NA	NA	NA	NA	NA	NA	NA	NA	NA
ILMN_1783985	COQ6	0,007910292	NA	NA	NA	NA	NA	NA	NA	NA	NA
ILMN_1745807	TMEM62	0,007920865	NA	NA	NA	NA	NA	NA	NA	NA	NA
ILMN_1717261	HLA-DRB3	0,007959254	NA	NA	NA	NA	NA	NA	NA	NA	NA
ILMN_3225102	ZNF738	0,007973796	NA	NA	NA	NA	NA	NA	NA	NA	NA
ILMN_3262348	IP6K2	0,007974435	NA	NA	NA	NA	NA	NA	NA	NA	NA
ILMN_1655011	SERF1B	0,007986278	NA	NA	NA	NA	NA	NA	NA	NA	NA
ILMN_1797668	INSM1	0,007998447	NA	NA	NA	NA	NA	NA	NA	NA	NA
ILMN_3283592	LOC442609	0,008042143	NA	NA	NA	NA	NA	NA	NA	NA	NA
ILMN_1663313	AMY1C	0,008080509	NA	NA	NA	NA	NA	NA	NA	NA	NA
ILMN_3200299	LOC646942	0,008087759	NA	NA	NA	NA	NA	NA	NA	NA	NA
ILMN_2356909	MOSPD3	0,008107201	NA	NA	NA	NA	NA	NA	NA	NA	NA
ILMN_1687080	LOC402251	0,008128215	NA	NA	NA	NA	NA	NA	NA	NA	NA
ILMN_1810423	RPP40	0,008202752	NA	NA	NA	NA	NA	NA	NA	NA	NA
ILMN_1771884	ZNF45	0,008221111	NA	NA	NA	NA	NA	NA	NA	NA	NA
ILMN_2133936	ZFP1	0,008221824	NA	NA	NA	NA	NA	NA	NA	NA	NA
ILMN_2041788	PLS3	0,008251349	NA	NA	NA	NA	NA	NA	NA	NA	NA
ILMN_2319996	RPL3	0,00826204	NA	NA	NA	NA	NA	NA	NA	NA	NA
ILMN_1705814	KRT80	0,008286272	NA	NA	NA	NA	NA	NA	NA	NA	NA
ILMN_1674024	IKZF5	0,008297935	NA	NA	NA	NA	NA	NA	NA	NA	NA
ILMN_1701173	KCNK6	0,008314277	NA	NA	NA	NA	NA	NA	NA	NA	NA
ILMN_1714335	RDH10	0,008322688	NA	NA	NA	NA	NA	NA	NA	NA	NA
ILMN_1682095	RCOR3	0,008322891	NA	NA	NA	NA	NA	NA	NA	NA	NA
ILMN_1808769	C1orf97	0,008332517	NA	NA	NA	NA	NA	NA	NA	NA	NA
ILMN_1741957	RABEPK	0,008340157	NA	NA	NA	NA	NA	NA	NA	NA	NA
ILMN_1678353	FARP1	0,008362338	NA	NA	NA	NA	NA	NA	NA	NA	NA
ILMN_2129234	TMEM47	0,008380596	NA	NA	NA	NA	NA	NA	NA	NA	NA
ILMN_1763147	NDUFB6	0,008425013	NA	NA	NA	NA	NA	NA	NA	NA	NA
ILMN_1706246	CCT5	0,008431193	NA	NA	NA	NA	NA	NA	NA	NA	NA
ILMN_1740523	KTN1	0,008439215	NA	NA	NA	NA	NA	NA	NA	NA	NA
ILMN_2059173	SLC35E1	0,008446606	NA	NA	NA	NA	NA	NA	NA	NA	NA
ILMN_1655348	GPR1	0,008500701	NA	NA	NA	NA	NA	NA	NA	NA	NA
ILMN_1705241	TDRD7	0,008562136	NA	NA	NA	NA	NA	NA	NA	NA	NA
ILMN_2050911	SLC22A4	0,008620624	NA	NA	NA	NA	NA	NA	NA	NA	NA
ILMN_1764871	PIGP	0,00863281	NA	NA	NA	NA	NA	NA	NA	NA	NA
ILMN_1777344	RPS13	0,008639311	NA	NA	NA	NA	NA	NA	NA	NA	NA
ILMN_2354515	NDUFAF3	0,008677776	NA	NA	NA	NA	NA	NA	NA	NA	NA
ILMN_1813028	CBX5	0,008719324	NA	NA	NA	NA	NA	NA	NA	NA	NA
ILMN_1678612	ANXA6	0,008732482	NA	NA	NA	NA	NA	NA	NA	NA	NA
ILMN_1742318	RNF121	0,008801563	NA	NA	NA	NA	NA	NA	NA	NA	NA
ILMN_1705064	NDEL1	0,008807841	NA	NA	NA	NA	NA	NA	NA	NA	NA
ILMN_1711124	MARVELD2	0,008835893	NA	NA	NA	NA	NA	NA	NA	NA	NA
ILMN_1714945	ITPKC	0,008853563	NA	NA	NA	NA	NA	NA	NA	NA	NA
ILMN_1683065	C2orf64	0,008866873	NA	NA	NA	NA	NA	NA	NA	NA	NA
ILMN_3245006	C12orf68	0,008962581	NA	NA	NA	NA	NA	NA	NA	NA	NA
ILMN_1667994	AMD1	0,008970536	NA	NA	NA	NA	NA	NA	NA	NA	NA
ILMN_1750518	THOC4	0,008980629	NA	NA	NA	NA	NA	NA	NA	NA	NA
ILMN_1813104	GALNTL2	0,009057261	NA	NA	NA	NA	NA	NA	NA	NA	NA
ILMN_2151818	PSMA6	0,009069866	NA	NA	NA	NA	NA	NA	NA	NA	NA
ILMN_1786759	C11orf10	0,009095613	NA	NA	NA	NA	NA	NA	NA	NA	NA
ILMN_2391176	STAR	0,009099526	NA	NA	NA	NA	NA	NA	NA	NA	NA
ILMN_1739335	LOC400948	0,009136455	NA	NA	NA	NA	NA	NA	NA	NA	NA
ILMN_1714461	RNF14	0,009157332	NA	NA	NA	NA	NA	NA	NA	NA	NA
ILMN_1678671	KLHL24	0,009171256	NA	NA	NA	NA	NA	NA	NA	NA	NA
ILMN_3276016	LOC339843	0,009206915	NA	NA	NA	NA	NA	NA	NA	NA	NA
ILMN_2368292	TSEN34	0,009243774	NA	NA	NA	NA	NA	NA	NA	NA	NA
ILMN_1846771		0,009300543	NA	NA	NA	NA	NA	NA	NA	NA	NA
ILMN_1669142	NARG1	0,009311001	NA	NA	NA	NA	NA	NA	NA	NA	NA
ILMN_2093720	THG1L	0,009349699	NA	NA	NA	NA	NA	NA	NA	NA	NA
ILMN_2383913	WDR33	0,009351393	NA	NA	NA	NA	NA	NA	NA	NA	NA
ILMN_1915076		0,009360046	NA	NA	NA	NA	NA	NA	NA	NA	NA
ILMN_2119945	NDUFB3	0,009505209	NA	NA	NA	NA	NA	NA	NA	NA	NA
ILMN_1747217	C15orf41	0,009541952	NA	NA	NA	NA	NA	NA	NA	NA	NA
ILMN_1704094	PSMA6	0,009616764	NA	NA	NA	NA	NA	NA	NA	NA	NA
ILMN_3238106	FAM161A	0,009618148	NA	NA	NA	NA	NA	NA	NA	NA	NA
ILMN_1737015	RPL39	0,009640617	NA	NA	NA	NA	NA	NA	NA	NA	NA
ILMN_2225887	ATP5EP2	0,009665617	NA	NA	NA	NA	NA	NA	NA	NA	NA
ILMN_1749447	RPS9	0,009704473	NA	NA	NA	NA	NA	NA	NA	NA	NA

ILMN_1798588	HLTF	0,009706297	NA	NA	NA	NA	NA	NA	NA	NA	NA
ILMN_2091084	C8orf37	0,009718112	NA	NA	NA	NA	NA	NA	NA	NA	NA
ILMN_1671486	HOMER2	0,009737609	NA	NA	NA	NA	NA	NA	NA	NA	NA
ILMN_1748819	MRPL22	0,009838921	NA	NA	NA	NA	NA	NA	NA	NA	NA
ILMN_2408908	MAP4K5	0,009871778	NA	NA	NA	NA	NA	NA	NA	NA	NA
ILMN_2217809	TMEM126A	0,00987477	NA	NA	NA	NA	NA	NA	NA	NA	NA
ILMN_1800855	PPTC7	0,009919736	NA	NA	NA	NA	NA	NA	NA	NA	NA
ILMN_2053538	RHBDL2	0,009960945	NA	NA	NA	NA	NA	NA	NA	NA	NA
ILMN_3258321	LOC1001309	0,009993713	NA	NA	NA	NA	NA	NA	NA	NA	NA
