# Supplementary material for: Stratifying Cumulus Cell Samples Based on Molecular Profiling to Help Resolve Biomarker Discrepancies and to Predict Oocyte Developmental Competence
Source: Int J Mol Sci. 2021 Jun 15;22(12):6377. doi: 10.3390/ijms22126377 (PMC8232172; doi:10.3390/ijms22126377)
Supplement: Supplementary file 1 [file ijms-22-06377-s001.zip › Suppl Table 4 Patients treatment protocols summary.pdf]

Supplementary Table 4

Long protocol

		**Menst. Day**																					**1**	**2**	**3**	**4**	**5**	**6**	**7**	**8**	**9**	**10**	**11**	**12**	**13**	**14**
**VS1**	FINOX	recomb FSH (U)																					150	150	150	150	150	150	150	150	150	150	150	150	75	
	Decapeptil	Agonist (ng)							10	10	10	10	10	10	10	10	10	10	10	10	10	10	10	10	10	10	10	10	10	10	10	10	10	10	10	10
		**E2 pg/ml**																					**1**						**124**		**290**			**885**		**735**
		egg number																					**14**						**16**		**13**			**13**		**20**
		size																					**10**						**10**		**15**			**16**		**20**
		**Endom mm**																					**1,4**						**8,5**		**9,2**			**9,8**		
		**Menst. Day**																					**1**	**2**	**3**	**4**	**5**	**6**	**7**	**8**	**9**	**10**	**11**	**12**	**13**	
**VS2**	FINOX	recomb FSH																					150	150	150	150	150	150	150	150	150	150	150	150		
	Decapeptil	Agonist								10	10	10	10	10	10	10	10	10	10	10	10	10	10	10	10	10	10	10	10	10	10	10	10	10	10	
		**E2 pg/ml**																					**20**						**308**			**793**			**2721**	
		**Follicul number**																											16			17			16	
		**size**																																	20	
		**Endom mm**																											**8,7**			**9,2**			**idle**	
		**Menst. Day**																					**1**	**2**	**3**	**4**	**5**	**6**	**7**	**8**	**9**	**10**	**11**			
**VS4**	FINOX	recomb FSH																					150	150	150	150	150	150	150	150	150	150	150			
	Decapeptil	Agonist						10	10	10	10	10	10	10	10	10	10	10	10	10	10	10	10	10	10	10	10	10	10	10	10	10	10			
		**E2 pg/ml**																					**20**						**213**				**1578**			
		**Follicul number**																											16				16			
		**Size**																											11				18			
		**Endom mm**																											**9**				**14,1**			
		**Menst. Day**																					**1**	**2**	**3**	**4**	**5**	**6**	**7**	**8**	**9**	**10**	**11**	**12**	**13**	
**VS5**	FINOX	recomb FSH																					150	150	150	150	150	150	150	150	150	150	150	150		
	Decapeptil	Agonist						10	10	10	10	10	10	10	10	10	10	10	10	10	10	10	10	10	10	10	10	10	10	10	10	10	10	10	10	
		**E2 pg/ml**																					**22**						**262**				**915**		**2848**	
		**Follikel R**																											18				17		14	
		**Follikel L**																											13				18		19	
		**Endom mm**																											**8,5**				**9,3**		**9,7**	
		**Menst. Day**																					**1**	**2**	**3**	**4**	**5**	**6**	**7**	**8**	**9**	**10**	**11**	**12**	**13**	
**VS6**	FINOX	recomb FSH																					150	150	150	150	150	150	150	150	150	150	150	150		
	Decapeptil	Agonist						10	10	10	10	10	10	10	10	10	10	10	10	10	10	10	10	10	10	10	10	10	10	10	10	10	10	10	10	
		**E2 pg/ml**																					**20**						**108**			**420**			**1581**	
		**Follicul number**																											9			11			14	
		**size**																											10			15			19	
		**Endom mm**																					**3**						**5**			**10,2**			**10,8 (?)**	
		**Menst. Day**																					**1**	**2**	**3**	**4**	**5**	**6**	**7**	**8**	**9**	**10**	**11**			
**VS10**	Gonal F	recomb FSH																					112.5	112.5	112.5	112.5	112.5	112.5	112.5	112.5	112.5	112.5	37.5			
	Decapeptil	Agonist	10	10	10	10	10	10	10	10	10	10	10	10	10	10	10	10	10	10	10	10	10	10	10	10	10	10	10	10	10	10	10			
		**E2 pg/ml**																	**20**										**335**			**721**		**1421**			
		**Follicule numb**																	**10**										10			10		13			
		**Size**																	**10**										10			16		20			
		**Endom mm**																											**7**			**9**		**11**			
		**Menst. Days**																					**1**	**2**	**3**	**4**	**5**	**6**	**7**	**8**	**9**	**10**	**11**	**12**	**13**	**14**
**VS13**	Puregon	recomb FSH																					150	150	150	150	150	175	175	175	200	200	200	200	200	150
	Decapeptil	Agonist							10	10	10	10	10	10	10	10	10	10	10	10	10	10	10	10	10	10	10	10	10	10	10	10	10	10	10	10
		**E2 pg/ml**																					**10**					**34**			**134**			**405**		**683**
		**Follicule numb**																										9			10			10		7
		**Size**																										10			12			17		19
		**Endom mm**																										**4**			**7**			**9,2**		**12**

Short protocol

		**Menst. Days**	**2**	**3**	**4**	**5**	**6**	**7**	**8**	**9**	**10**	**11**	**12**	**13**
**VS8**	Gonal F	recomb FSH (U)	150	150	225	225	150	225	225	225	150			
	Orgalutran	Antagonist (ng)	250	250	250	250	250	250	250	250	250	250	250	250
		**E2 pg/ml**			**147**			**683**			**1613**			
		**Follicule numb**			7			8			7			
		**size**			10			15			22			
		**Endom mm**			**6**			**11**			**12**			
		**Menst. Days**	**2**	**3**	**4**	**5**	**6**	**7**	**8**	**9**	**10**	**11**	**12**	
**VS9**	Puregon	recomb FSH (U)	150	150	150	150	200	200	200	200	200	200		
	Cetrotide	Antagonist (ng)		250	250	250	250	250	250	250	250	250	250	
		**E2 pg/ml**					**245**			**540**		**1610**		
		**Follicule num**					10			12		14		
		**Follicule size**					13			16		21		
		**Endom mm**					**6,7**			**9,4**		**8**		
		**Menst. Days**	**2**	**3**	**4**	**5**	**6**	**7**	**8**	**9**	**10**	**11**	**12**	
**VS11**	Bravelle	recomb FSH (U)	150	150	150	150	225	225	225	225	225	225	225	
	Orgalutran	Antagonist (ng)					250	250	250	250	250	250	250	
		**E2 pg/ml**					**297**			**541**			**1447**	
		**Follikel Num**					10			8			10	
		**Follikel size**					13			19			25	
		**Endom mm**					**6,7**			**9,5**			**10**	
