# Supplementary material for: Stratifying Cumulus Cell Samples Based on Molecular Profiling to Help Resolve Biomarker Discrepancies and to Predict Oocyte Developmental Competence
Source: Int J Mol Sci. 2021 Jun 15;22(12):6377. doi: 10.3390/ijms22126377 (PMC8232172; doi:10.3390/ijms22126377)
Supplement: Supplementary file 1 [file ijms-22-06377-s001.zip › Suppl Table 5 Clincal parameters.pdf]

Supplementary Table 5

Gender	AgeMat	Donor #	DonNum	EggNum	SampleName	Spindle	Zona	Pronuclei #	Pronuclei Pattern	Estrogen at d12	Endo size at d12	Lead Foll Size at d12	Slope Estrogen	Slope Endom size	Slope Foll size	Protocol	ChemPreg
Female	30	1	VS1	4A	VS1-4A	Pos	-2,8	2VK	AS	2,9542	0,99376	17,13793	0,16998	0,01211	1,25862	Long	No
Female	30	1	VS1	7A	VS1-7A	Neg	0,2	2VK	S	2,9542	0,99376	17,13793	0,16998	0,01211	1,25862	Long	No
Female	30	1	VS1	10A	VS1-10A	Pos	12,9	2VK	AS	2,9542	0,99376	17,13793	0,16998	0,01211	1,25862	Long	No
Female	30	1	VS1	12A	VS1-12A	Pos	-9,2	2VK	AS	2,9542	0,99376	17,13793	0,16998	0,01211	1,25862	Long	No
Female	34	8	VS10	4A	VS10-4A	Neg	5,4	2VK	AS	3,25973	1,03799	22	0,12436	0,03903	2	Long	No
Female	34	8	VS10	5A	VS10-5A	Neg	1,6	2VK	AS	3,25973	1,03799	22	0,12436	0,03903	2	Long	No
Female	32	9	VS11	1A	VS11-1A	Neg	28,7	2VK	AS	3,13266	1,02156	25	0,11462	0,02899	2	Short	No
Female	32	9	VS11	5A	VS11-5A	Neg	-0,7	2VK	AS	3,13266	1,02156	25	0,11462	0,02899	2	Short	No
Female	35	11	VS13	4A	VS13-4A	Pos	-6,2	2VK	S	2,56199	0,97341	16,57143	0,16393	0,05764	1,18367	Long	No
Female	35	11	VS13	5A	VS13-5A	Neg	-4	2VK	S	2,56199	0,97341	16,57143	0,16393	0,05764	1,18367	Long	No
Female	29	2	VS2	1A	VS2-1A	Neg	-8,3	2VK	S	3,25624	0,97997	19,89	0,1577	0,00808953	1,55	Long	Yes
Female	29	2	VS2	2A	VS2-2A	Neg	-5,4	2VK	AS	3,25624	0,97997	19,89	0,1577	0,00808953	1,55	Long	Yes
Female	35	4	VS4	4A	VS4-4A	Neg	1,1	2VK	AS	3,41554	1,19796	19,75	0,21743	0,04874	1,75	Long	No
Female	35	4	VS4	8A	VS4-8A	Neg	-0,1	2VK	AS	3,41554	1,19796	19,75	0,21743	0,04874	1,75	Long	No
Female	29	2	VS5	5A	VS5-5A	Pos	12,9	2VK	AS	3,22381	0,97754	18,39286	0,16743	0,0095884	1,03571	Long	No
Female	29	2	VS5	8A	VS5-8A	Neg	17,3	2VK	AS	3,22381	0,97754	18,39286	0,16743	0,0095884	1,03571	Long	No
Female	30	1	VS6	1A	VS6-1A	Pos	-10,3	2VK	AS	3,00704	1,02515	17,66667	0,19425	0,05574	1,5	Long	No
Female	30	1	VS6	5A	VS6-5A	Neg	-8,9	2VK	AS	3,00704	1,02515	17,66667	0,19425	0,05574	1,5	Long	No
Female	30	1	VS6	7A	VS6-7A	Neg	-9,1	2VK	AS	3,00704	1,02515	17,66667	0,19425	0,05574	1,5	Long	No
Female	30	1	VS6	9A	VS6-9A	Neg	-6	2VK	AS	3,00704	1,02515	17,66667	0,19425	0,05574	1,5	Long	No
Female	34	6	VS8	2A	VS8-2A	Neg	10,6	2VK	AS	3,77677	1,26727	25,66667	0,17339	0,05017	2	Short	No
Female	34	6	VS8	4A	VS8-4A	Neg	-4,5	2VK	AS	3,77677	1,26727	25,66667	0,17339	0,05017	2	Short	No
Female	39	7	VS9	3A	VS9-3A	Neg	11,4	2VK	S	3,30831	0,96095	21,84211	0,15965	0,01806	1,55263	Short	No
Female	39	7	VS9	5A	VS9-5A	Pos	15,6	2VK	AS	3,30831	0,96095	21,84211	0,15965	0,01806	1,55263	Short	No
