# Supplementary material for: Stratifying Cumulus Cell Samples Based on Molecular Profiling to Help Resolve Biomarker Discrepancies and to Predict Oocyte Developmental Competence
Source: Int J Mol Sci. 2021 Jun 15;22(12):6377. doi: 10.3390/ijms22126377 (PMC8232172; doi:10.3390/ijms22126377)
Supplement: Supplementary file 1 [file ijms-22-06377-s001.zip › Suppl. Fig 1 ICSI Basic Clinical parameters Kopieb Kopie.pdf]

Supplementary Figure 1

A)

Donor ID	Treatment ID	Treatment Protocol	Transfered samples	Additional Sample	Pregnancy test	Pregnancy Result	age F	age M	Male inf. severity vol	conc	mor	Mot %	Notes
1	VS1	Long	4 and 7	10 and 12	Pos.	No Cont Preg	30	38	3	1		10	Same as VS6
1	VS6	Long	1 and 5	7 and 9	Pos.	Pregnant	30	38	3,4	1,6	<5	19	Same as VS1
2	VS2	Long	1 and 2		Pos.	No Cont Preg	29	30	3	6	<5	48	Same as VS5
2	VS5	Long	5 and 8		Neg.		29	30	2	13	<5	50	Same as VS2
4	VS4	Long	4 and 8		Pos.	Pregnant	35	39	1,7	31	<3	40	
6	VS8	Short	2 and 4		Neg.		34	38	5	36	<5	34	
7	VS9	Short	3 and 5		Neg.		40	50	0,7	21	<4	43	kryosperm
8	VS10	Long	4 and 5		Pos.	Pregnant	35	34	4,4	32	<5	55	
9	VS11	Short	1 and 5		Neg.		33	38	0,6	4	<4	5	kryosperm
11	VS13	Long	4 and 5		Neg.		36	38	1,5	14	<3	25	

B)

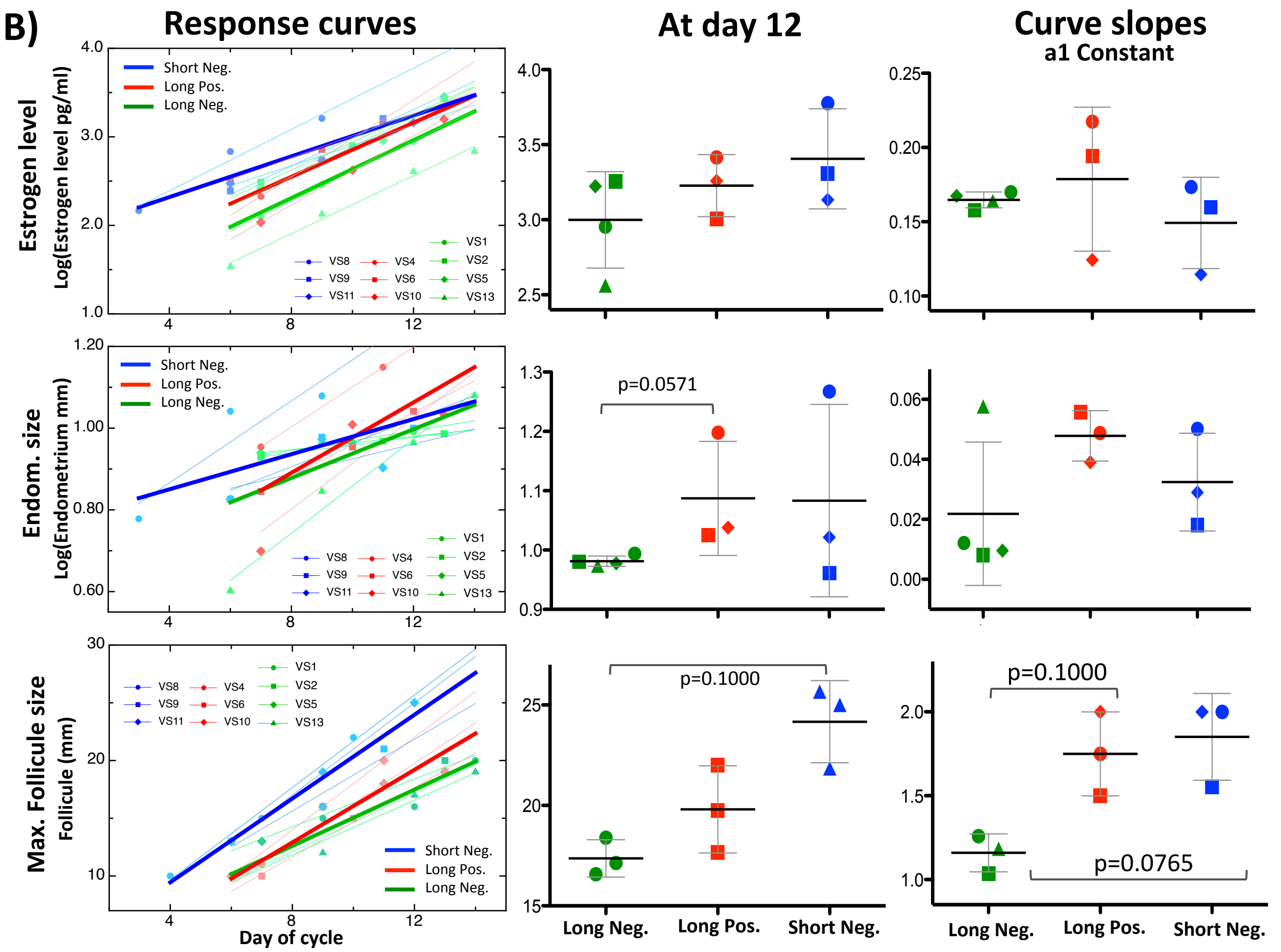

C)

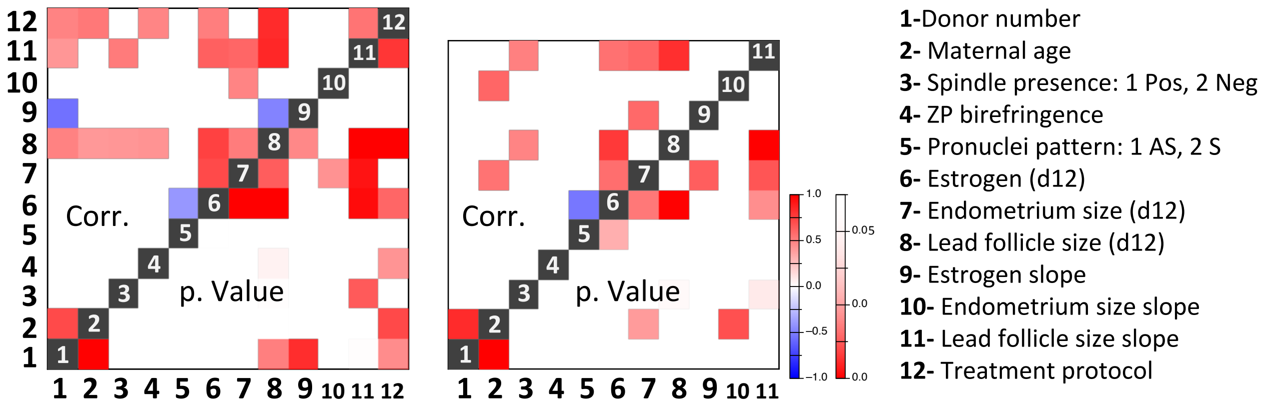

Suppl. Figure 1: Clinical characteristics of the collected samples. A) Details characteristics of the donated samples and the male fertility parameters. B) The linear fitted response curves (per patient/treatment) for estrogen levels, endometrium thickness and maximum follicular size for each treatment are shown as dashed lines at the left column. A representative average representing curve for each of the three categories is drawn as a thick non-dashed line. The middle column shows the measured or the curve-based extrapolated values for each treatment of the three parameters at day 12. The right column shows the slopes of the fitted linear curves corresponding to each treatment. Blue, red and green correspond to short-negative, long-positive and long-negative protocols, respectively. C) Correlation between collected clinical parameters.
