# Supplementary material for: Stratifying Cumulus Cell Samples Based on Molecular Profiling to Help Resolve Biomarker Discrepancies and to Predict Oocyte Developmental Competence
Source: Int J Mol Sci. 2021 Jun 15;22(12):6377. doi: 10.3390/ijms22126377 (PMC8232172; doi:10.3390/ijms22126377)

Supplementary Figure 2

A) All data

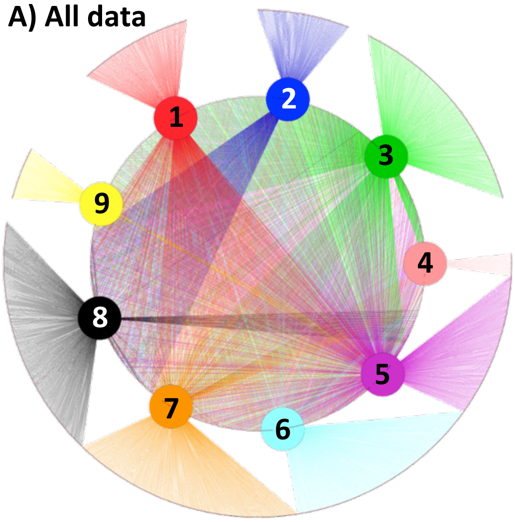

Parameter	probes
1 Estrogen (d12)	490
2 Endometrium size (d12)	304
3 Lead follicle size (d12)	1119
4 ZP birefringence	440
5 Lead follice size slope	790
6 Spindle presence	48
7 Estrogen slope	554
8 Endometrium size slope	421
9 Pronuclei pattern	109

Intersection

	1	2	3	4	5	6	7	8	9
1									
2	78								
3	270	80							
4	20	1	113						
5	132	60	529	51					
6	4	0	8	2	9				
7	10	19	145	57	89	0			
8	2	29	4	8	23	2	17		
9	22	3	21	2	18	1	2	0	

Top 10 correlated genes:

Estrogen (d12)		Endom. Size (d12)		Lead Follicle Size (d12)		ZP birefrengence		Lead Follicle Size Slope		Spindle Presence		Estrogen slope		Endom. Size Slope		Pronuceli pattern	
Gene	p value	Gene	p value	Gene	p value	Gene	p value	Gene	p value	Gene	p value	Gene	p value	Gene	p value	Gene	p value
SNHG8	1,46E-08	SNHG8	1,46E-08	SNHG8	1,46E-08	GFPT1	2,83E-06	PPP3CC	7,15E-06	ZNF200	6,64E-04	BBS2	9,52E+10	GTF2IRD1	6,29E+10	FUT4	0,0003406
GSTT1	1,89E-07	ZNF439	7,32E-07	GSTT1	1,89E-07	C15orf38	2,07E-05	GSTT1	1,03E-05	ACYP1	1,57E-03	HES6	9,97E+10	ADI1	0.00028	FAM150B	0,000375
MVP	6,98E-07	SAPS3	2,85E-06	MVP	6,98E-07	MXRA7	2,98E-05	PTPRE	1,34E-05	NUDT22	1,74E-03	ERRFI1	5,18E+11	STAT1	0.00067	CLCNKA	0,000382
ZNF439	7,32E-07	SLK	1,13E-05	ZNF439	7,32E-07	CHD7	3,79E-05	FNDC3A	1,41E-05	TNKS1BP1	2,00E-03	HSDL2	0.00010	FAM53B	0.00075	LOC285016	0,0004802
MVP	2,09E-06	FAM119B	1,88E-05	MVP	2,09E-06	MAPRE2	6,27E-05	MVP	1,91E-05	LOC651894	2,11E-03	SOCS5	0.00011	NR2C2AP	0.00087	LOC100133627	0,0004883
SAPS3	2,85E-06	LAMA2	4,38E-05	SAPS3	2,85E-06	TMEM176A	6,40E-05	PTPRE	1,94E-05	TRIM37	2,21E-03	MID1	0.00019	EXOSC6	0.00097	DSCR3	0,0005625
RIT1	3,41E-06	SUCLA2	6,74E-05	RIT1	3,41E-06	LOC100130511	6,66E-05	SCML1	2,36E-05	SFRP1	2,23E-03	ALDH1A2	0.00020	ADSS	0.00133	BTBD3	0,0005909
FAM44B	3,60E-06	MOCOS	7,87E-05	FAM44B	3,60E-06	DCTN2	7,72E-05	CLDN12	2,54E-05	TMEM165	2,33E-03	RGS7	0.00025	C6orf85	0.00170	LOC402560	0,0006322
MED4	3,77E-06	DEPDC5	9,89E-05	MED4	3,77E-06	GAA	8,68E-05	TMEM51	3,76E-05		2,76E-03	FAM188B	0.00029	A4GALT	0.00263	CLCNKA	0,0006468
SCML1	4,44E-06	NT5DC1	1,12E-04	SCML1	4,44E-06	FAM108C1	1,11E-04	PACSIN2	3,77E-05	PBX1	3,27E-03	NSFL1C	0.00038	RICS	0.00299	TRAF6	0,0007674

Top enriched biological function in the correlated probes:

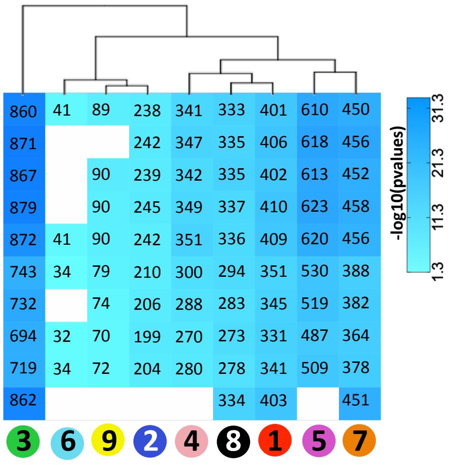

	3	6	9	2	4	8	1	5	7
Carcinoma	860	41	89	238	341	333	401	610	450
Nonhematologic malignant neoplasm	871			242	347	335	406	618	456
Non-melanoma solid tumor	867		90	239	342	335	402	613	452
Malignant solid tumor	879		90	245	349	337	410	623	458
Extracranial solid tumor	872	41	90	242	351	336	409	620	456
Cancer of secretory structure	743	34	79	210	300	294	351	530	388
Head and neck tumor	732		74	206	288	283	345	519	382
Thyroid carcinoma	694	32	70	199	270	273	331	487	364
Head and neck carcinoma	719	34	72	204	280	278	341	509	378
Tumorigenesis of tissue	862					334	403		451

Suppl. Figure 2: Correlation between clinical parameters and the expression data when considering A) all data of long and short protocol or B) long protocol only. Upper panel: The left diagram display the overlaps in genes set correlated with the clinical parameters. The middle panel shows the number of probes positively correlated at 0.001 with each of the clinical parameters. The right triangle shows the number of overlapping probes between two clinical parameters. **Middle panel:** The top 10 correlated genes are listed for each clinical parameter. **Lower panel:** The top 10 enriched biological function in correlated probes (number of probes in each category is shown on the heat map).

Supplementary Figure 2B

B) Long protocol only

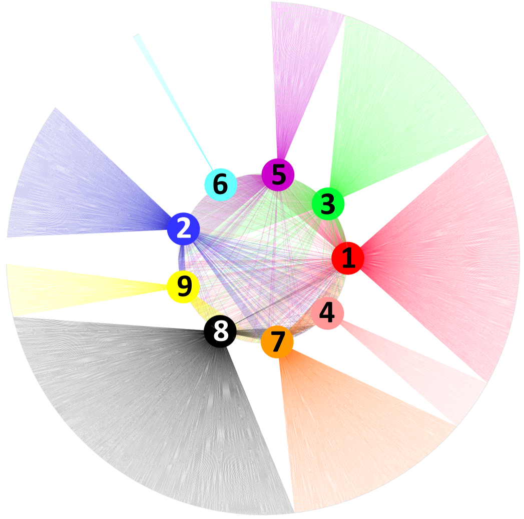

Parameter	probes	Intersection								
1 Estrogen (d12)	847	1								
2 Endometrium size (d12)	415	38	2							
3 Lead follicle size (d12)	781	233	17	3						
4 ZP birefringence	130	11	0	10	4					
5 Lead follice size slope	343	32	31	165	2	5				
6 Spindle presence	16	1	0	1	0	1	6			
7 Estrogen slope	443	0	51	28	1	3	0	7		
8 Endometrium size slope	783	22	30	0	9	6	0	6	8	
9 Pronuclei pattern	224	92	9	48	2	9	0	0	0	9

Top 10 correlated genes:

Estrogen (d12)		Endom. Size (d12)		Lead Follicle Size (d12)		ZP birefrengence		Lead Follicle Size Slope	
Gene	p value	Gene	p value	Gene	p value	Gene	p value	Gene	p value
TTC19	6,04E-06	DDRGK1	1,21E-06	AZIN1	4,63E-06	UBE2D2	5,52E-05	YTHDF2	6,94E-07
SESN1	7,81E-06	NPEPL1	2,20E-06		6,03E-06	HSCB	8,52E-05	CBX1	8,16E-06
C2orf79	1,42E-05		3,68E-06	MGC16169	8,58E-06	CLP1	1,67E-04	LOC728779	1,27E-05
PVRL2	2,11E-05	PAPD1	2,57E-05	GCNT3	8,72E-06	FZD7	2,49E-04	OC10013130	5,63E-05
LOC344595	2,17E-05	GTF3C6	2,65E-05	OC10012988	2,32E-05	NARS	2,51E-04	PDGFD	1,00E-04
TNS3	2,51E-05	OSBP2	2,76E-05	C2orf25	2,39E-05		2,63E-04	LOC645251	1,16E-04
PDE5A	2,58E-05	AGPAT2	4,37E-05	NSFL1C	2,51E-05	SYNCRIP	3,79E-04	OSBPL11	1,19E-04
CLCNKA	3,30E-05	HLA-C	4,43E-05	KCND2	4,71E-05	ZNF331	4,01E-04	LOC728060	1,43E-04
FLVCR2	3,79E-05	LOC653513	4,67E-05	FAM14B	5,16E-05	C9orf103	4,39E-04	AGPAT9	1,56E-04
USP6	4,01E-05	NFIB	5,32E-05	NSFL1C	5,23E-05	NSL1	4,58E-04	FBLN1	2,43E-04

Spindle Presence		Estrogen slope		Endom. Size Slope		Pronuceli pattern	
Gene	p value	Gene	p value	Gene	p value	Gene	p value
MAN1A2	7,23E-04	LOC650832	2,15E-05	GREM1	1,69E-07	KIAA0174	5,16E-05
NIT1	1,55E-03	LOC641983	2,15E-05	SLC25A28	2,90E-06	VCAM1	3,24E-04
LIPT1	2,23E-03	G3BP1	2,67E-05	ELOVL1	3,15E-06	MTX2	3,48E-04
RRAS2	2,43E-03	CDC14B	3,13E-05	WAC	3,90E-06	LOC728006	3,52E-04
DEPDC5	3,31E-03	ABHD12	3,15E-05	DPYD	5,88E-06	IFNAR2	3,63E-04
PAFAH1B2	5,18E-03	MAP2K3	3,34E-05	ALDH18A1	7,41E-06	SLC44A1	3,68E-04
TRIM37	5,58E-03	GSTM2	3,89E-05	WDR59	9,12E-06	LOC646214	3,79E-04
CD151	5,67E-03	CDC14B	4,10E-05	PKP2	9,59E-06	TBC1D23	4,26E-04
C9orf102	6,72E-03	TRADD	4,28E-05	ABAT	1,33E-05	MAPK1	5,23E-04
ADAM17	7,11E-03	PELI2	4,43E-05	CASC4	1,64E-05	CECR1	5,67E-04

Top enriched biological function in the correlated probes:

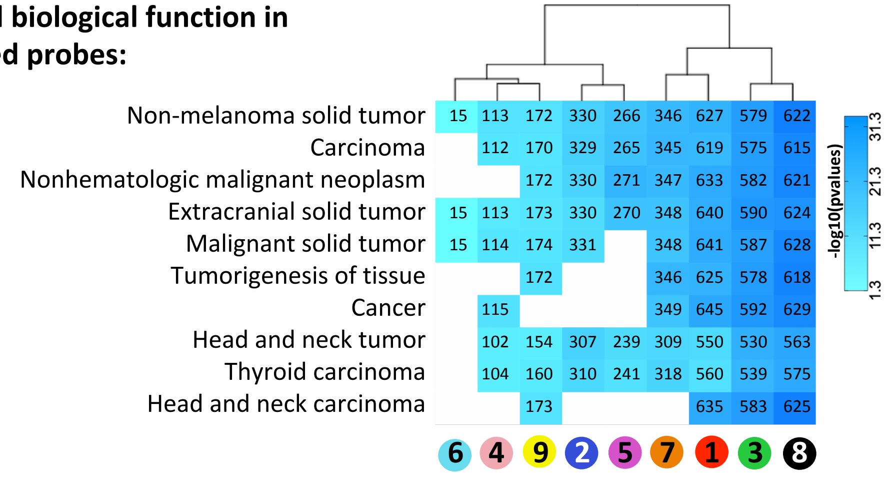

Supplement: Supplementary file 1 [file ijms-22-06377-s001.zip › Suppl. Fig 2 Expression correlation with clinical parameters all samples V2.pdf]
