# Supplementary material for: Stratifying Cumulus Cell Samples Based on Molecular Profiling to Help Resolve Biomarker Discrepancies and to Predict Oocyte Developmental Competence
Source: Int J Mol Sci. 2021 Jun 15;22(12):6377. doi: 10.3390/ijms22126377 (PMC8232172; doi:10.3390/ijms22126377)
Supplement: Supplementary file 1 [file ijms-22-06377-s001.zip › Suppl. Fig 3 Methy correlation with clinical parameters long protocol only.pdf]

Supplementary Figure 3

A) All data

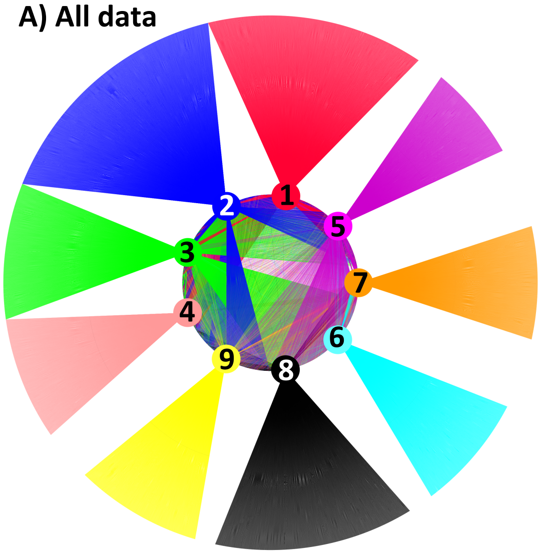

	Parameter	probes
1	Estrogen (d12)	9283
2	Endometrium size (d12)	9291
3	Lead follicle size (d12)	7492
4	ZP birefringence	3009
5	Lead follice size slope	5247
6	Spindle presence	2988
7	Estrogen slope	3061
8	Endometrium size slope	5062
9	Pronuclei pattern	3413

Intersection

1								
2427	2							
2350	1078	3						
41	4	147	4					
928	838	2411	11	5				
134	76	144	3	232	6			
23	229	207	71	86	4	7		
14	428	21	53	140	9	70	8	
392	145	167	16	166	4	1	1	9

Estrogen (d12)		End. size (d12)		Lead foll. size (d12)		ZP birefringence		Lead foll. size slope	
cg numb.	p Value	cg numb.	p Value	cg numb.	p Value	cg numb.	p Value	cg numb.	p Value
cg01081438	1,00E-04	cg12244647	1,00E-04	cg11574174	1,00E-04	cg15006101	1,06E-04	cg02143936	1,01E-04
cg03879460	1,00E-04	cg16145187	1,01E-04	cg04117764	1,01E-04	cg16271912	1,06E-04	cg07313705	1,08E-04
cg26679884	1,01E-04	cg06550274	1,01E-04	cg25691775	1,01E-04	cg10374523	1,08E-04	cg16412745	1,08E-04
cg05870739	1,01E-04	cg01577114	1,02E-04	cg01836137	1,02E-04	cg19363579	1,16E-04	cg17421062	1,08E-04
cg00206463	1,02E-04	cg18676273	1,03E-04	cg04834502	1,02E-04	cg02929310	1,19E-04	cg08790807	1,10E-04
cg25705508	1,03E-04	cg22576055	1,04E-04	cg13127920	1,04E-04	cg15819808	1,29E-04	cg09633081	1,11E-04
cg26205751	1,03E-04	cg21340093	1,04E-04	cg00039864	1,04E-04	cg04414683	1,30E-04	cg17054045	1,11E-04
cg17965926	1,05E-04	cg01874081	1,05E-04	cg23793213	1,04E-04	cg12873262	1,41E-04	cg23950724	1,12E-04
cg00875849	1,05E-04	cg13562284	1,05E-04	cg22865908	1,04E-04	cg04289130	1,43E-04	cg08319019	1,13E-04
cg08691159	1,05E-04	cg15227231	1,05E-04	cg21240736	1,05E-04	cg11260046	1,46E-04	cg24357503	1,13E-04

Spindle presence		Estrogen slope		Endo. Size slope		Pronuclei pattern	
cg numb.	p Value	cg numb.	p Value	cg numb.	p Value	cg numb.	p Value
cg12398870	1,03E-04	cg12339131	1,08E-04	cg02567750	1,02E-04	cg03663984	1,00E-04
cg08021564	1,08E-04	cg05487769	1,12E-04	cg04792777	1,07E-04	cg26555401	1,01E-04
cg02406084	1,19E-04	cg13150801	1,13E-04	cg14495443	1,09E-04	cg12290635	1,01E-04
cg14143519	1,36E-04	cg11379315	1,20E-04	cg25822881	1,09E-04	cg24671229	1,02E-04
cg05192145	1,42E-04	cg11177059	1,21E-04	cg22185268	1,15E-04	cg12286540	1,19E-04
cg16702014	1,56E-04	cg13661648	1,22E-04	cg12937413	1,17E-04	cg26276327	1,23E-04
cg23167594	1,69E-04	cg26167010	1,23E-04	cg01410801	1,19E-04	cg21544658	1,30E-04
cg05109266	1,72E-04	cg09230938	1,24E-04	cg19490678	1,19E-04	cg00647106	1,49E-04
cg16279748	1,78E-04	cg06792126	1,26E-04	cg12932539	1,21E-04	cg04324615	1,53E-04
cg21004129	1,93E-04	cg01931028	1,29E-04	cg08880817	1,21E-04	cg25757149	1,54E-04

B) Long protocol only

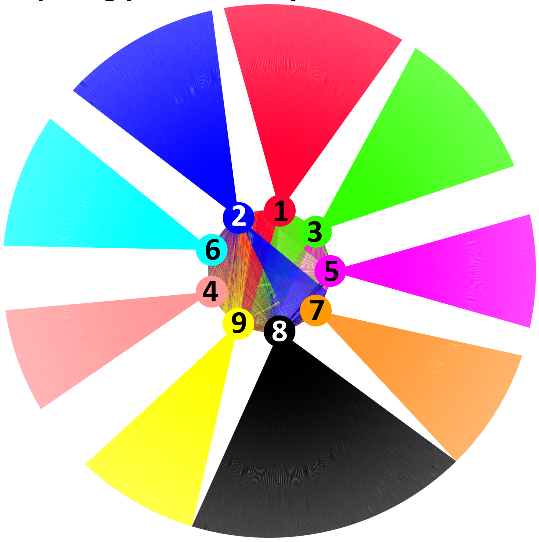

	Parameter	probes
1	Estrogen (d12)	7538
2	Endometrium size (d12)	5639
3	Lead follicle size (d12)	7695
4	ZP birefringence	2750
5	Lead follice size slope	5319
6	Spindle presence	3648
7	Estrogen slope	3962
8	Endometrium size slope	7421
9	Pronuclei pattern	3896

Intersection

1								
396	2							
1887	177	3						
57	0	12	4					
383	473	2026	6	5				
70	19	154	0	122	6			
3	634	199	3	46	0	7		
115	159	6	58	31	0	35	8	
724	56	166	26	39	0	4	0	9

Estrogen (d12)		Endometrium size (d12)		Lead foll. size (d12)		ZP birefringence		Lead foll. size slope	
cg numb.	p Value	cg numb.	p Value	cg numb.	p Value	cg numb.	p Value	cg numb.	p Value
cg23489384	2,14E-10	cg13184710	6,10E-10	cg24826564	9,28E-08	cg05017628	3,09E-06	cg17466245	3,96E-07
cg13979581	6,03E-09	cg11666534	6,23E-09	cg09404119	2,93E-06	cg04923496	6,08E-06	cg03253629	3,00E-06
cg15469871	7,25E-09	cg01872988	1,08E-08	cg00555420	3,19E-06	cg14265502	1,06E-05	cg12085660	5,84E-06
cg03075889	1,00E-08	cg17220237	1,20E-08	cg02226252	3,52E-06	cg06350432	1,37E-05	cg16322581	6,03E-06
cg04227238	1,46E-08	cg09289202	1,40E-08	cg24453349	4,11E-06	cg14725392	1,50E-05	cg13869341	6,78E-06
cg13824456	2,02E-08	cg08293392	1,68E-08	cg15312203	5,41E-06	cg20445967	2,33E-05	cg07222421	1,05E-05
cg20536971	3,01E-08	cg19021236	3,07E-08	cg02366642	5,98E-06	cg03286774	2,39E-05	cg18881641	1,09E-05
cg10295955	1,20E-07	cg21467828	3,67E-08	cg24770292	6,91E-06	cg16458170	2,50E-05	cg27286337	1,17E-05
cg10879348	3,39E-07	cg03279219	5,13E-08	cg00813440	7,82E-06	cg02172312	2,74E-05	cg08060578	1,52E-05
cg23636571	6,66E-07	cg10759817	5,38E-08	cg23134838	9,51E-06	cg09845000	2,77E-05	cg05101920	2,03E-05

Spindle presence		Estrogen slope		Endo. Size slope		Pronuclei pattern	
cg numb.	p Value	cg numb.	p Value	cg numb.	p Value	cg numb.	p Value
cg12829747	2,22E-06	cg08198851	2,98E-07	cg02611183	3,92E-07	cg27579097	5,41E-07
cg13454226	6,36E-06	cg23970189	1,91E-06	cg16757384	9,11E-07	cg19519630	2,32E-06
cg14843143	9,38E-06	cg02275035	2,31E-06	cg18446336	1,41E-06	cg07270289	3,31E-06
cg17900495	1,22E-05	cg02614045	2,58E-06	cg11340603	2,03E-06	cg21544658	5,14E-06
cg16513905	1,82E-05	cg01527307	4,05E-06	cg00946479	2,40E-06	cg27573975	6,61E-06
cg19821361	1,95E-05	cg26499095	4,39E-06	cg07207937	2,75E-06	cg07107453	7,43E-06
cg09730697	2,16E-05	cg26161820	4,91E-06	cg07672051	3,90E-06	cg20342460	8,60E-06
cg19983801	2,35E-05	cg22836207	5,17E-06	cg06836319	4,31E-06	cg16841748	1,21E-05
cg05425877	2,42E-05	cg00998146	5,87E-06	cg11478766	5,15E-06	cg09254001	1,24E-05
cg26449450	2,48E-05	cg18992293	6,60E-06	cg13667243	5,37E-06	cg17757000	1,41E-05

Suppl. Figure 4 : Correlation between clinical parameters and the methylation data when considering A) all data or B) only long protocol. The left diagram display the overlaps in genes set correlated with the clinical parameters. The middle panel shows the number of probes positively correlated at 0.001 with each of the clinical parameters. The right triangle shows the number of overlapping probes between two clinical parameters. The top 10 correlated cgs are listed for each clinical parameter.
