# Supplementary material for: Stratifying Cumulus Cell Samples Based on Molecular Profiling to Help Resolve Biomarker Discrepancies and to Predict Oocyte Developmental Competence
Source: Int J Mol Sci. 2021 Jun 15;22(12):6377. doi: 10.3390/ijms22126377 (PMC8232172; doi:10.3390/ijms22126377)
Supplement: Supplementary file 1 [file ijms-22-06377-s001.zip › Suppl. Fig 5 treatment protocol differences Long Vs Short Ver4.pdf]

Supplementary Figure 5

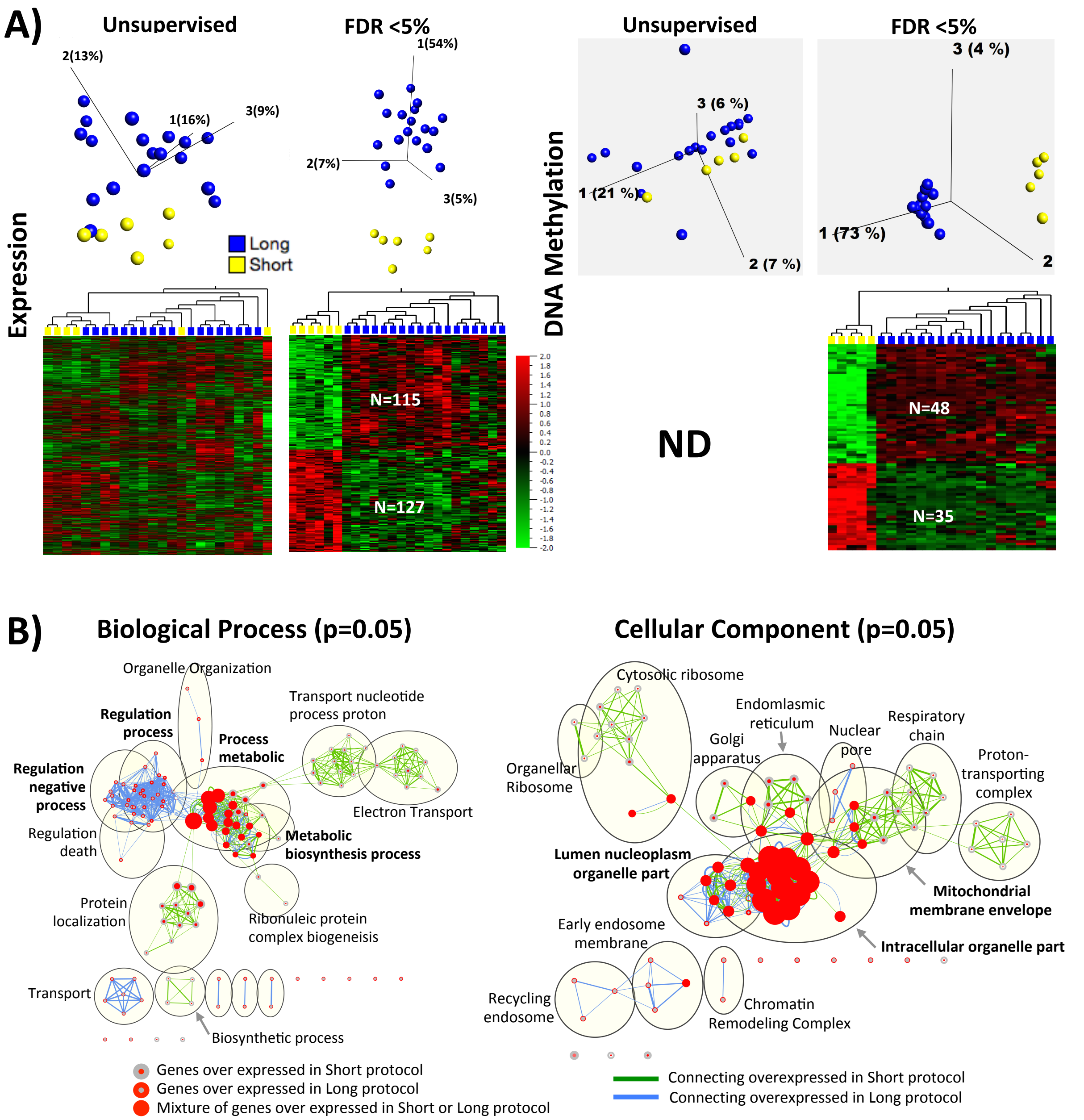

Suppl. Figure 5: Differences in expression and CpG methylation profiles between long and short protocols. **A)** 3D-PCA plots and heatmaps of expression and CpGs methylation profiling. Result of unsupervised and significance at FDR <5% are shown. **B)** General ontology analysis of the genes with difference in expression at p=0.05 for both biological process and cellular component.

Supplementary Figure 5 (Continued)

C)

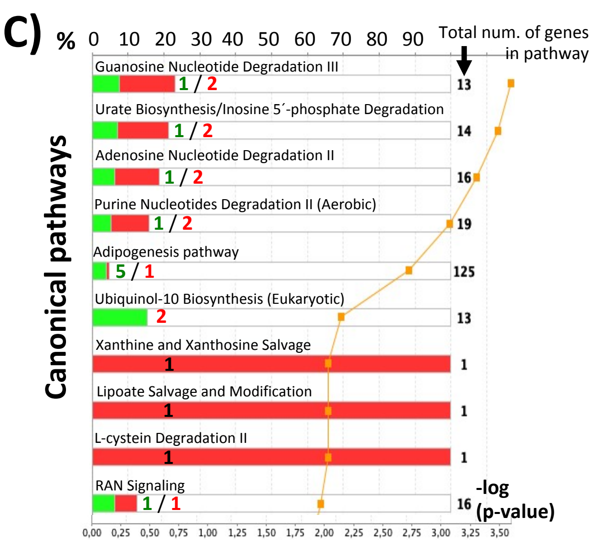

D)

Upstream regulator

Upstream regulator	Activation Z-Score	P-value of overlap	Number of regulated genes in data
HAND2	1,067	8,57E-04	4
TBX5	1,067	1,13E-03	4
MYOCD	1,067	3,22E-03	4
miR-29b-3p		4,75E-03	3
TRPS1		5,87E-03	2
TCF		8,53E-03	4
GPR174		1,07E-02	1
NRG (family)		1,07E-02	1
AHCTF1		1,07E-02	1

E) Disease and Function

Sized by Z-Score; colored by Z-Score

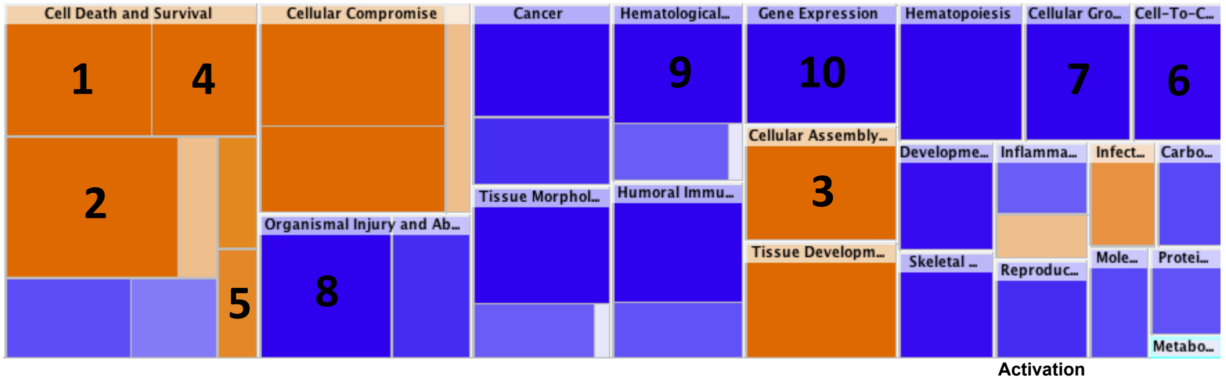

	Categories	Function	Diseases or Functions Annotati	p-value	Activation z-score	# Molecules
1	Cell Death and Survival,Cellular Compromise	cytotoxicity	cytotoxicity of cytotoxic T cells	4,05E-04	1,982	4 / 44
2	Cell Death and Survival	apoptosis	apoptosis of pancreatic cancer cell lines	3,49E-02	1,974	4 / 287
3	Cellular Assembly and Organization,Tissue Development	fibrogenesis	fibrogenesis	3,88E-02	1,890	9 / 915
4	Cell Death and Survival,Cellular Compromise	cytotoxicity	cytotoxicity of lymphocytes	2,04E-02	1,432	5 / 244
5	Cell Death and Survival	cytolysis	cytolysis	1,20E-02	1,000	8 / 581
6	Cell-To-Cell Signaling and Interaction	adhesion	adhesion of tumor cell lines	2,74E-02	-1,227	8 / 614
7	Cellular Growth and Proliferation	proliferation	proliferation of cells	1,20E-02	-1,444	64 / 8613
8	Cancer,Organismal Injury and Abnormalities	advanced malignancy	advanced malignant tumor	4,25E-02	-1,647	17 / 2732
9	Hematological System Development and Function, Hematopoiesis,Humoral Immune Response,Tissue Morphology	quantity	quantity of pre-B lymphocytes	3,13E-02	-1,697	4 / 158
10	Gene Expression	transactivation	transactivation of RNA	8,17E-03	-1,984	13 / 992

Supplementary Figure 5 (continued): IPA analysis (at FDR <5%) showing top ten affected **C)** canonical pathway, **D)** upstream regulators and **E)** diseases and biofunction.
