# Supplementary material for: Stratifying Cumulus Cell Samples Based on Molecular Profiling to Help Resolve Biomarker Discrepancies and to Predict Oocyte Developmental Competence
Source: Int J Mol Sci. 2021 Jun 15;22(12):6377. doi: 10.3390/ijms22126377 (PMC8232172; doi:10.3390/ijms22126377)
Supplement: Supplementary file 1 [file ijms-22-06377-s001.zip › Suppl. Fig 6 Overlaps between Correlated probes with clinical parameters and Pos vs. Neg in long protocol V2.pdf]

Supplementary Figure 6

Clinical Parameter	Total Correlated probes	% in correlated probes	Overlaps with diff expressed probes Pos vs. Neg in long protocol	% in Diff Expressed probes Pos. vs. Neg.
	Presented in details in Suppl. Fig 3		Presented below in this figure	
Estrogen (d12)	847	9.4%	80	5.1%
Endom. size (d12)	415	49.9%	207	13.3%
Lead follicle size (d12)	781	22.2%	173	11.1%
ZP birefringence	130	2.3%	3	0.2%
Spindle presence	16	12.5%	2	0.1%
Pronuclei pattern	224	33.9%	76	4.9%
Estrogen slope	443	2.9%	13	0.8%
Endom. size slope	783	27.8%	218	14%
Lead follicle size slope	343	70%	240	15.4%

First 10 Overlaps with the diff. expressed between positive and negative

Estrogen d12

		Estrogen d12		Data in Long Neg vs Pos	
PROBE_ID	Gene	p0.05	Rank	p0.05	Rank
ILMN_2051731	DUSP5P	8,59E-03	770	2,36E-03	164
ILMN_1709044	TGIF2	7,71E-04	152	2,95E-03	188
ILMN_1792689	HIST1H2AC	1,85E-03	259	3,10E-03	198
ILMN_1759154	PABPN1	4,74E-03	518	3,18E-03	202
ILMN_2129927	EXT1	3,91E-03	445	3,35E-03	207
ILMN_2087060	TOMM7	3,65E-03	421	4,15E-03	240
ILMN_2356311	C21orf51	4,96E-03	536	4,24E-03	245
ILMN_3238680	C7orf55	3,36E-03	397	4,68E-03	267
ILMN_2115991	TNNT1	7,39E-03	684	4,98E-03	278
ILMN_2211122	TRAPPC2P1	1,27E-03	211	5,12E-03	289

Endometrium d12

		Endometrium Size (d12)		Data in Long Neg vs Pos	
PROBE_ID	SYMBOL	p0.05	Rank	p0.05	Rank
ILMN_3245057	**ASAP1**	2,33E-03	133	1,94E-06	1
ILMN_1690963	**ASAP1**	6,41E-03	304	2,87E-06	2
ILMN_2229032	**NME6**	2,71E-03	154	3,84E-06	3
ILMN_2098418	LOC652968	1,12E-03	75	7,19E-05	8
ILMN_1685580	CBLB	5,11E-03	250	7,75E-05	9
ILMN_1794885	FRMD5	3,35E-03	185	9,71E-05	15
ILMN_1703228	AGFG2	5,65E-03	269	1,64E-04	23
ILMN_1797298	ARMC7	2,60E-03	147	2,22E-04	30
ILMN_3238452	FAM21D	6,12E-03	292	2,40E-04	31
ILMN_1778991	NFIB	5,32E-05	10	2,41E-04	32

ZP

		ZP birefringence		Data in Long Neg vs Pos	
PROBE_ID	SYMBOL	p0.05	Rank	p0.05	Rank
ILMN_1669617	GRB10	8,27E-03	109	2,60E-02	939
ILMN_2043845	IMPACT	2,90E-03	35	3,39E-02	1139
ILMN_2089175	SYAP1	5,46E-03	76	4,08E-02	1315

Spindle

		Spindle Presence		Data in Long Neg vs Pos	
PROBE_ID	SYMBOL	p0.05	Rank	p0.05	Rank
ILMN_1765779	ADAM17	7,11E-03	10	4,99E-03	280
ILMN_1743319	PAFAH1B2	5,18E-03	6	2,34E-02	883

Lead Follicle d12

		Lead Follice Size (d12)		Data in Long Neg vs Pos	
PROBE_ID	SYMBOL	p0.05	Rank	p0.05	Rank
ILMN_1767068	SMAD6	9,35E-03	741	1,00E-04	16
ILMN_1730658	YTHDF2	4,76E-03	448	1,00E-04	17
ILMN_1794875	AGPAT9	1,32E-03	152	2,21E-04	29
ILMN_2231020	TMEM185B	6,20E-03	562	3,05E-04	36
ILMN_1713496	ST3GAL5	9,13E-03	735	3,93E-04	45
ILMN_2401641	ALDH3A2	7,17E-03	624	1,51E-03	120
ILMN_2405521	MTHFD2	8,02E-03	672	1,52E-03	122
ILMN_1700541	FBLN1	2,94E-03	310	1,64E-03	126
ILMN_1735192	COLEC11	5,26E-03	482	2,19E-03	155
ILMN_2206953	PLCL1	5,41E-03	494	2,28E-03	159

Lead Follicle slope

		Lead Follicle Size Slope		Data in Long Neg vs Pos	
PROBE_ID	SYMBOL	p0.05	Rank	p0.05	Rank
ILMN_3245057	**ASAP1**	3,24E-03	115	1,94E-06	1
ILMN_1690963	**ASAP1**	8,60E-03	290	2,87E-06	2
ILMN_2229032	**NME6**	6,84E-03	230	3,84E-06	3
ILMN_1727592	FRMD5	7,82E-03	269	3,86E-05	4
ILMN_2048700	ATAD2	8,04E-03	274	9,09E-05	13
ILMN_1767068	SMAD6	1,05E-03	47	1,00E-04	16
ILMN_1730658	YTHDF2	6,94E-07	1	1,00E-04	17
ILMN_2387090	CGGBP1	8,63E-03	291	1,21E-04	19
ILMN_1748272	MAP3K7IP3	8,48E-03	284	1,63E-04	22
ILMN_1794490	FLJ10781	2,10E-03	83	1,74E-04	26

Estrogen slope

		Estrogen slope		Data in Long Neg vs Pos	
PROBE_ID	SYMBOL	p0.05	Rank	p0.05	Rank
ILMN_1697286	SF3A1	4,00E-03	244	1,16E-03	98
ILMN_1748840	CALB2	1,09E-03	88	4,35E-03	250
ILMN_2353358	LGALS8	9,85E-03	441	8,44E-03	410
ILMN_2384857	DHRS2	6,14E-03	324	1,57E-02	669
ILMN_1809344	BTBD10	4,86E-03	277	1,67E-02	704
ILMN_1669113	ATF5	9,33E-03	424	2,19E-02	838
ILMN_1767337	SFXN5	2,17E-03	152	2,53E-02	922
ILMN_1794863	CAMK2N1	2,72E-03	138	2,59E-02	935
ILMN_2303166	MBNL3	4,82E-03	276	2,80E-02	992
ILMN_1798620	PQLC1	3,62E-03	226	3,13E-02	1082

Endometrium slope

		Endometrium Size Slope		Data in Long Neg vs Pos	
PROBE_ID	SYMBOL	p0.05	Rank	p0.05	Rank
ILMN_1727592	FRMD5	6,29E-04	99	3,86E-05	4
ILMN_1784300	TUBA4A	1,04E-03	132	4,73E-05	5
ILMN_1793522	PRKAB1	2,45E-03	270	6,51E-05	7
ILMN_1700384	KIAA1522	4,03E-03	418	8,51E-05	11
ILMN_1794885	FRMD5	6,46E-03	585	9,71E-05	15
ILMN_1728202	TMEM22	6,13E-03	562	1,21E-04	20
ILMN_1807044	UBAC1	2,91E-03	309	1,55E-04	21
ILMN_1748272	MAP3K7IP3	7,31E-03	640	1,63E-04	22
ILMN_1664772	ATP2B4	2,40E-03	268	1,72E-04	25
ILMN_1794490	FLJ10781	5,03E-03	481	1,74E-04	26

Pronuclei pattern

		Pronuceli pattern		Data in Long Neg vs Pos	
PROBE_ID	SYMBOL	p0.05	Rank	p0.05	Rank
ILMN_2392356	CTPS2	8,71E-03	194	9,49E-05	14
ILMN_2373556	SPAST	5,89E-03	134	3,90E-04	44
ILMN_1734288	DUSP18	5,25E-03	119	8,74E-04	78
ILMN_1693145	BUB3	4,40E-03	99	1,47E-03	118
ILMN_2405521	MTHFD2	9,00E-03	201	1,52E-03	122
ILMN_1659975	C1orf216	3,65E-03	79	1,62E-03	125
ILMN_3238740	CENPBD1	3,33E-03	72	2,22E-03	157
ILMN_2051731	DUSP5P	8,05E-03	178	2,36E-03	164
ILMN_1838313	-	2,78E-03	59	2,62E-03	180
ILMN_3243351	LOC646214	3,79E-04	7	2,84E-03	187

Supplementary Figure 6: Overlaps between expression data markers correlated with the clinical parameters on one hand and the differential expressed markers between long-protocol negative and positive markers on the other hand.
