# Supplementary material for: Stratifying Cumulus Cell Samples Based on Molecular Profiling to Help Resolve Biomarker Discrepancies and to Predict Oocyte Developmental Competence
Source: Int J Mol Sci. 2021 Jun 15;22(12):6377. doi: 10.3390/ijms22126377 (PMC8232172; doi:10.3390/ijms22126377)
Supplement: Supplementary file 1 [file ijms-22-06377-s001.zip › Suppl. Fig 7 best 10 cg meth markers and FSH responce PCA plot.pdf]

Supplementary Figure 7

A)

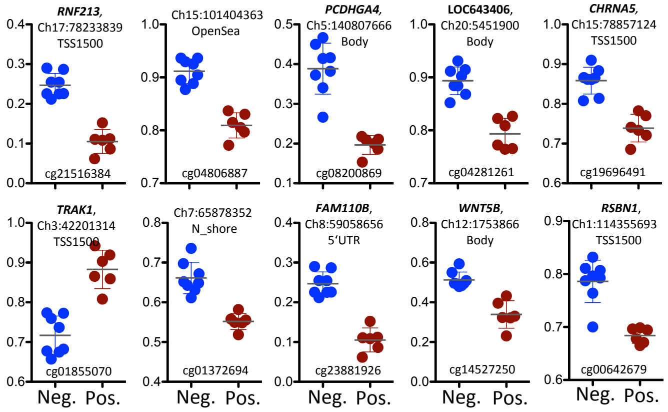

B)

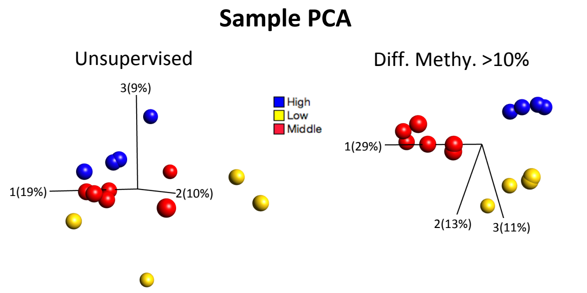

Supplementary Figure 7: differential DNA methylation between positive and negative groups of long protocol. **A)** top 10 differentially methylated CpG sites with significant p values (ttest <0.00001; Mann Whitney test =0.0007). The Illumina cg number, the name of the host gene if any, the chromosomal position and the relative position on the gene is shown. **B)** Sample PCA plot based on all the methylation data. The PCA on the left correspond to unsupervised PCA including all variables and the right one with variables of >10% differential methylation between positive and negative sample. The samples are labeled according to FSH response state in blue, red and yellow for high, middle and low FSH response.
