# Supplementary material for: Stratifying Cumulus Cell Samples Based on Molecular Profiling to Help Resolve Biomarker Discrepancies and to Predict Oocyte Developmental Competence
Source: Int J Mol Sci. 2021 Jun 15;22(12):6377. doi: 10.3390/ijms22126377 (PMC8232172; doi:10.3390/ijms22126377)
Supplement: Supplementary file 1 [file ijms-22-06377-s001.zip › Suppl. Fig 8 Genes Overlapping Variable plot V3.pdf]

Supplementary figure 8

A)

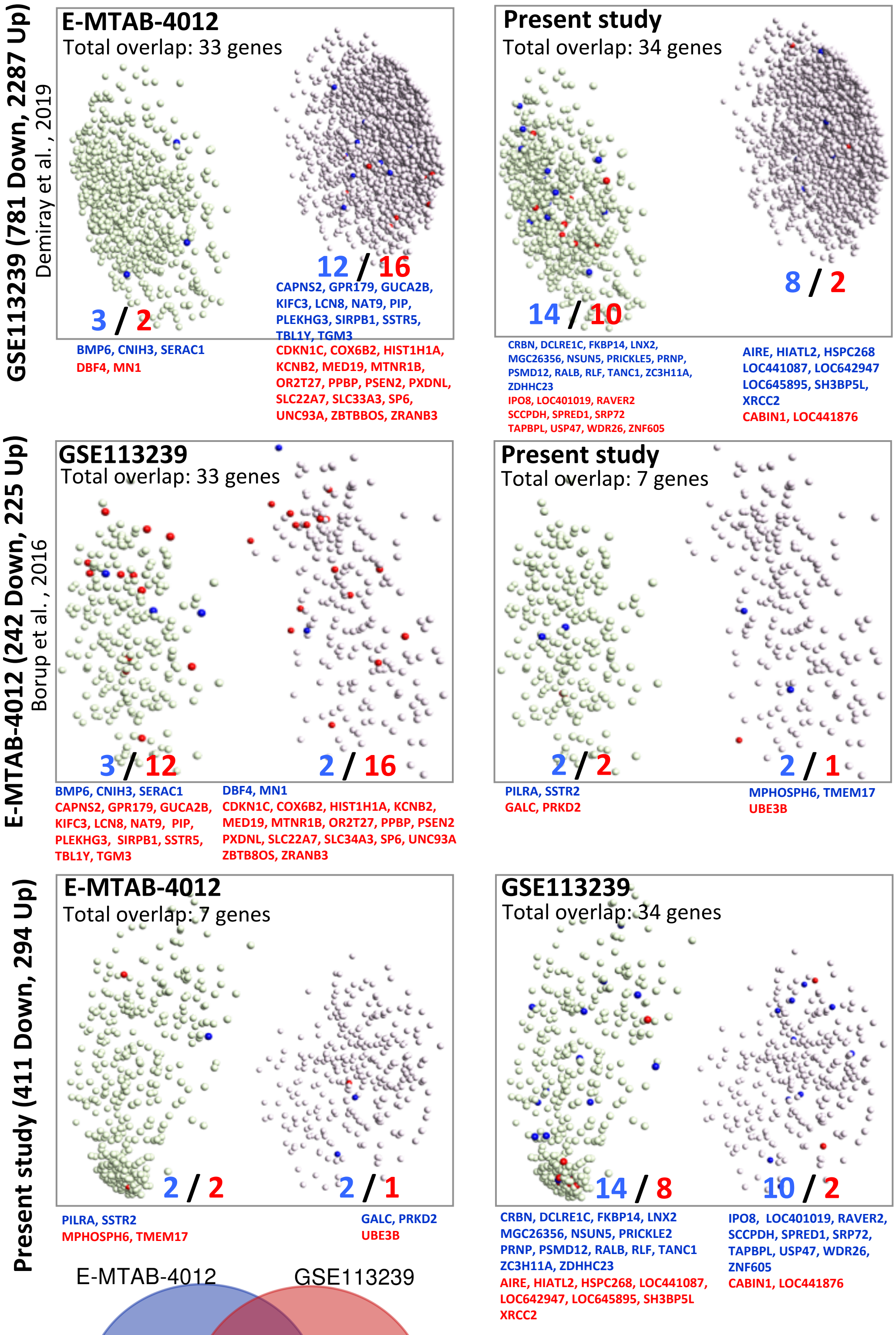

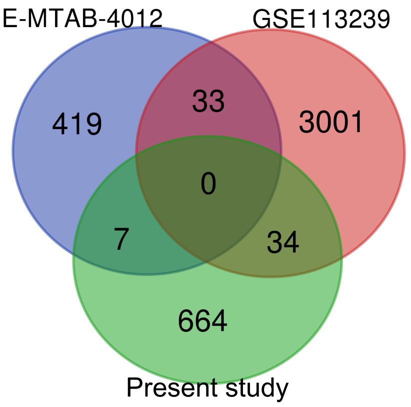

Supplementary Figure 8: Comparison of genomic data from two studies with the present study. **A)** Variable PCA plot of the three studies (green and pink represent under and over expressed in positive sample), each raw show one study and the overlaps with other studies are shown in blue (under expressed) and red (over expressed). The number of genes overlaps and the list of overlapped genes are also shown below each of the six comparisons. A Venn diagram showing intersection between all three studies is shown below.

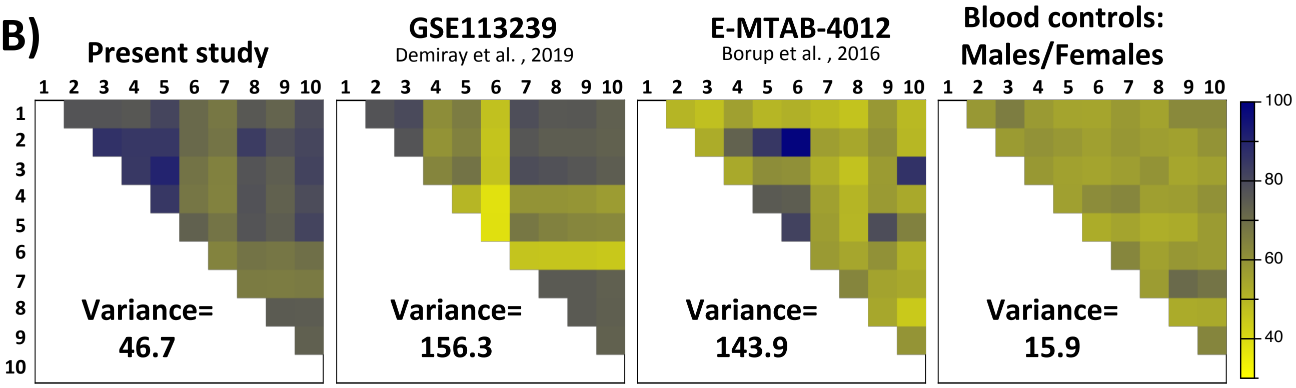

Supplementary figure 8 (Continued)

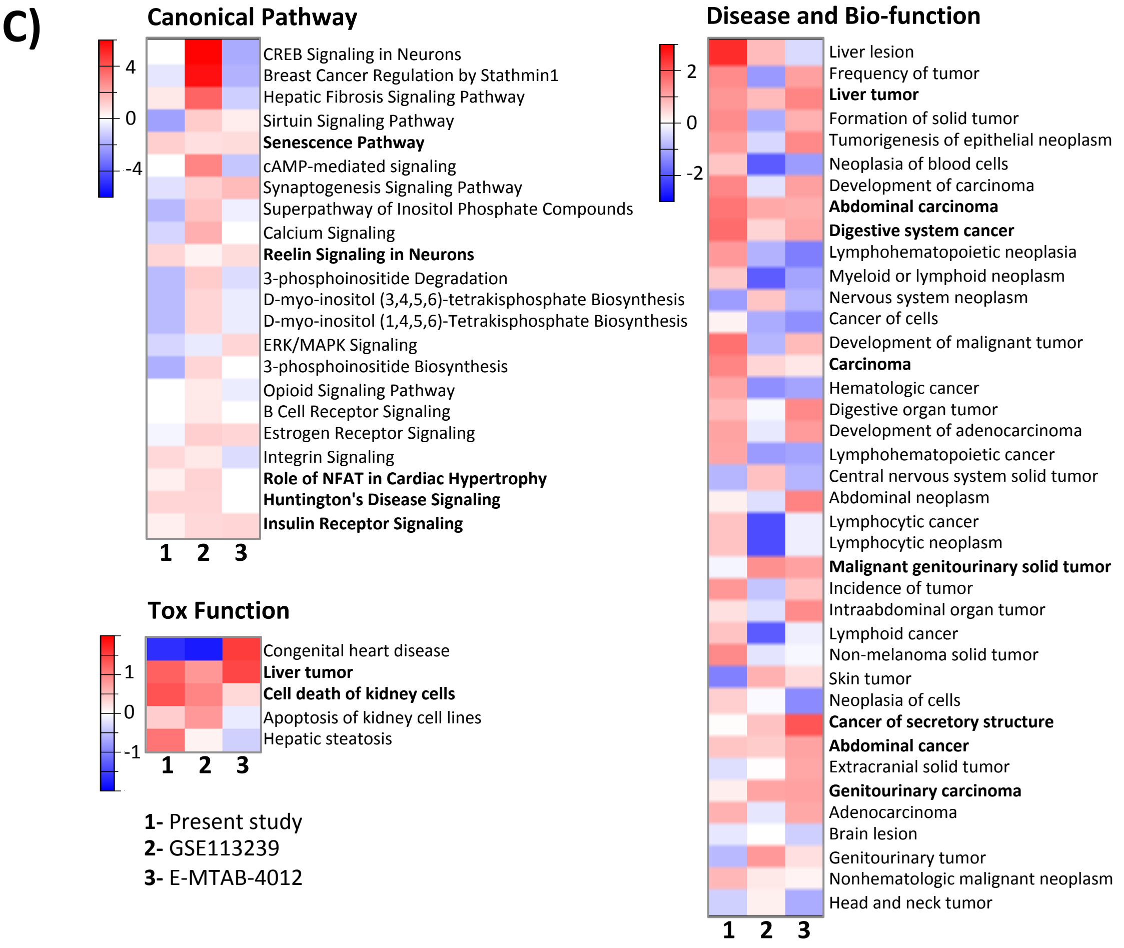

Supplementary figure 8 (continued). B) heat maps showing intersections (in %) in the results of 10 different testing between negative and positive samples when dropping a random 10% of the samples each time. The variance that estimate the heterogeneity of the samples is shown. A control set of homogenous blood samples testing male to female differences is shown to the right with lowest variance of 15.9%. **C)** comparison of activation/deactivation Z scores (as calculated by IPA) in the categories of canonical pathways, disease and bio-functions and toxicity function in the three studies (data for only pathways and function when predicted in all three data sets are shown). The pathway and the function showing comparable Z scores (and direction) are in bold.
