# Supplementary figures and images for: Stratifying Cumulus Cell Samples Based on Molecular Profiling to Help Resolve Biomarker Discrepancies and to Predict Oocyte Developmental Competence
Source: Int J Mol Sci. 2021 Jun 15;22(12):6377. doi: 10.3390/ijms22126377 (PMC8232172; doi:10.3390/ijms22126377)

Supplementary Figure 4

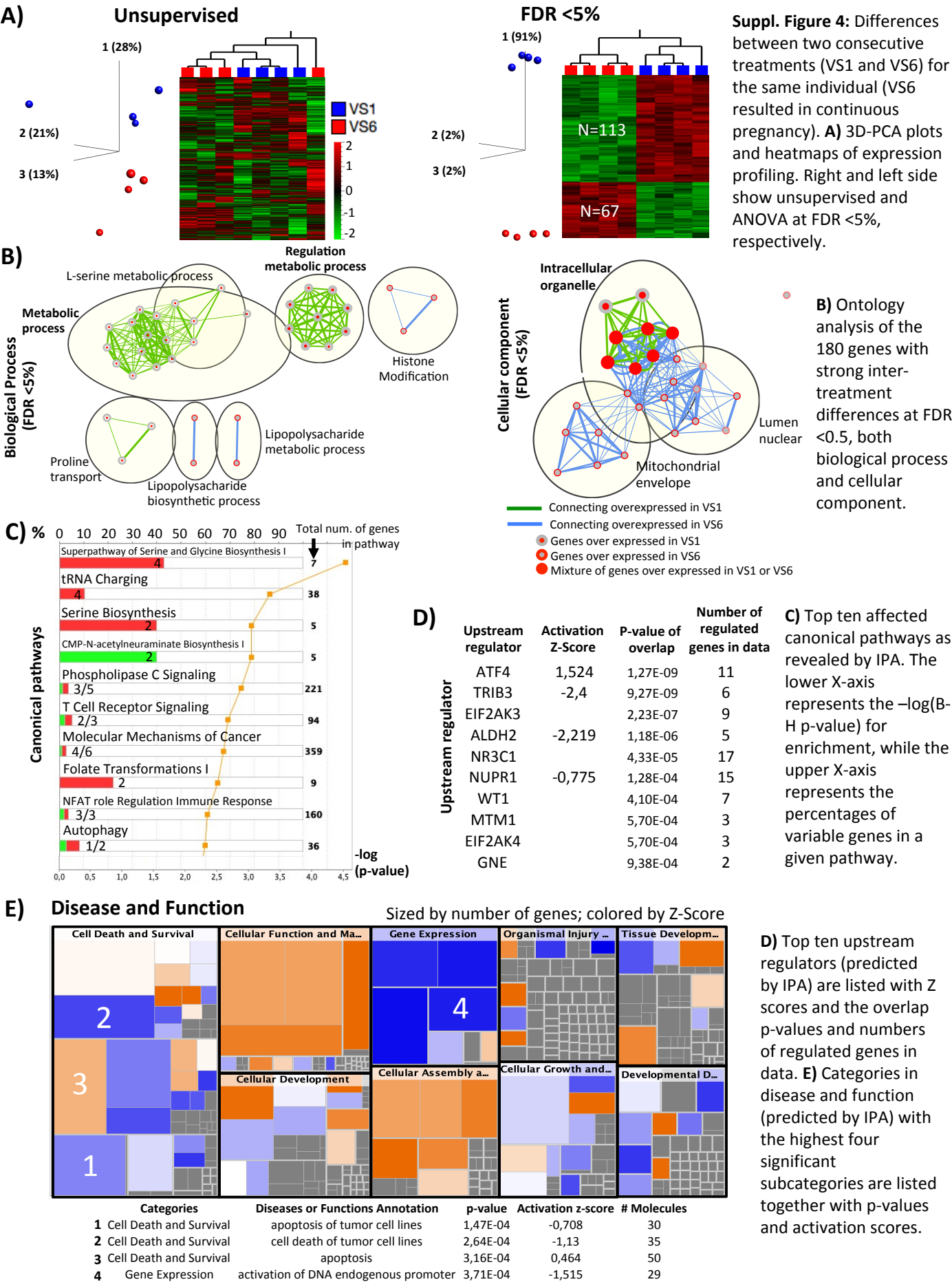

Supplement: Supplementary file 1 [file ijms-22-06377-s001.zip › Suppl. Fig 4 inter-treatment differences VS1 vs VS6 Ver6.pdf]
